# Supplementary material for: YTHDF2 facilitates aggresome formation via UPF1 in an m6A-independent manner
Source: Nat Commun. 2023 Oct 6;14:6248. doi: 10.1038/s41467-023-42015-w (PMC10558514; doi:10.1038/s41467-023-42015-w)
Supplement: Supplementary file 1 — Supplementary Information [file 41467_2023_42015_MOESM1_ESM.pdf]

Supplementary Information

YTHDF2 facilitates aggresome formation via UPF1 in an m^{6}A-independent manner

Hyun Jung Hwang[1,2], Tae Lim Park[1,2], Hyeong-In Kim[1,2], Yeonkyoung Park[1], Geunhee Kim[1], Chiyeol Song[1], Won-Ki Cho[1,*], and Yoon Ki Kim[1,*]

[1]Department of Biological Sciences, Korea Advanced Institute of Science and Technology, Daejeon 34141, Republic of Korea

[2]These authors contributed equally to this work.

*Correspondence: wonkicho@kaist.ac.kr; Tel.: +82 42-350-2647 (W.-K.C.)

*Correspondence: yk-kim@kaist.ac.kr; Tel.: +82 42-350-7927 (Y.K.K.)

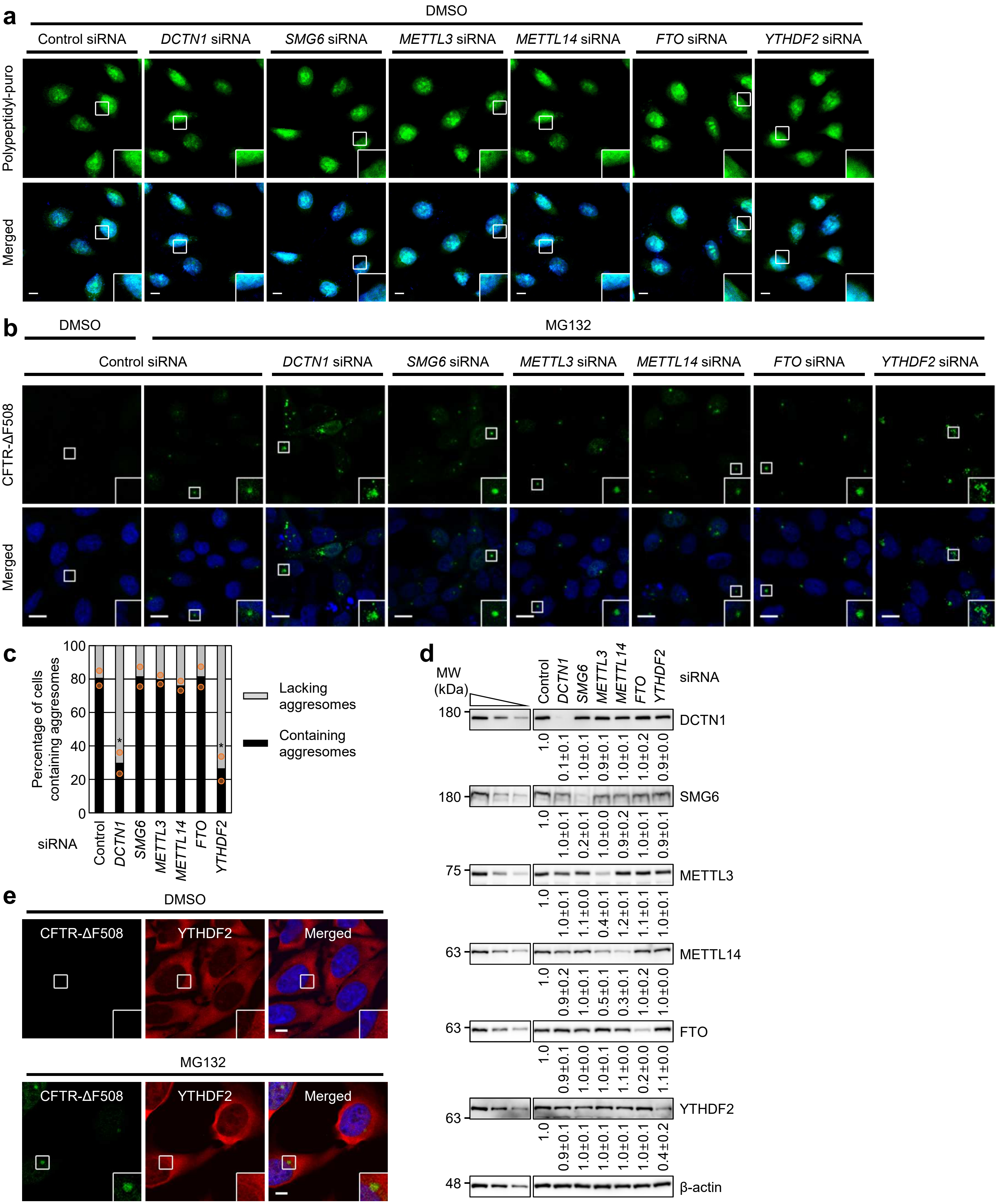
a
DMSO
Control siRNA
DCTN1 siRNA
SMG6 siRNA
METTL3 siRNA
METTL14 siRNA
FTO siRNA
YTHDF2 siRNA
Polypeptidyl-puro
Merged
b
DMSO
MG132
Control siRNA
DCTN1 siRNA
SMG6 siRNA
METTL3 siRNA
METTL14 siRNA
FTO siRNA
YTHDF2 siRNA
CFTR-ΔF508
Merged
c
Percentage of cells containing aggresomes
100
80
60
40
20
0
Lacking aggresomes
Containing aggresomes
siRNA
Control
DCTN1
SMG6
METTL3
METTL14
FTO
YTHDF2
d
MW (kDa)
Control
DCTN1
SMG6
METTL3
METTL14
FTO
YTHDF2
siRNA
180
DCTN1
1.0
0.1±0.1
1.0±0.1
0.9±0.1
1.0±0.1
1.0±0.2
0.9±0.0
180
SMG6
1.0
1.0±0.1
0.2±0.1
1.0±0.0
0.9±0.2
1.0±0.1
0.9±0.1
75
METTL3
1.0
1.0±0.1
1.1±0.0
0.4±0.1
1.2±0.1
1.1±0.1
1.0±0.1
63
METTL14
1.0
0.9±0.2
1.0±0.1
0.5±0.1
0.3±0.1
1.0±0.2
1.0±0.0
63
FTO
1.0
0.9±0.1
1.0±0.0
1.0±0.1
1.1±0.0
0.2±0.0
1.1±0.0
63
YTHDF2
1.0
0.9±0.1
1.0±0.1
1.0±0.1
1.0±0.1
1.0±0.1
0.4±0.2
48
β-actin
e
DMSO
CFTR-ΔF508
YTHDF2
Merged
MG132
CFTR-ΔF508
YTHDF2
Merged

Supplementary Fig. 1. Efficient formation of aggresomes containing misfolded polypeptides depends on YTHDF2. a, Immunostaining of polypeptidyl-puro aggresome under normal conditions. As performed in Fig. 1a, except that HeLa cells were treated with DMSO for 12 h and with puromycin for 1 h before fixation. All data represent at least three biologically independent replicates (n = 2). Scale bar, 10 μm. **b,** Immunostaining of the CFTR-ΔF508 aggresome. HeLa cells stably expressing CFTR-ΔF508 were transfected with the indicated siRNAs. Two days later, the cells were treated with either DMSO or MG132 for 12 h before fixation. The nuclei were visualized using DAPI (blue). Scale bar, 10 μm; n = 2. **c,** The percentage of cells either containing or lacking aggresomes of CFTR-ΔF508 in panel **b**. *, $P < 0.05$; n = 3. **d,** Western blots showing specific downregulation of the indicated proteins in Fig. 1a, and panels **a**, **b**. The total cell lysate was serially diluted three-fold and loaded in the three leftmost lanes to demonstrate that western blotting is semiquantitative in our experimental conditions. **e,** Immunostaining of the stably expressed CFTR-ΔF508 (green) aggresome and endogenous YTHDF2 (red). HeLa cells stably expressing CFTR-ΔF508 were treated with either DMSO or MG132 for 12 h before fixation; n = 2, Scale bar, 10 μm.

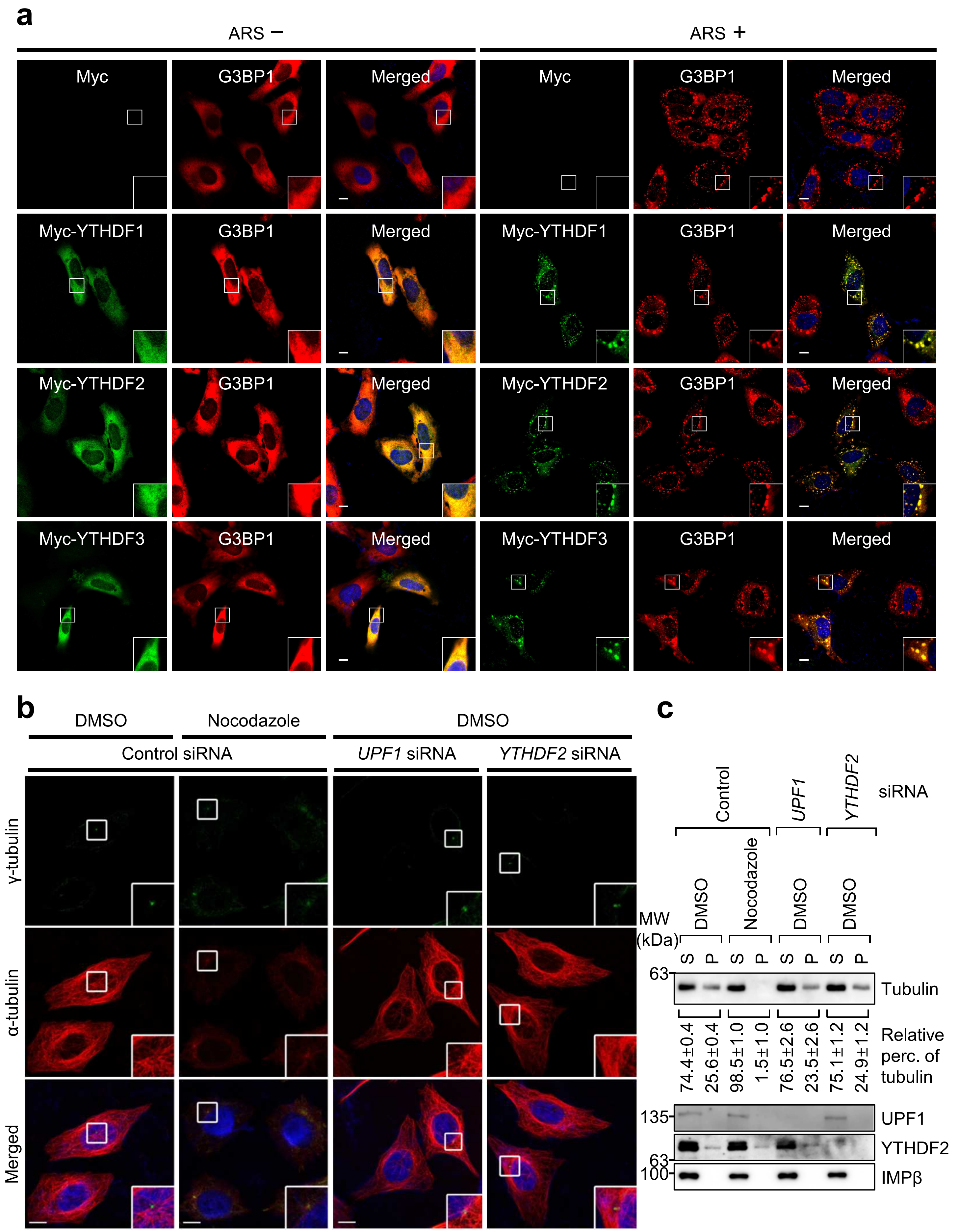
a
ARS −
ARS +
Myc
G3BP1
Merged
Myc-YTHDF1
Myc-YTHDF2
Myc-YTHDF3
b
DMSO
Nocodazole
Control siRNA
UPF1 siRNA
YTHDF2 siRNA
γ-tubulin
α-tubulin
Merged
c
Control
UPF1
YTHDF2
siRNA
MW (kDa)
S
P
63
Tubulin
74.4±0.4
25.6±0.4
98.5±1.0
1.5±1.0
76.5±2.6
23.5±2.6
75.1±1.2
24.9±1.2
Relative perc. of tubulin
135
UPF1
YTHDF2
100
IMPβ

Supplementary Fig. 2. YTHDF1–3 have a comparable ability to be localized to stress granules. a, Immunostaining of Myc or Myc-YTHDF proteins (green) with endogenous G3BP1 (red). HeLa cells were transfected with Myc or Myc-YTHDF proteins. Two days later, the cells were treated with sodium arsenite for 1 h before fixation. The nuclei were visualized using DAPI (blue). Scale bar, 10 μm; n = 2. **b,** Immunostaining of α-tubulin (red) and γ-tubulin (green). HeLa cells were treated with control, *UPF1*, or *YTHDF2* siRNAs. Two days later, the cells were treated with either DMSO or nocodazole for 12 h before fixation. The nuclei were visualized using DAPI (blue). Scale bar, 10 μm; n = 2. **c,** Relative changes in the levels of microtubule and free α/β-tubulin upon downregulation of UPF1 or YTHDF2. As performed in panel **b**, except that the cell extracts were lysed in a microtubule stabilization buffer, and the lysed extracts were subjected to an ultracentrifugation to separate polymerized microtubules (P, pellet fraction) from non-polymerized α/β-tubulin (S, supernatant fraction). Protein samples in P and S were analyzed by western blotting. The band intensities were quantitated, and the relative levels of α/β-tubulin in P and S were calculated and presented as a percentage. n = 3.

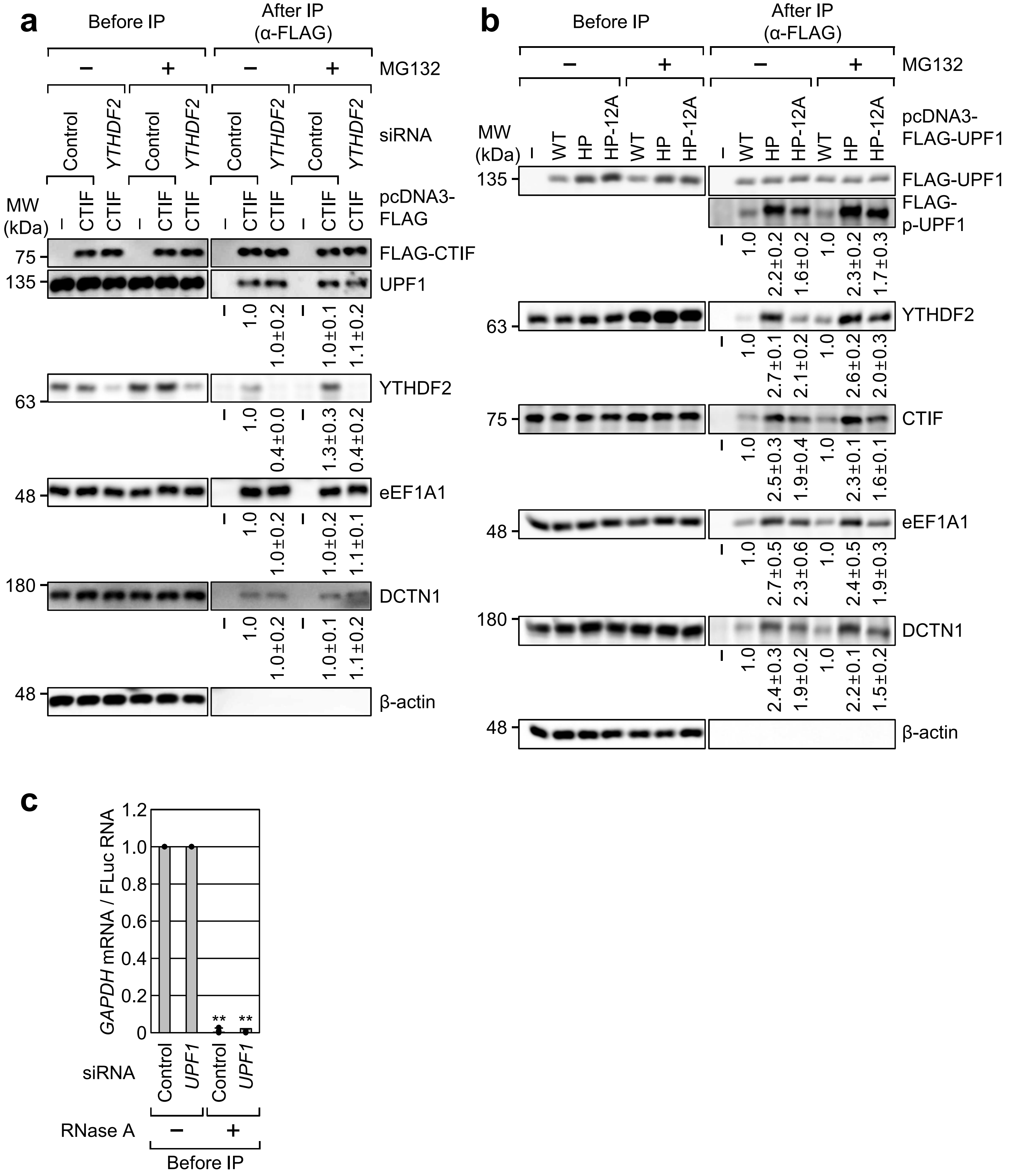

a
Before IP
After IP (α-FLAG)
MG132
siRNA
Control
YTHDF2
pcDNA3-FLAG
CTIF
MW (kDa)
FLAG-CTIF
UPF1
YTHDF2
eEF1A1
DCTN1
β-actin
75
135
63
48
180
1.0
1.0±0.2
1.0±0.1
1.1±0.2
0.4±0.0
1.3±0.3
0.4±0.2
1.1±0.1
b
Before IP
After IP (α-FLAG)
MG132
pcDNA3-FLAG-UPF1
WT
HP
HP-12A
FLAG-UPF1
FLAG-p-UPF1
2.2±0.2
1.6±0.2
2.3±0.2
1.7±0.3
2.7±0.1
2.1±0.2
2.6±0.2
2.0±0.3
2.5±0.3
1.9±0.4
2.3±0.1
1.6±0.1
2.7±0.5
2.3±0.6
2.4±0.5
1.9±0.3
2.4±0.3
1.9±0.2
2.2±0.1
1.5±0.2
CTIF
c
GAPDH mRNA / FLuc RNA
0
0.2
0.4
0.6
0.8
1.0
1.2
**
UPF1
RNase A
Before IP

Supplementary Fig. 3. Hyperphosphorylated UPF1 is more strongly associated with the YTHDF2 and CED complex. a, IP of FLAG-CTIF using extracts of HEK293T cells either undepleted or depleted of YTHDF2. Cells were transfected with *YTHDF2* siRNA. One day later, the cells were re-transfected with a plasmid expressing FLAG or FLAG-CTIF. The cells were treated with either DMSO or MG132 for 12 h before harvesting. The relative intensities of proteins after IP of FLAG-CTIF in the undepleted cells were arbitrarily set to 1.0; n = 3. **b,** IP of FLAG-UPF1-WT or its variants using HEK293T cell extracts. Cells were transfected with FLAG-UPF1-WT or its variants. Two days later, the cells were treated with either DMSO or MG132 for 12 h before harvesting. The relative intensities of proteins after IP of FLAG-UPF1-WT in cells treated with either DMSO or MG132 were arbitrarily set to 1.0; n = 3. **c**, Validation of a proper removal of endogenous RNAs after RNase A treatment in Fig. 3c. Total-cell RNAs before or after treatment with RNase A were subjected to qRT-PCRs using specific oligonucleotides to amplify endogenous *GAPDH* mRNA and FLuc RNA (spike RNA). **, $P < 0.01$; n = 3.

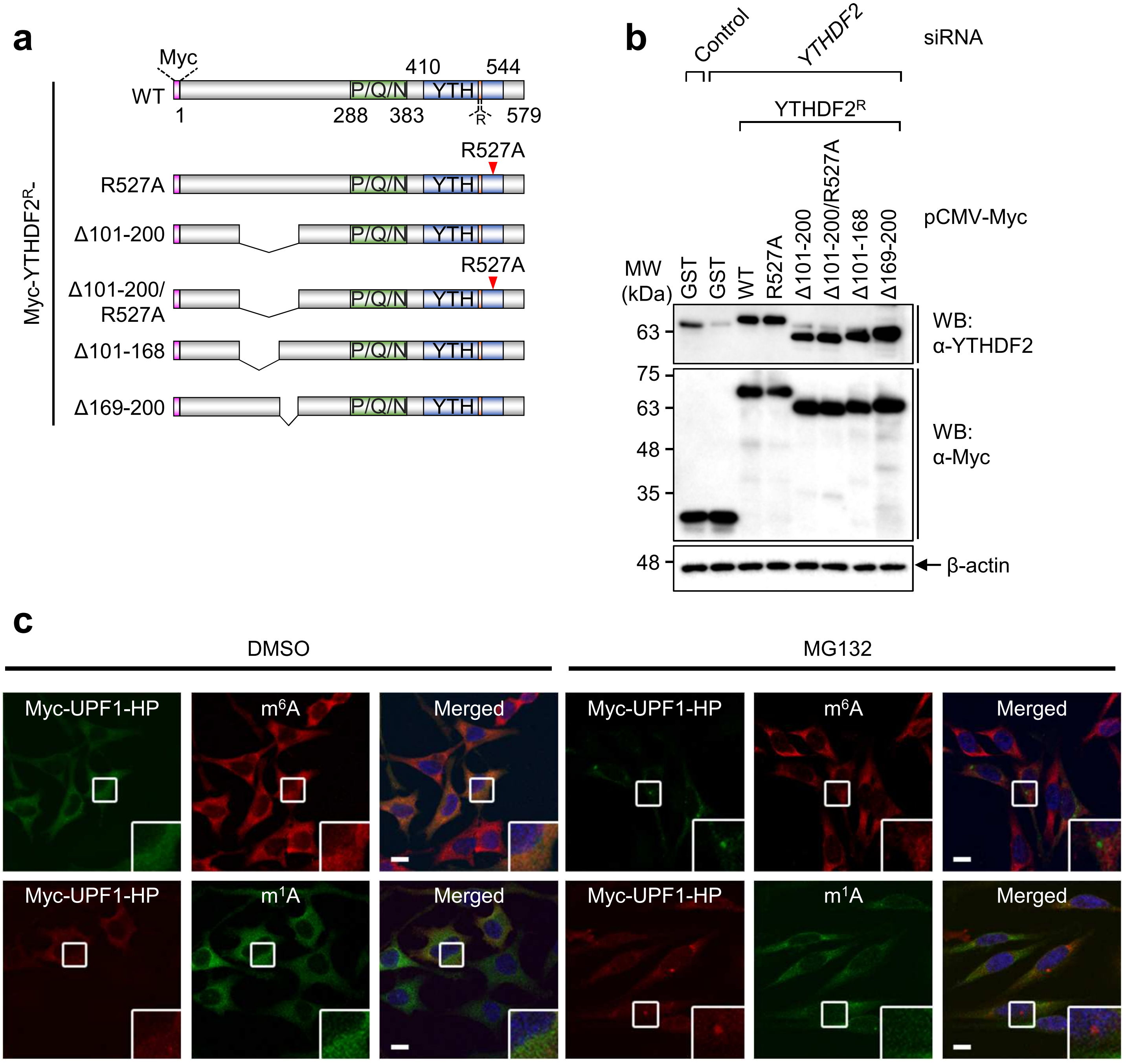

Supplementary Fig. 4. Validation of a specific downregulation of endogenous YTHDF2 and comparable expression levels of Myc-YTHDF2^{R} WT and variants. a, Schematic diagram of Myc-YTHDF2^{R} and its variants used in this study. Mutated residues are indicated by red arrowheads. **b,** Western blot showing selective downregulation of endogenous YTHDF2 and comparable expression levels of Myc-YTHDF2^{R} (WT and its variants) in Fig. 4b,c. **c,** Immunostaining of Myc-UPF1-HP (used as an aggresomal marker) and either m^{6}A or m^{1}A before or after MG132 treatment. HeLa cells were transiently transfected with the plasmid expressing Myc-UPF1-HP. Two days later, the cells were treated with DMSO or MG132 for 12 h before fixation. Scale bar, 10 μm; n = 2.

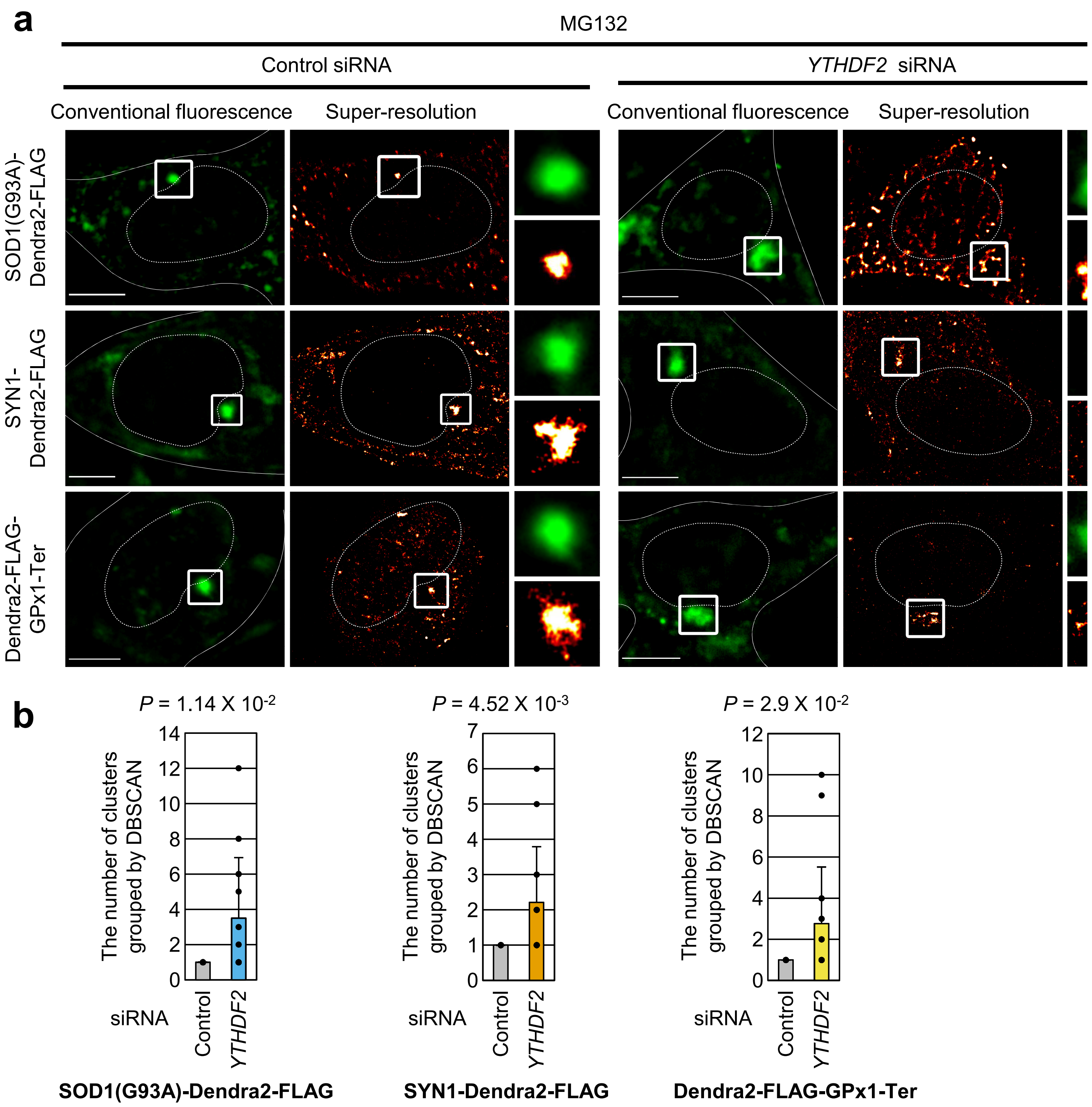

Supplementary Fig. 5. Aggresome detected by super-resolution microscopy. a, Representative images HeLa cells with aggresomes detected using either conventional fluorescence microscopy or super-resolut microscopy. Scale bar, 10 μm. **b,** Number of clusters (aggregates) detected in cells expressing e misfolding-prone protein. After cells were treated with MG132 for 12 h, each aggresome was imaged us super-resolution microscopy. Each cluster was determined using DBSCAN (n = 13–17).

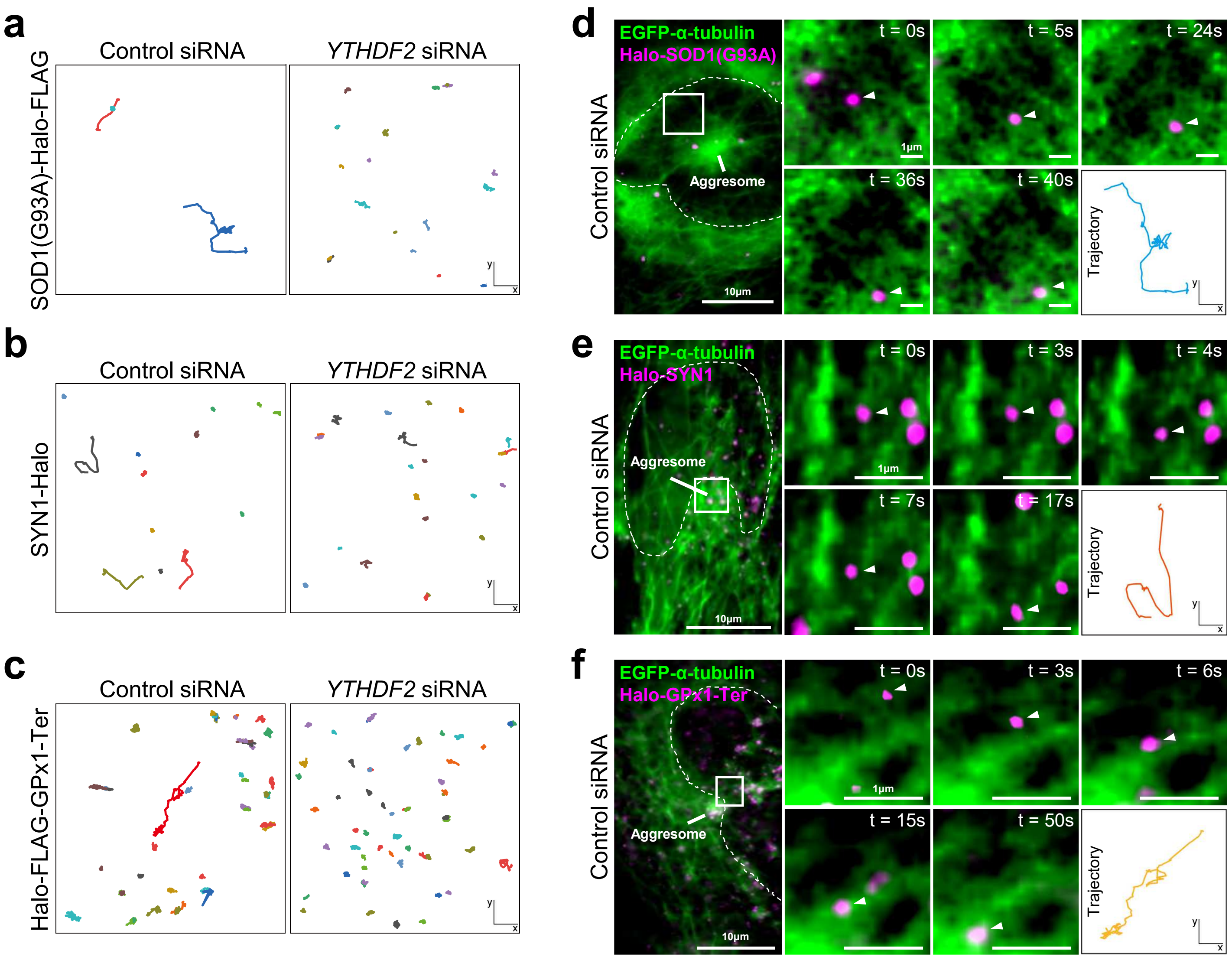

Supplementary Figure 6. Example traces of misfolded polypeptides associated with the microtubule network in the presence of YTHDF2. a–c, Trajectories of misfolded polypeptides in a field of view were represented for control siRNA-treated or YTHDF2 siRNA-treated conditions. **d–f.** Representative images of single-particle tracking of misfolded polypeptides on the microtubules (left panels; nucleus is represented with a dotted line). HeLa cells were transiently expressed with HaloTag-labeled misfolding-prone proteins and EGFP-labeled α-tubulin to visualize the microtubules. Misfolded polypeptides were labeled with JF646-HaloTag ligand. Zoomed-in time-lapse images corresponding to the region specified by a white box show the movement of misfolded polypeptides along microtubules under MG132-treated conditions. The overall trajectory of each misfolded polypeptide (a white arrowhead) in panels **d, e, f** is represented in the right bottom panel.

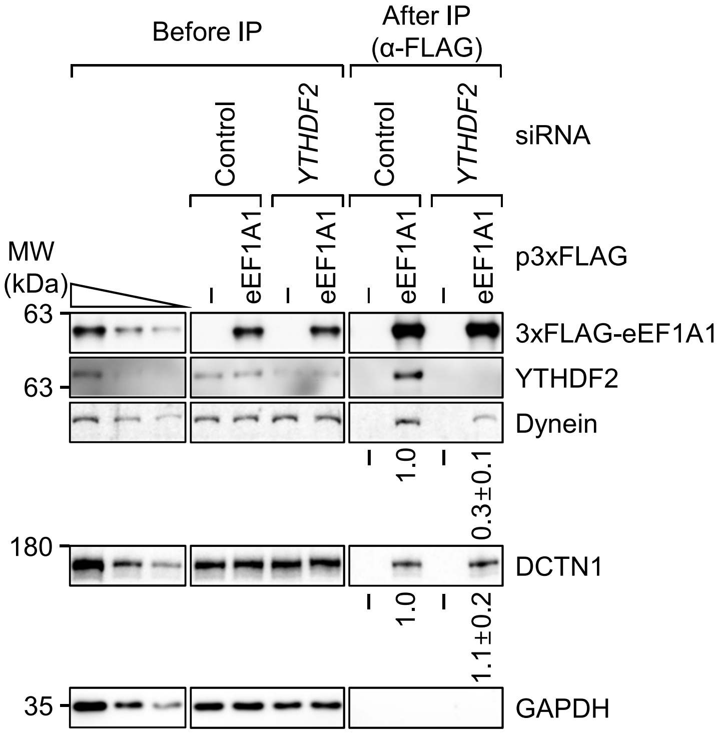

Supplementary Fig. 7. YTHDF2 downregulation inhibits the association between eEF1A1 and dynein motor proteins. As performed in Fig. 8a,b, except that the MG132-treated cells transiently expressing 3xFLAG-eEF1A1 were subjected to IPs using α-FLAG antibody. The cell lysate before IP was serially diluted three-fold and loaded in the three leftmost lanes to demonstrate that western blotting is semiquantitative in our experimental conditions; n = 3.
