# Supplementary material for: YTHDF2 facilitates aggresome formation via UPF1 in an m6A-independent manner
Source: Nat Commun. 2023 Oct 6;14:6248. doi: 10.1038/s41467-023-42015-w (PMC10558514; doi:10.1038/s41467-023-42015-w)
Supplement: Supplementary file 3 — Description of Additional Supplementary Files [file 41467_2023_42015_MOESM3_ESM.pdf]

Description of Additional Supplementary Files

File Name: Supplementary Movie 1

Description: Representative movie of single-particle tracking of SOD1(G93A) labeled with the JF646-HaloTag ligand under control siRNA-treated (left) or *YTHDF2* siRNA-treated (right) conditions. Scale bar, 2 μm.

File Name: Supplementary Movie 2

Description: Representative movie of single-particle tracking of SYN1 labeled with the JF646-HaloTag ligand under control siRNA-treated (left) or *YTHDF2* siRNA-treated (right) conditions. Scale bar, 2 μm.

File Name: Supplementary Movie 3

Description: Representative movie of single-particle tracking of GPx1-Ter labeled with the JF646-HaloTag ligand under control siRNA-treated (left) or *YTHDF2* siRNA-treated (right) conditions. Scale bar, 2 μm.

File Name: Supplementary Movie 4

Description: Representative movie of dual-color single-particle tracking of YTHDF2 and SYN1. YTHDF2 was labeled with the TMR-SnapTag ligand, and SYN1 was labeled with the JF646-HaloTag ligand. Scale bar, 1 μm.

File Name: Supplementary Movie 5

Description: Movie of single-particle tracking on the microtubules. Representative movie of single-particle tracking of SOD1(G93A) under control siRNA-treated conditions. HeLa cells were co-transfected with plasmids expressing EGFP-tagged α-tubulin and HaloTag-fused misfolding-prone SOD1(G93A). Both microtubules and misfolded polypeptide were simultaneously imaged to observe single-particles moving on the microtubules in the presence of MG132. Each of misfolded polypeptides was labeled with the JF646-HaloTag ligands. Scale bar, 1 μm.

File Name: Supplementary Movie 6

Description: Movie of single-particle tracking on the microtubules. As performed in Supplementary Movie 5, except that HeLa cells were co-transfected with plasmids expressing EGFP-tagged α-tubulin and HaloTag-fused misfolding-prone SYN1.

File Name: Supplementary Movie 7

Description: Movie of single-particle tracking on the microtubules. As performed in Supplementary Movie 5, except that HeLa cells were co-transfected with plasmids expressing EGFP-tagged α-tubulin and HaloTag-fused misfolding-prone GPx1-Ter.
