# Supplementary material for: YTHDF2 facilitates aggresome formation via UPF1 in an m6A-independent manner
Source: Nat Commun. 2023 Oct 6;14:6248. doi: 10.1038/s41467-023-42015-w (PMC10558514; doi:10.1038/s41467-023-42015-w)
Supplement: Supplementary file 11 — Reporting Summary [file 41467_2023_42015_MOESM11_ESM.pdf]

nature portfolio

Corresponding author(s): Yoon Ki Kim

Last updated by author(s): Sep 6, 2023

Reporting Summary

Nature Portfolio wishes to improve the reproducibility of the work that we publish. This form provides structure for consistency and transparency in reporting. For further information on Nature Portfolio policies, see our Editorial Policies and the Editorial Policy Checklist.

Statistics

For all statistical analyses, confirm that the following items are present in the figure legend, table legend, main text, or Methods section.

n/a	Confirmed	
☐	☒	The exact sample size (*n*) for each experimental group/condition, given as a discrete number and unit of measurement
☐	☒	A statement on whether measurements were taken from distinct samples or whether the same sample was measured repeatedly
☐	☒	The statistical test(s) used AND whether they are one- or two-sided<br>*Only common tests should be described solely by name; describe more complex techniques in the Methods section.*
☐	☒	A description of all covariates tested
☐	☒	A description of any assumptions or corrections, such as tests of normality and adjustment for multiple comparisons
☐	☒	A full description of the statistical parameters including central tendency (e.g. means) or other basic estimates (e.g. regression coefficient) AND variation (e.g. standard deviation) or associated estimates of uncertainty (e.g. confidence intervals)
☐	☒	For null hypothesis testing, the test statistic (e.g. *F*, *t*, *r*) with confidence intervals, effect sizes, degrees of freedom and *P* value noted<br>*Give P values as exact values whenever suitable.*
☒	☐	For Bayesian analysis, information on the choice of priors and Markov chain Monte Carlo settings
☐	☒	For hierarchical and complex designs, identification of the appropriate level for tests and full reporting of outcomes
☒	☐	Estimates of effect sizes (e.g. Cohen's *d*, Pearson's *r*), indicating how they were calculated

Our web collection on statistics for biologists contains articles on many of the points above.

Software and code

Policy information about availability of computer code

Data collection	NIS elements AR V5.4 (Nikon) was used to acquire images from super-resolution microscopy and from dual-color single-particle tracking. LSM 510 Meta software (Carl Zeiss), LSM 700 software (Carl Zeiss), and Zeiss LSM Image Browser and Zen 2.1 (black; Carl Zeiss) were used to acquire confocal images.
Data analysis	ImageJ (v1.53c) software was used for analyzing super-resolution images and for exporting coordinate data from single particle tracking (TrackMate plugin). Matlab (R2021b) was used for analyzing circularity and protein localization of misfolding-prone proteins within aggresome and for calculating MSD, alpha values, D values (diffusion coefficient), and displacement.

For manuscripts utilizing custom algorithms or software that are central to the research but not yet described in published literature, software must be made available to editors and reviewers. We strongly encourage code deposition in a community repository (e.g. GitHub). See the Nature Portfolio guidelines for submitting code & software for further information.

Data

Policy information about availability of data

All manuscripts must include a data availability statement. This statement should provide the following information, where applicable:

- Accession codes, unique identifiers, or web links for publicly available datasets
- A description of any restrictions on data availability
- For clinical datasets or third party data, please ensure that the statement adheres to our policy

All datasets are available in manuscript, figures, and source data file.

Human research participants

Policy information about studies involving human research participants and Sex and Gender in Research.

Reporting on sex and gender	N/A
Population characteristics	N/A
Recruitment	N/A
Ethics oversight	N/A

Note that full information on the approval of the study protocol must also be provided in the manuscript.

Field-specific reporting

Please select the one below that is the best fit for your research. If you are not sure, read the appropriate sections before making your selection.

☒ Life sciences ☐ Behavioural & social sciences ☐ Ecological, evolutionary & environmental sciences

For a reference copy of the document with all sections, see nature.com/documents/nr-reporting-summary-flat.pdf

Life sciences study design

All studies must disclose on these points even when the disclosure is negative.

Sample size	All experiments were performed at least two times independently. More than 50 cells in each experiment were counted to quantify relative distribution of aggresome in confocal data in three independent biological replicates. Super-resolution imaging was performed on at least 13 cells from two independent experiments and analyzed for circularity and protein localization. 20 cells from three independent biological replicates were analyzed for calculating MSD, alpha values, D values (diffusion coefficient), and displacement from single particle tracking data.
Data exclusions	No data was excluded.
Replication	Completely independent experiments were performed at least two replicates.
Randomization	Randomization was not relevant because there was no experimental groups in this study.
Blinding	Confocal analysis for quantitating relative distribution of aggresome was performed in blind at least two people

Reporting for specific materials, systems and methods

We require information from authors about some types of materials, experimental systems and methods used in many studies. Here, indicate whether each material, system or method listed is relevant to your study. If you are not sure if a list item applies to your research, read the appropriate section before selecting a response.

Materials & experimental systems

n/a	Involved in the study
☐	☒ Antibodies
☐	☒ Eukaryotic cell lines
☒	☐ Palaeontology and archaeology
☒	☐ Animals and other organisms
☒	☐ Clinical data
☒	☐ Dual use research of concern

Methods

n/a	Involved in the study
☒	☐ ChIP-seq
☒	☐ Flow cytometry
☒	☐ MRI-based neuroimaging

Antibodies

Antibodies used

FLAG (DYKDDDDK; 14793, Cell Signaling Technology or A8592, Sigma-Aldrich)
Myc (9E10; OP10L, Calbiochem or 2272, Cell Signaling Technology)
FTO (ab124892, Abcam)
GFP (sc-9996, Santa Cruz Biotechnology)
DCTN1 (p150glued; 610474, BD Biosciences)
eEF1A1 (CBP-KK1; EF1α; 05-235, Merck Millipore)
YTHDF1 (17479-1-AP, Proteintech)
YTHDF2 (24744-1-AP, Proteintech)
YTHDF3 (sc-377119, Santa Cruz Biotechnology)
METTL3 (15073-1-AP, Proteintech)
METTL14 (HPA038002, Sigma-Aldrich)
p-(S/T)Q ATM/ATR substrate (2851, Cell Signaling Technology)
m6A (#202003, Synaptic Systems)
m1A (D345-3, MBL)
puromycin (12D10; MABE343, Merck Millipore)
β-actin (A5441, Sigma-Aldrich)
GAPDH (LF-PA0212, AbFrontier)
α-tubulin (sc-53030, Santa Cruz Biotechnology)
γ-tubulin (sc-17788, Santa Cruz Biotechnology)
dynein (sc-9115, Santa Cruz Biotechnology)
G3BP1 (13057-2-AP, Proteintech)

Alexa Fluor 488 goat α-mouse IgG (A-11017, Invitrogen)
rhodamine-conjugated goat α-rabbit IgG (31670, Invitrogen)

Validation

All antibodies used in this study were purchased from commercial manufacturers. The manufacturers provide information about the validation in their website.

Eukaryotic cell lines

Policy information about cell lines and Sex and Gender in Research

Cell line source(s)

HEK293T and HeLa cells are purchased from ATCC. HeLa cells stably expressing CFTR-ΔF508 was described in previous paper (Park et al., 2017. Naturecommunications)

Authentication

HeLa cells stably expressing CFTR-ΔF508 was verified by confocal analysis with GFP antibody (sc-9996, Santa Cruz Biotechnology).

Mycoplasma contamination

Cell lines were tested negative for mycoplasma contamination using MycoAlert PLUS Mycoplasma detection kit.

Commonly misidentified lines
(See ICLAC register)

No commonly misidentified lines were utilized.
