# Supplementary figures and images for: YTHDF2 facilitates aggresome formation via UPF1 in an m6A-independent manner (part 1 of 2)
Source: Nat Commun. 2023 Oct 6;14:6248. doi: 10.1038/s41467-023-42015-w (PMC10558514; doi:10.1038/s41467-023-42015-w)

Figure 3

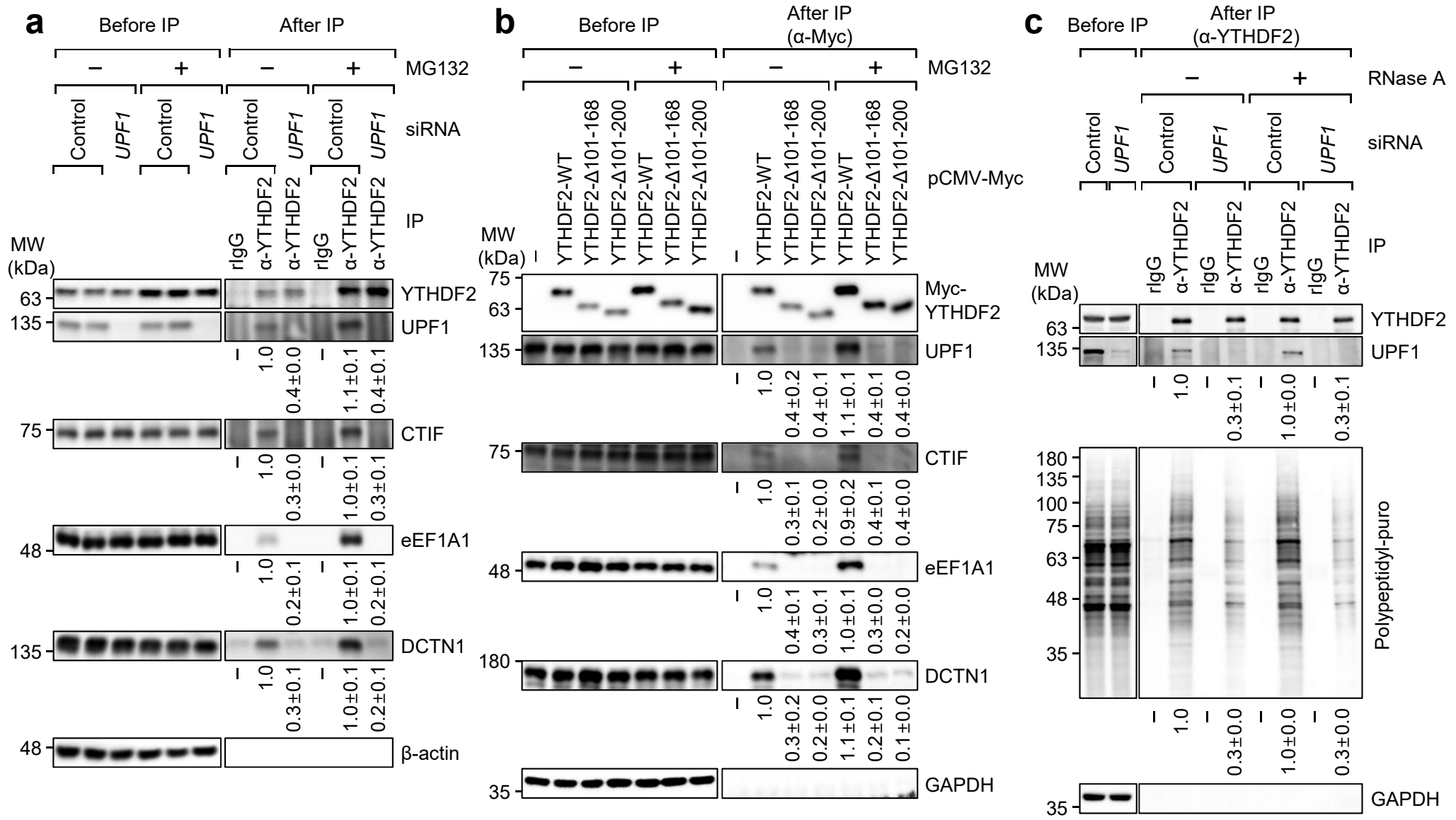

Supplement: Supplementary file 12 — Source Data [file 41467_2023_42015_MOESM12_ESM.pdf]

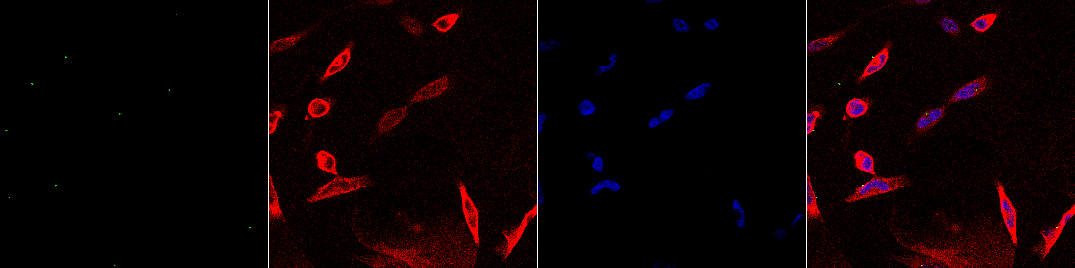

Supplement: Supplementary file 13 — Figure 3 (OLD) [file 41467_2023_42015_MOESM13_ESM.zip › Figure 4/Figure4b/WT.jpg]

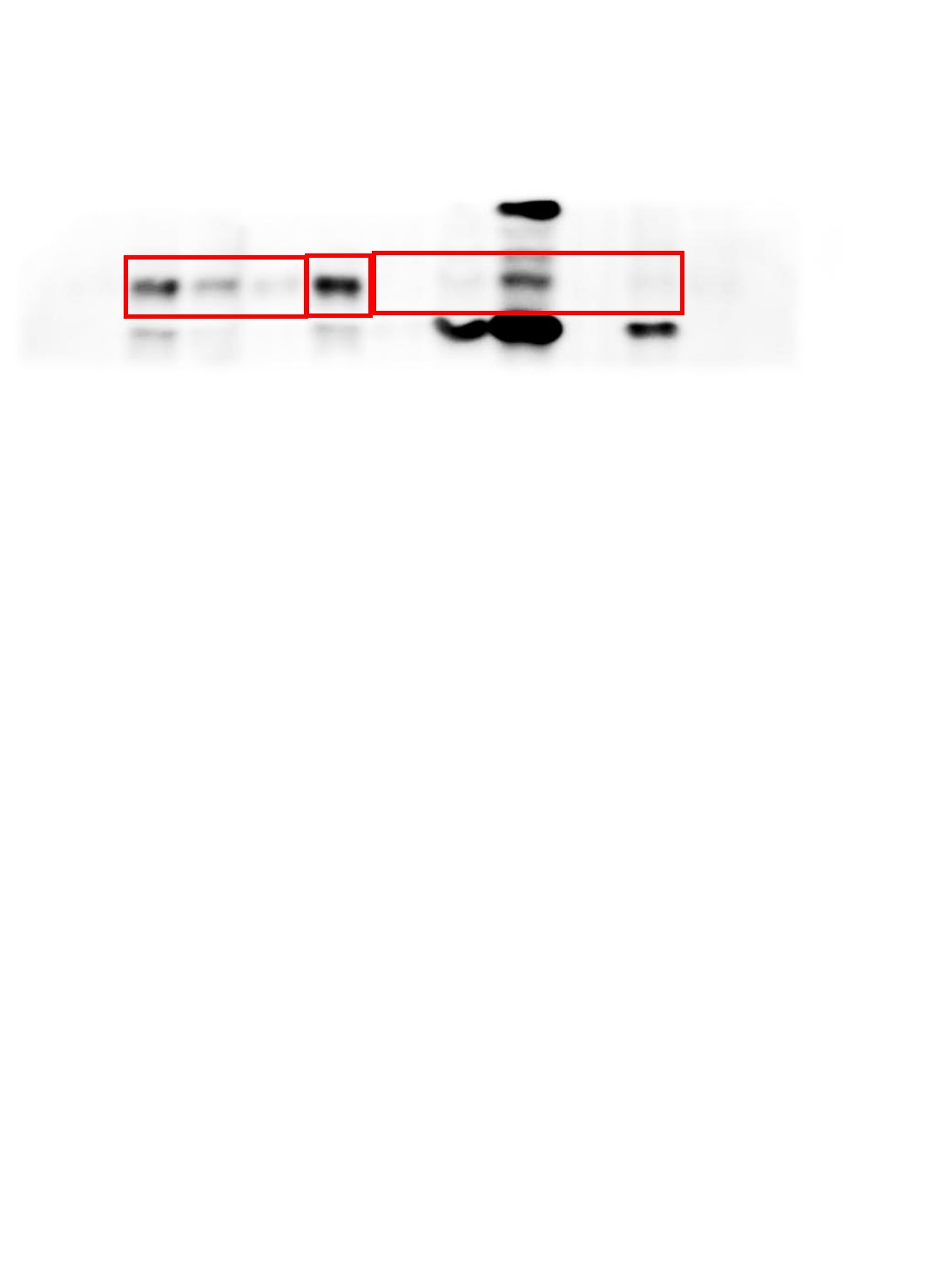

Supplement: Supplementary file 13 — Figure 3 (OLD) [file 41467_2023_42015_MOESM13_ESM.zip › Figure 2/Figure2f/CTIF.jpg]

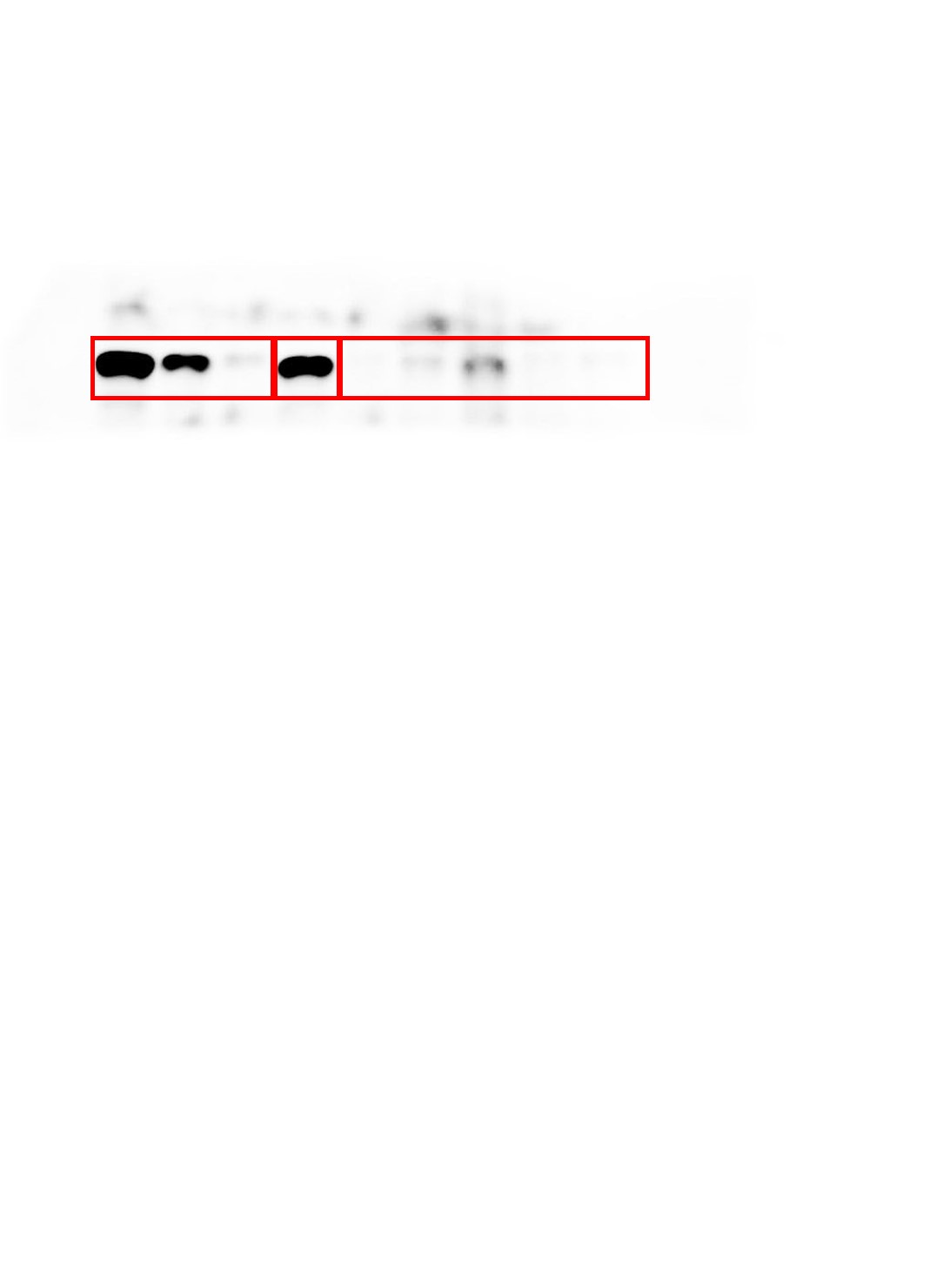

Supplement: Supplementary file 13 — Figure 3 (OLD) [file 41467_2023_42015_MOESM13_ESM.zip › Figure 2/Figure2f/UPF1.jpg]

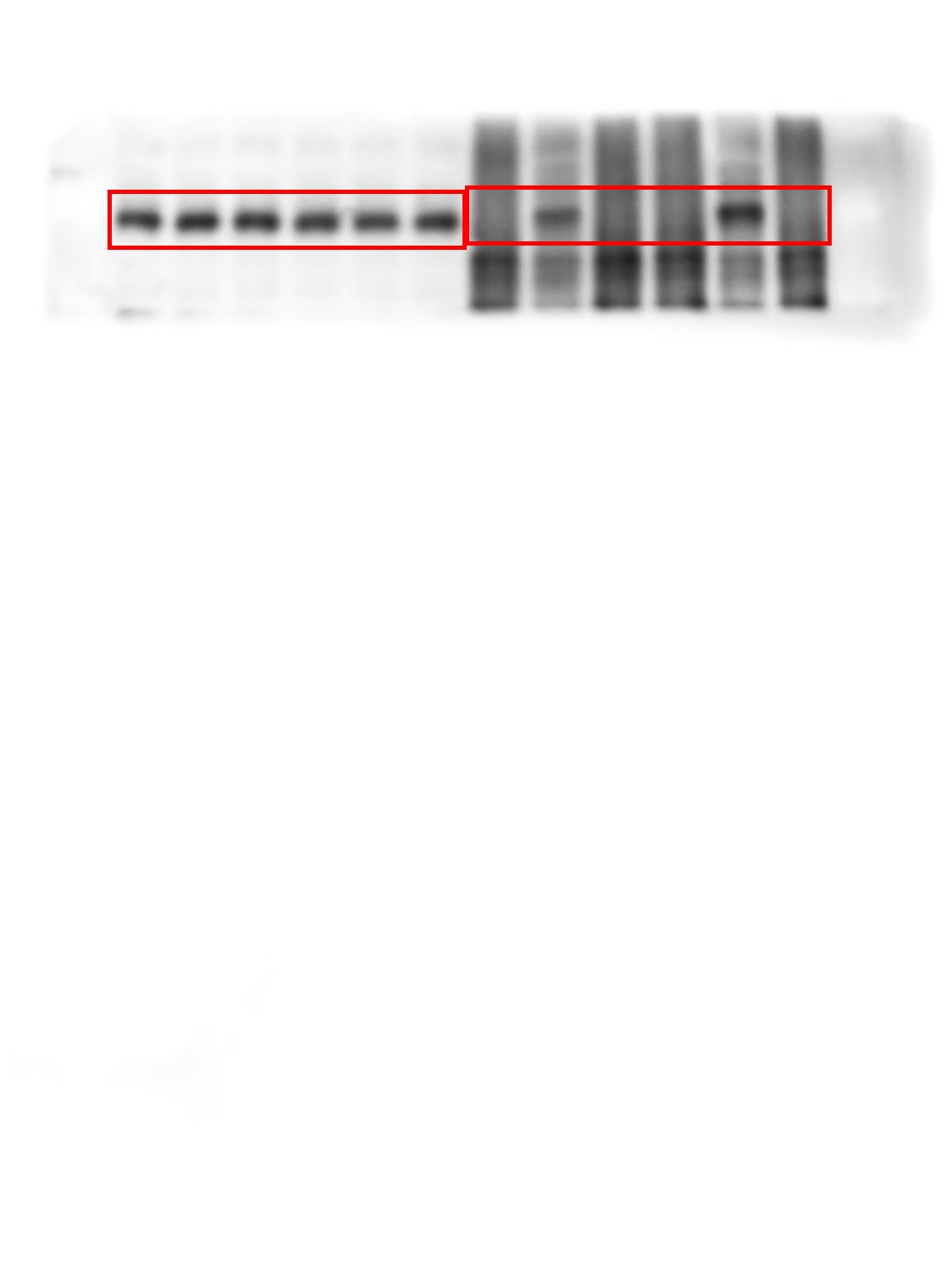

Supplement: Supplementary file 13 — Figure 3 (OLD) [file 41467_2023_42015_MOESM13_ESM.zip › Figure 3/Figure3a/CTIF.jpg]

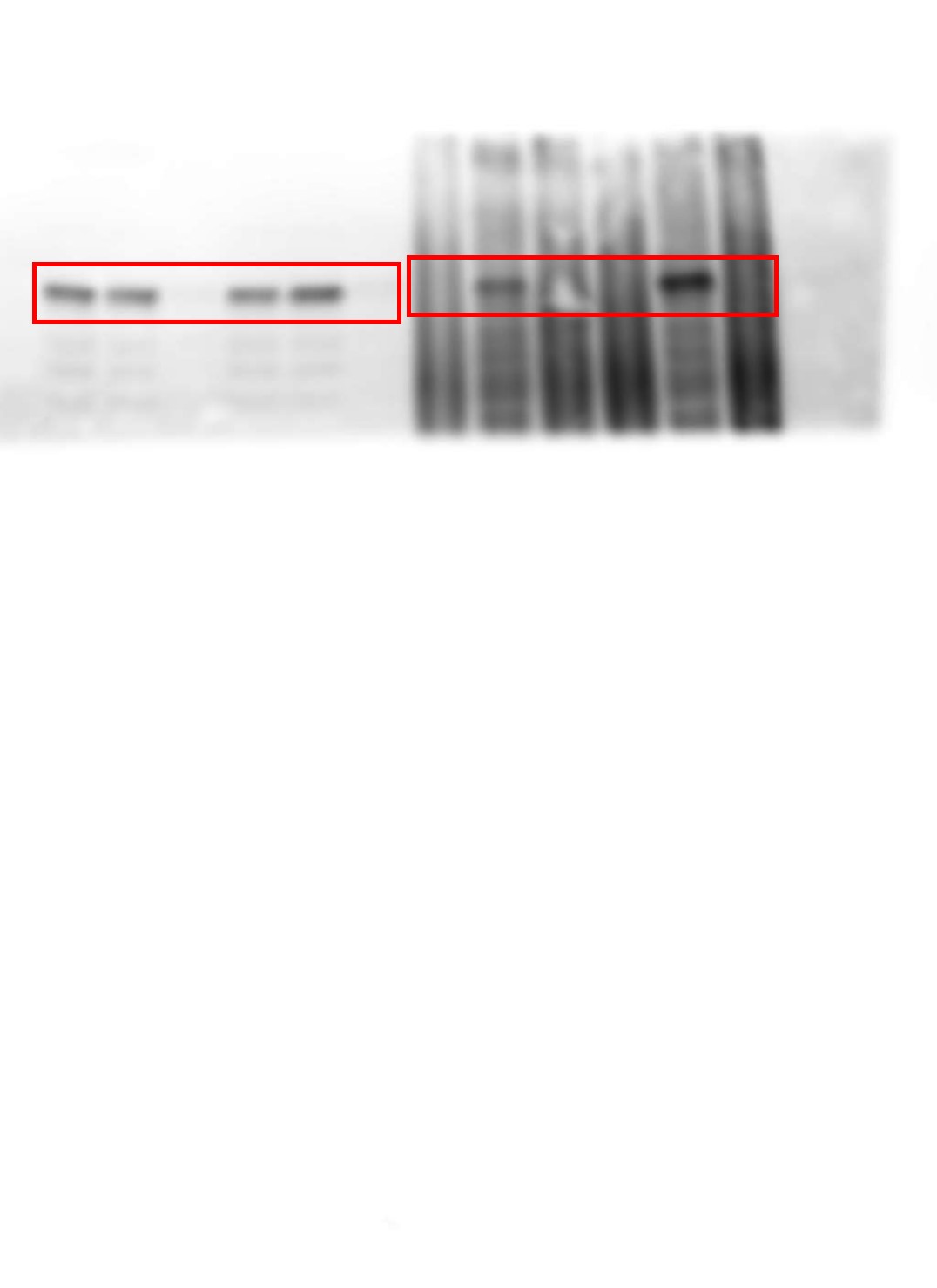

Supplement: Supplementary file 13 — Figure 3 (OLD) [file 41467_2023_42015_MOESM13_ESM.zip › Figure 3/Figure3a/UPF1.jpg]

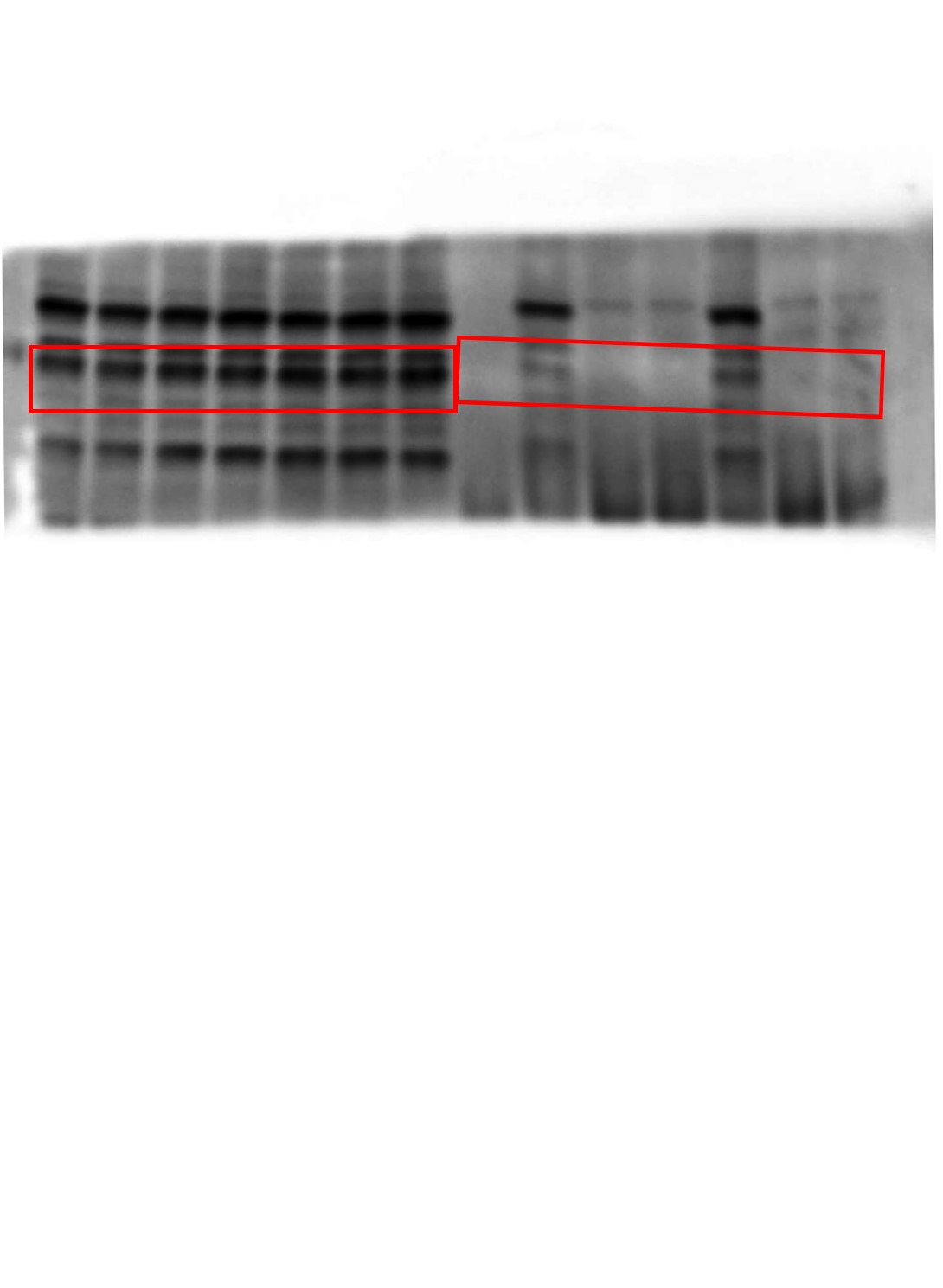

Supplement: Supplementary file 13 — Figure 3 (OLD) [file 41467_2023_42015_MOESM13_ESM.zip › Figure 3/Figure3b/CTIF.jpg]

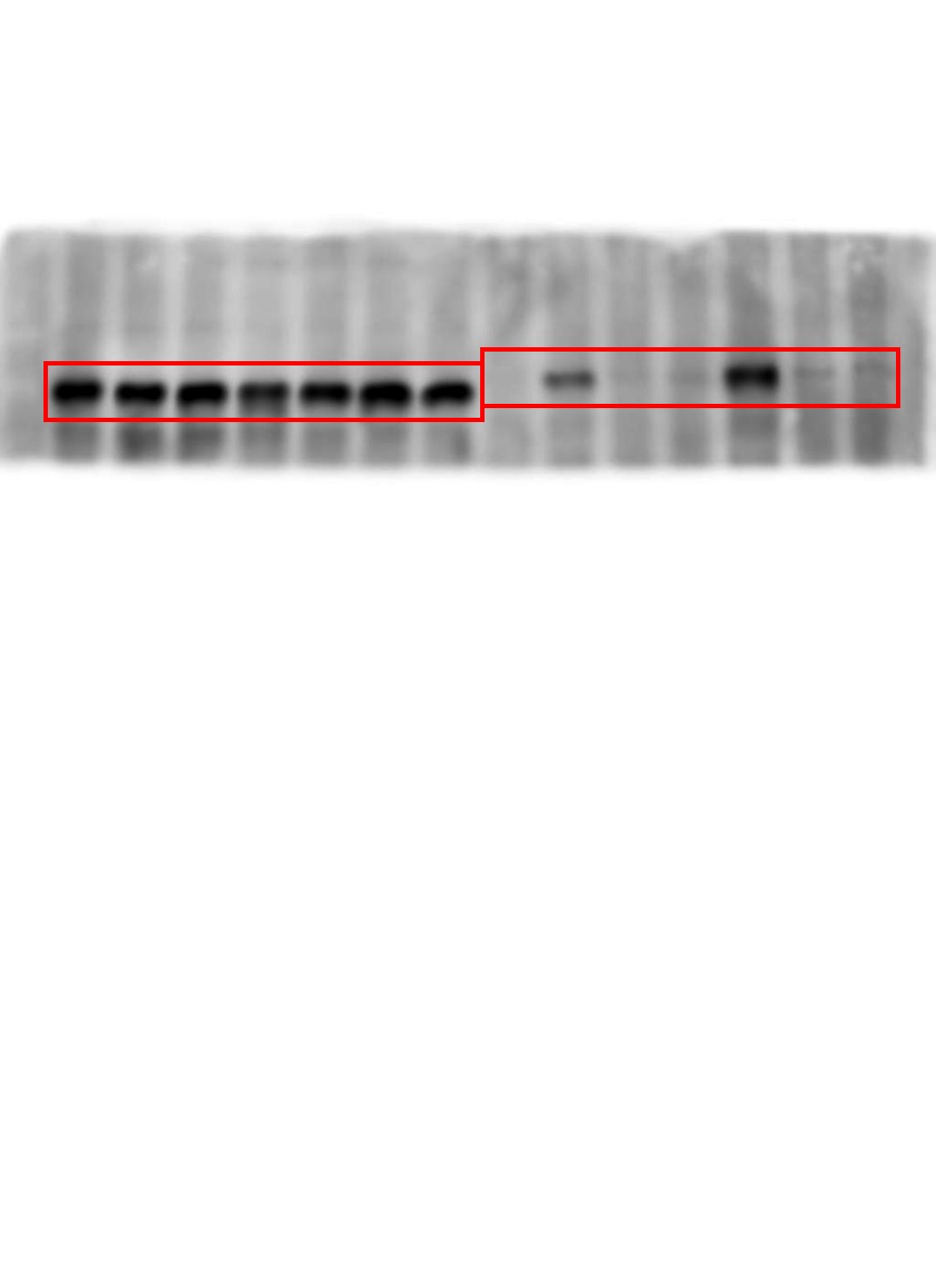

Supplement: Supplementary file 13 — Figure 3 (OLD) [file 41467_2023_42015_MOESM13_ESM.zip › Figure 3/Figure3b/UPF1.jpg]

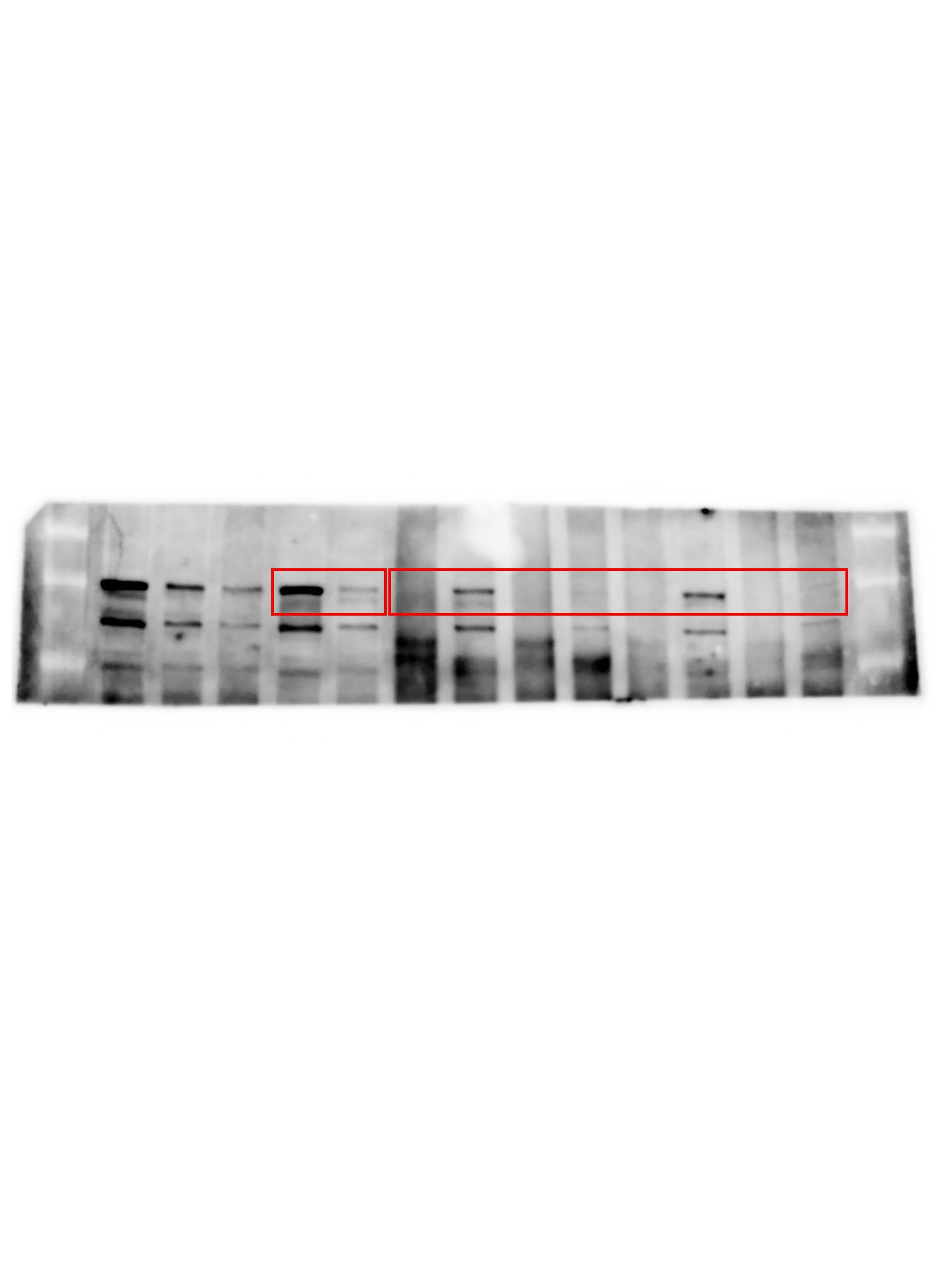

Supplement: Supplementary file 13 — Figure 3 (OLD) [file 41467_2023_42015_MOESM13_ESM.zip › Figure 3/Figure3c/UPF1.jpg]

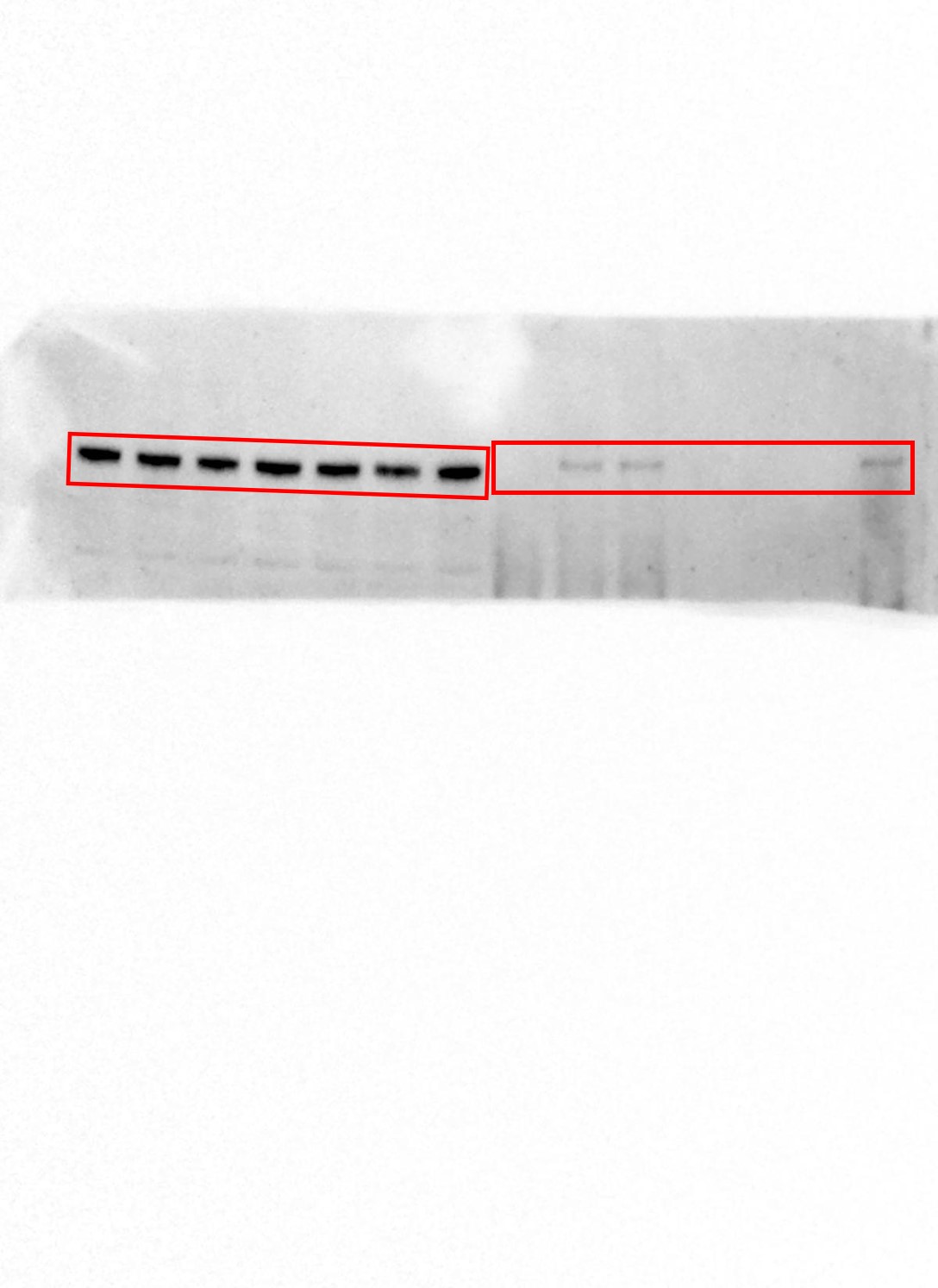

Supplement: Supplementary file 13 — Figure 3 (OLD) [file 41467_2023_42015_MOESM13_ESM.zip › Figure 4/Figure4a/UPF1.jpg]

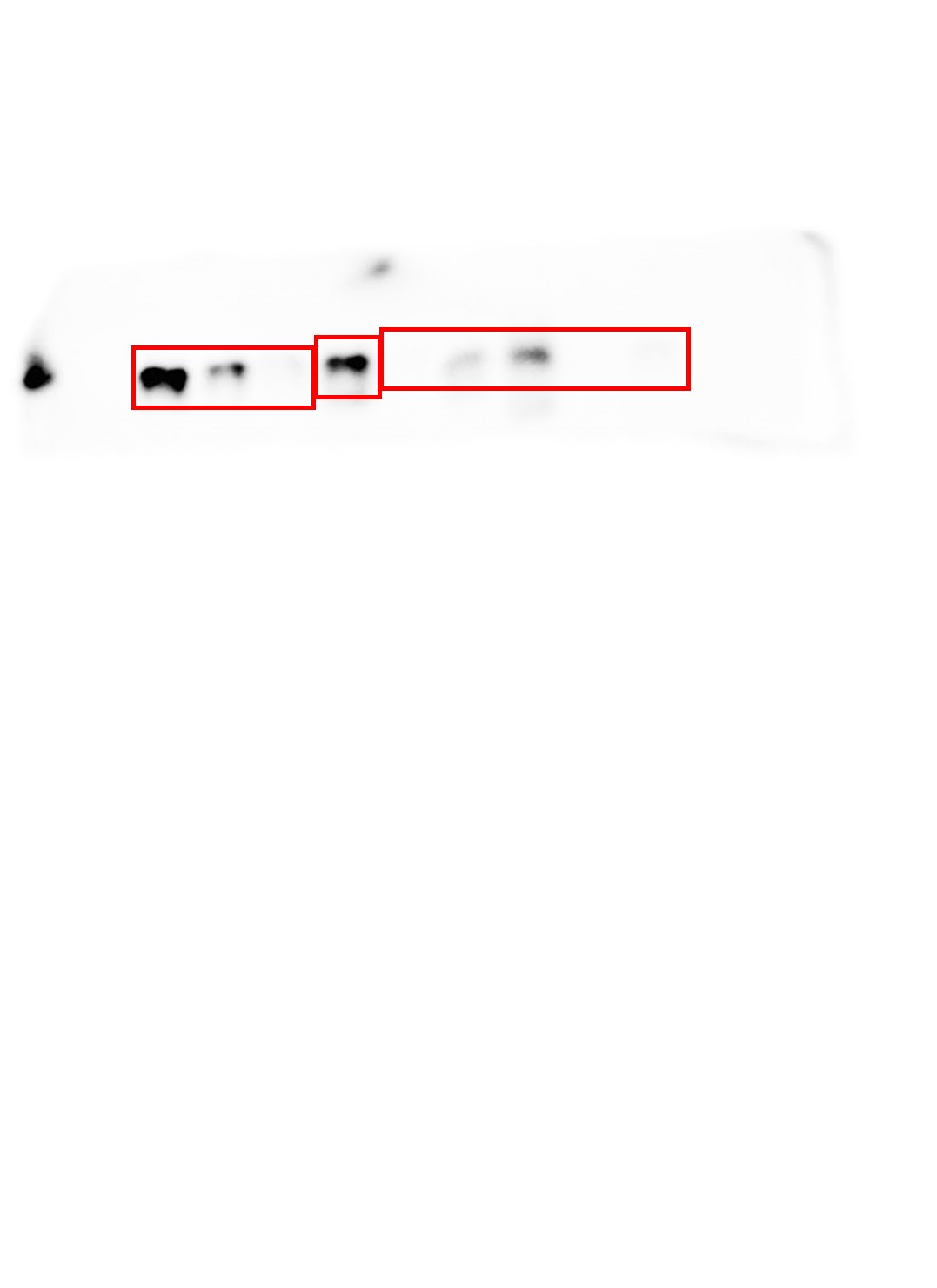

Supplement: Supplementary file 13 — Figure 3 (OLD) [file 41467_2023_42015_MOESM13_ESM.zip › Figure 2/Figure2f/DCTN1.jpg]

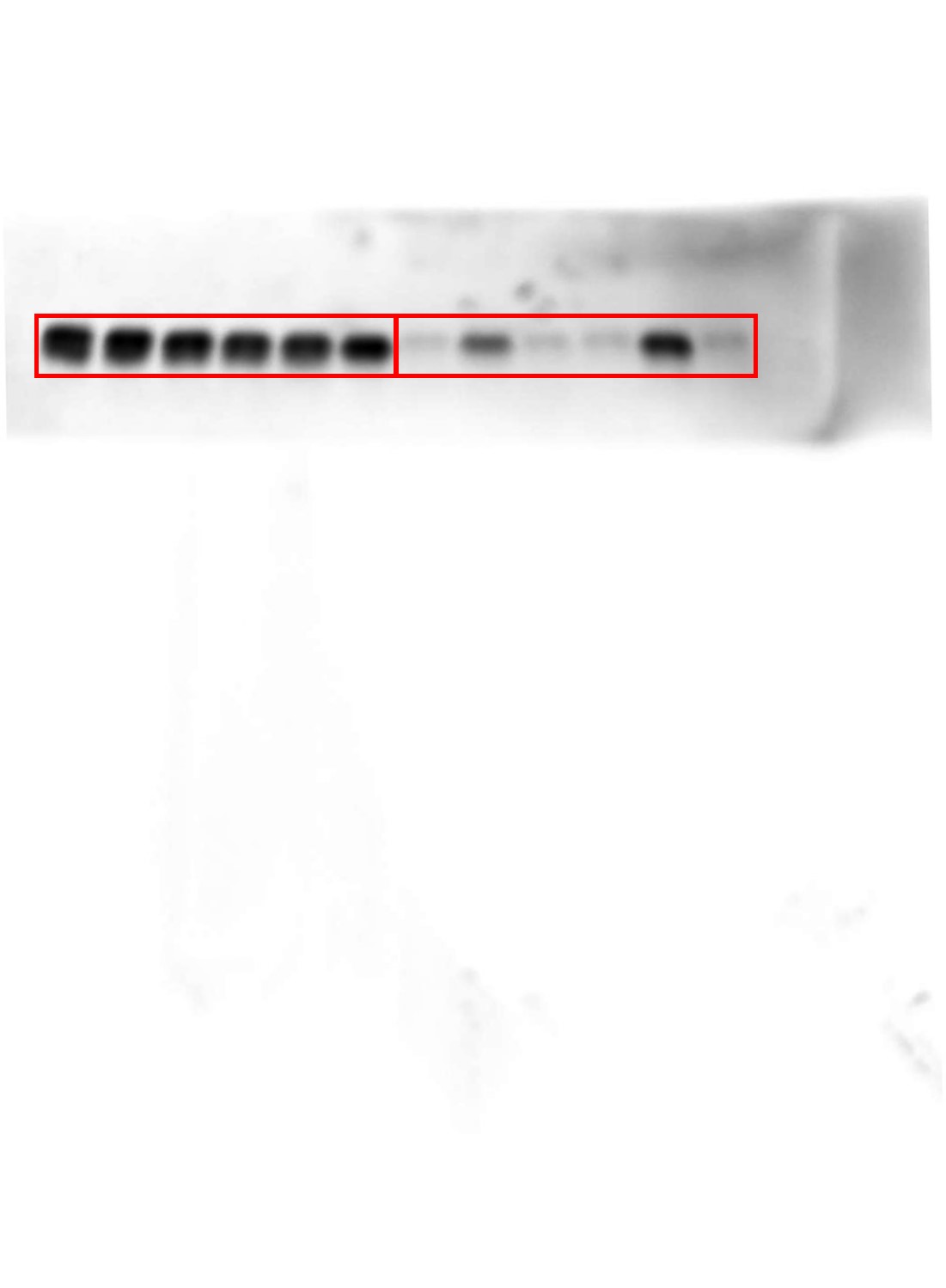

Supplement: Supplementary file 13 — Figure 3 (OLD) [file 41467_2023_42015_MOESM13_ESM.zip › Figure 3/Figure3a/DCTN1.jpg]

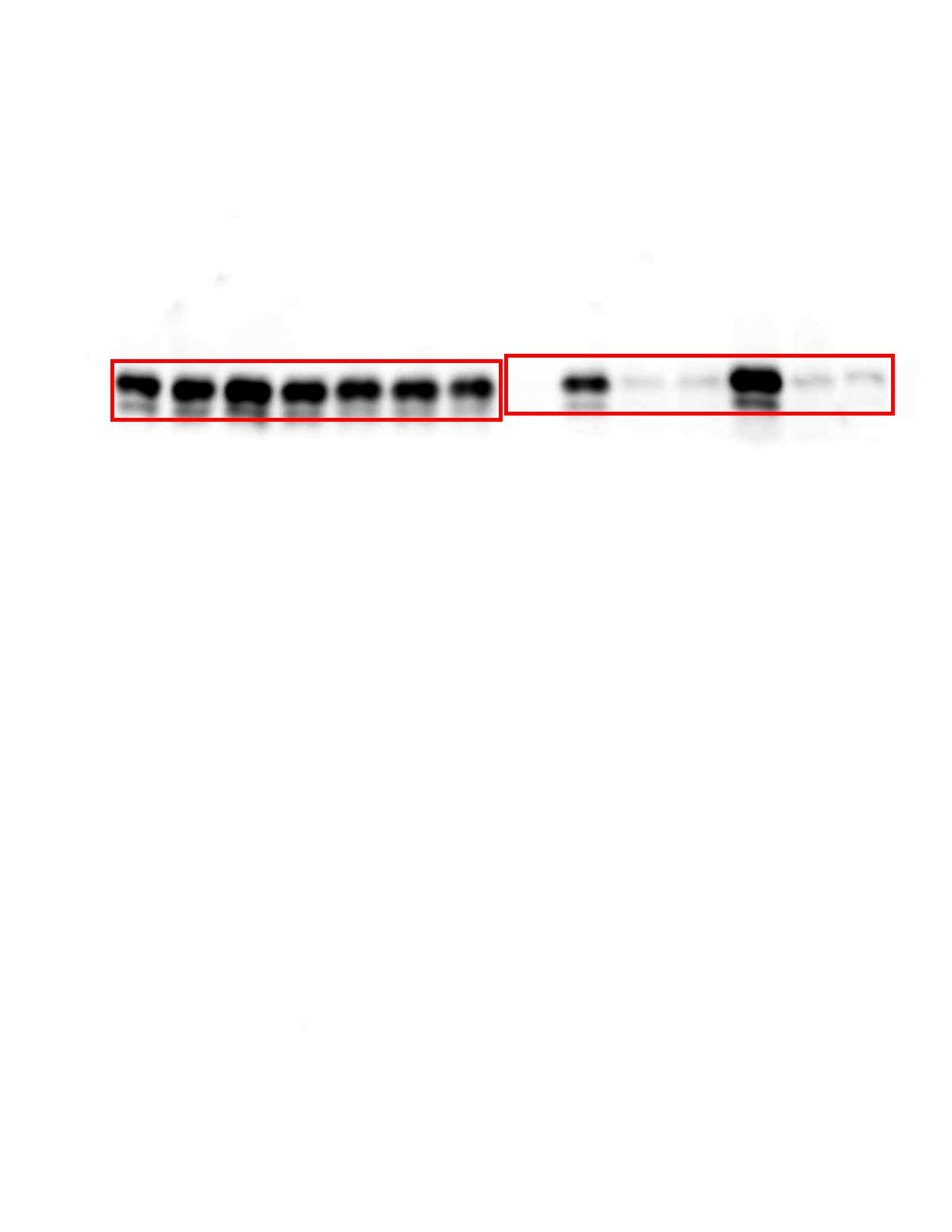

Supplement: Supplementary file 13 — Figure 3 (OLD) [file 41467_2023_42015_MOESM13_ESM.zip › Figure 3/Figure3b/DCTN1.jpg]

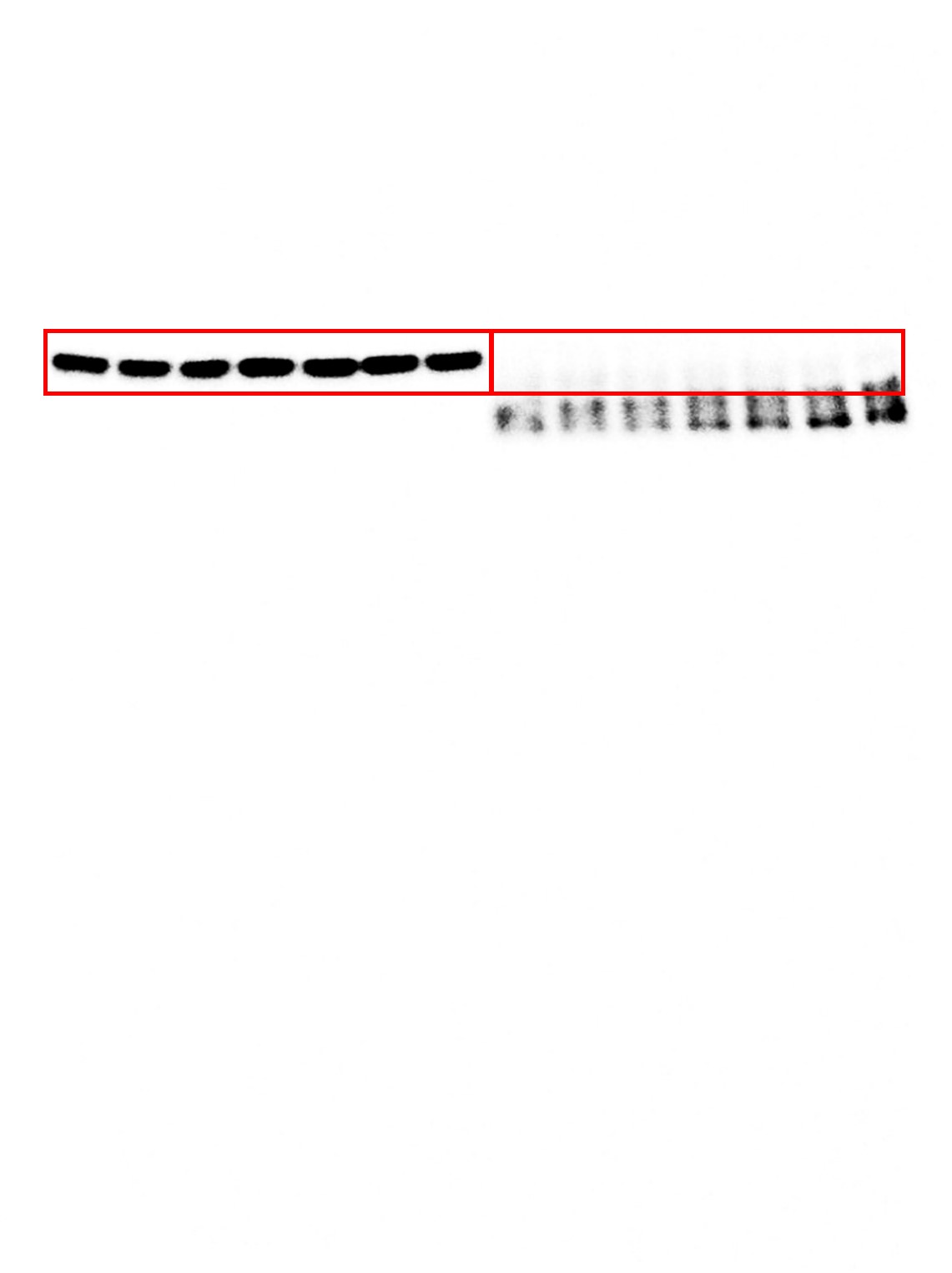

Supplement: Supplementary file 13 — Figure 3 (OLD) [file 41467_2023_42015_MOESM13_ESM.zip › Figure 3/Figure3b/GAPDH.jpg]

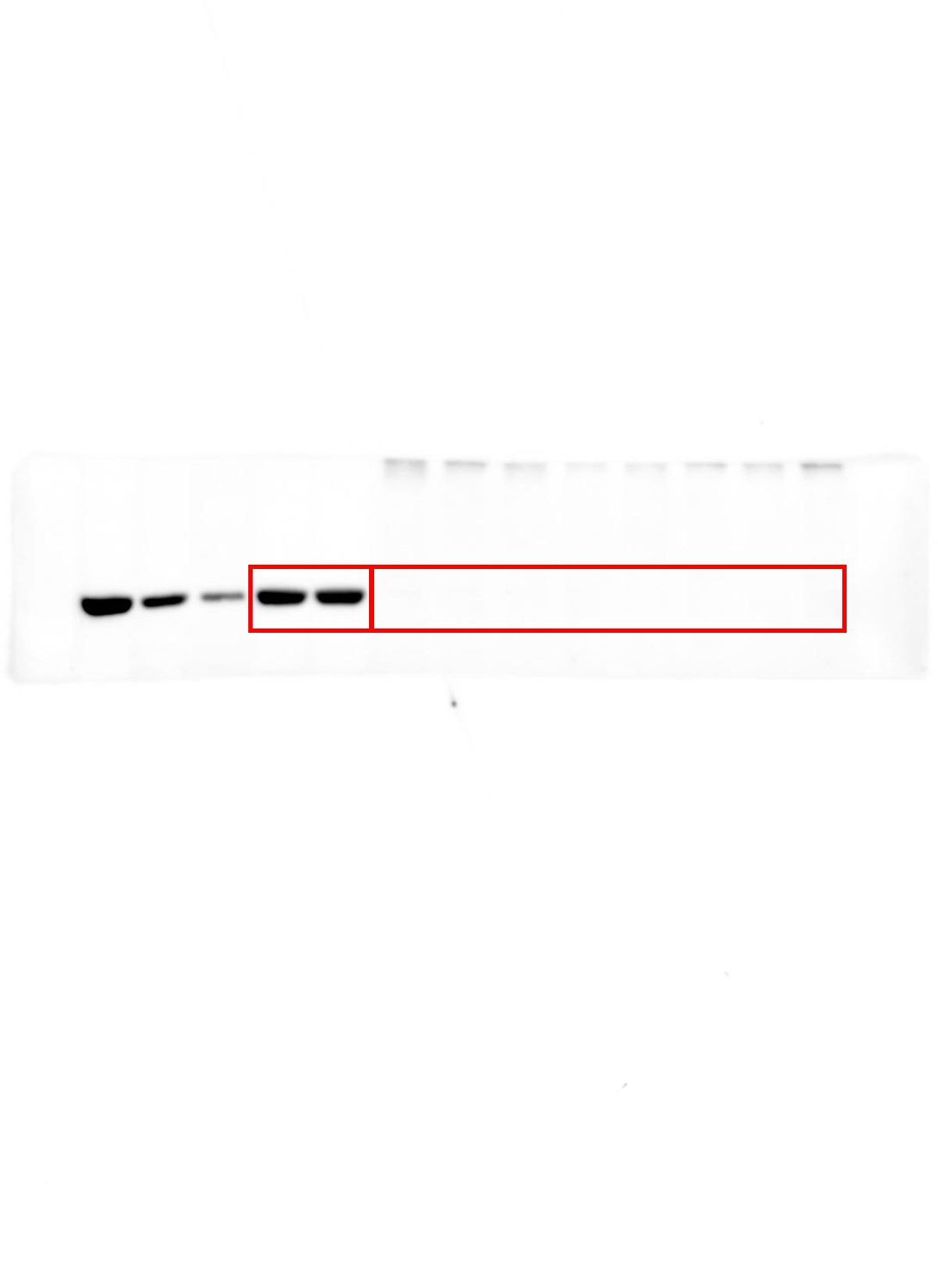

Supplement: Supplementary file 13 — Figure 3 (OLD) [file 41467_2023_42015_MOESM13_ESM.zip › Figure 3/Figure3c/GAPDH.jpg]

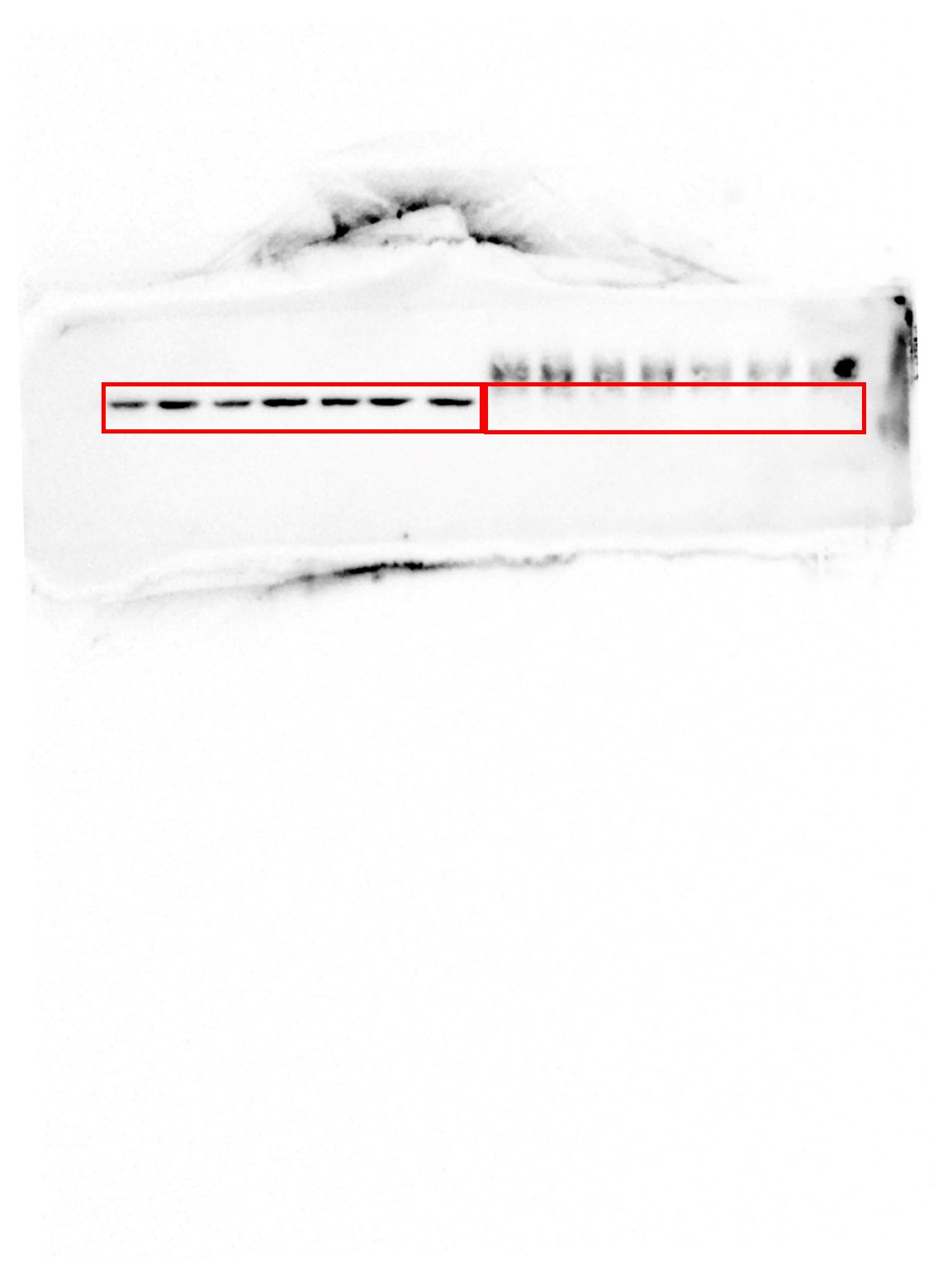

Supplement: Supplementary file 13 — Figure 3 (OLD) [file 41467_2023_42015_MOESM13_ESM.zip › Figure 4/Figure4a/GAPDH.jpg]

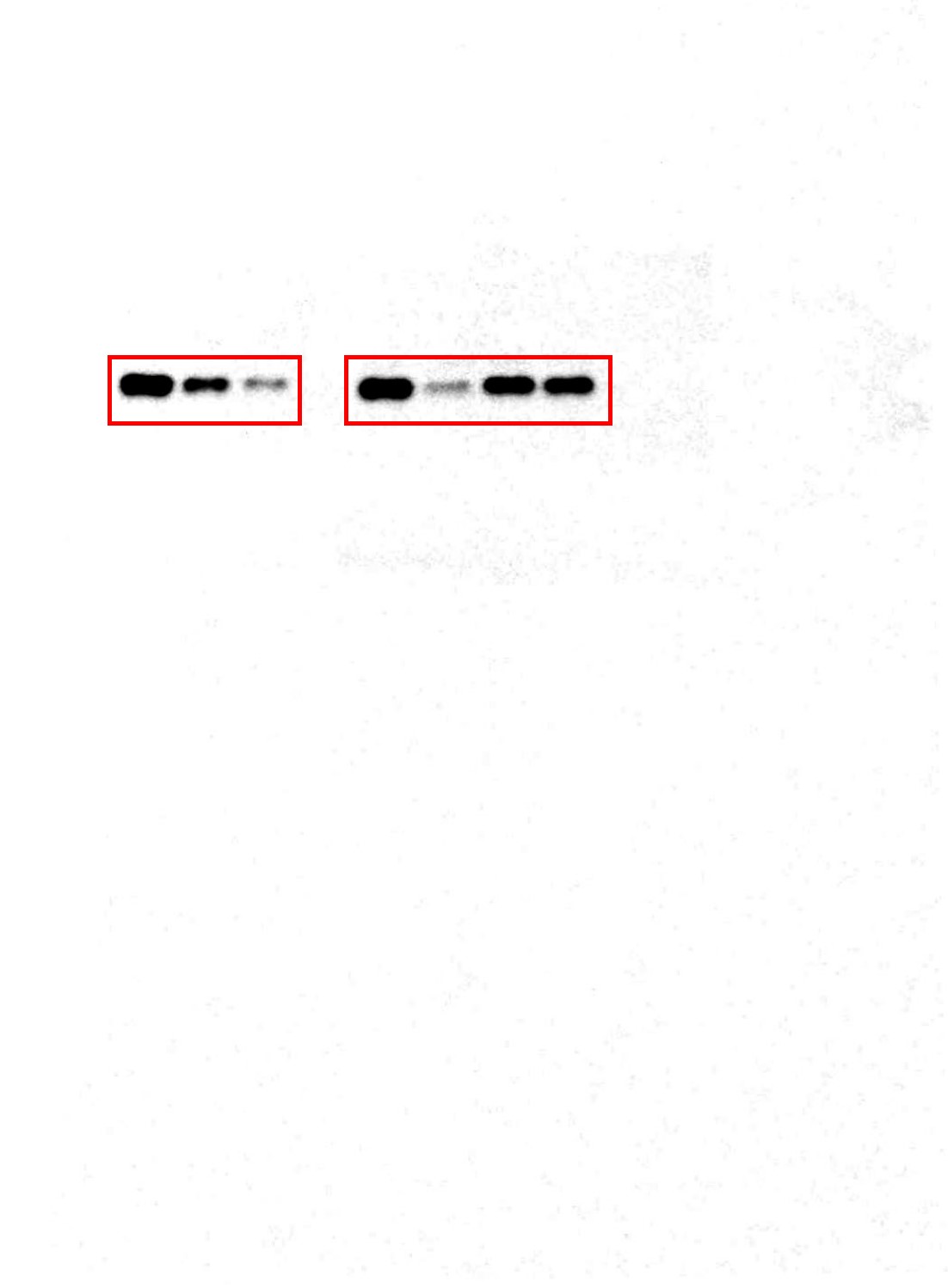

Supplement: Supplementary file 13 — Figure 3 (OLD) [file 41467_2023_42015_MOESM13_ESM.zip › Figure 2/Figure2e/YTHDF1.jpg]

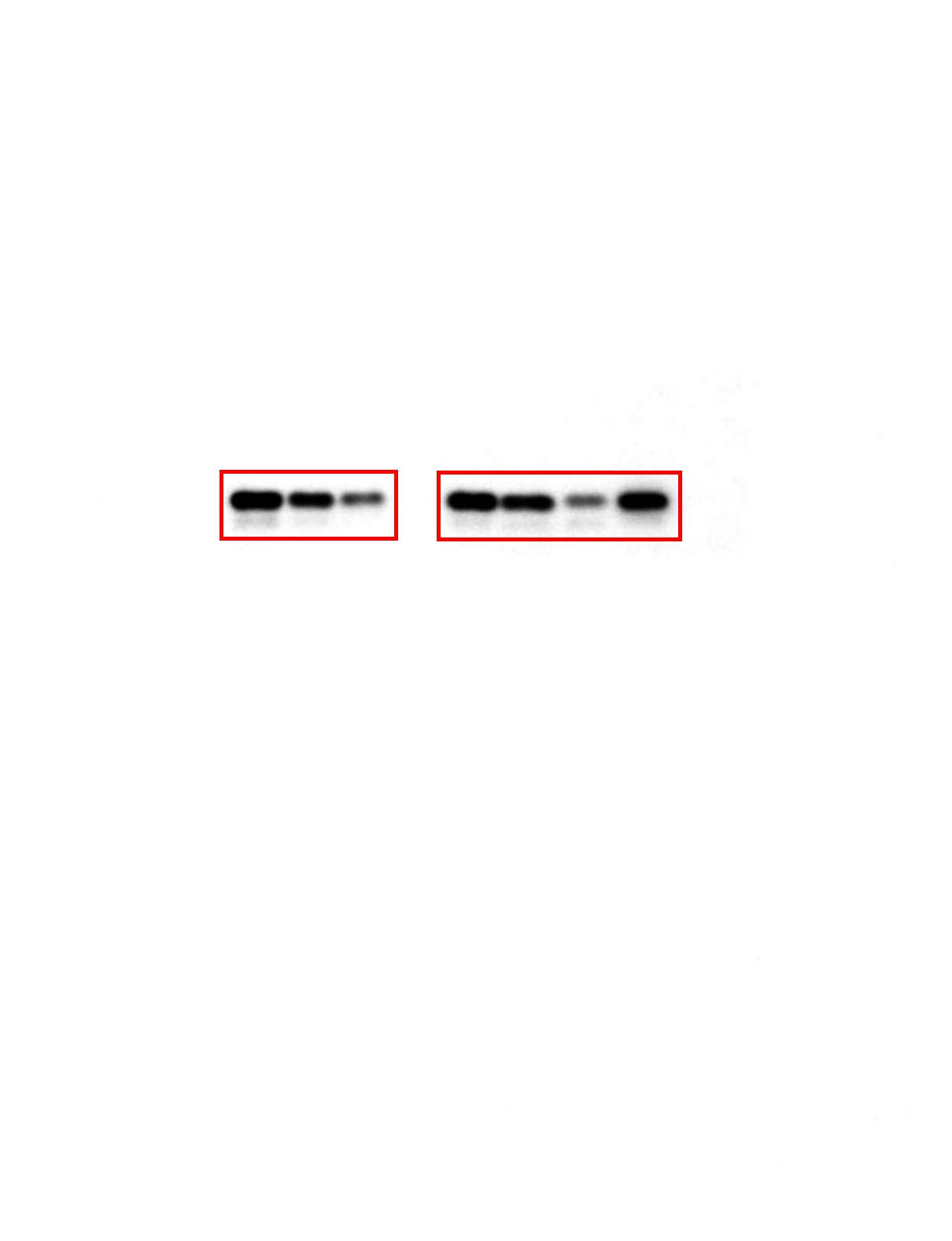

Supplement: Supplementary file 13 — Figure 3 (OLD) [file 41467_2023_42015_MOESM13_ESM.zip › Figure 2/Figure2e/YTHDF2.jpg]

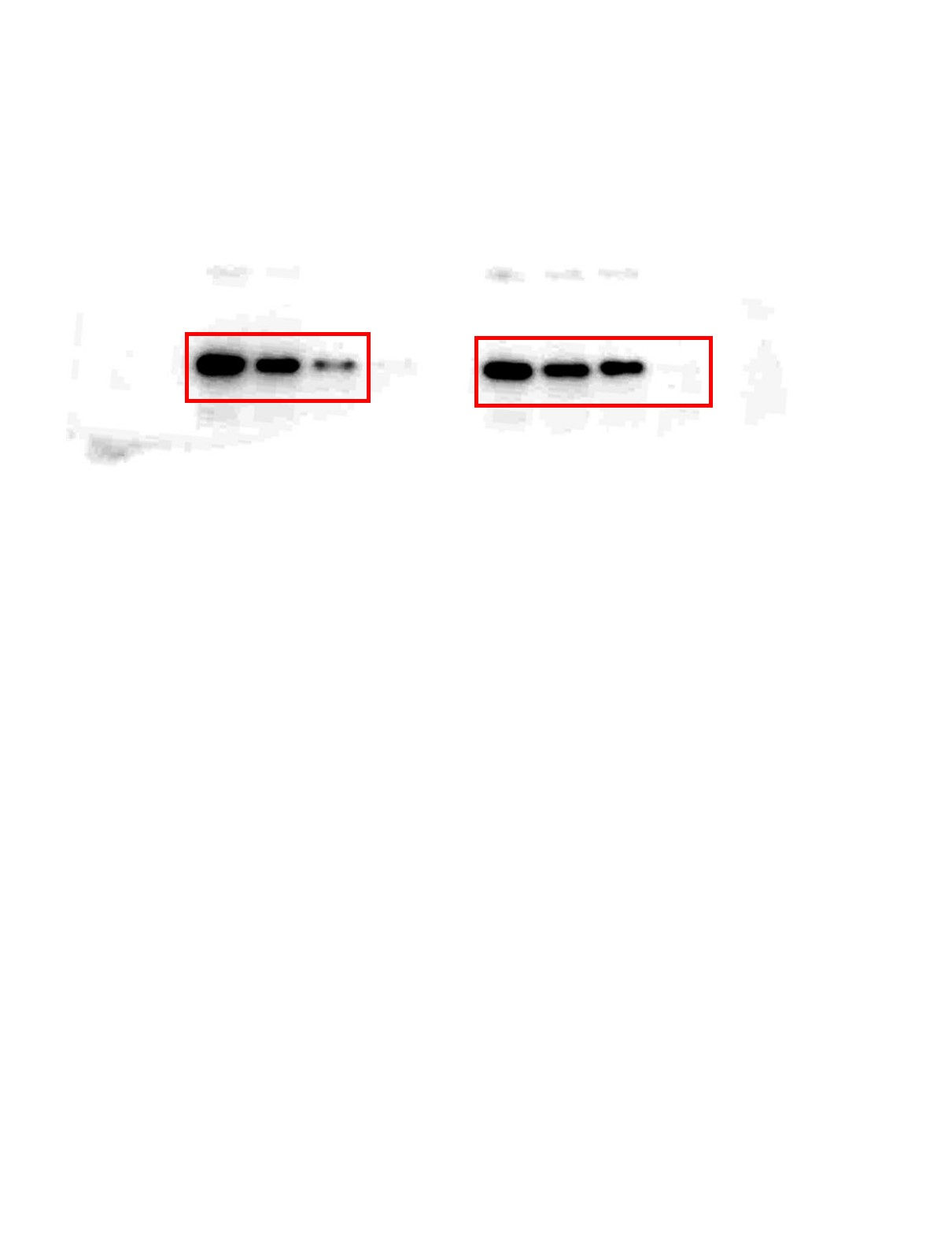

Supplement: Supplementary file 13 — Figure 3 (OLD) [file 41467_2023_42015_MOESM13_ESM.zip › Figure 2/Figure2e/YTHDF3.jpg]

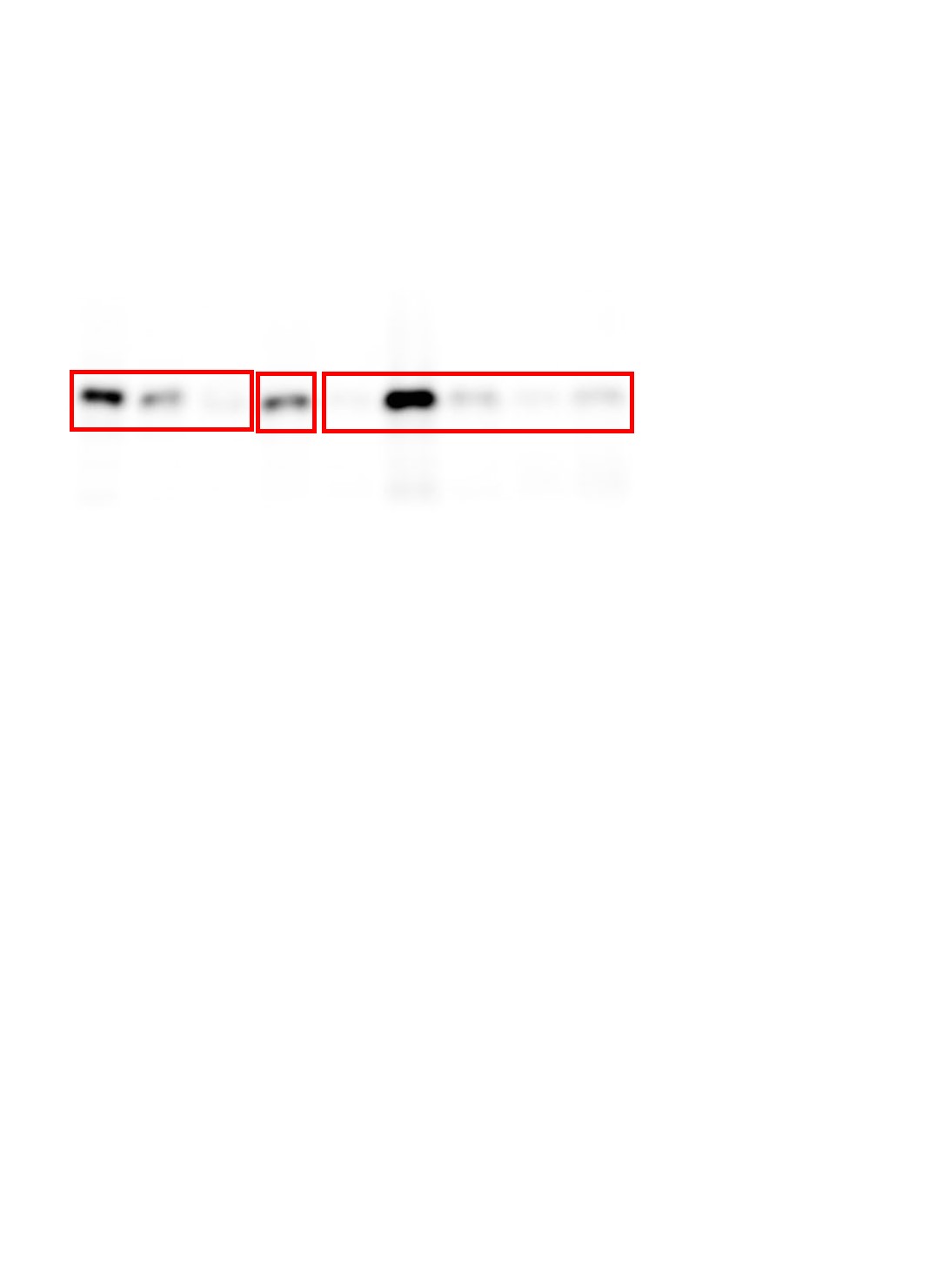

Supplement: Supplementary file 13 — Figure 3 (OLD) [file 41467_2023_42015_MOESM13_ESM.zip › Figure 2/Figure2f/YTHDF1.jpg]

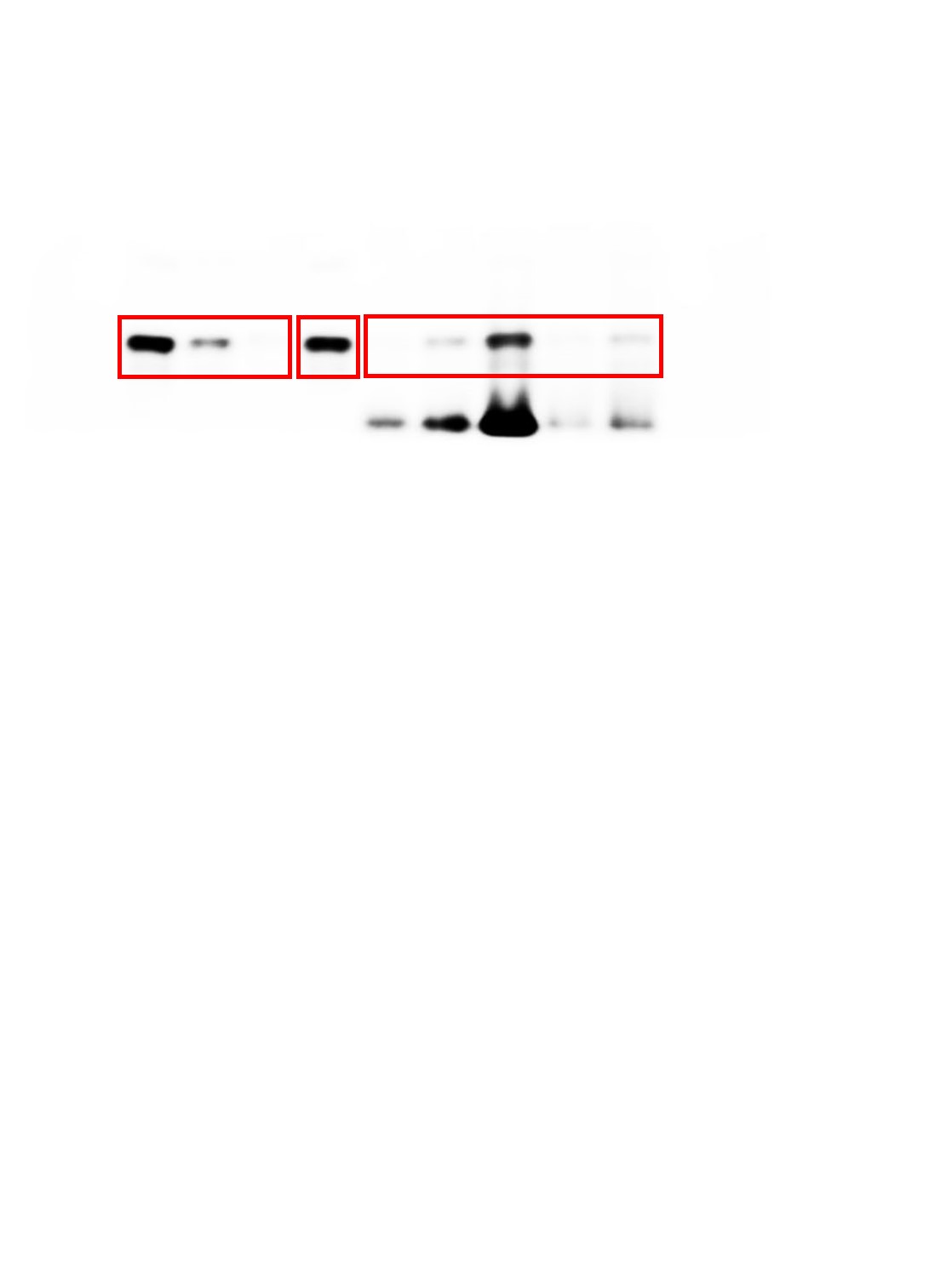

Supplement: Supplementary file 13 — Figure 3 (OLD) [file 41467_2023_42015_MOESM13_ESM.zip › Figure 2/Figure2f/YTHDF2.jpg]

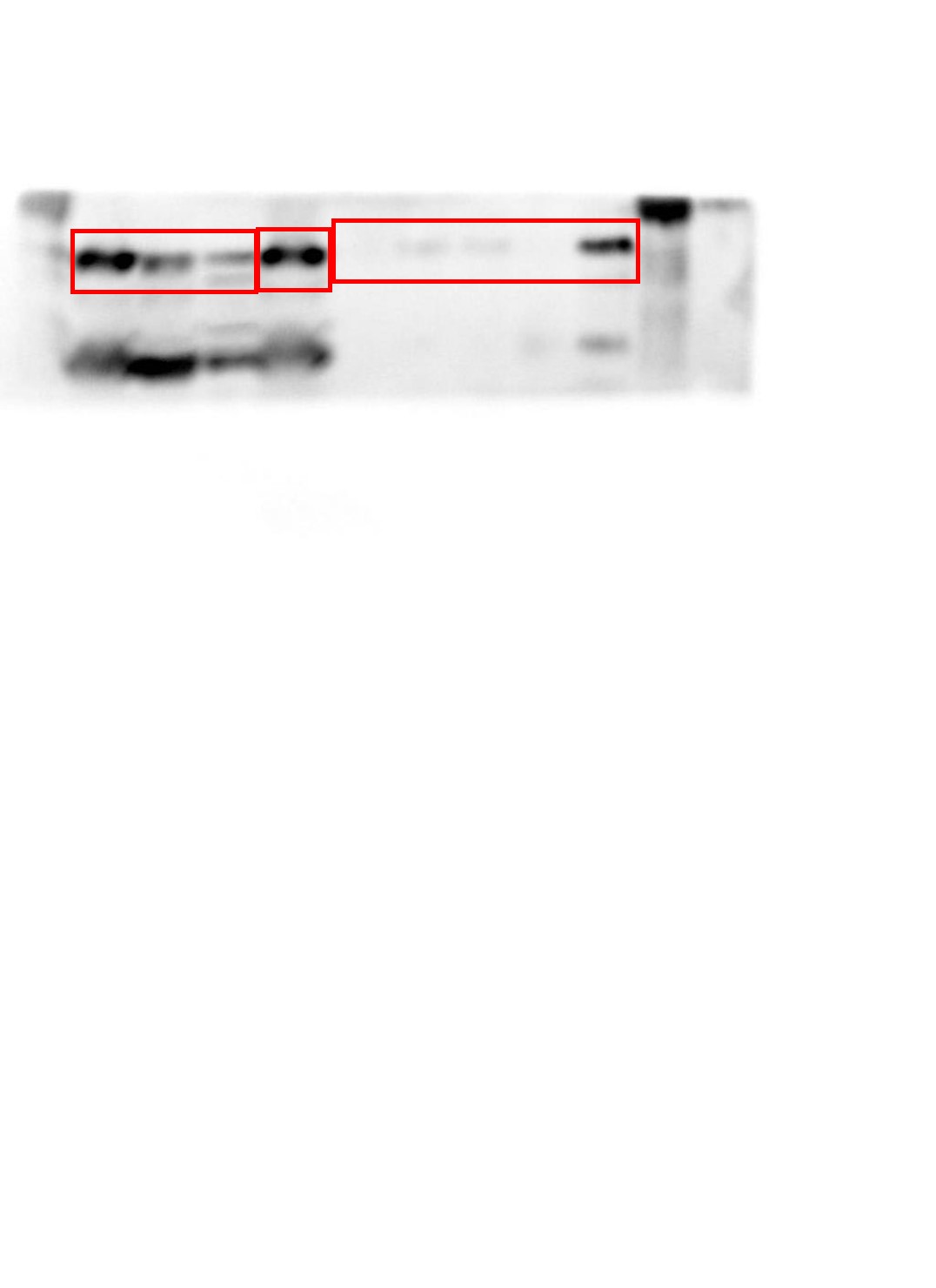

Supplement: Supplementary file 13 — Figure 3 (OLD) [file 41467_2023_42015_MOESM13_ESM.zip › Figure 2/Figure2f/YTHDF3.jpg]

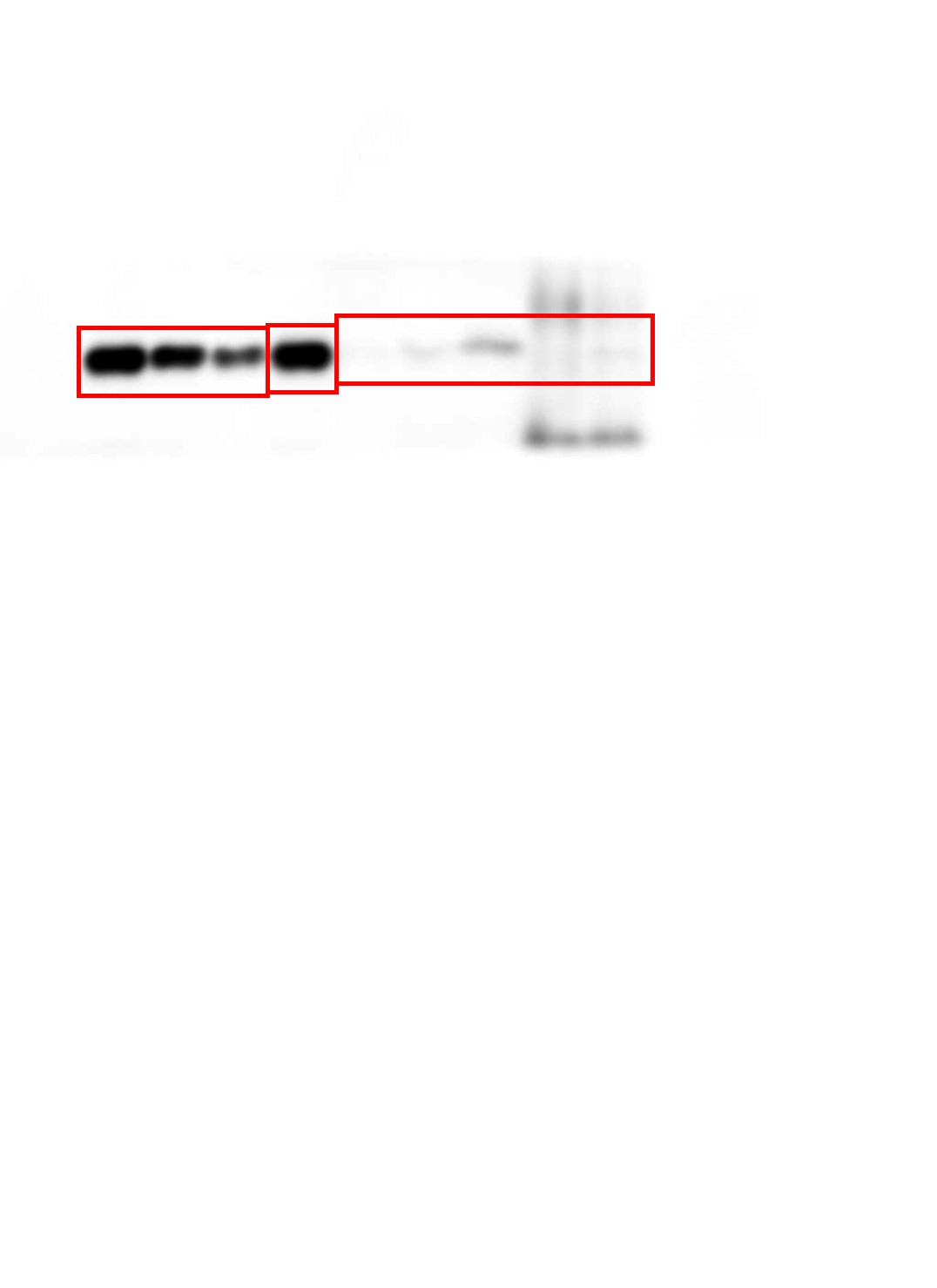

Supplement: Supplementary file 13 — Figure 3 (OLD) [file 41467_2023_42015_MOESM13_ESM.zip › Figure 2/Figure2f/eEF1A1.jpg]

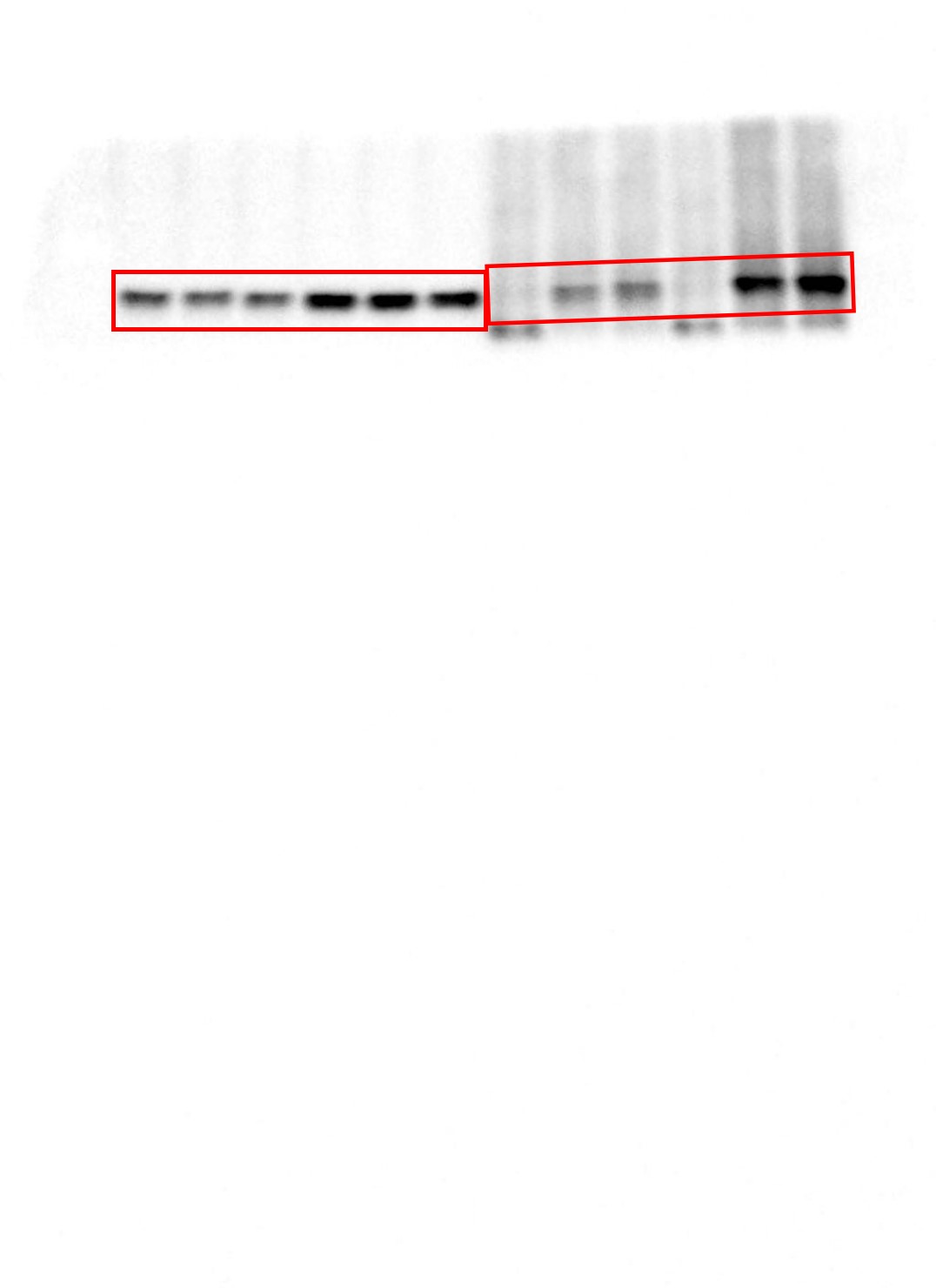

Supplement: Supplementary file 13 — Figure 3 (OLD) [file 41467_2023_42015_MOESM13_ESM.zip › Figure 3/Figure3a/YTHDF2.jpg]

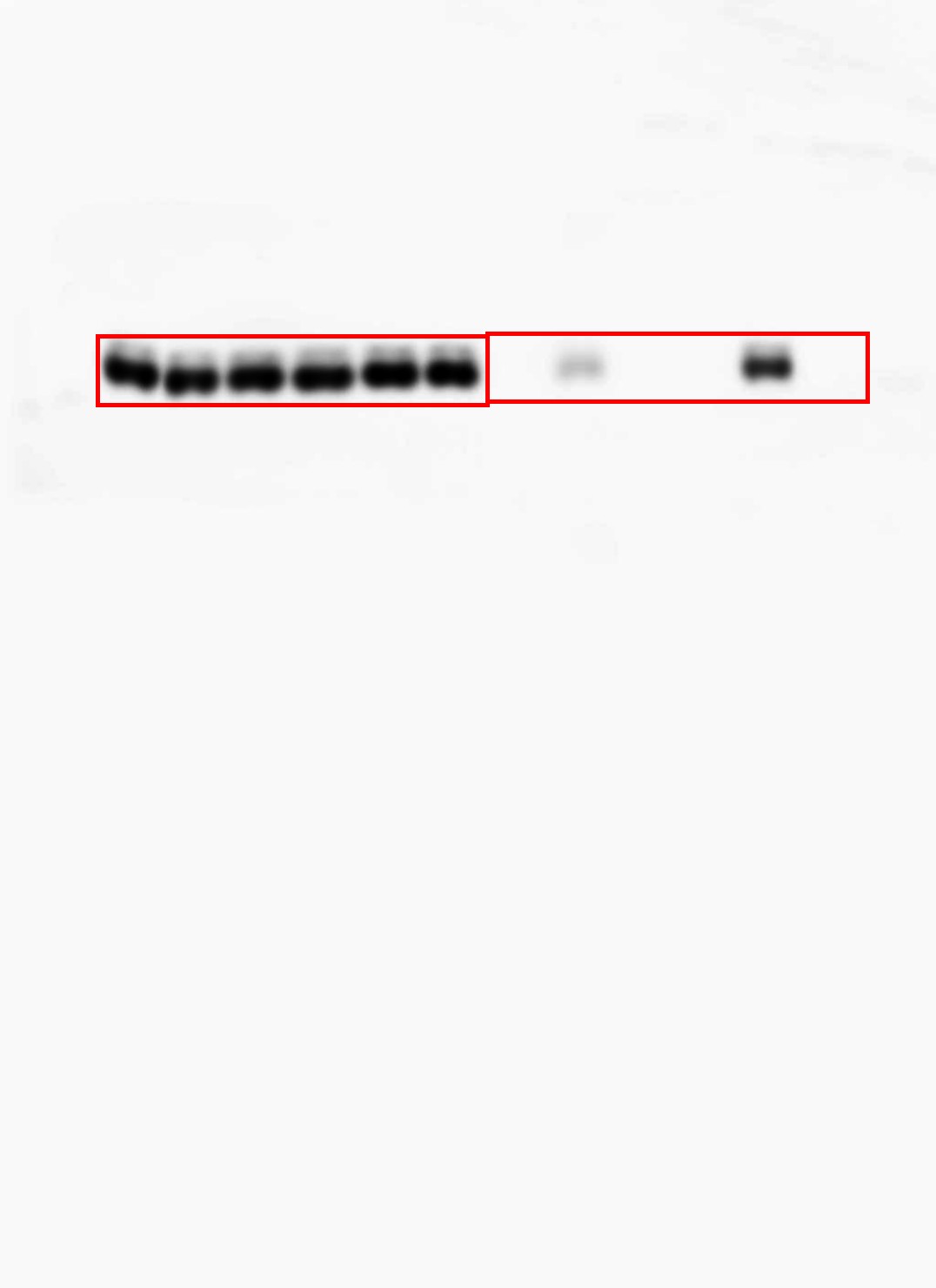

Supplement: Supplementary file 13 — Figure 3 (OLD) [file 41467_2023_42015_MOESM13_ESM.zip › Figure 3/Figure3a/eEF1A1.jpg]

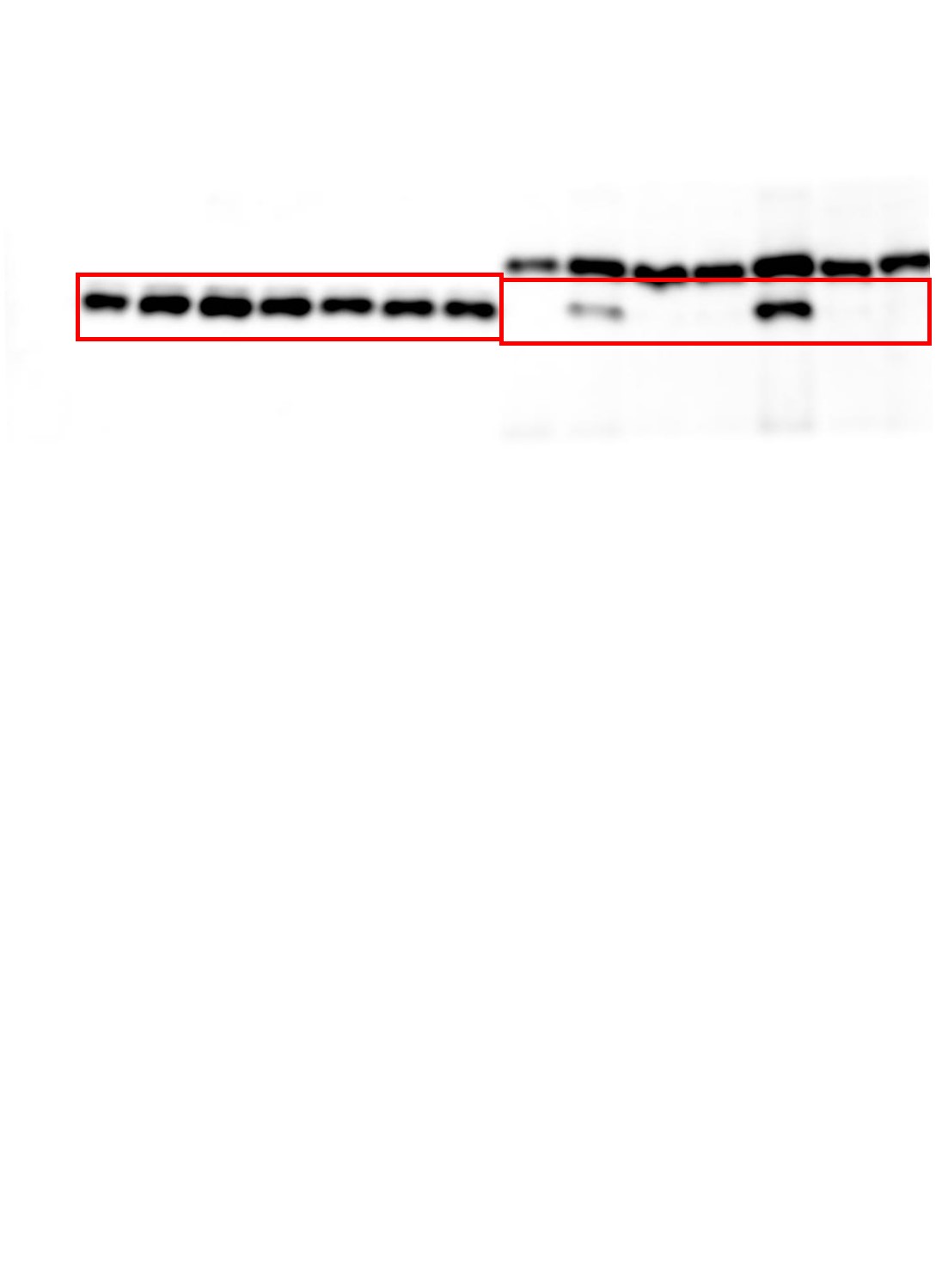

Supplement: Supplementary file 13 — Figure 3 (OLD) [file 41467_2023_42015_MOESM13_ESM.zip › Figure 3/Figure3b/eEF1A1.jpg]

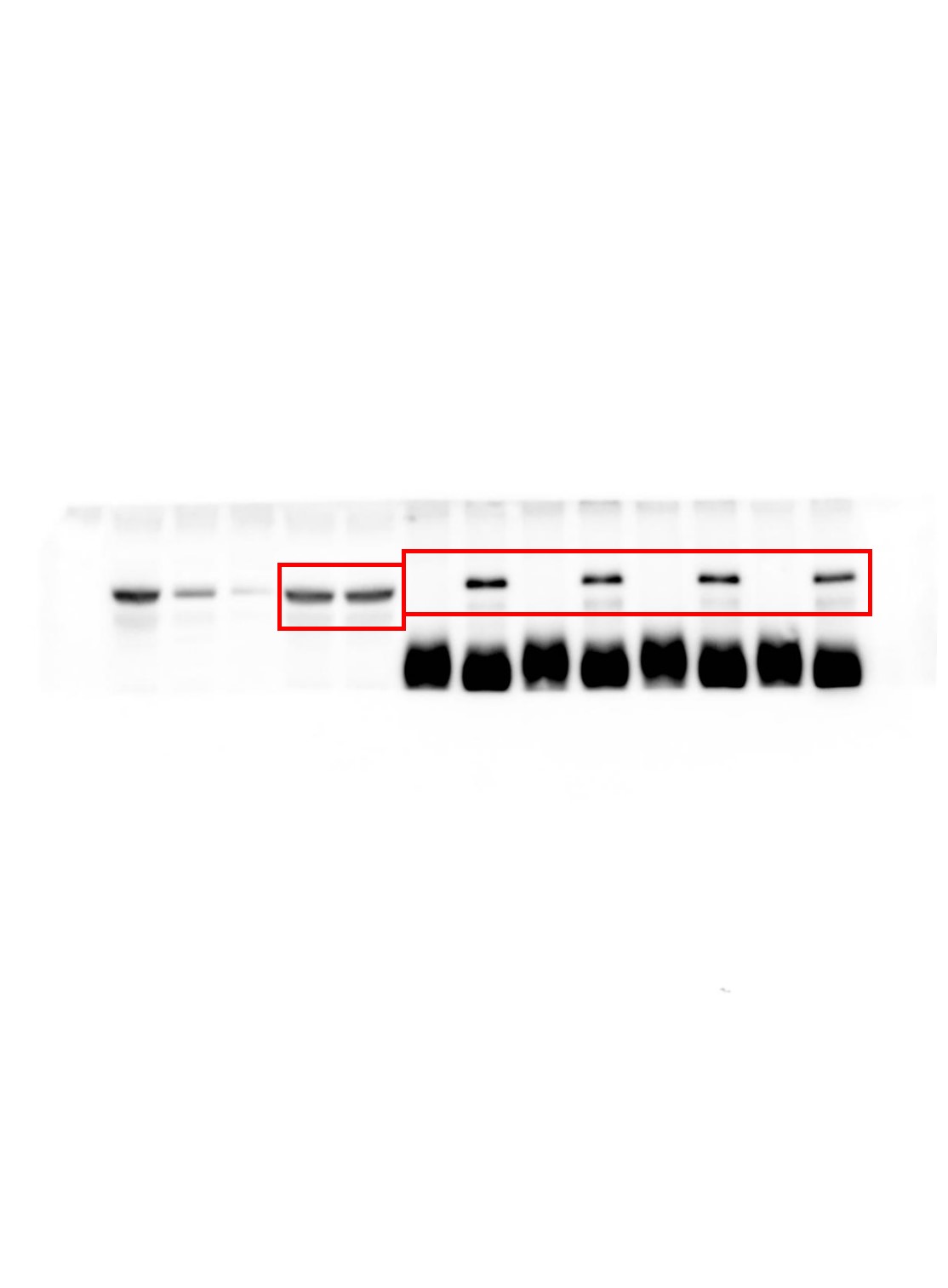

Supplement: Supplementary file 13 — Figure 3 (OLD) [file 41467_2023_42015_MOESM13_ESM.zip › Figure 3/Figure3c/YTHDF2.jpg]

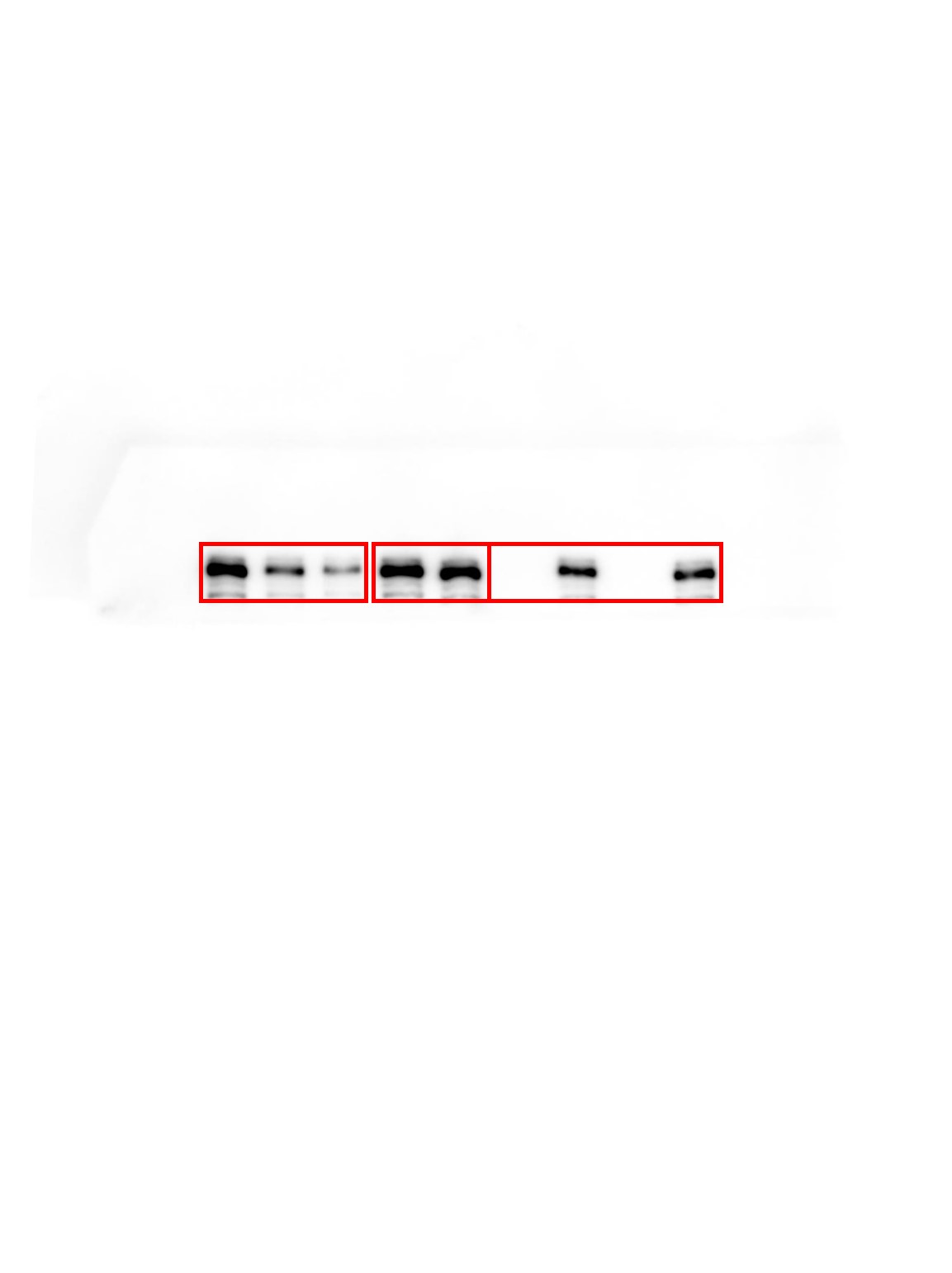

Supplement: Supplementary file 13 — Figure 3 (OLD) [file 41467_2023_42015_MOESM13_ESM.zip › Figure 8/Figure 8a/DCTN1.jpg]

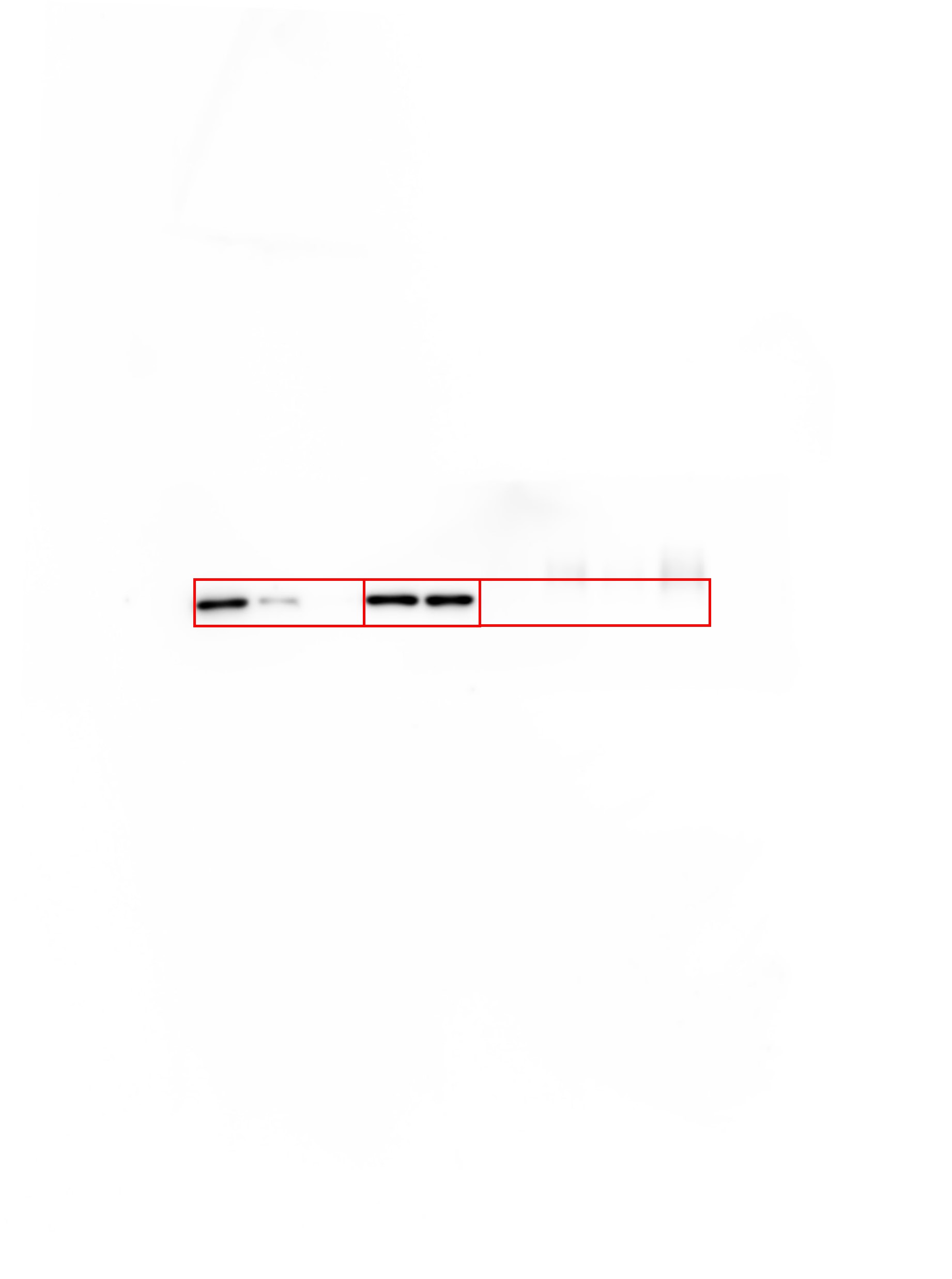

Supplement: Supplementary file 13 — Figure 3 (OLD) [file 41467_2023_42015_MOESM13_ESM.zip › Figure 8/Figure 8a/GAPDH.jpg]

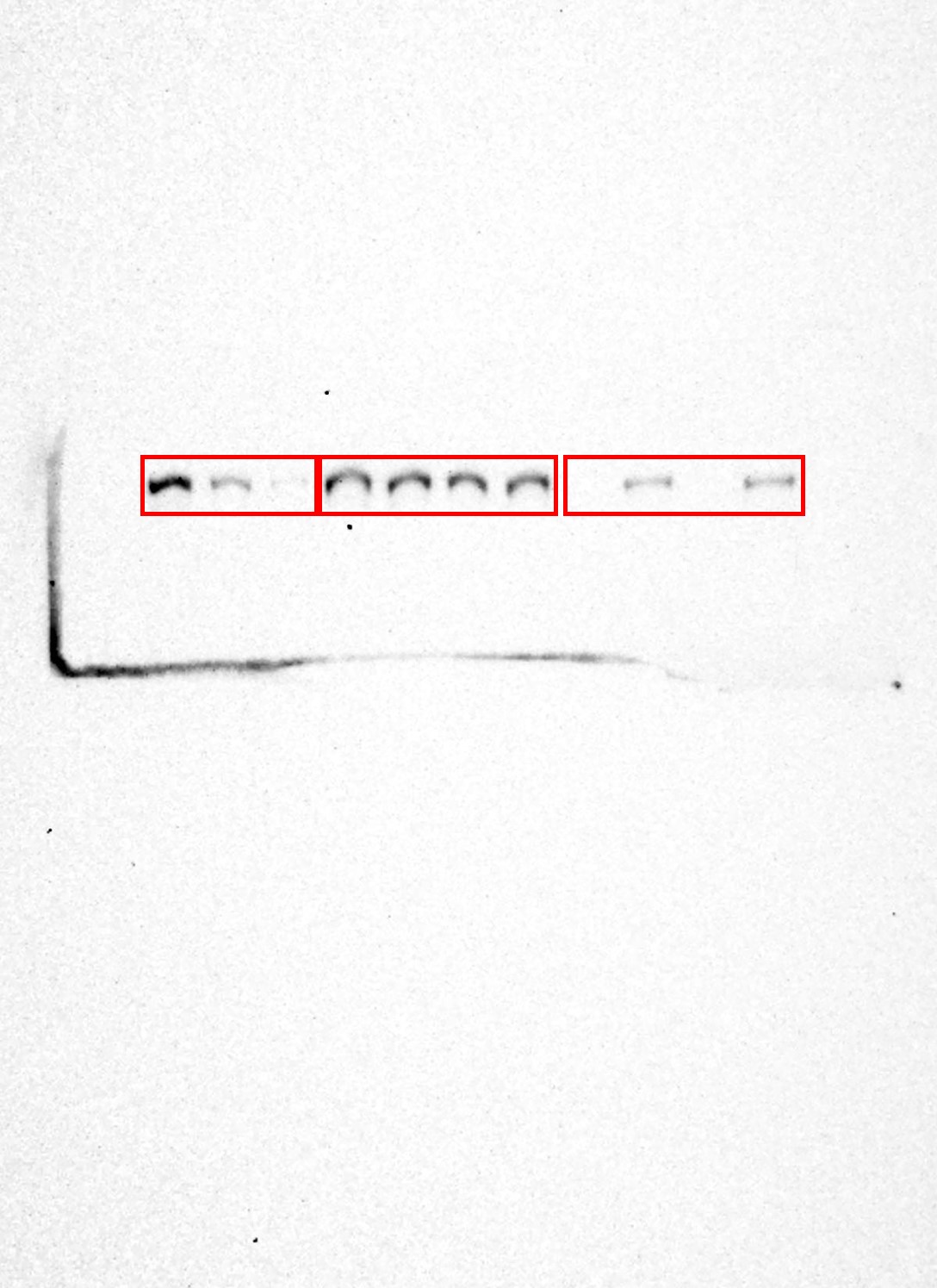

Supplement: Supplementary file 13 — Figure 3 (OLD) [file 41467_2023_42015_MOESM13_ESM.zip › Figure 8/Figure 8b/DCTN1.jpg]

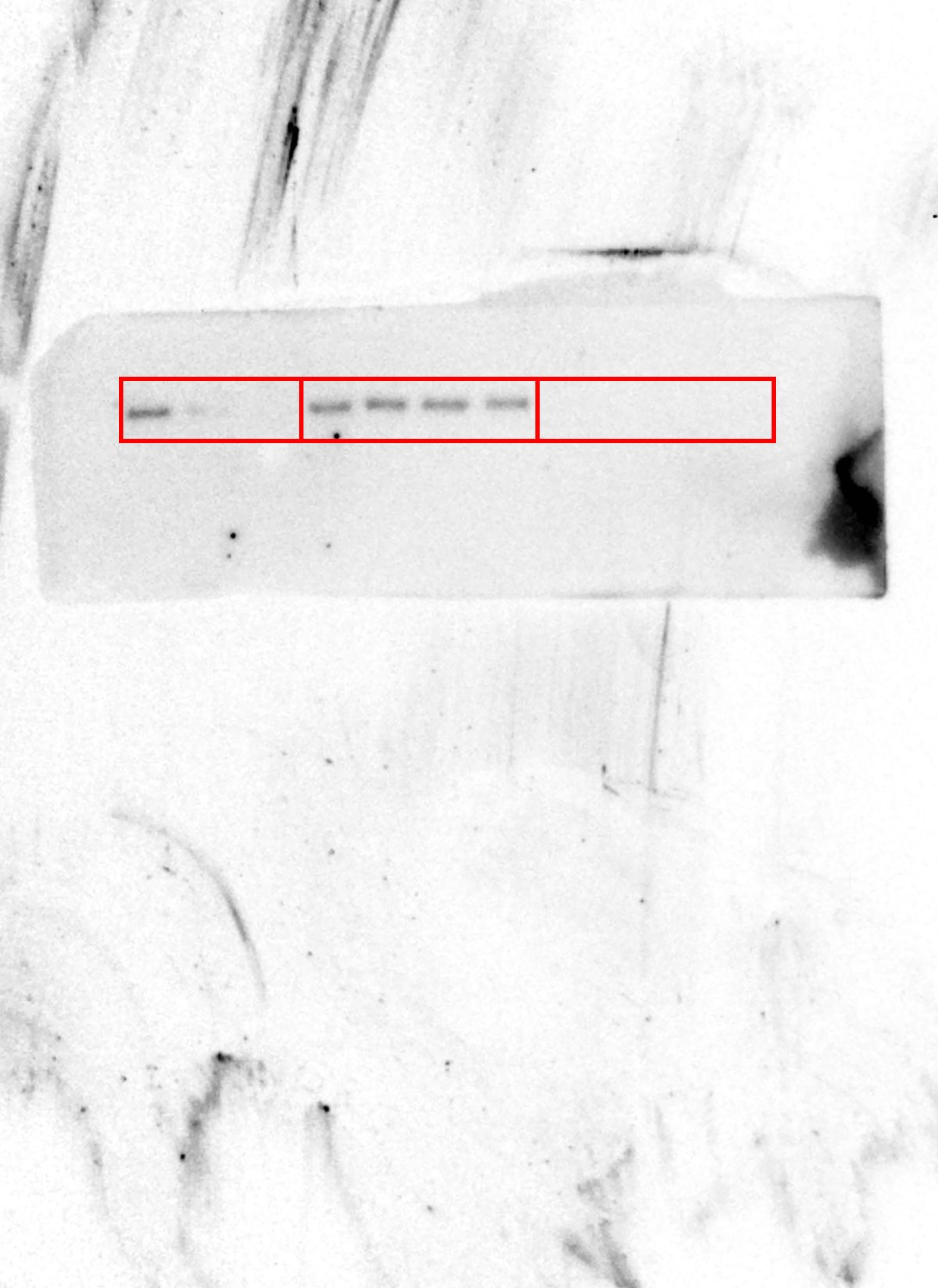

Supplement: Supplementary file 13 — Figure 3 (OLD) [file 41467_2023_42015_MOESM13_ESM.zip › Figure 8/Figure 8b/GAPDH.jpg]

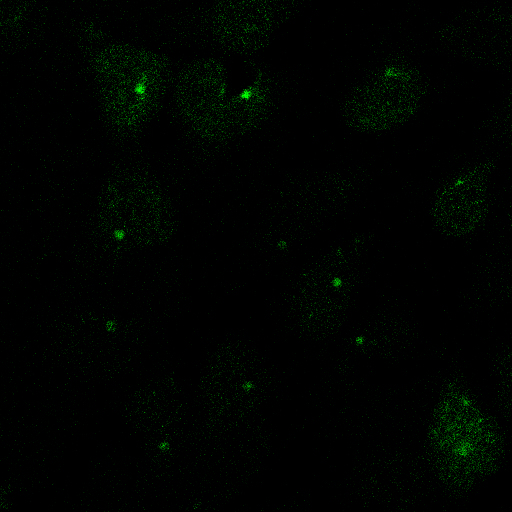

Supplement: Supplementary file 13 — Figure 3 (OLD) [file 41467_2023_42015_MOESM13_ESM.zip › Figure 2/Figure2c/Con_508.jpg]

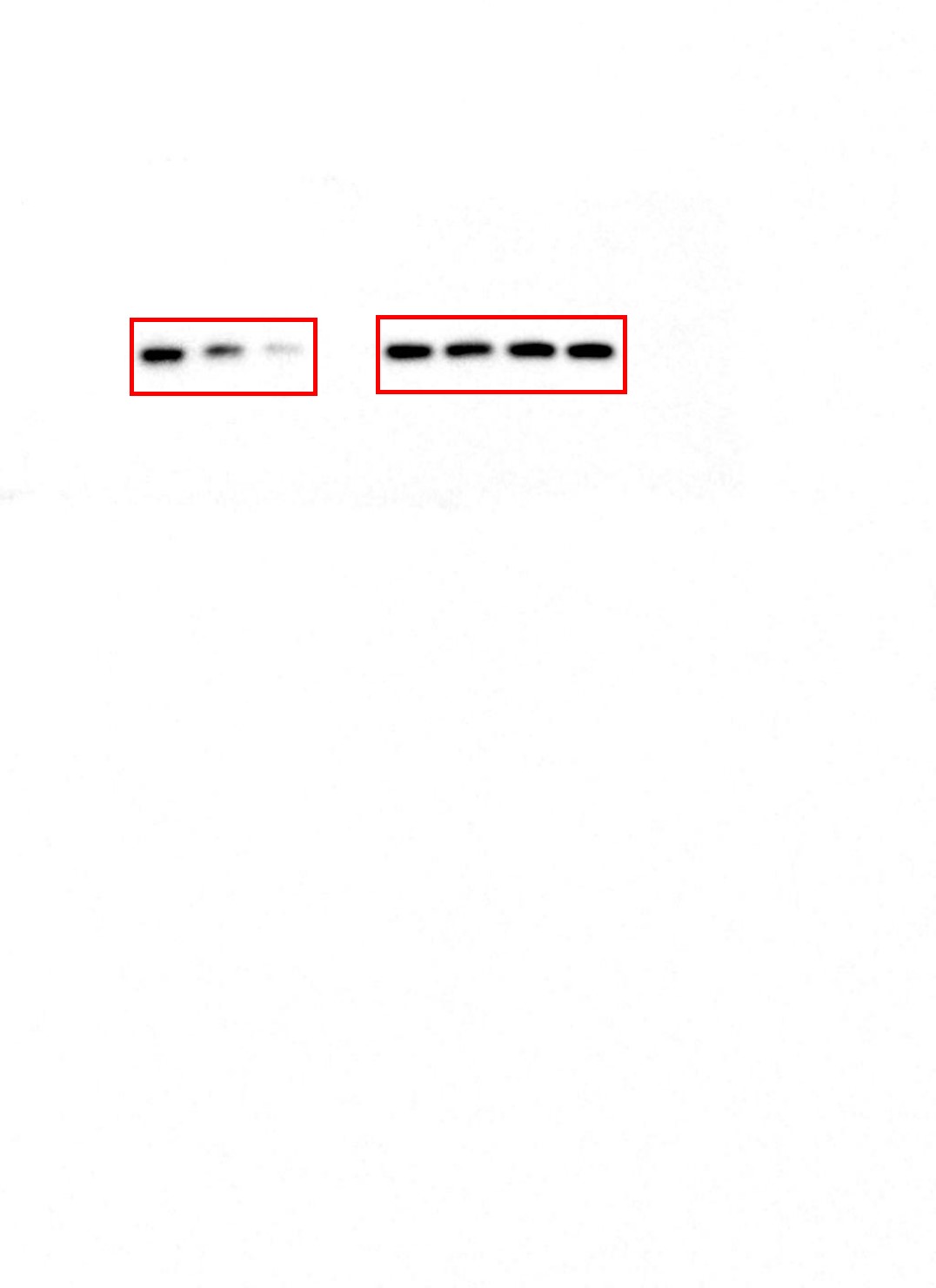

Supplement: Supplementary file 13 — Figure 3 (OLD) [file 41467_2023_42015_MOESM13_ESM.zip › Figure 2/Figure2e/b-actin.jpg]

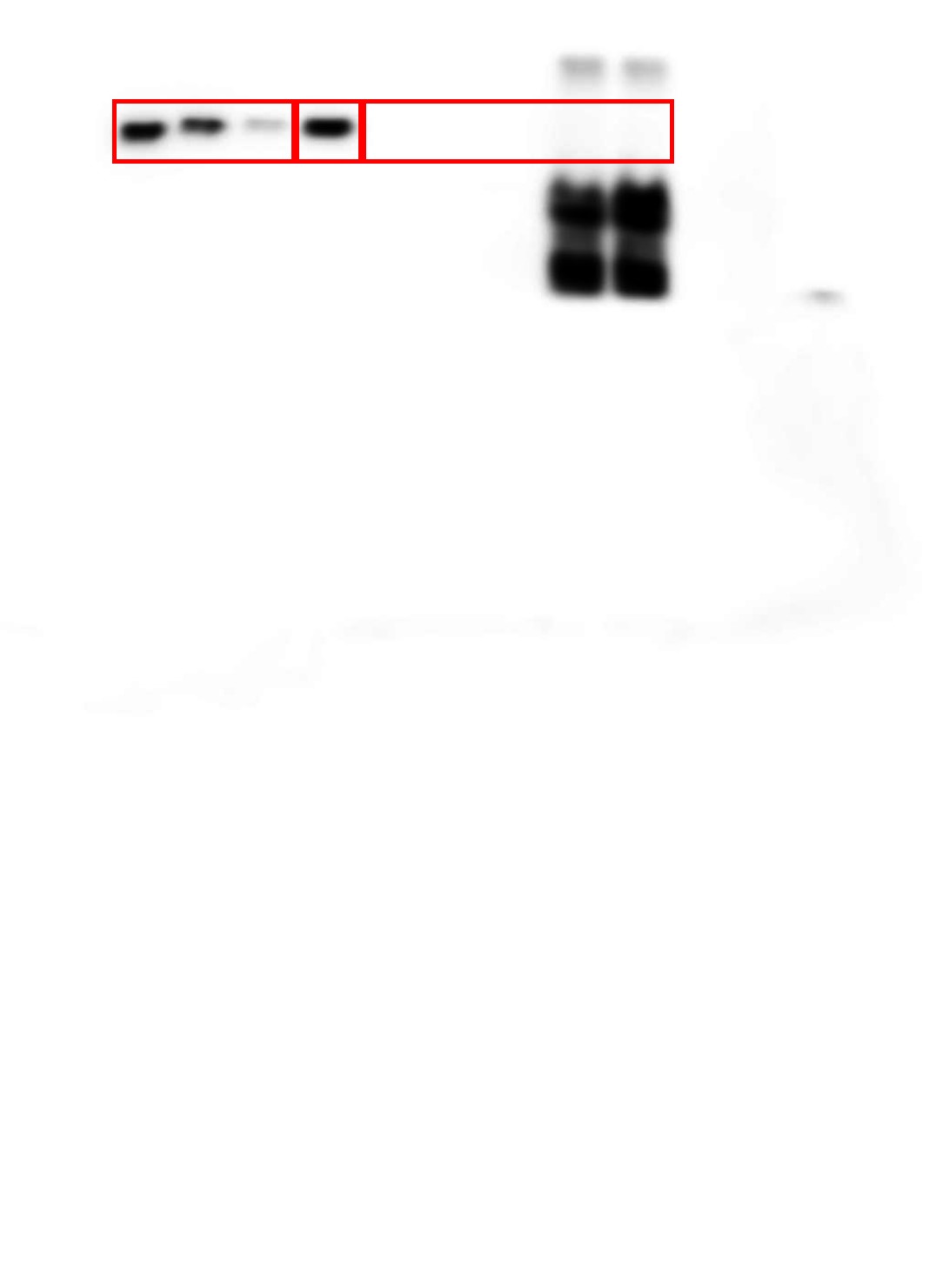

Supplement: Supplementary file 13 — Figure 3 (OLD) [file 41467_2023_42015_MOESM13_ESM.zip › Figure 2/Figure2f/b-actin.jpg]

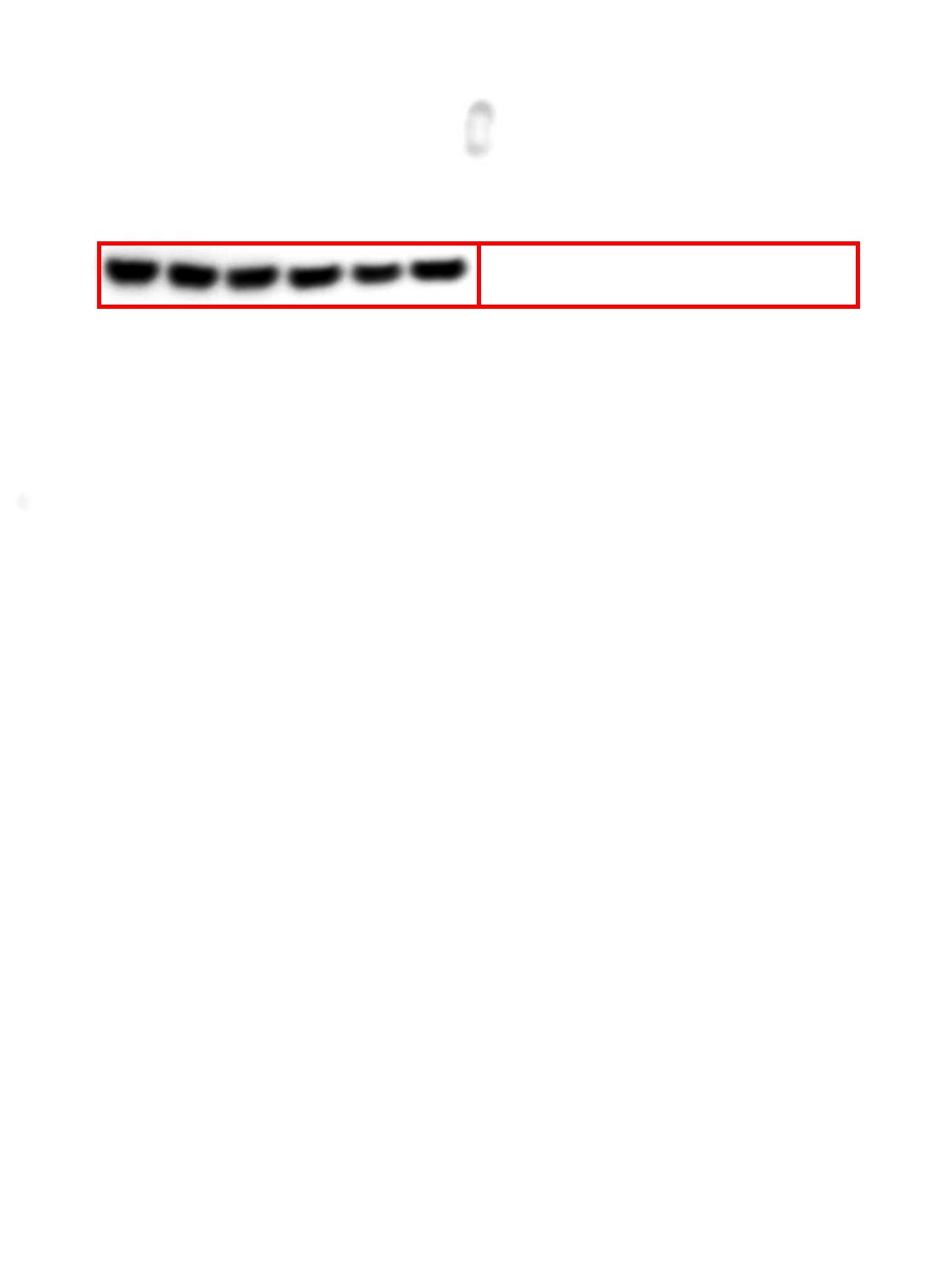

Supplement: Supplementary file 13 — Figure 3 (OLD) [file 41467_2023_42015_MOESM13_ESM.zip › Figure 3/Figure3a/b-actin.jpg]

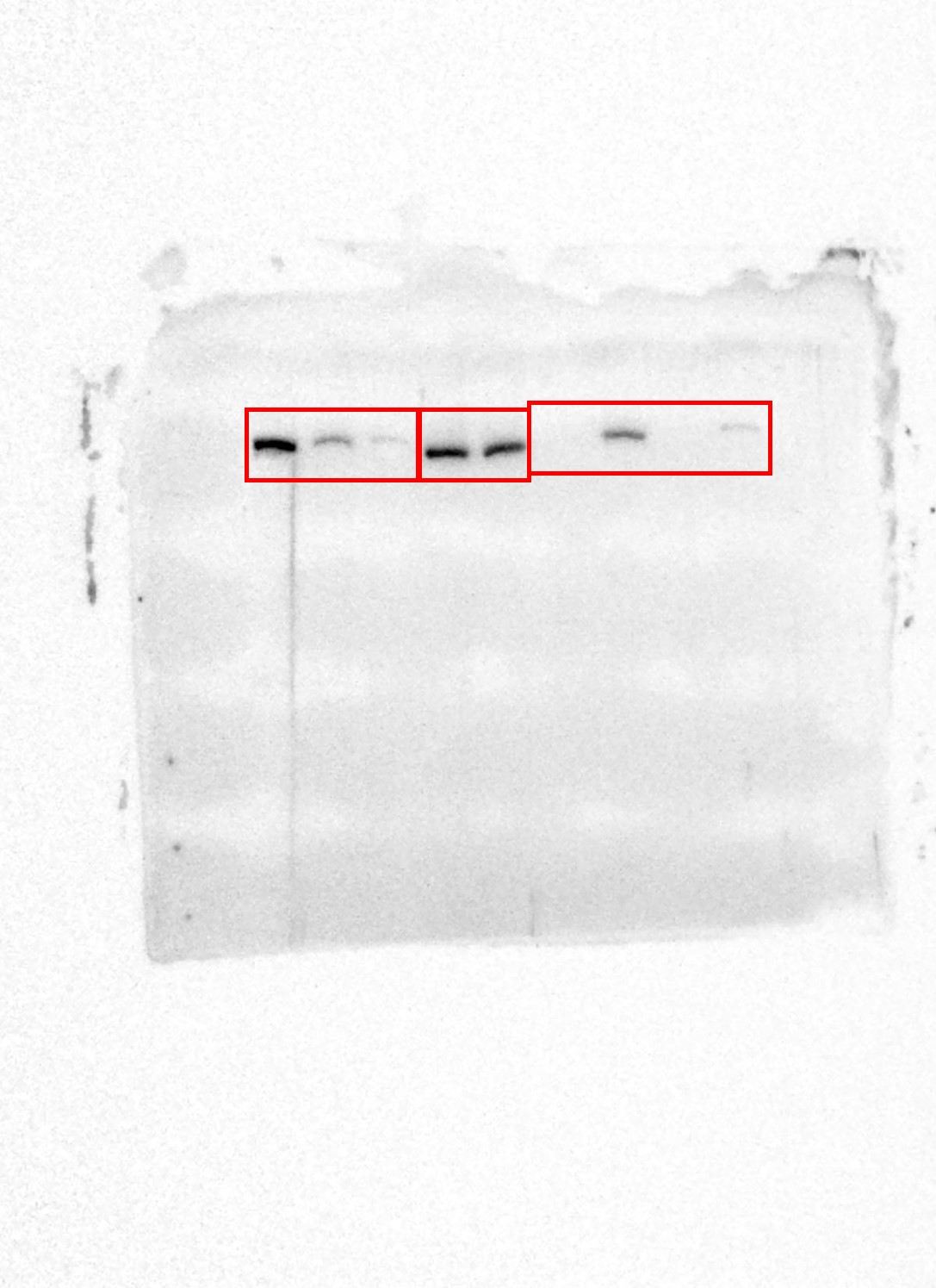

Supplement: Supplementary file 13 — Figure 3 (OLD) [file 41467_2023_42015_MOESM13_ESM.zip › Figure 8/Figure 8a/Dynein.jpg]

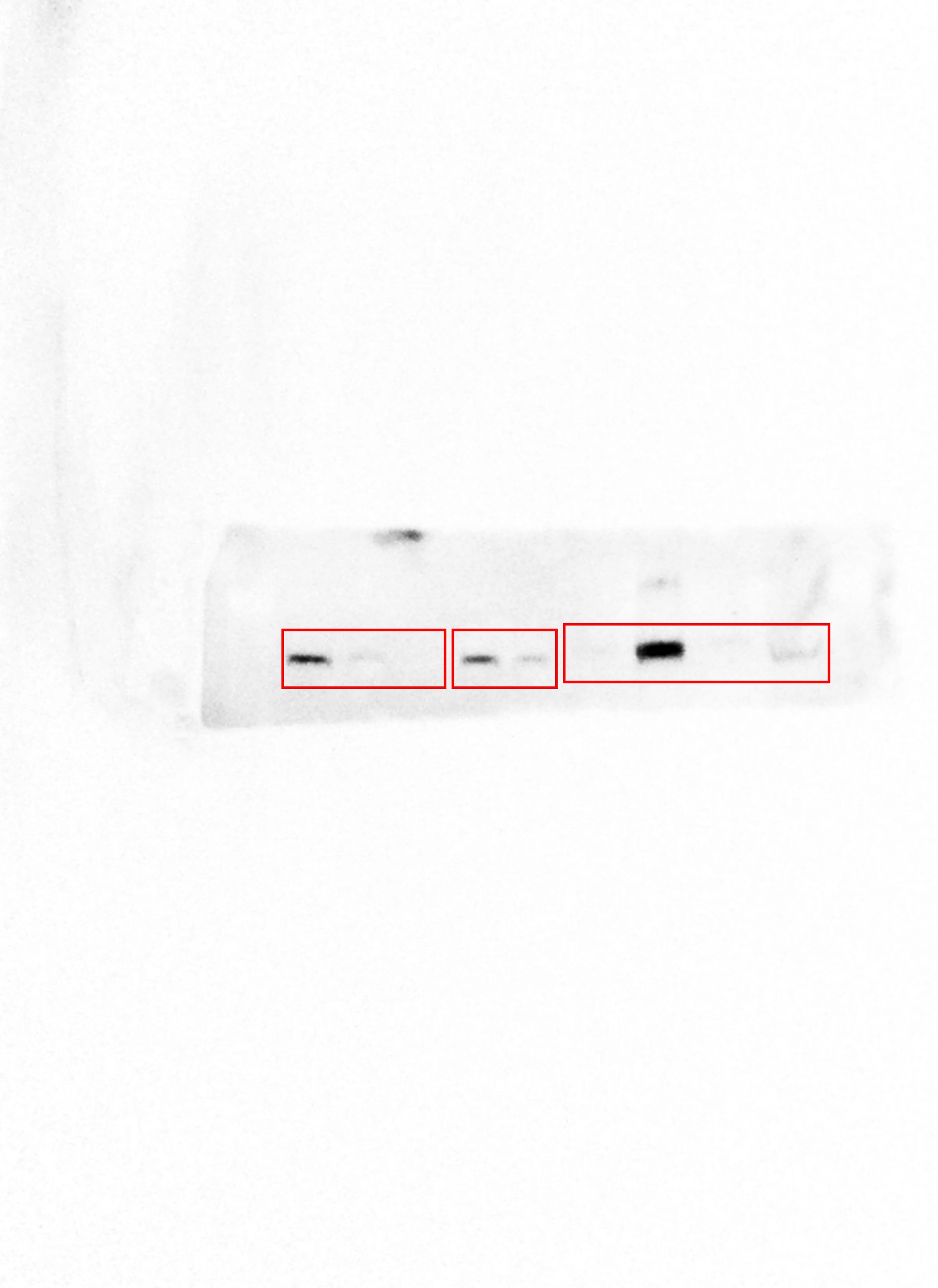

Supplement: Supplementary file 13 — Figure 3 (OLD) [file 41467_2023_42015_MOESM13_ESM.zip › Figure 8/Figure 8a/YTHDF2.jpg]

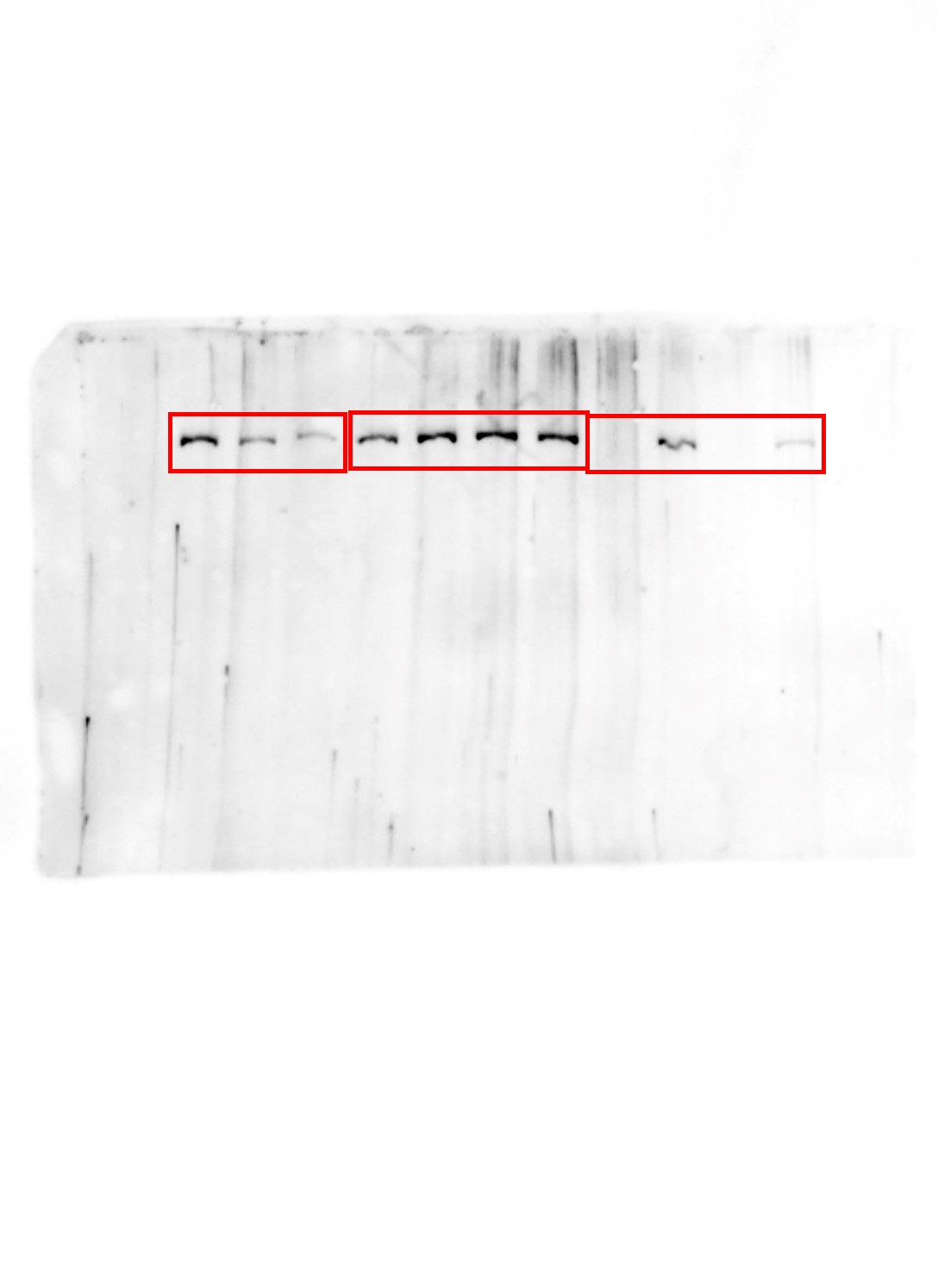

Supplement: Supplementary file 13 — Figure 3 (OLD) [file 41467_2023_42015_MOESM13_ESM.zip › Figure 8/Figure 8b/Dynein.jpg]

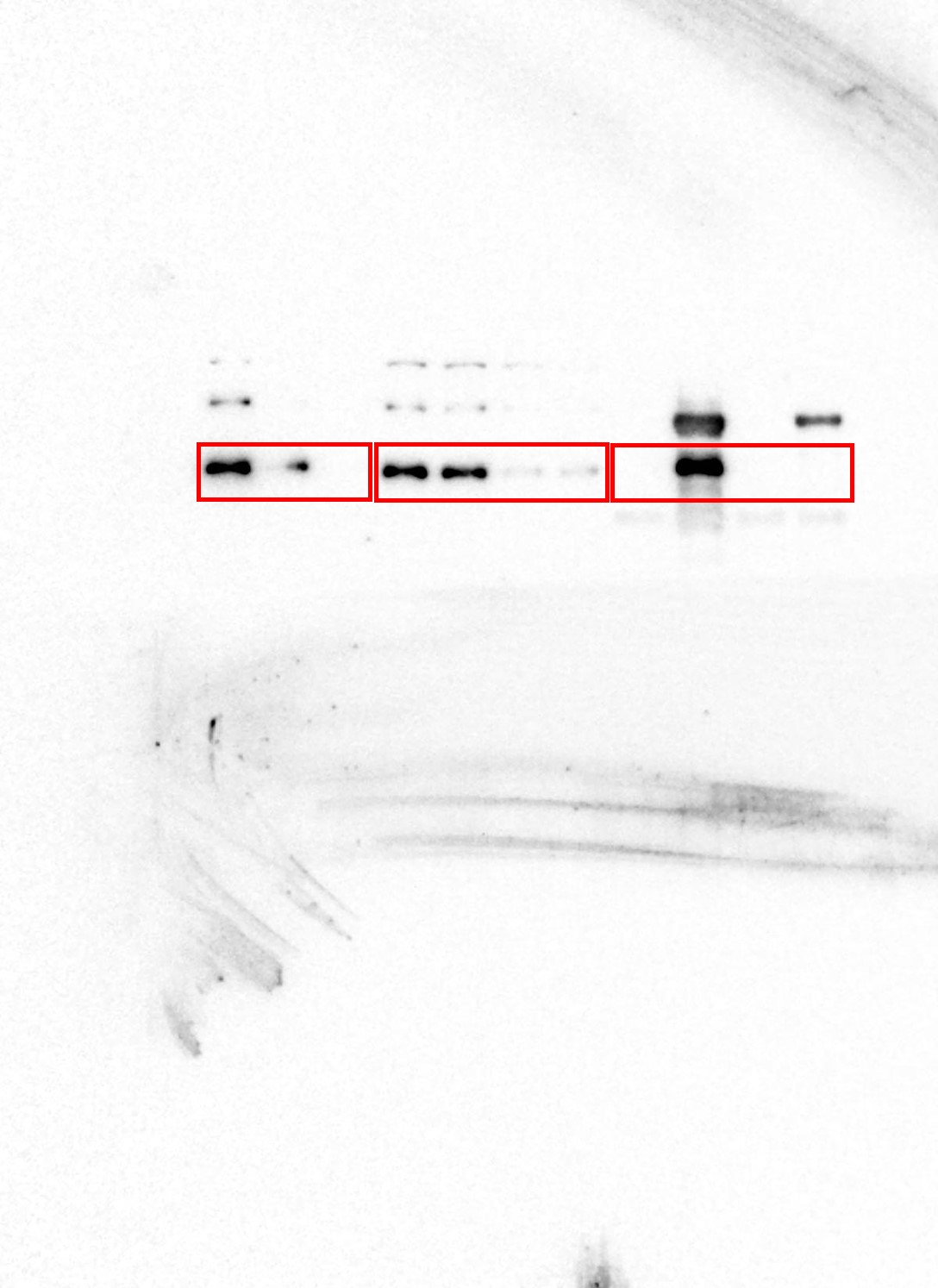

Supplement: Supplementary file 13 — Figure 3 (OLD) [file 41467_2023_42015_MOESM13_ESM.zip › Figure 8/Figure 8b/YTHDF2.jpg]

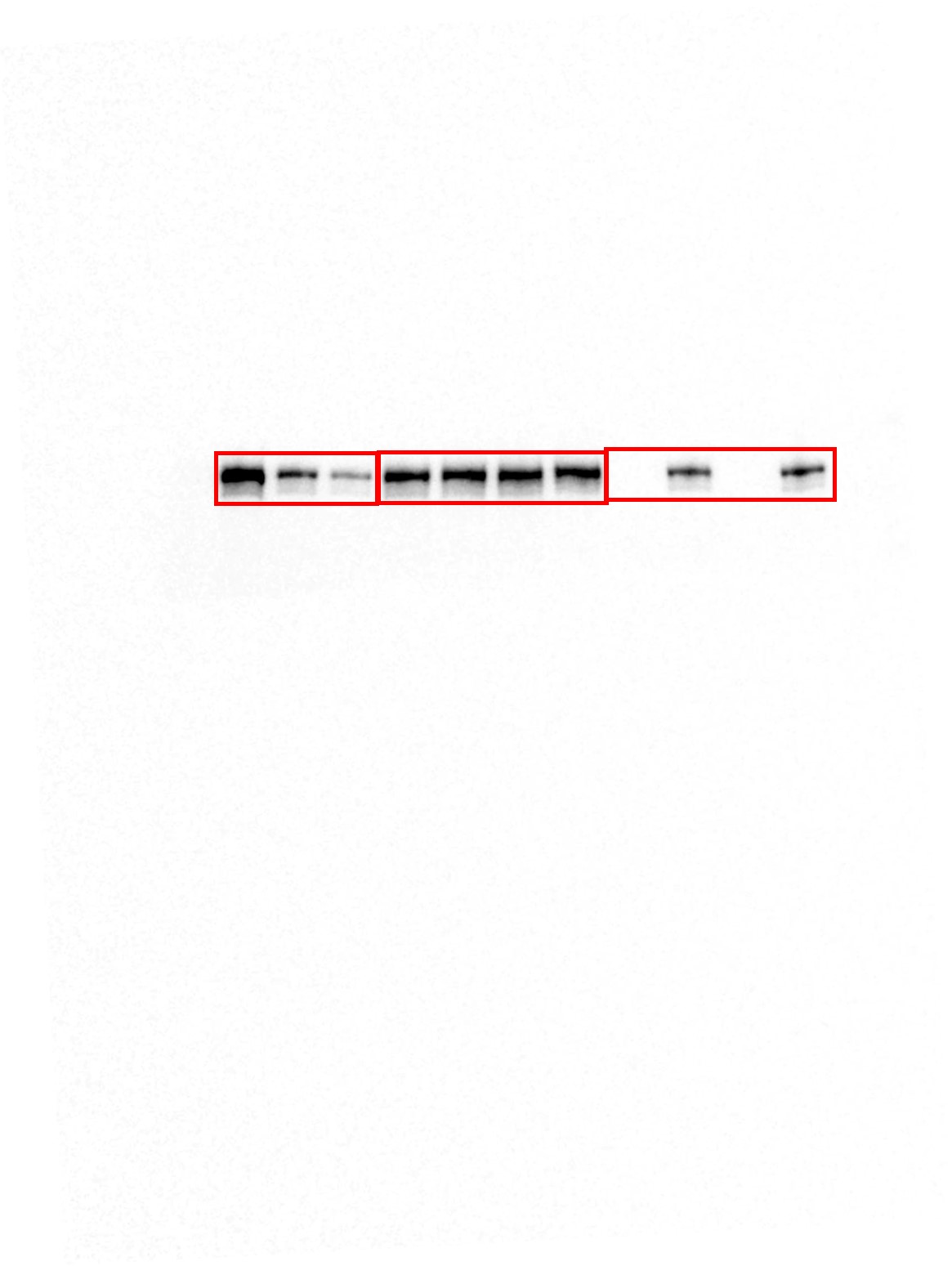

Supplement: Supplementary file 13 — Figure 3 (OLD) [file 41467_2023_42015_MOESM13_ESM.zip › Supplementary Fig. 7/DCTN1.jpg]

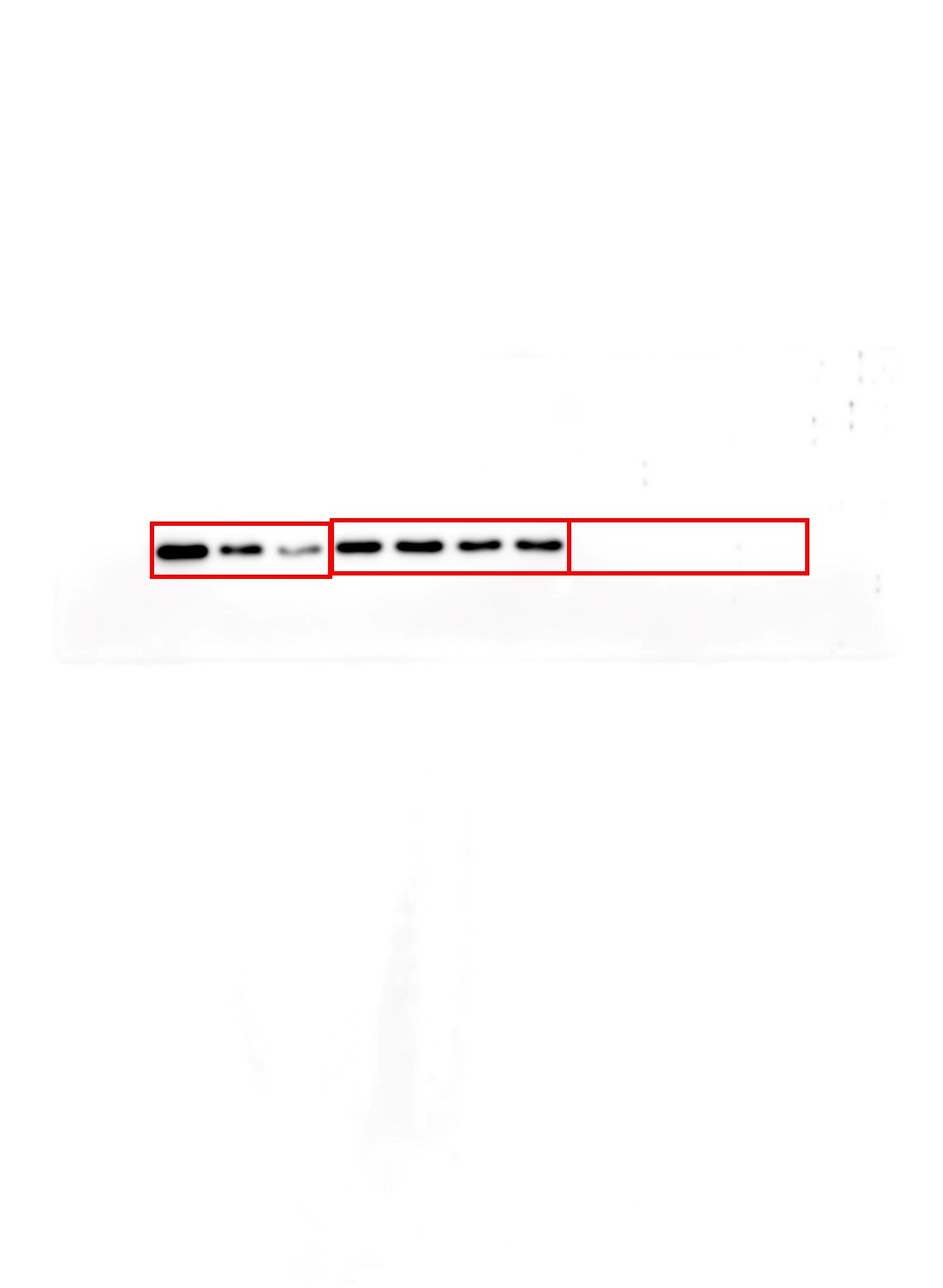

Supplement: Supplementary file 13 — Figure 3 (OLD) [file 41467_2023_42015_MOESM13_ESM.zip › Supplementary Fig. 7/GAPDH.jpg]

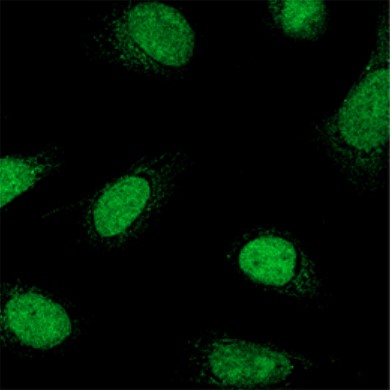

Supplement: Supplementary file 13 — Figure 3 (OLD) [file 41467_2023_42015_MOESM13_ESM.zip › Figure 1/Figure1a/Con_puro.jpg]

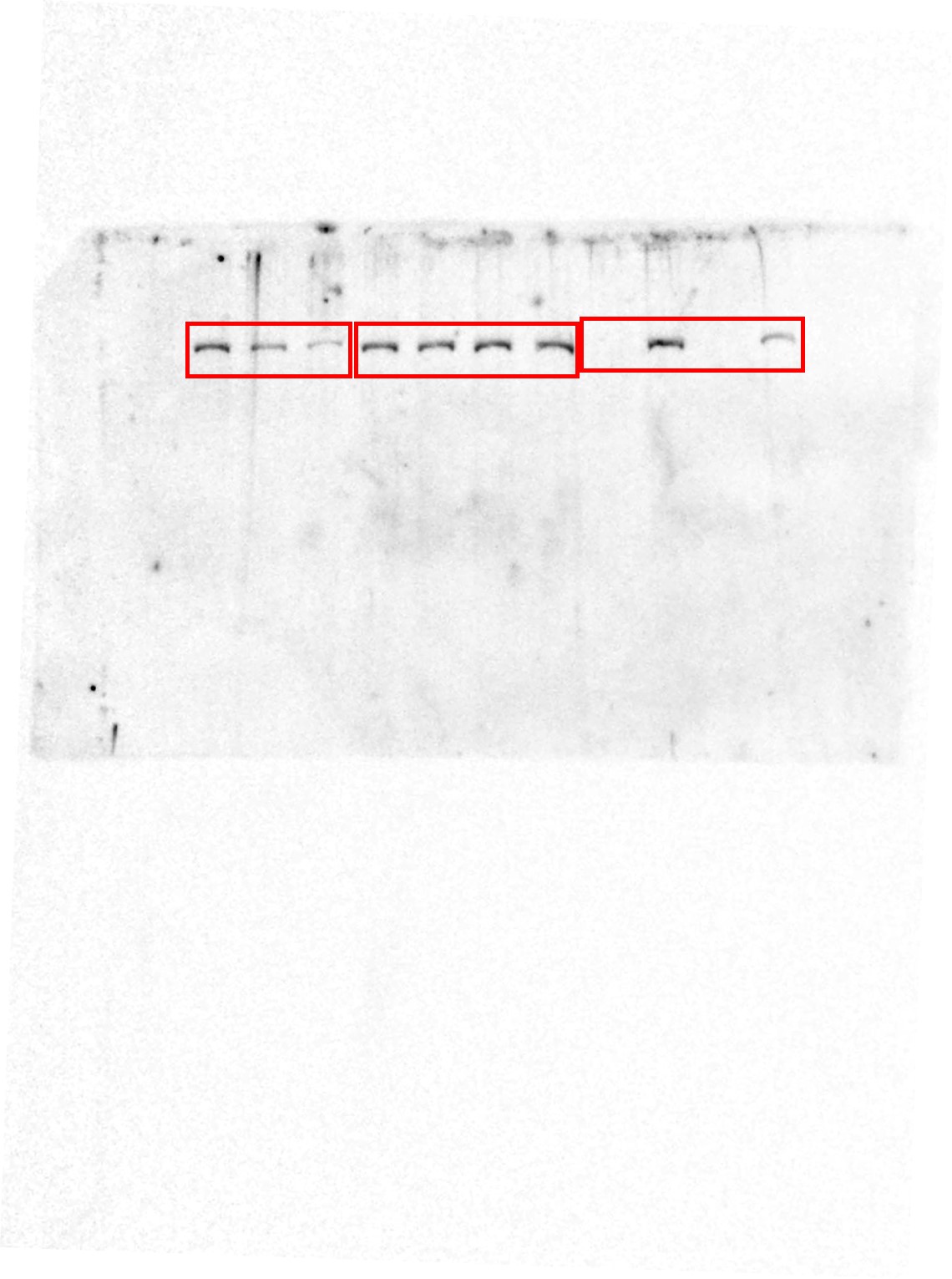

Supplement: Supplementary file 13 — Figure 3 (OLD) [file 41467_2023_42015_MOESM13_ESM.zip › Supplementary Fig. 7/Dynein.jpg]

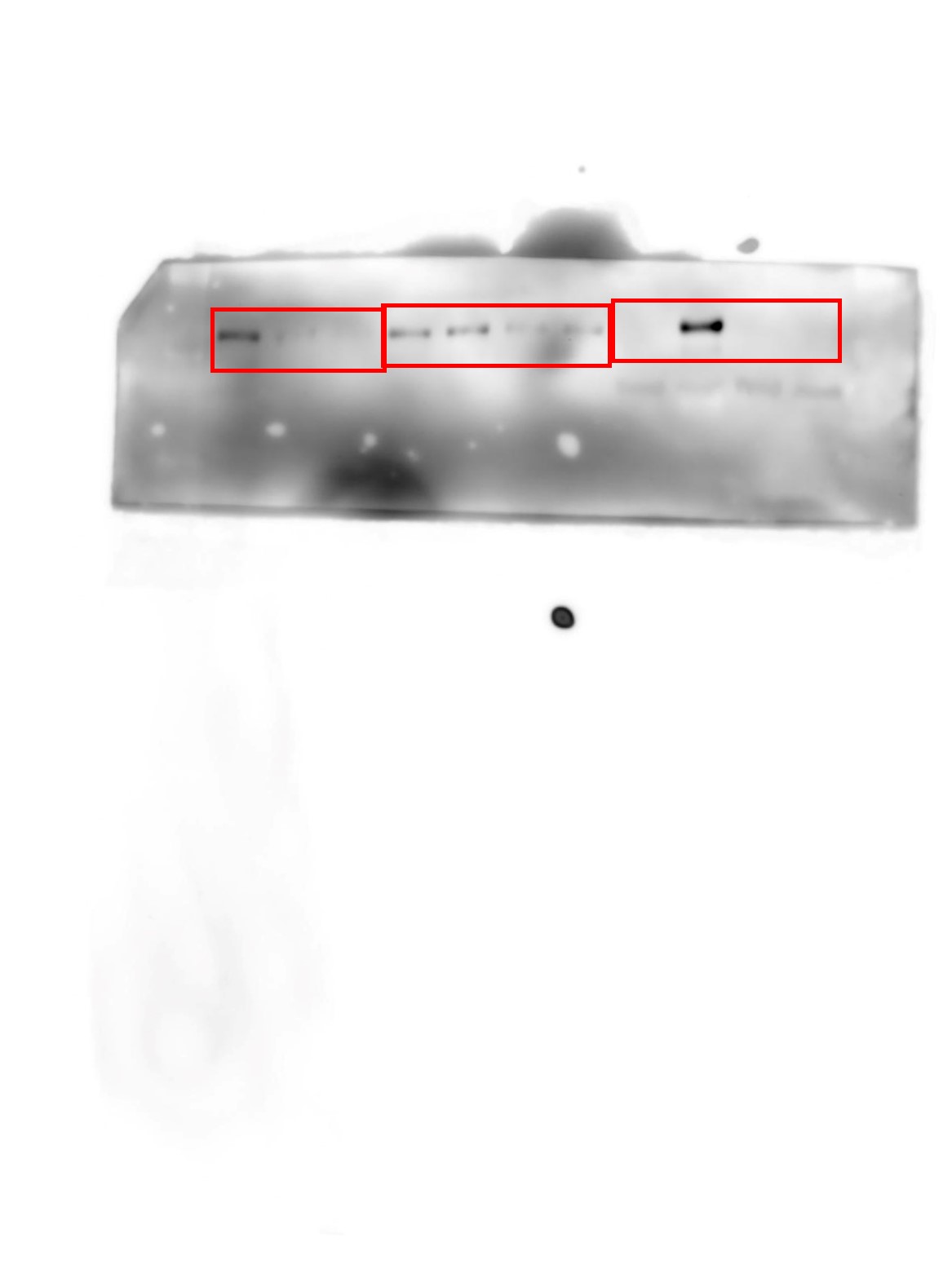

Supplement: Supplementary file 13 — Figure 3 (OLD) [file 41467_2023_42015_MOESM13_ESM.zip › Supplementary Fig. 7/YTHDF2.jpg]

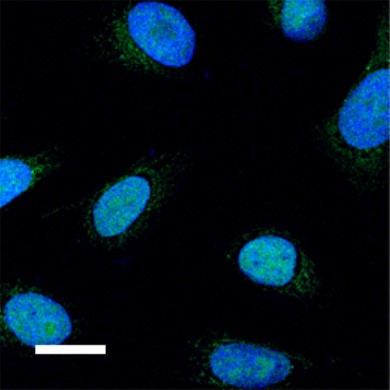

Supplement: Supplementary file 13 — Figure 3 (OLD) [file 41467_2023_42015_MOESM13_ESM.zip › Figure 1/Figure1a/Con_merged.jpg]

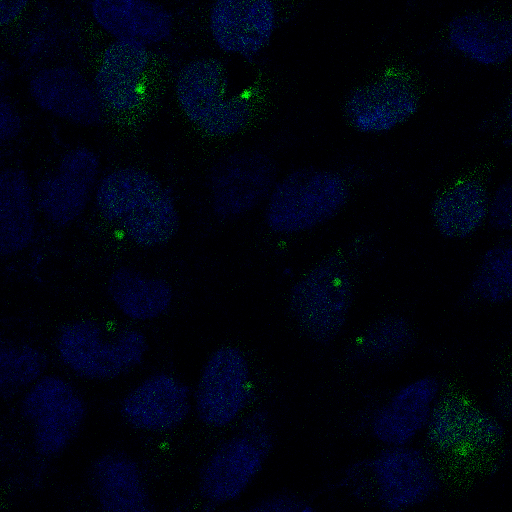

Supplement: Supplementary file 13 — Figure 3 (OLD) [file 41467_2023_42015_MOESM13_ESM.zip › Figure 2/Figure2c/Con_merged.jpg]

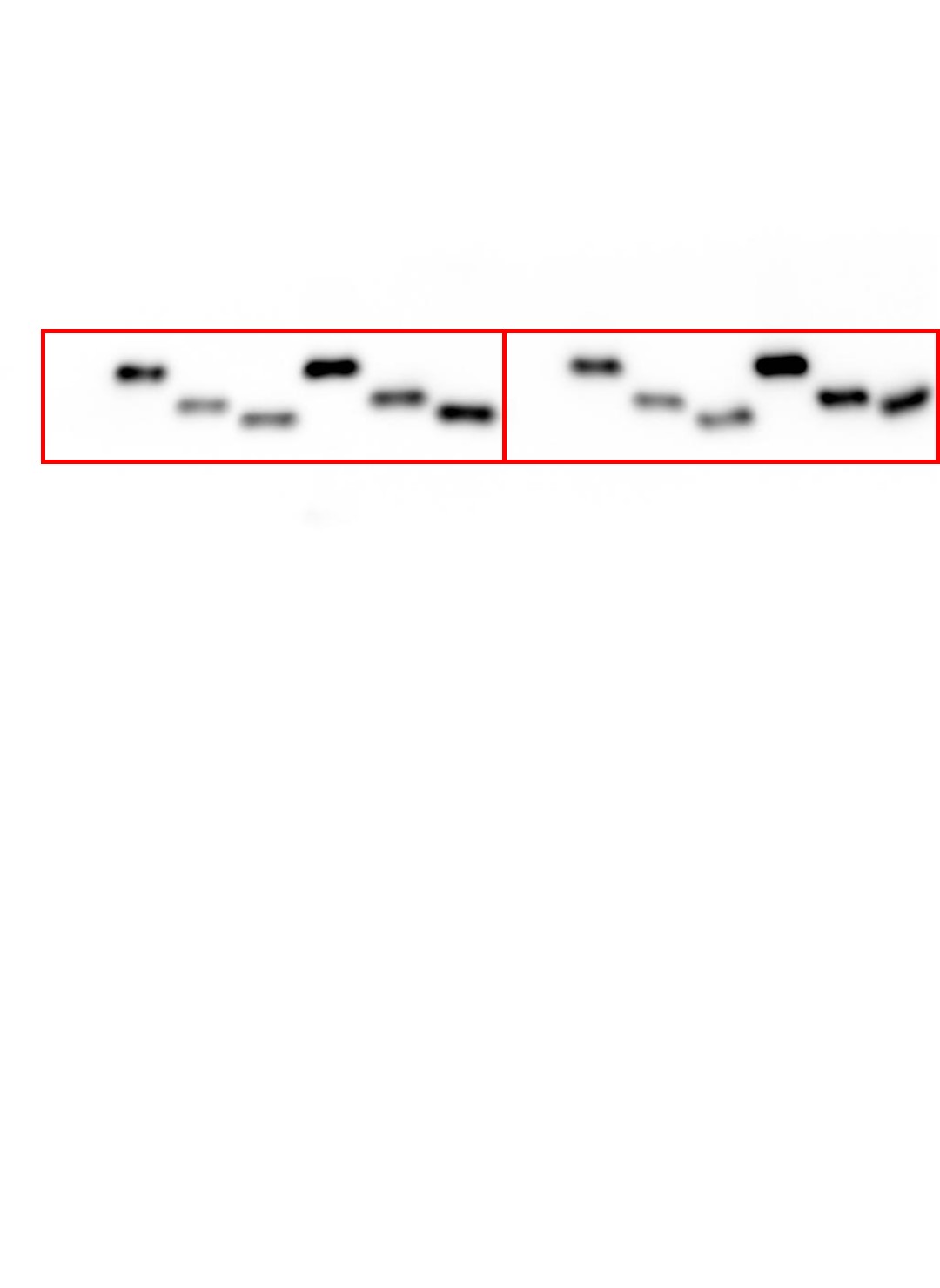

Supplement: Supplementary file 13 — Figure 3 (OLD) [file 41467_2023_42015_MOESM13_ESM.zip › Figure 3/Figure3b/Myc-YTHDF2.jpg]

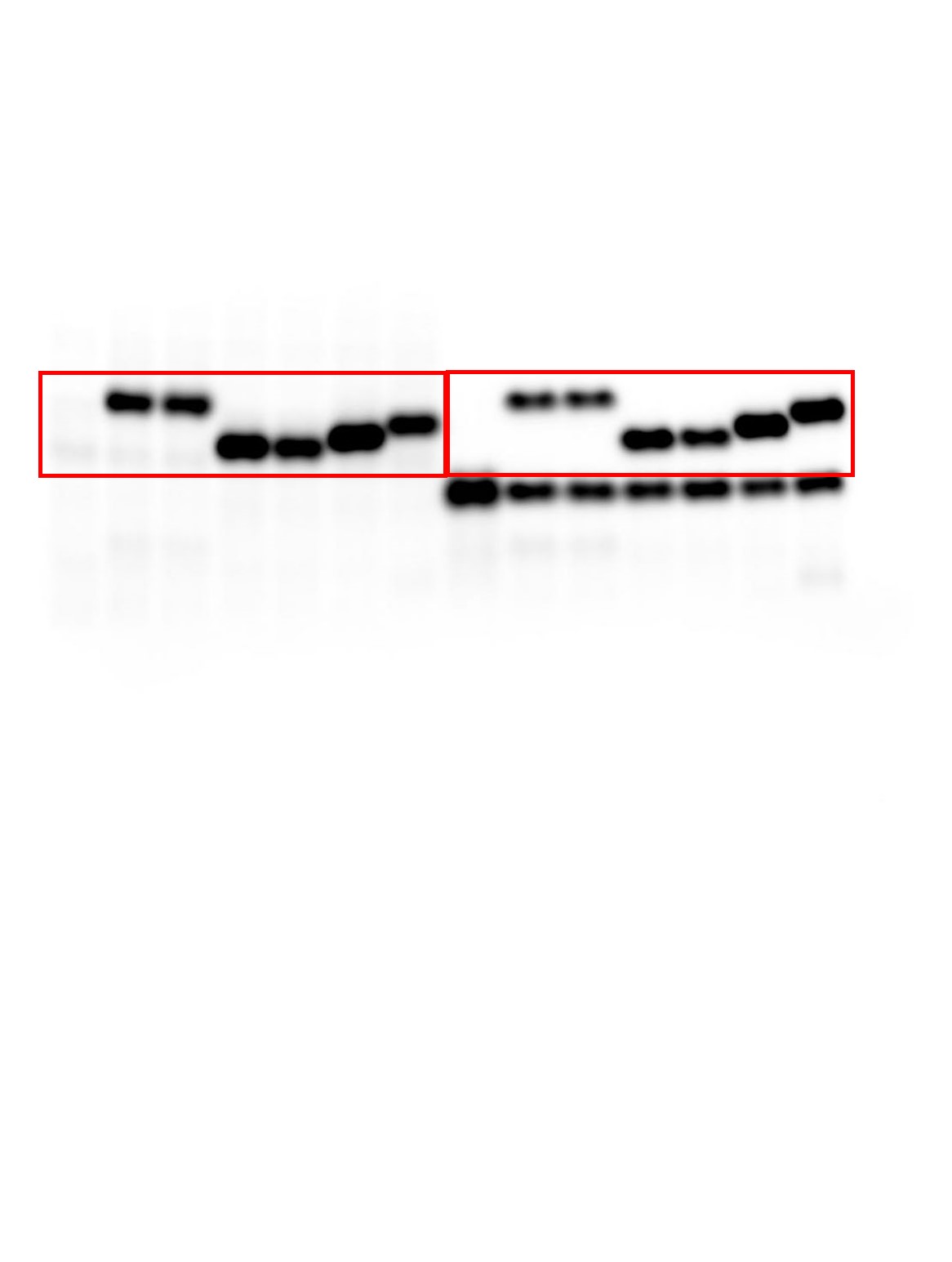

Supplement: Supplementary file 13 — Figure 3 (OLD) [file 41467_2023_42015_MOESM13_ESM.zip › Figure 4/Figure4a/Myc-YTHDF2.jpg]

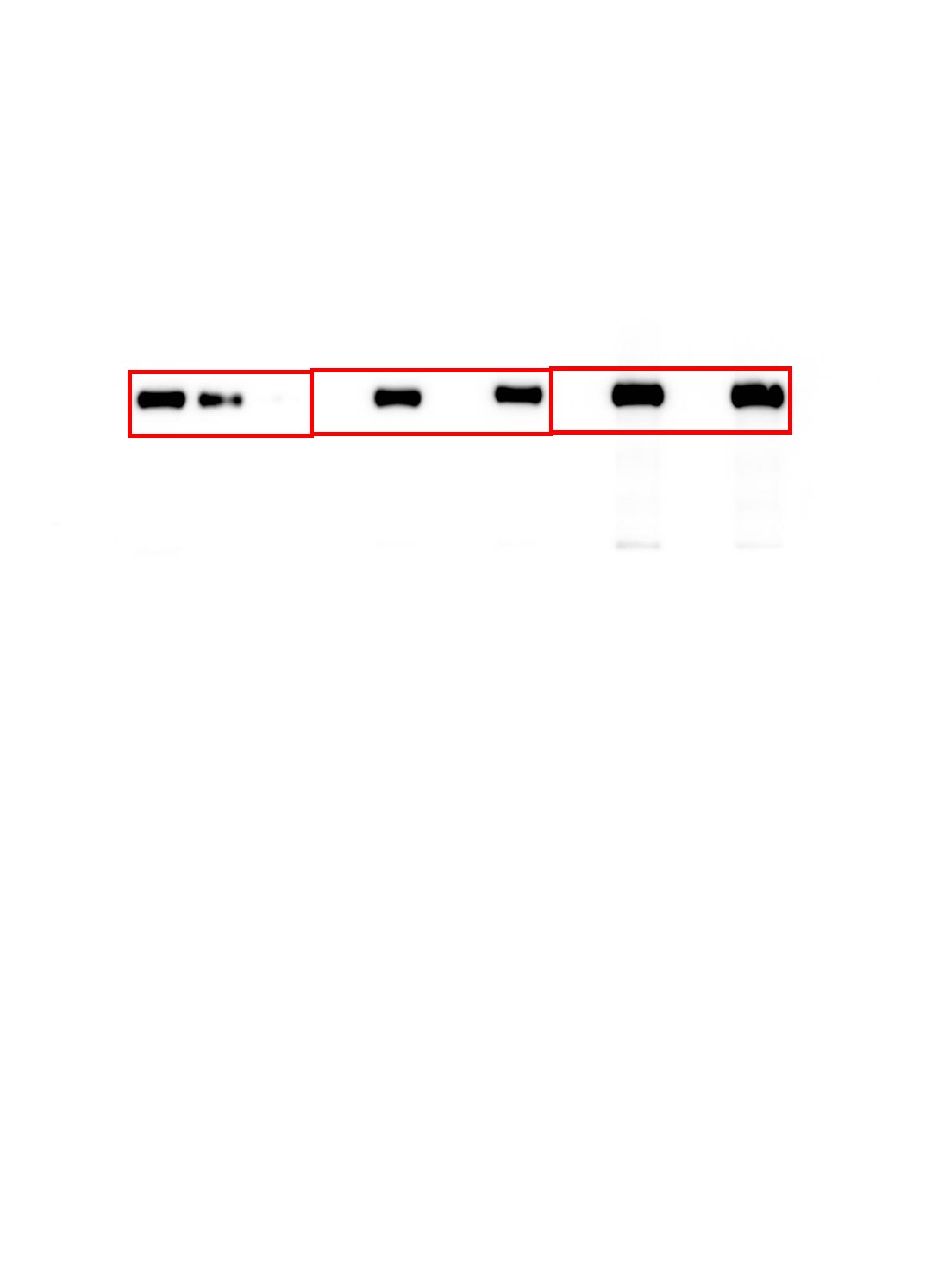

Supplement: Supplementary file 13 — Figure 3 (OLD) [file 41467_2023_42015_MOESM13_ESM.zip › Figure 8/Figure 8b/FLAG-CTIF.jpg]

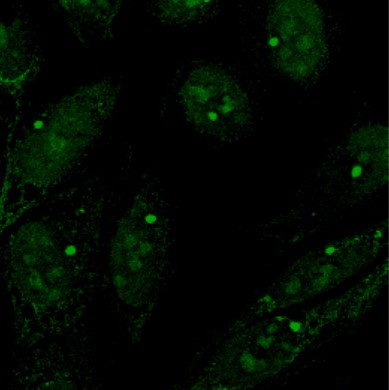

Supplement: Supplementary file 13 — Figure 3 (OLD) [file 41467_2023_42015_MOESM13_ESM.zip › Figure 1/Figure1a/Con_MG_puro.jpg]

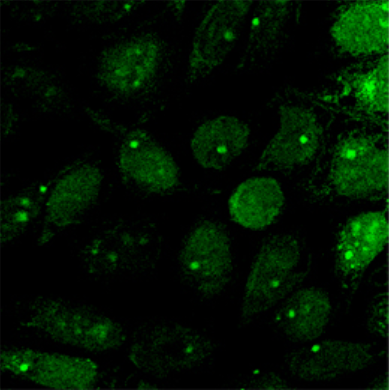

Supplement: Supplementary file 13 — Figure 3 (OLD) [file 41467_2023_42015_MOESM13_ESM.zip › Figure 2/Figure2a/Control_puro.png]

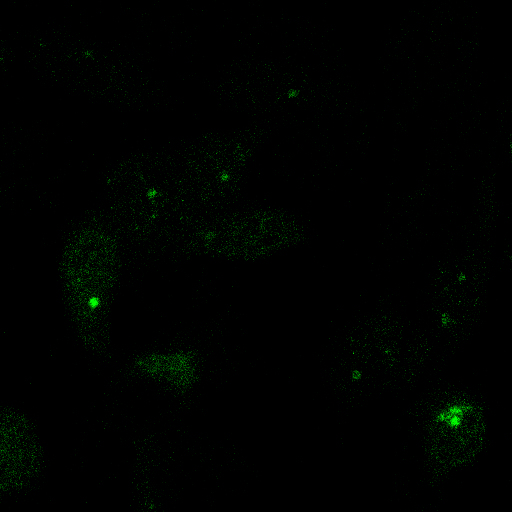

Supplement: Supplementary file 13 — Figure 3 (OLD) [file 41467_2023_42015_MOESM13_ESM.zip › Figure 2/Figure2c/YTHDF1si_508.jpg]

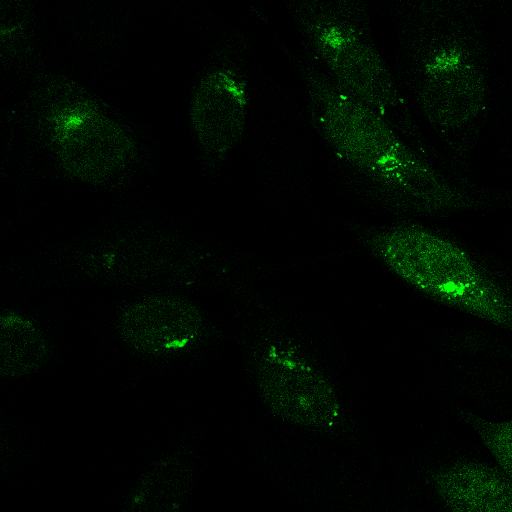

Supplement: Supplementary file 13 — Figure 3 (OLD) [file 41467_2023_42015_MOESM13_ESM.zip › Figure 2/Figure2c/YTHDF2si_508.jpg]

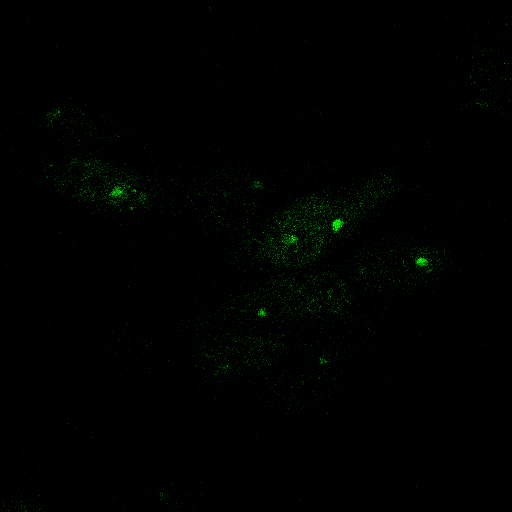

Supplement: Supplementary file 13 — Figure 3 (OLD) [file 41467_2023_42015_MOESM13_ESM.zip › Figure 2/Figure2c/YTHDF3si_508.jpg]

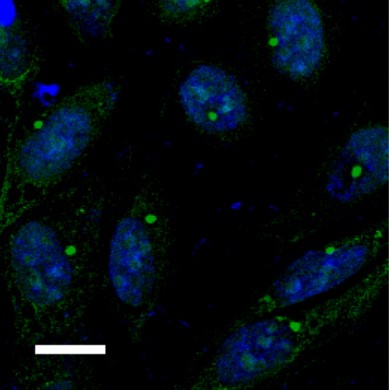

Supplement: Supplementary file 13 — Figure 3 (OLD) [file 41467_2023_42015_MOESM13_ESM.zip › Figure 1/Figure1a/Con_MG_merged.jpg]

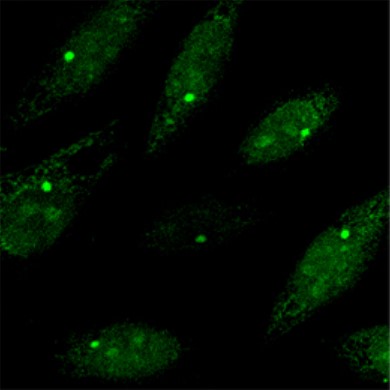

Supplement: Supplementary file 13 — Figure 3 (OLD) [file 41467_2023_42015_MOESM13_ESM.zip › Figure 1/Figure1a/FTOsi_MG_puro.jpg]

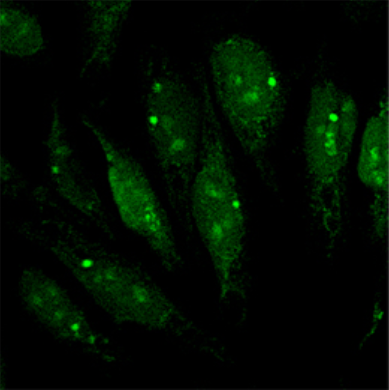

Supplement: Supplementary file 13 — Figure 3 (OLD) [file 41467_2023_42015_MOESM13_ESM.zip › Figure 2/Figure2a/YTHDF1si_puro.png]

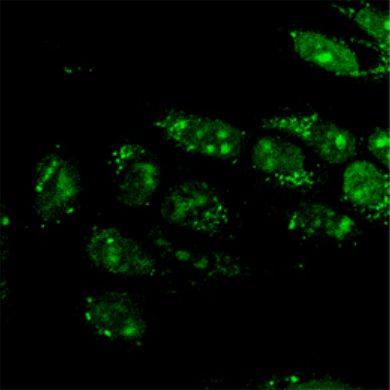

Supplement: Supplementary file 13 — Figure 3 (OLD) [file 41467_2023_42015_MOESM13_ESM.zip › Figure 2/Figure2a/YTHDF2si_puro.png]

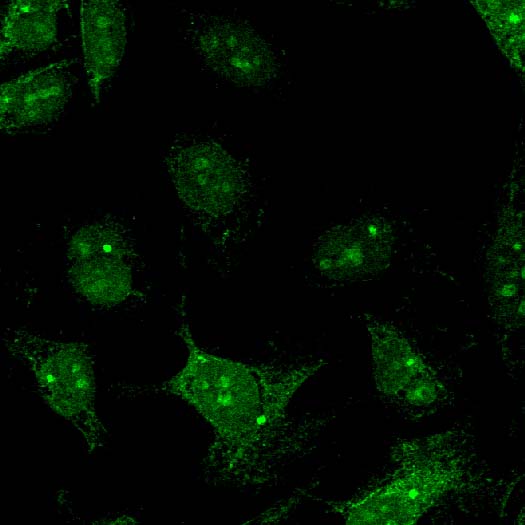

Supplement: Supplementary file 13 — Figure 3 (OLD) [file 41467_2023_42015_MOESM13_ESM.zip › Figure 2/Figure2a/YTHDF3si_puro.jpg]

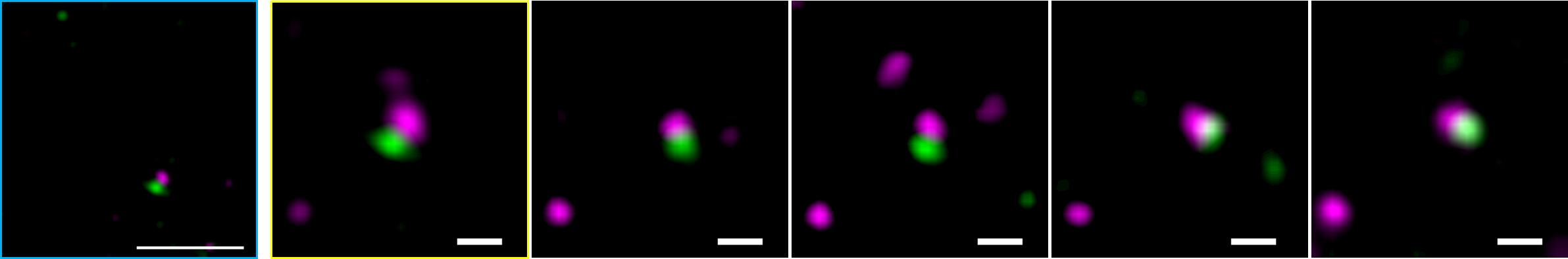

Supplement: Supplementary file 13 — Figure 3 (OLD) [file 41467_2023_42015_MOESM13_ESM.zip › Figure 7/Figure 7d/YTHDF2, Gpx1.png]

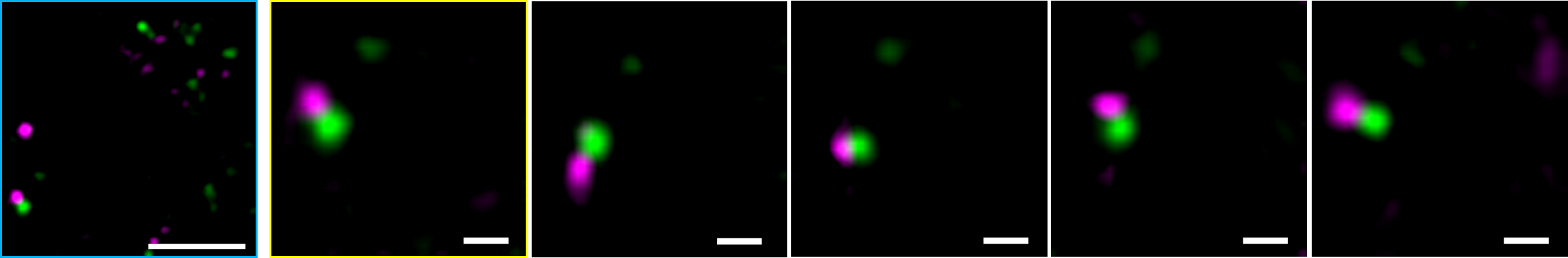

Supplement: Supplementary file 13 — Figure 3 (OLD) [file 41467_2023_42015_MOESM13_ESM.zip › Figure 7/Figure 7d/YTHDF2, SOD1.png]

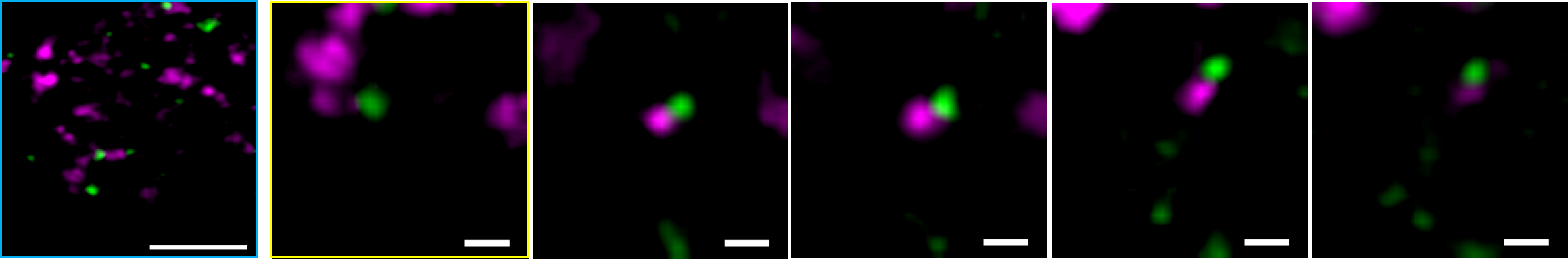

Supplement: Supplementary file 13 — Figure 3 (OLD) [file 41467_2023_42015_MOESM13_ESM.zip › Figure 7/Figure 7d/YTHDF2, SYN1.png]

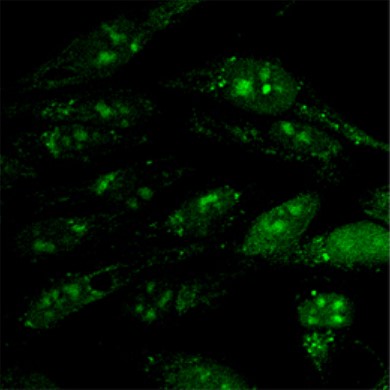

Supplement: Supplementary file 13 — Figure 3 (OLD) [file 41467_2023_42015_MOESM13_ESM.zip › Figure 1/Figure1a/DCTNsi_MG_puro.jpg]

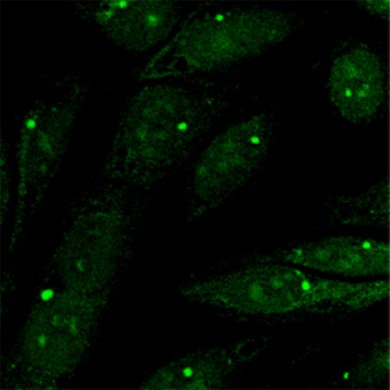

Supplement: Supplementary file 13 — Figure 3 (OLD) [file 41467_2023_42015_MOESM13_ESM.zip › Figure 1/Figure1a/SMG6si_MG_puro.jpg]

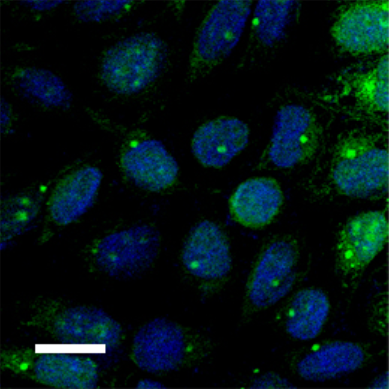

Supplement: Supplementary file 13 — Figure 3 (OLD) [file 41467_2023_42015_MOESM13_ESM.zip › Figure 2/Figure2a/Control_merged.png]

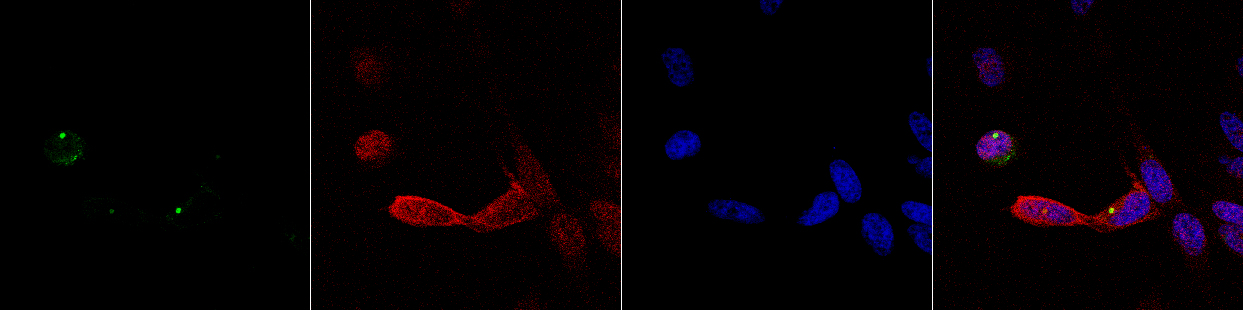

Supplement: Supplementary file 13 — Figure 3 (OLD) [file 41467_2023_42015_MOESM13_ESM.zip › Figure 4/Figure4b/YTHDF2si_R527A.jpg]

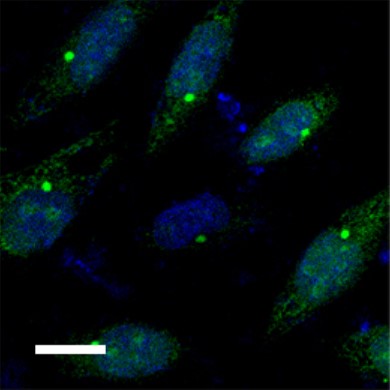

Supplement: Supplementary file 13 — Figure 3 (OLD) [file 41467_2023_42015_MOESM13_ESM.zip › Figure 1/Figure1a/FTOsi_MG_merged.jpg]

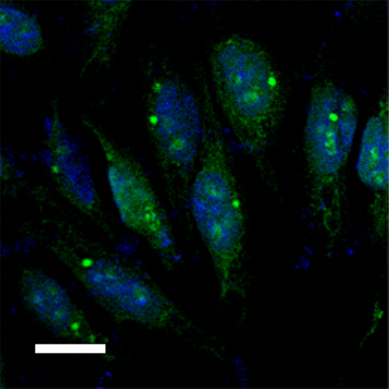

Supplement: Supplementary file 13 — Figure 3 (OLD) [file 41467_2023_42015_MOESM13_ESM.zip › Figure 2/Figure2a/YTHDF1si_merged.png]

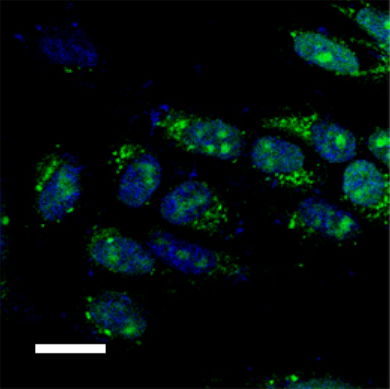

Supplement: Supplementary file 13 — Figure 3 (OLD) [file 41467_2023_42015_MOESM13_ESM.zip › Figure 2/Figure2a/YTHDF2si_merged.png]

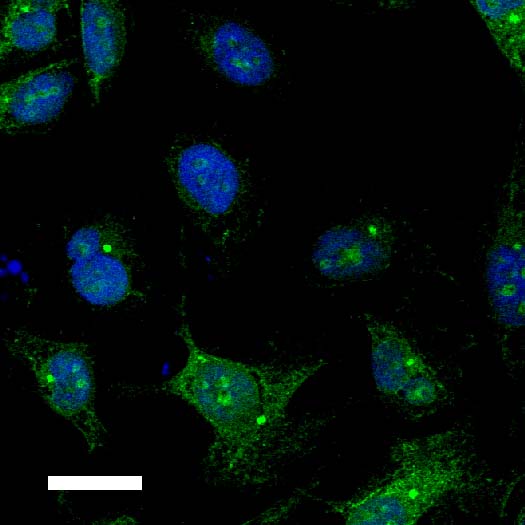

Supplement: Supplementary file 13 — Figure 3 (OLD) [file 41467_2023_42015_MOESM13_ESM.zip › Figure 2/Figure2a/YTHDF3si_merged.jpg]

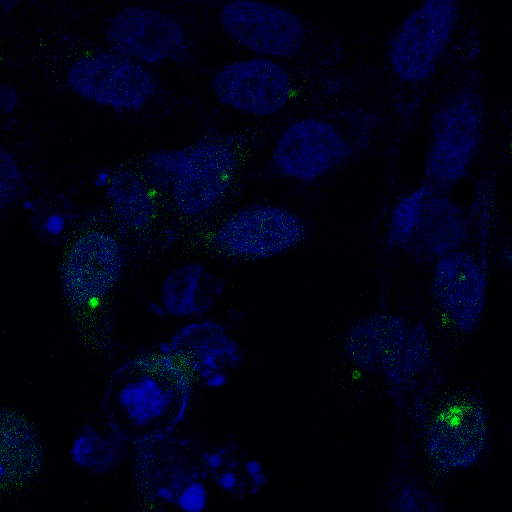

Supplement: Supplementary file 13 — Figure 3 (OLD) [file 41467_2023_42015_MOESM13_ESM.zip › Figure 2/Figure2c/YTHDF1si_merged.jpg]

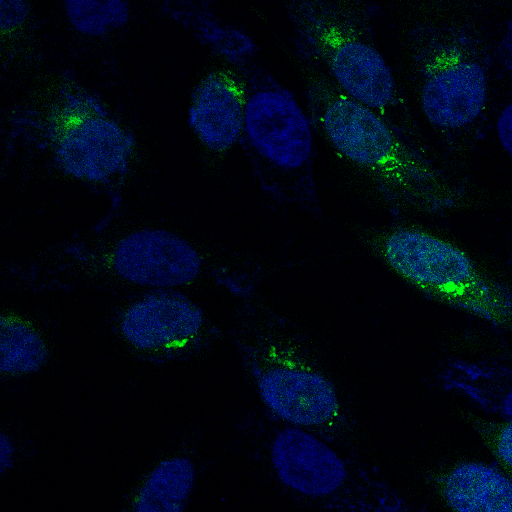

Supplement: Supplementary file 13 — Figure 3 (OLD) [file 41467_2023_42015_MOESM13_ESM.zip › Figure 2/Figure2c/YTHDF2si_merged.jpg]

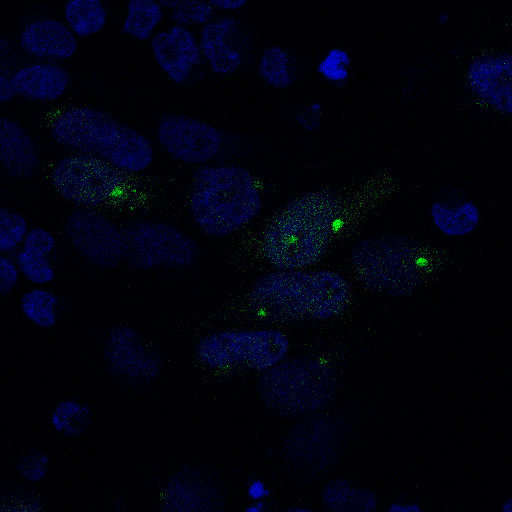

Supplement: Supplementary file 13 — Figure 3 (OLD) [file 41467_2023_42015_MOESM13_ESM.zip › Figure 2/Figure2c/YTHDF3si_merged.jpg]

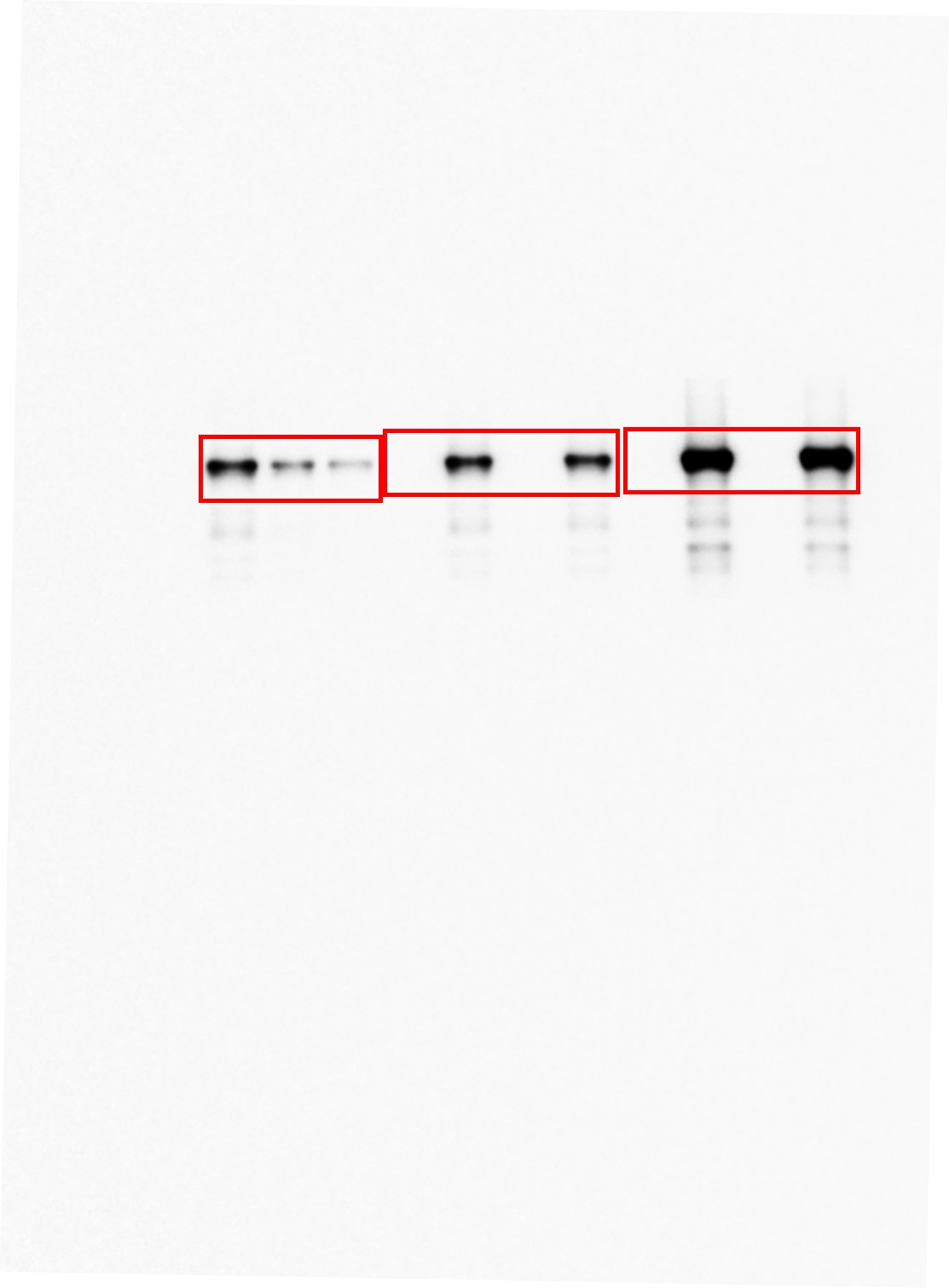

Supplement: Supplementary file 13 — Figure 3 (OLD) [file 41467_2023_42015_MOESM13_ESM.zip › Supplementary Fig. 7/3xFLAG-eEF1A1.jpg]

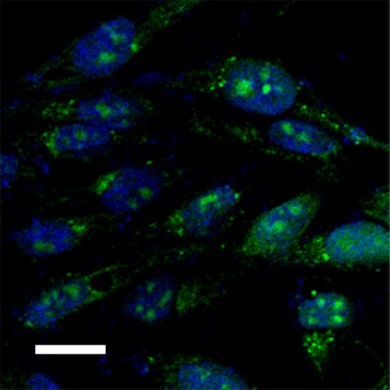

Supplement: Supplementary file 13 — Figure 3 (OLD) [file 41467_2023_42015_MOESM13_ESM.zip › Figure 1/Figure1a/DCTNsi_MG_merged.jpg]

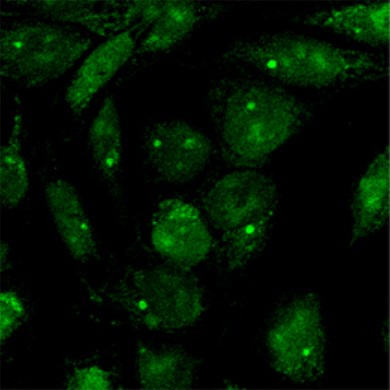

Supplement: Supplementary file 13 — Figure 3 (OLD) [file 41467_2023_42015_MOESM13_ESM.zip › Figure 1/Figure1a/METTL3si_MG_puro.jpg]

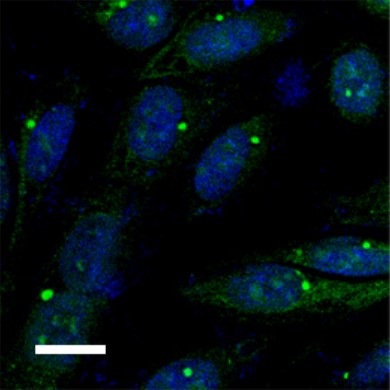

Supplement: Supplementary file 13 — Figure 3 (OLD) [file 41467_2023_42015_MOESM13_ESM.zip › Figure 1/Figure1a/SMG6si_MG_merged.jpg]

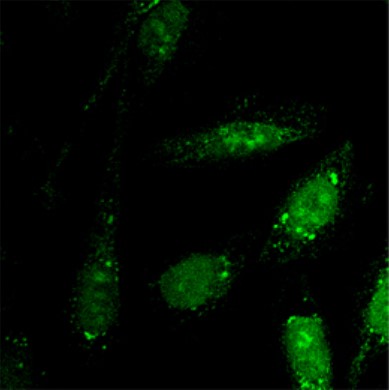

Supplement: Supplementary file 13 — Figure 3 (OLD) [file 41467_2023_42015_MOESM13_ESM.zip › Figure 1/Figure1a/YTHDF2si_MG_puro.jpg]

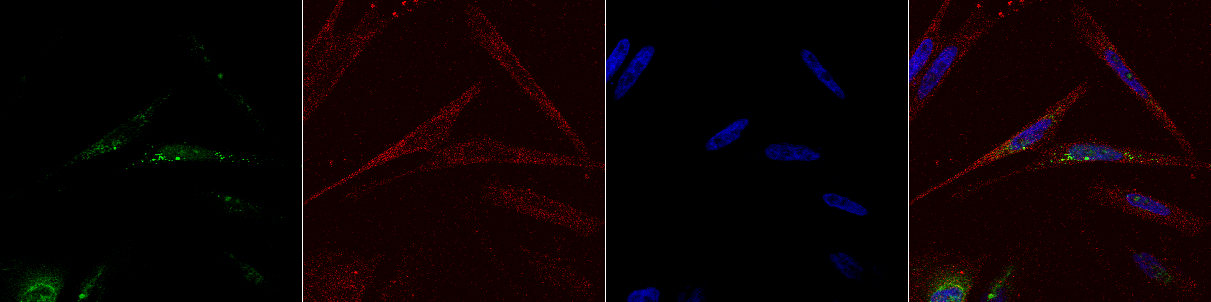

Supplement: Supplementary file 13 — Figure 3 (OLD) [file 41467_2023_42015_MOESM13_ESM.zip › Figure 4/Figure4b/YTHDF2si_Myc-GST.jpg]

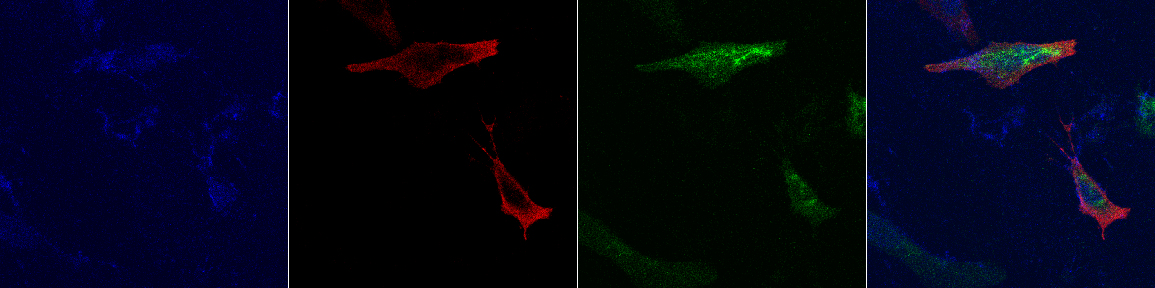

Supplement: Supplementary file 13 — Figure 3 (OLD) [file 41467_2023_42015_MOESM13_ESM.zip › Figure 4/Figure4b/YTHDF2si_101-168.jpg]

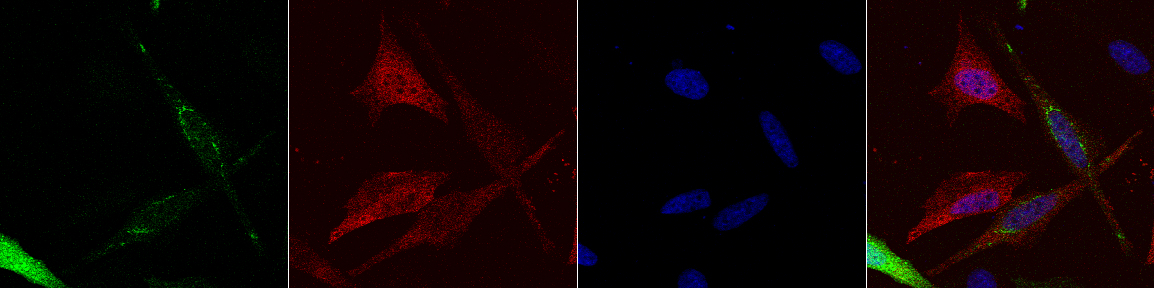

Supplement: Supplementary file 13 — Figure 3 (OLD) [file 41467_2023_42015_MOESM13_ESM.zip › Figure 4/Figure4b/YTHDF2si_101-200.jpg]

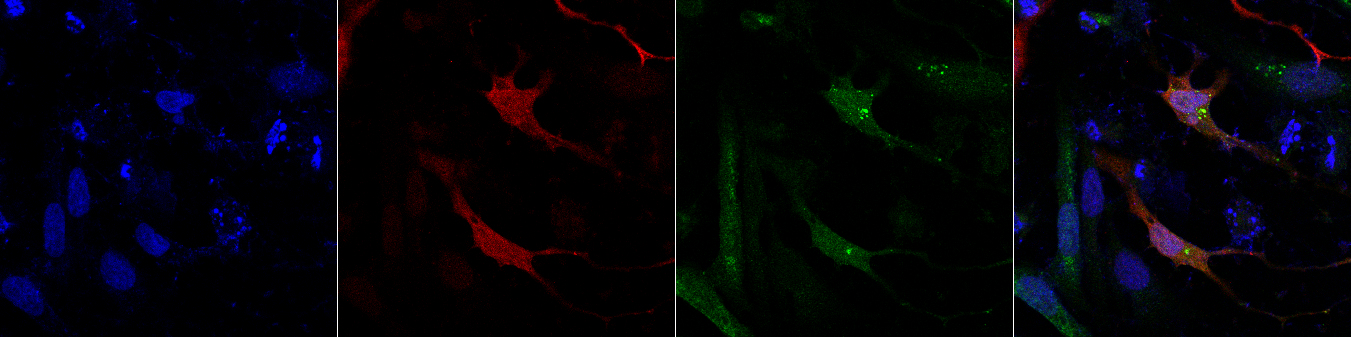

Supplement: Supplementary file 13 — Figure 3 (OLD) [file 41467_2023_42015_MOESM13_ESM.zip › Figure 4/Figure4b/YTHDF2si_169-200.jpg]

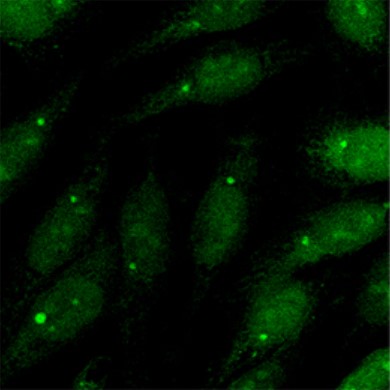

Supplement: Supplementary file 13 — Figure 3 (OLD) [file 41467_2023_42015_MOESM13_ESM.zip › Figure 1/Figure1a/METTL14si_MG_puro.jpg]

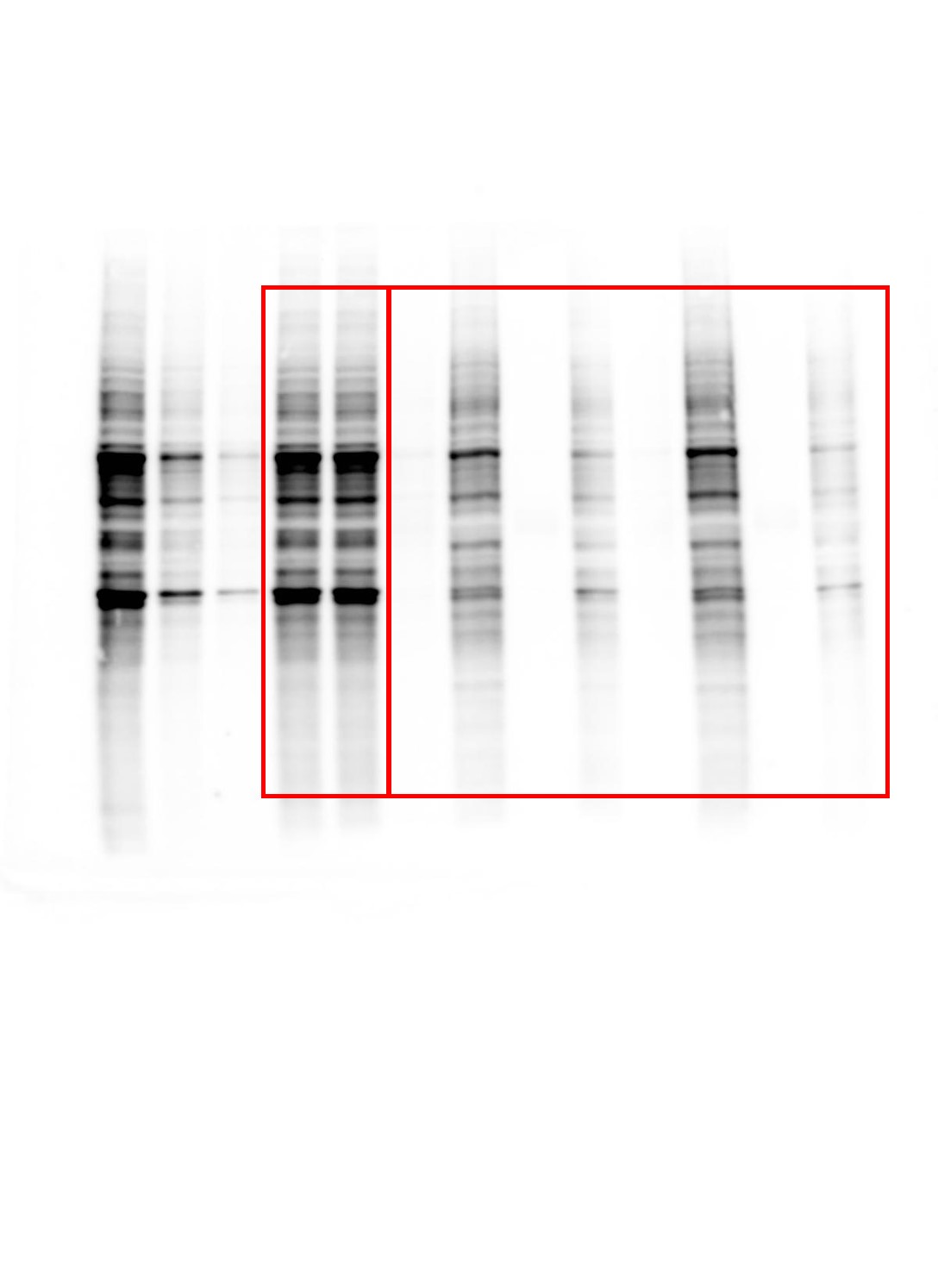

Supplement: Supplementary file 13 — Figure 3 (OLD) [file 41467_2023_42015_MOESM13_ESM.zip › Figure 3/Figure3c/Polypeptidyl-puro.jpg]

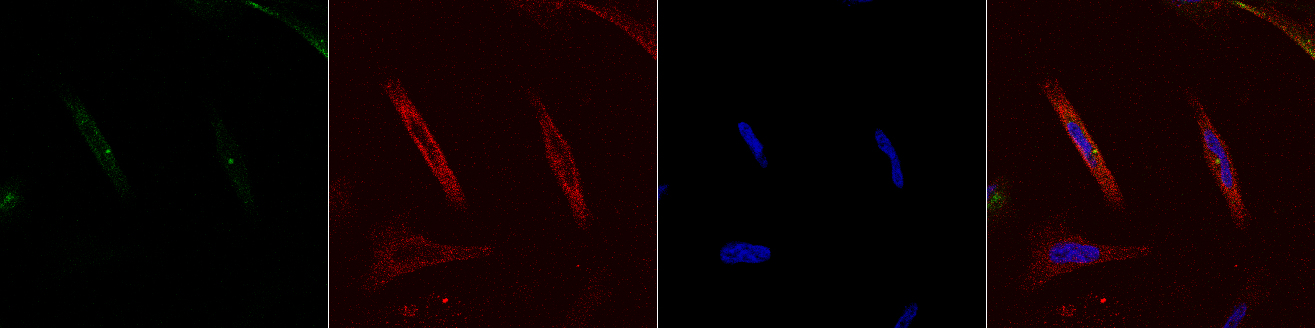

Supplement: Supplementary file 13 — Figure 3 (OLD) [file 41467_2023_42015_MOESM13_ESM.zip › Figure 4/Figure4b/Controlsi_Myc-GST.jpg]

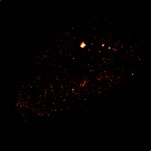

Supplement: Supplementary file 13 — Figure 3 (OLD) [file 41467_2023_42015_MOESM13_ESM.zip › Figure 6/Figure 6a/Super-resolution.jpg]

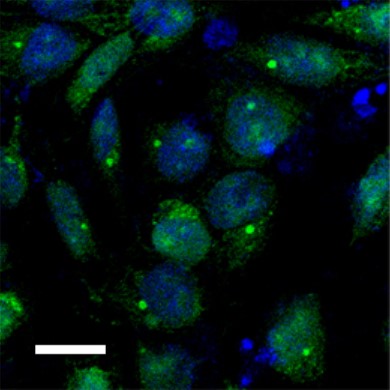

Supplement: Supplementary file 13 — Figure 3 (OLD) [file 41467_2023_42015_MOESM13_ESM.zip › Figure 1/Figure1a/METTL3si_MG_merged.jpg]

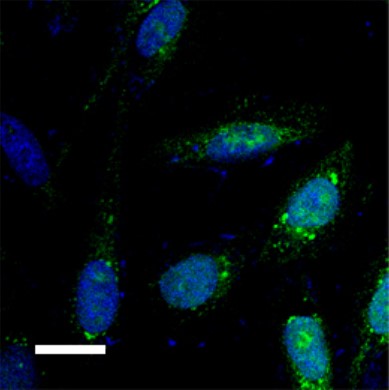

Supplement: Supplementary file 13 — Figure 3 (OLD) [file 41467_2023_42015_MOESM13_ESM.zip › Figure 1/Figure1a/YTHDF2si_MG_merged.jpg]

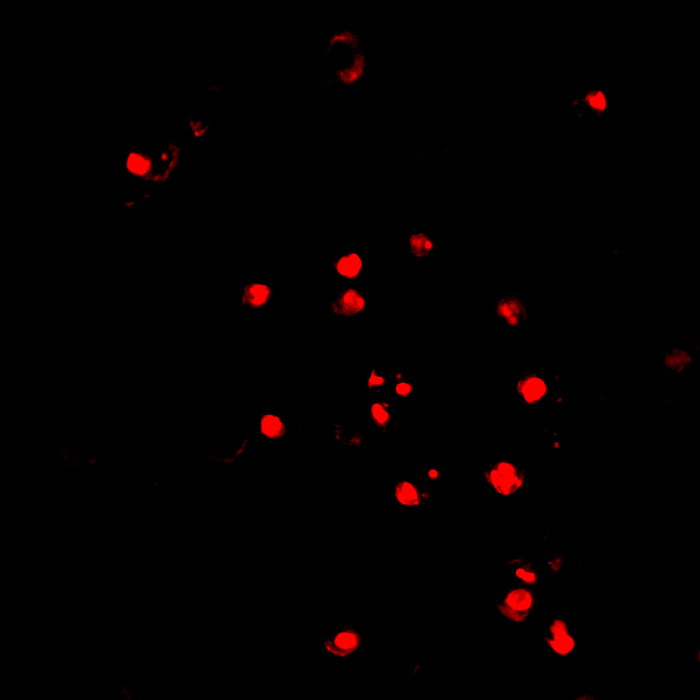

Supplement: Supplementary file 13 — Figure 3 (OLD) [file 41467_2023_42015_MOESM13_ESM.zip › Figure 5/Figure5a/YTHDF2si_Myc_TUNEL.jpg]

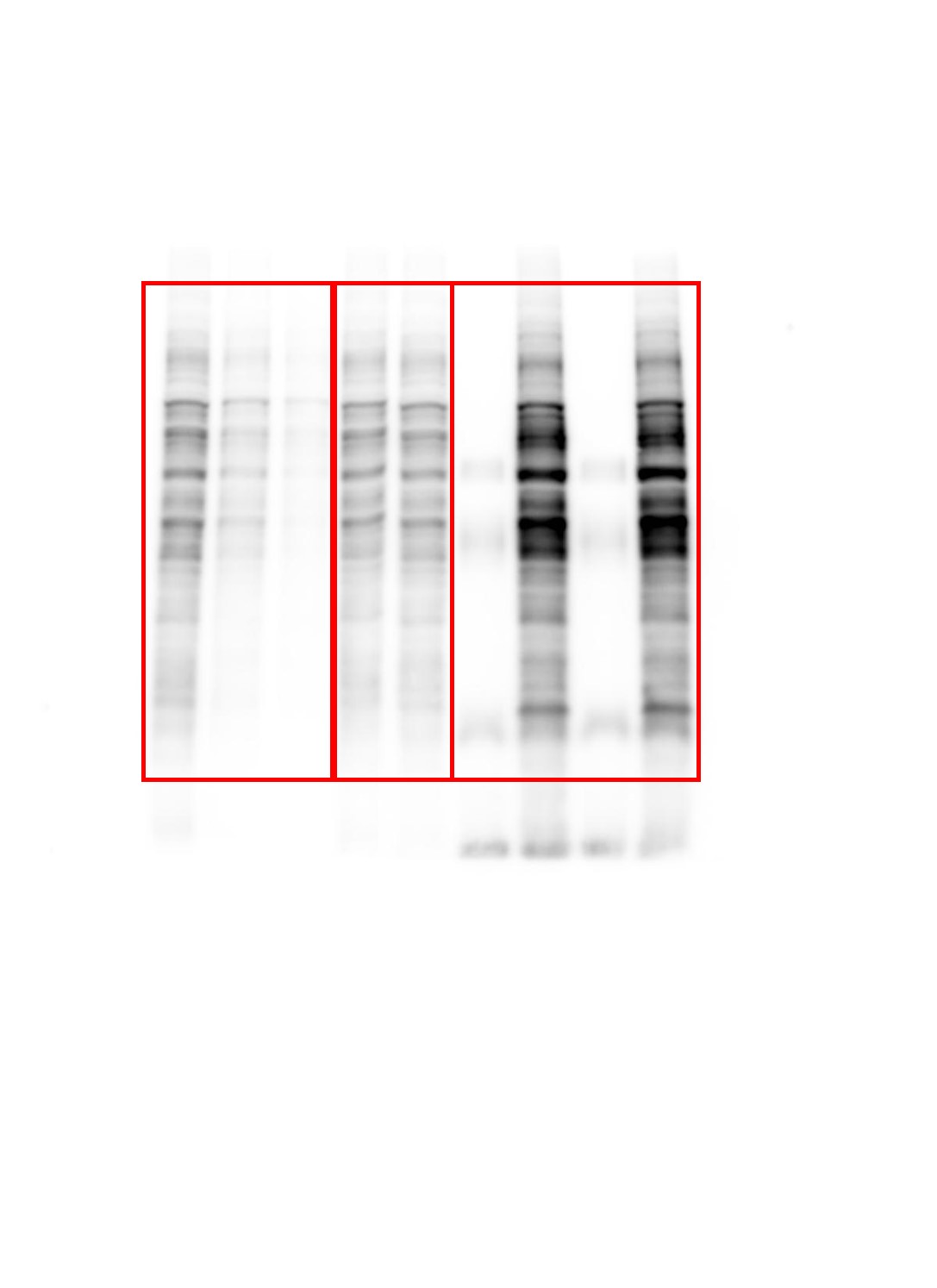

Supplement: Supplementary file 13 — Figure 3 (OLD) [file 41467_2023_42015_MOESM13_ESM.zip › Figure 8/Figure 8a/Polypeptidyl-puro.jpg]

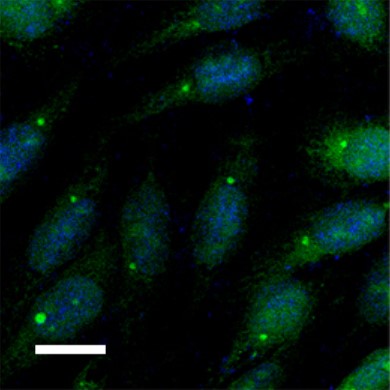

Supplement: Supplementary file 13 — Figure 3 (OLD) [file 41467_2023_42015_MOESM13_ESM.zip › Figure 1/Figure1a/METTL14si_MG_merged.jpg]

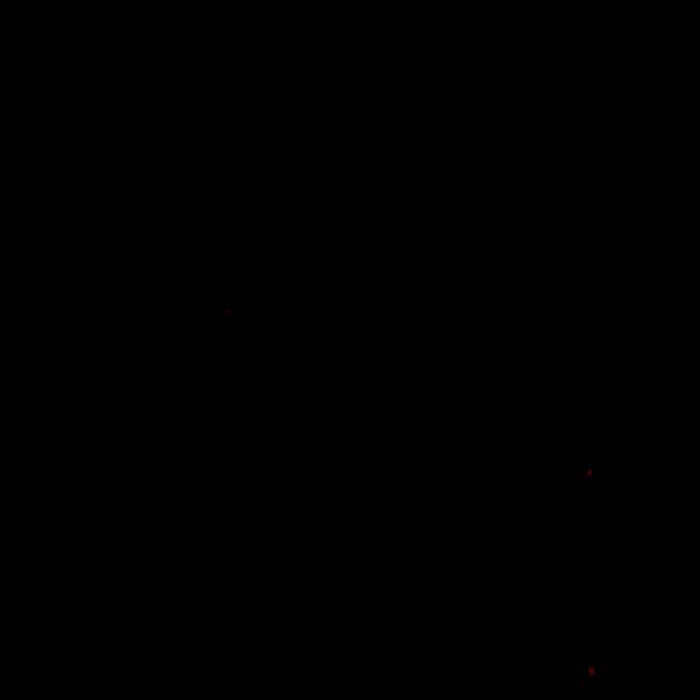

Supplement: Supplementary file 13 — Figure 3 (OLD) [file 41467_2023_42015_MOESM13_ESM.zip › Figure 5/Figure5a/Controlsi-Myc_TUNEL.jpg]

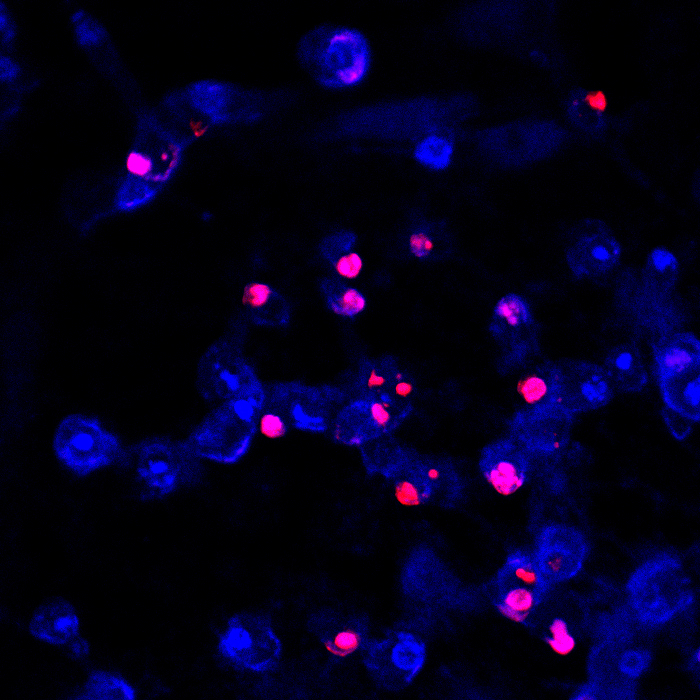

Supplement: Supplementary file 13 — Figure 3 (OLD) [file 41467_2023_42015_MOESM13_ESM.zip › Figure 5/Figure5a/YTHDF2si_Myc_Merged.jpg]

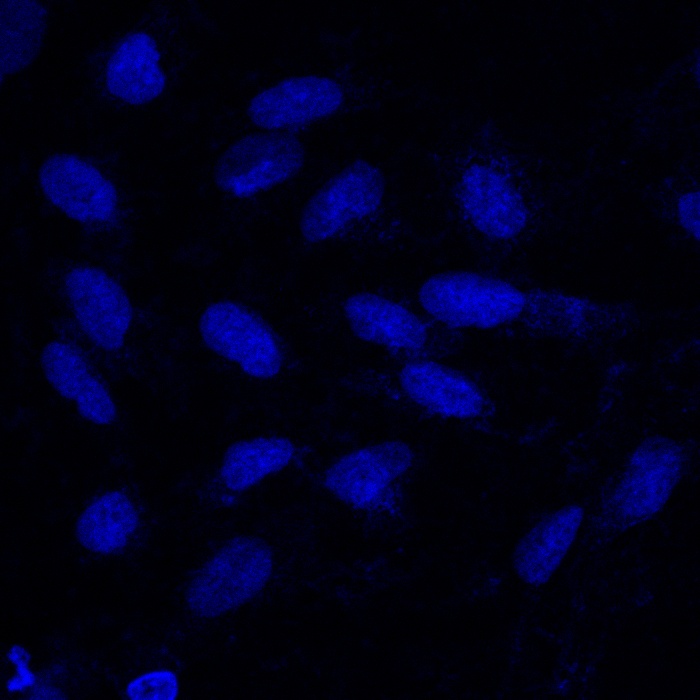

Supplement: Supplementary file 13 — Figure 3 (OLD) [file 41467_2023_42015_MOESM13_ESM.zip › Figure 5/Figure5a/Controlsi-Myc_Merged.jpg]

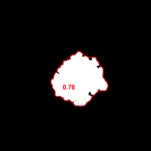

Supplement: Supplementary file 13 — Figure 3 (OLD) [file 41467_2023_42015_MOESM13_ESM.zip › Figure 6/Figure 6a/Measured circularity.jpg]

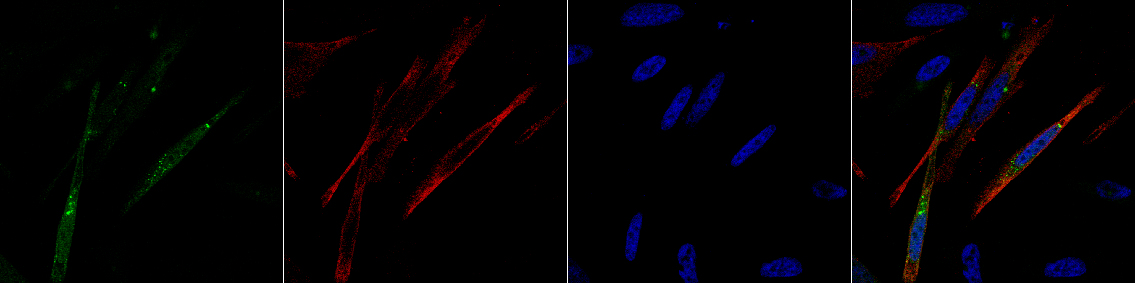

Supplement: Supplementary file 13 — Figure 3 (OLD) [file 41467_2023_42015_MOESM13_ESM.zip › Figure 4/Figure4b/YTHDF2si_101-200-R527A.jpg]

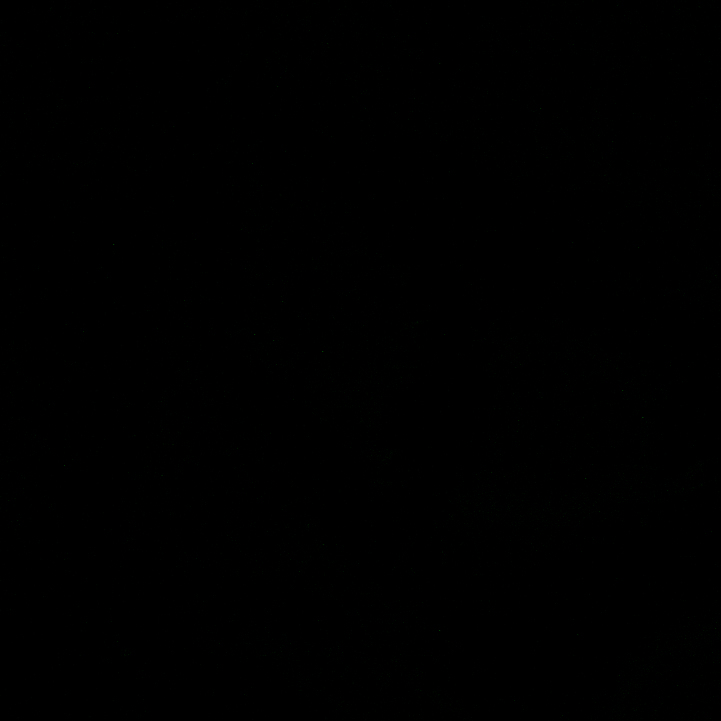

Supplement: Supplementary file 13 — Figure 3 (OLD) [file 41467_2023_42015_MOESM13_ESM.zip › Figure 1/Figure1c/508_FLAG-YTHDF2_DMSO_508.jpg]

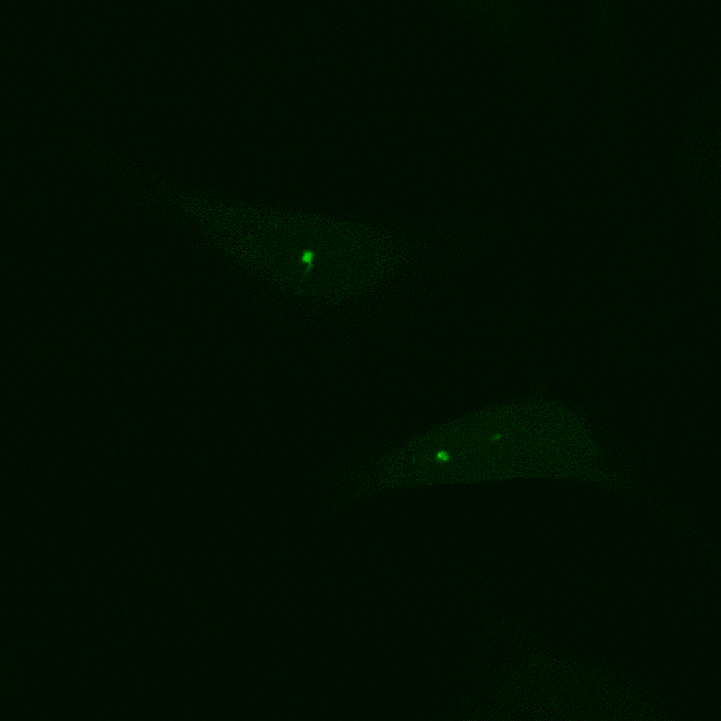

Supplement: Supplementary file 13 — Figure 3 (OLD) [file 41467_2023_42015_MOESM13_ESM.zip › Figure 1/Figure1c/508_FLAG-YTHDF2_MG132_508.jpg]

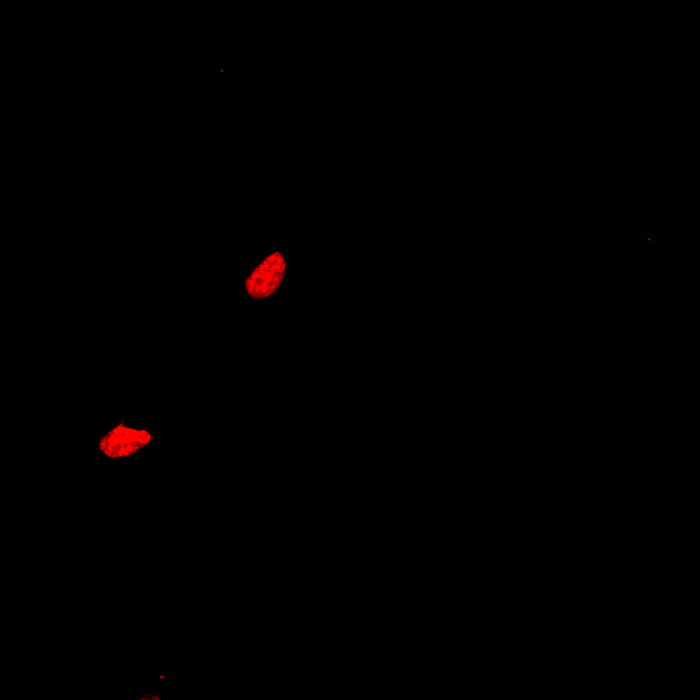

Supplement: Supplementary file 13 — Figure 3 (OLD) [file 41467_2023_42015_MOESM13_ESM.zip › Figure 5/Figure5a/YTHDF2si_Myc-YTHDF2_TUNEL.jpg]

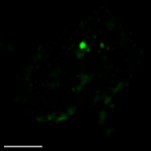

Supplement: Supplementary file 13 — Figure 3 (OLD) [file 41467_2023_42015_MOESM13_ESM.zip › Figure 6/Figure 6a/Conventional florescence.jpg]
