# Supplementary figures and images for: YTHDF2 facilitates aggresome formation via UPF1 in an m6A-independent manner (part 2 of 2)
Source: Nat Commun. 2023 Oct 6;14:6248. doi: 10.1038/s41467-023-42015-w (PMC10558514; doi:10.1038/s41467-023-42015-w)

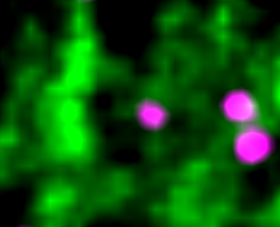

Supplement: Supplementary file 13 — Figure 3 (OLD) [file 41467_2023_42015_MOESM13_ESM.zip › Supplementary Fig. 6/SYN1/Cell_1446_0s_zoom.png]

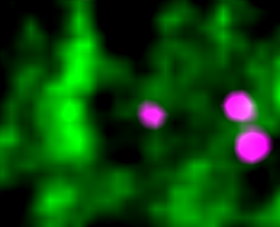

Supplement: Supplementary file 13 — Figure 3 (OLD) [file 41467_2023_42015_MOESM13_ESM.zip › Supplementary Fig. 6/SYN1/Cell_1446_3s_zoom.png]

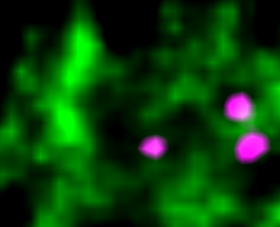

Supplement: Supplementary file 13 — Figure 3 (OLD) [file 41467_2023_42015_MOESM13_ESM.zip › Supplementary Fig. 6/SYN1/Cell_1446_4s_zoom.png]

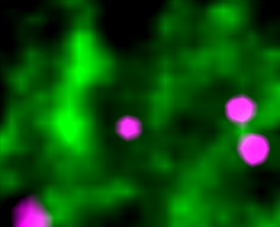

Supplement: Supplementary file 13 — Figure 3 (OLD) [file 41467_2023_42015_MOESM13_ESM.zip › Supplementary Fig. 6/SYN1/Cell_1446_7s_zoom.png]

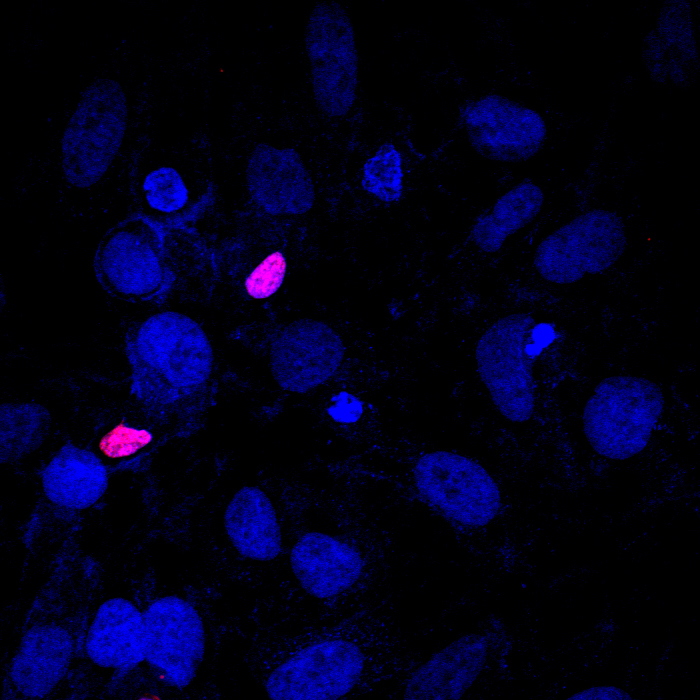

Supplement: Supplementary file 13 — Figure 3 (OLD) [file 41467_2023_42015_MOESM13_ESM.zip › Figure 5/Figure5a/YTHDF2si_Myc-YTHDF2_Merged.jpg]

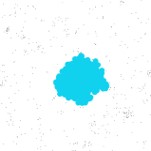

Supplement: Supplementary file 13 — Figure 3 (OLD) [file 41467_2023_42015_MOESM13_ESM.zip › Figure 6/Figure 6a/Detected cluster (DBSCAN).jpg]

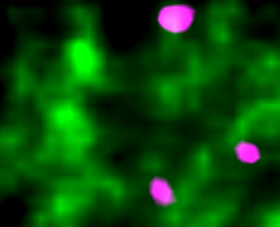

Supplement: Supplementary file 13 — Figure 3 (OLD) [file 41467_2023_42015_MOESM13_ESM.zip › Supplementary Fig. 6/SYN1/Cell_1446_17s_zoom.png]

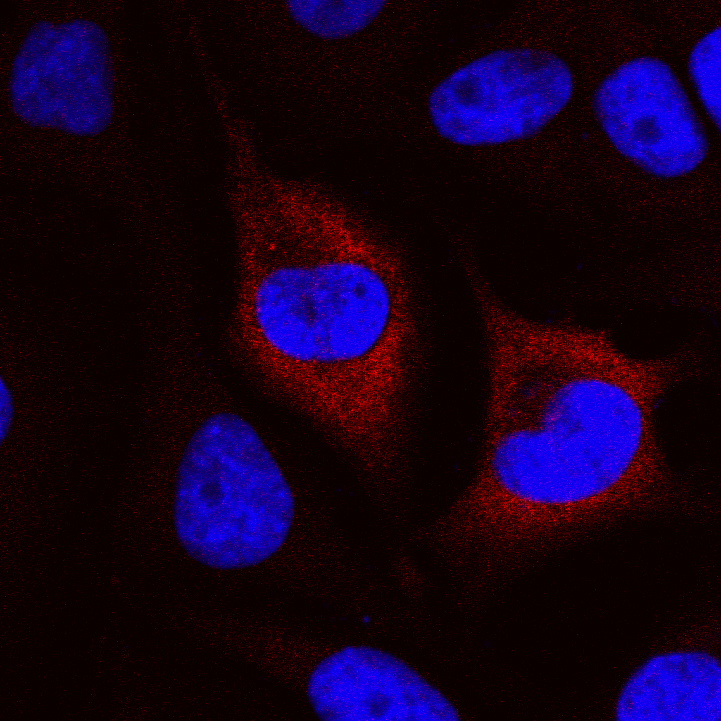

Supplement: Supplementary file 13 — Figure 3 (OLD) [file 41467_2023_42015_MOESM13_ESM.zip › Figure 1/Figure1c/508_FLAG-YTHDF2_DMSO_merged.jpg]

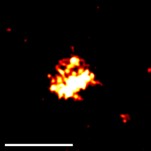

Supplement: Supplementary file 13 — Figure 3 (OLD) [file 41467_2023_42015_MOESM13_ESM.zip › Figure 6/Figure 6a/Super-resolution-aggresome.jpg]

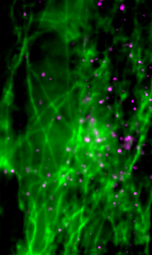

Supplement: Supplementary file 13 — Figure 3 (OLD) [file 41467_2023_42015_MOESM13_ESM.zip › Supplementary Fig. 6/SYN1/Cell_1446_composite.png]

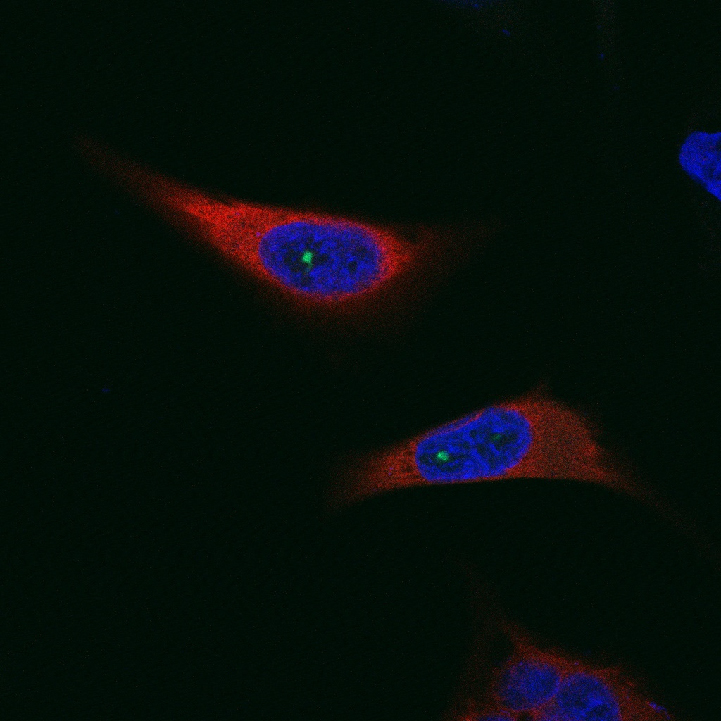

Supplement: Supplementary file 13 — Figure 3 (OLD) [file 41467_2023_42015_MOESM13_ESM.zip › Figure 1/Figure1c/508_FLAG-YTHDF2_MG132_merged.jpg]

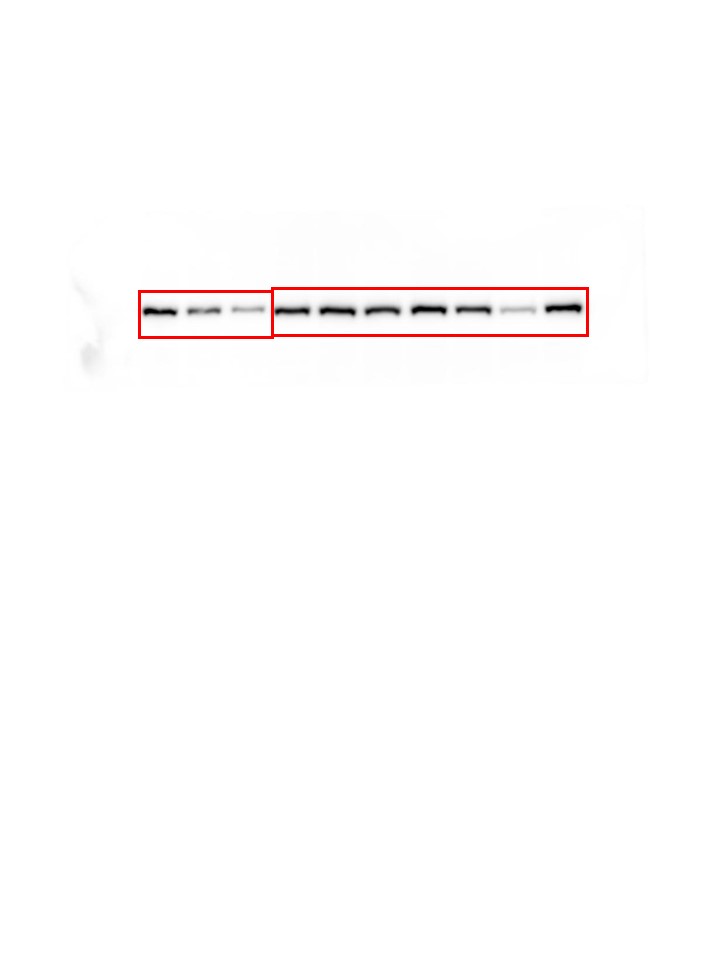

Supplement: Supplementary file 13 — Figure 3 (OLD) [file 41467_2023_42015_MOESM13_ESM.zip › Supplementary Fig. 1/Supplementary Fig. 1d/FTO.jpg]

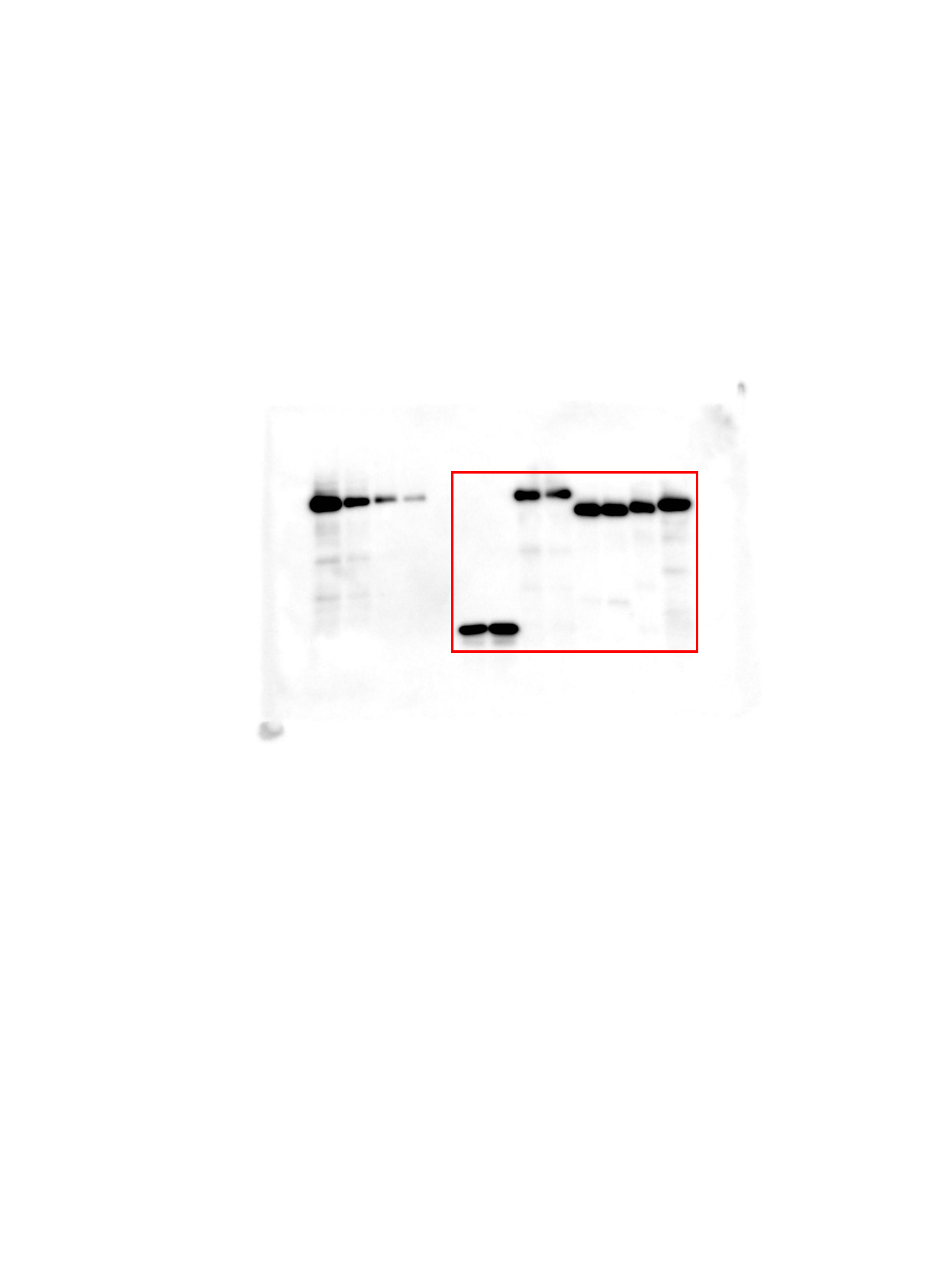

Supplement: Supplementary file 13 — Figure 3 (OLD) [file 41467_2023_42015_MOESM13_ESM.zip › Supplementary Fig. 4/Supplementary Fig. 4b/Myc.png]

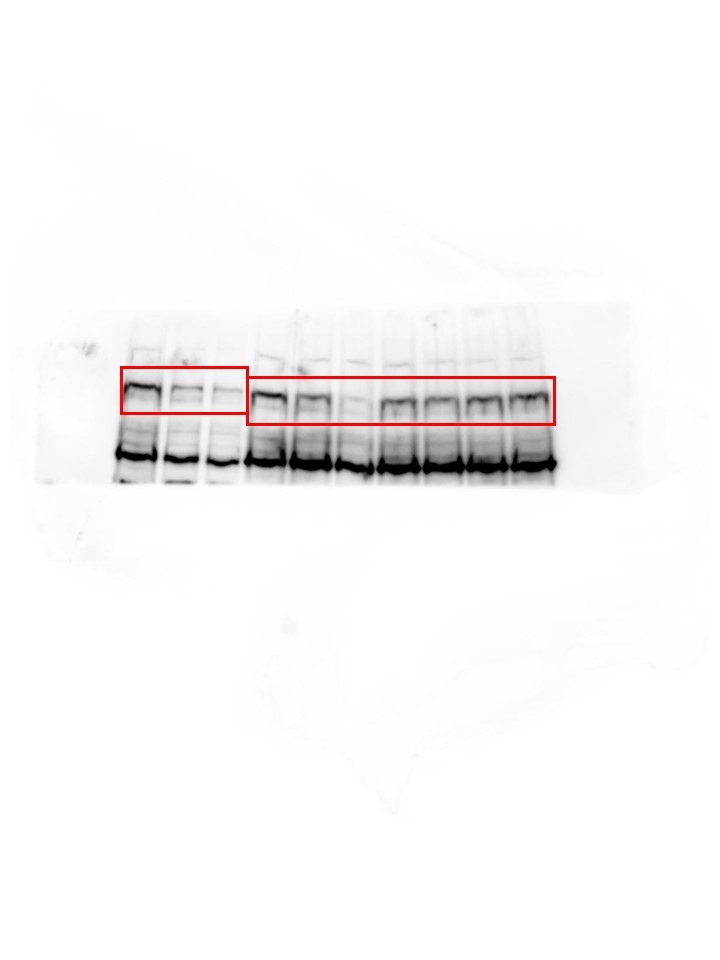

Supplement: Supplementary file 13 — Figure 3 (OLD) [file 41467_2023_42015_MOESM13_ESM.zip › Supplementary Fig. 1/Supplementary Fig. 1d/SMG6.jpg]

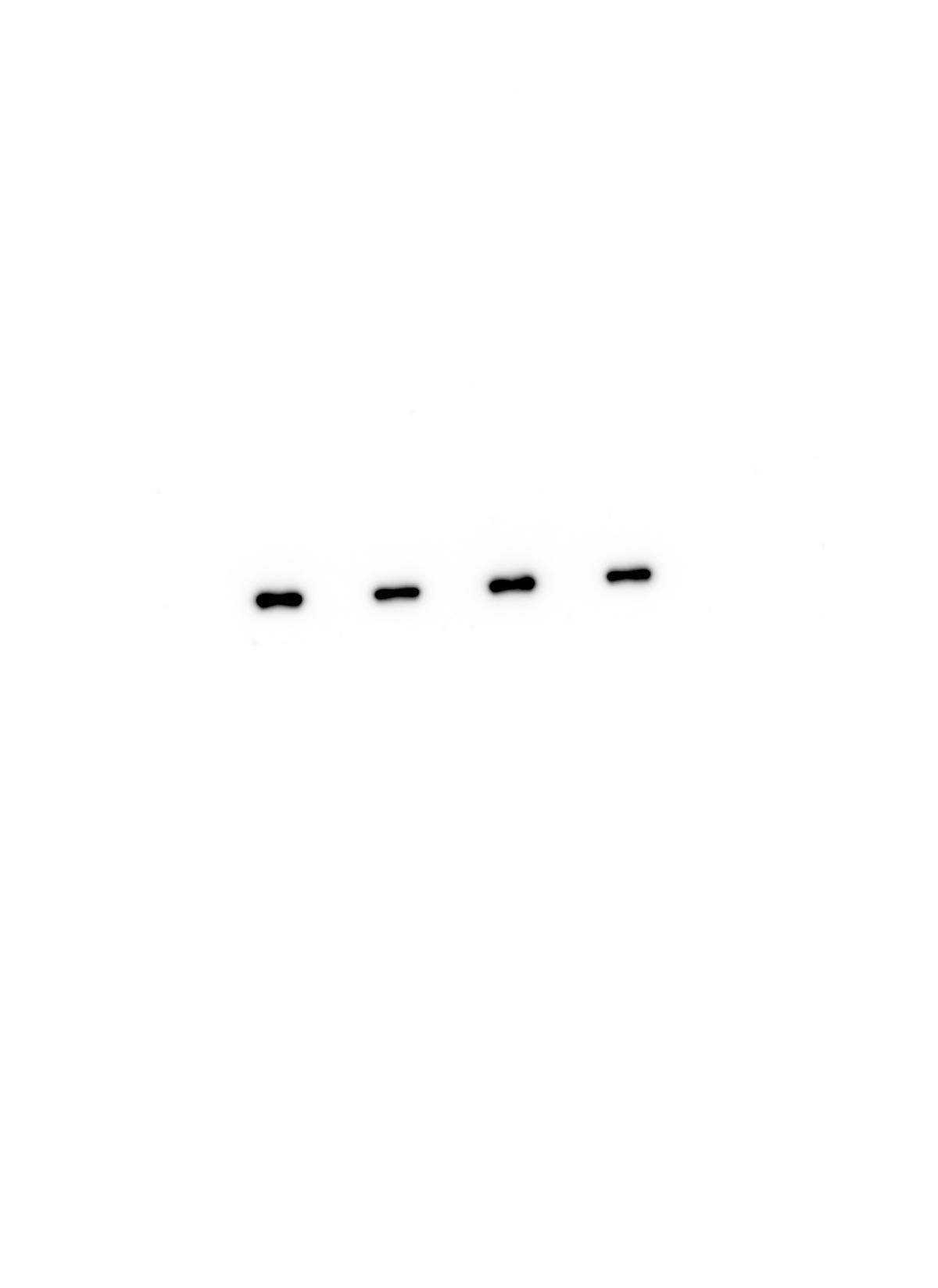

Supplement: Supplementary file 13 — Figure 3 (OLD) [file 41467_2023_42015_MOESM13_ESM.zip › Supplementary Fig. 2/Supplementary Fig. 2c/IMPb.jpg]

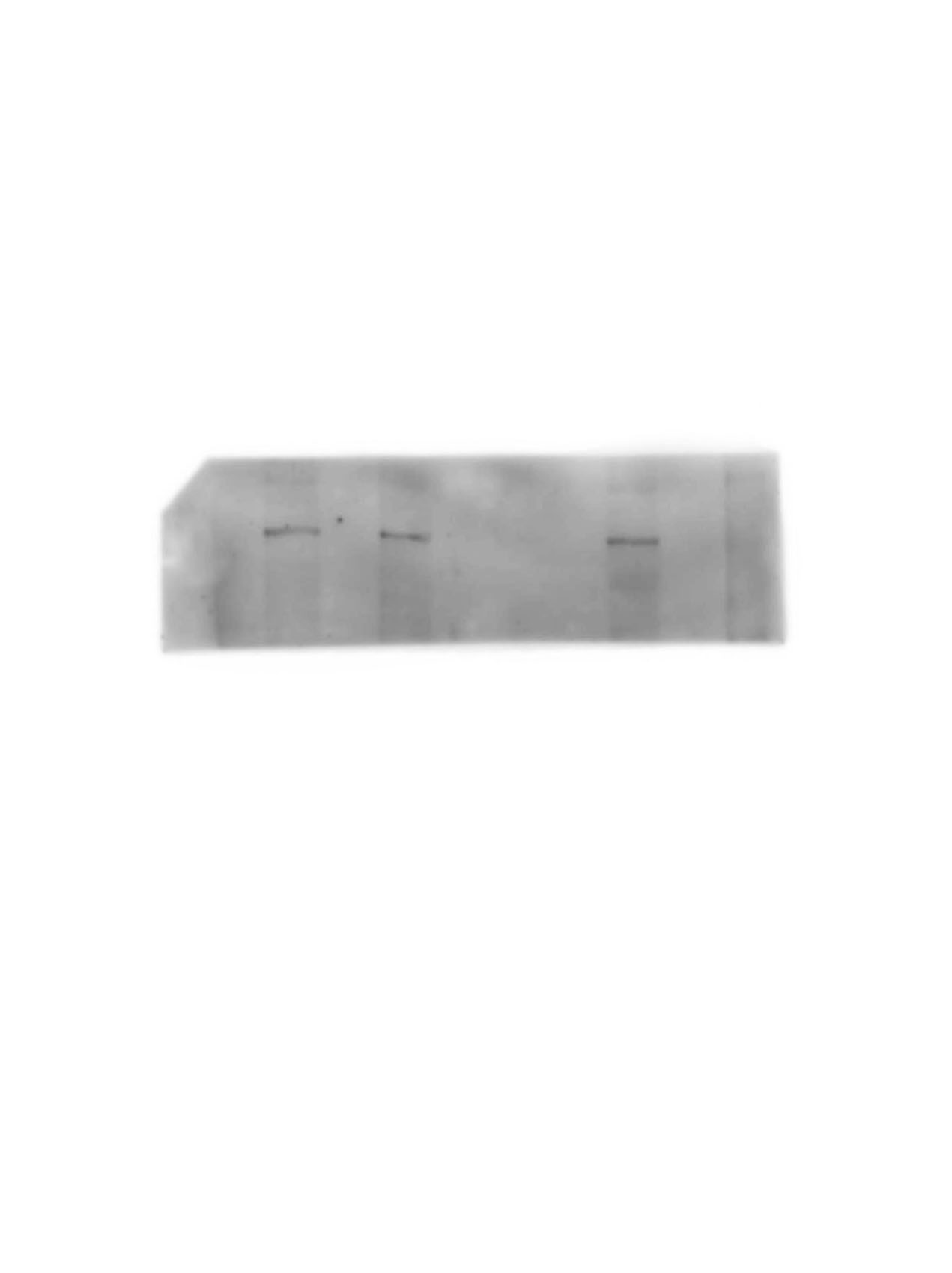

Supplement: Supplementary file 13 — Figure 3 (OLD) [file 41467_2023_42015_MOESM13_ESM.zip › Supplementary Fig. 2/Supplementary Fig. 2c/UPF1.jpg]

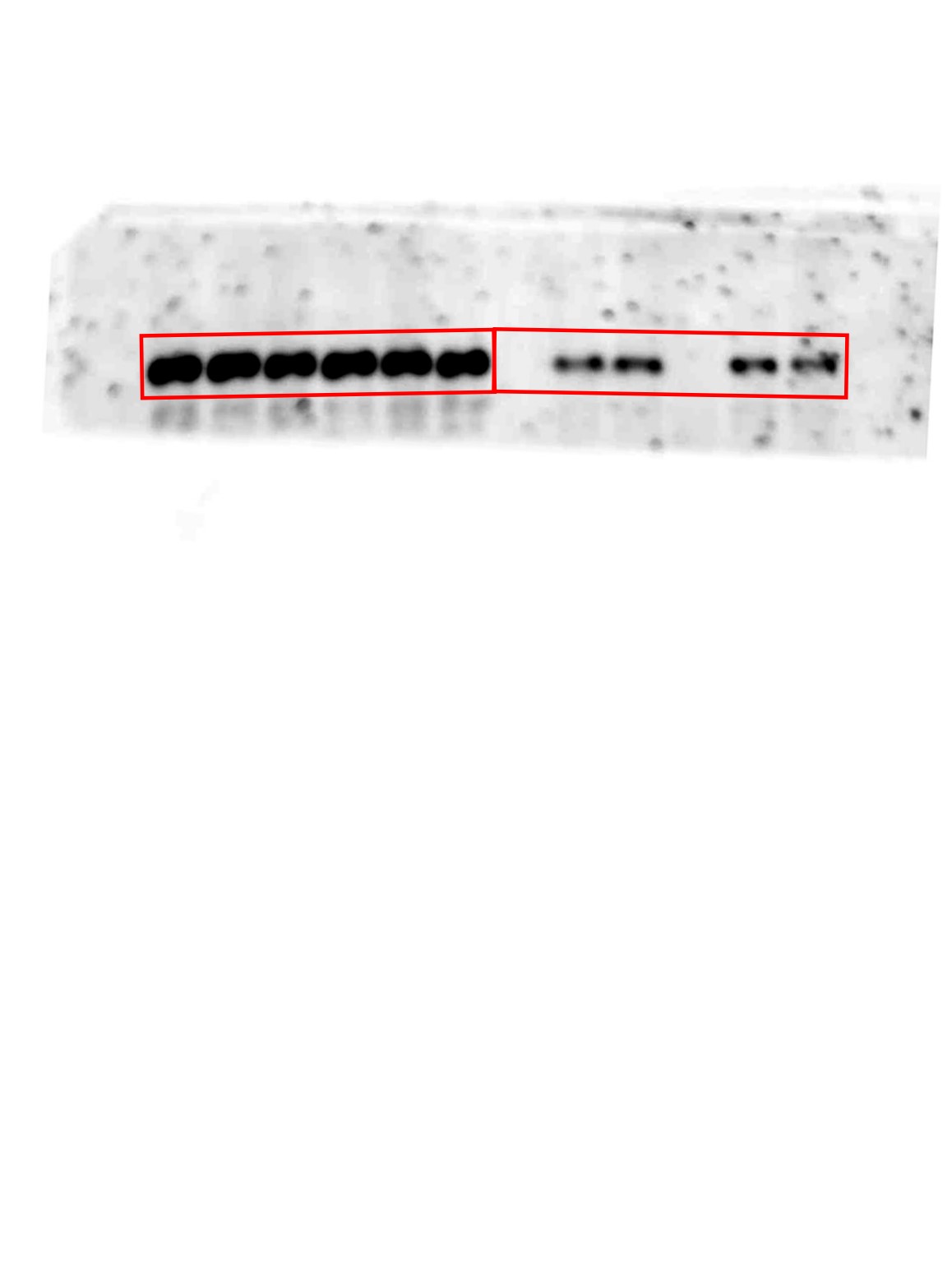

Supplement: Supplementary file 13 — Figure 3 (OLD) [file 41467_2023_42015_MOESM13_ESM.zip › Supplementary Fig. 3/Supplementary Fig. 3a/UPF1.jpg]

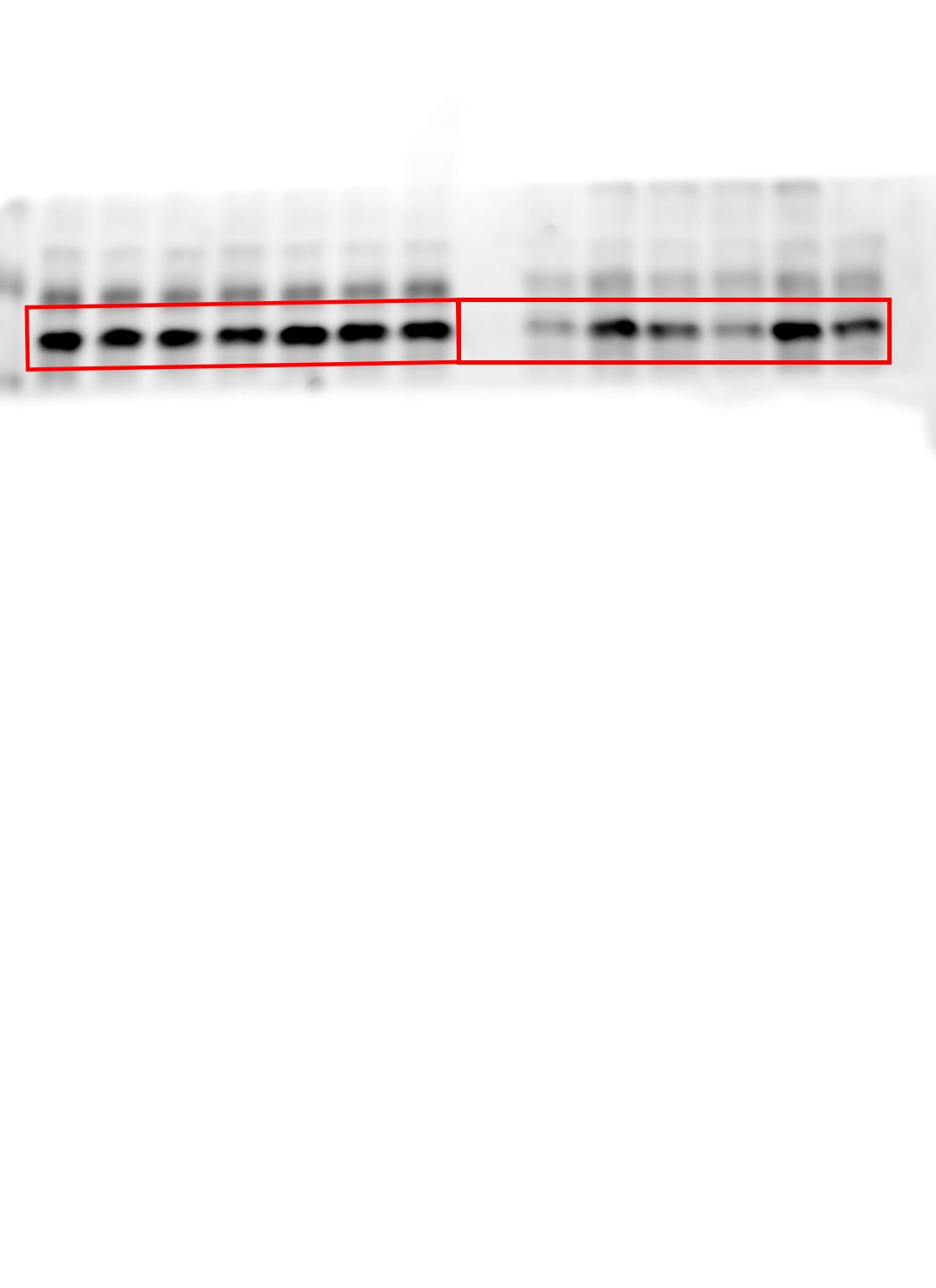

Supplement: Supplementary file 13 — Figure 3 (OLD) [file 41467_2023_42015_MOESM13_ESM.zip › Supplementary Fig. 3/Supplementary Fig. 3b/CTIF.jpg]

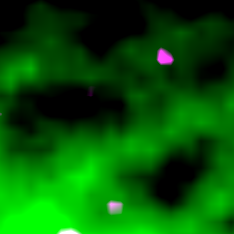

Supplement: Supplementary file 13 — Figure 3 (OLD) [file 41467_2023_42015_MOESM13_ESM.zip › Supplementary Fig. 6/GPx1-Ter/Cell_1476_0s_zoom.png]

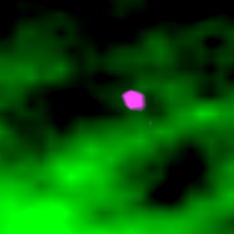

Supplement: Supplementary file 13 — Figure 3 (OLD) [file 41467_2023_42015_MOESM13_ESM.zip › Supplementary Fig. 6/GPx1-Ter/Cell_1476_3s_zoom.png]

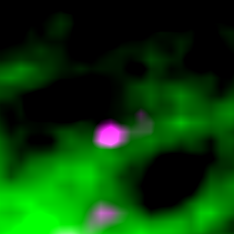

Supplement: Supplementary file 13 — Figure 3 (OLD) [file 41467_2023_42015_MOESM13_ESM.zip › Supplementary Fig. 6/GPx1-Ter/Cell_1476_6s_zoom.png]

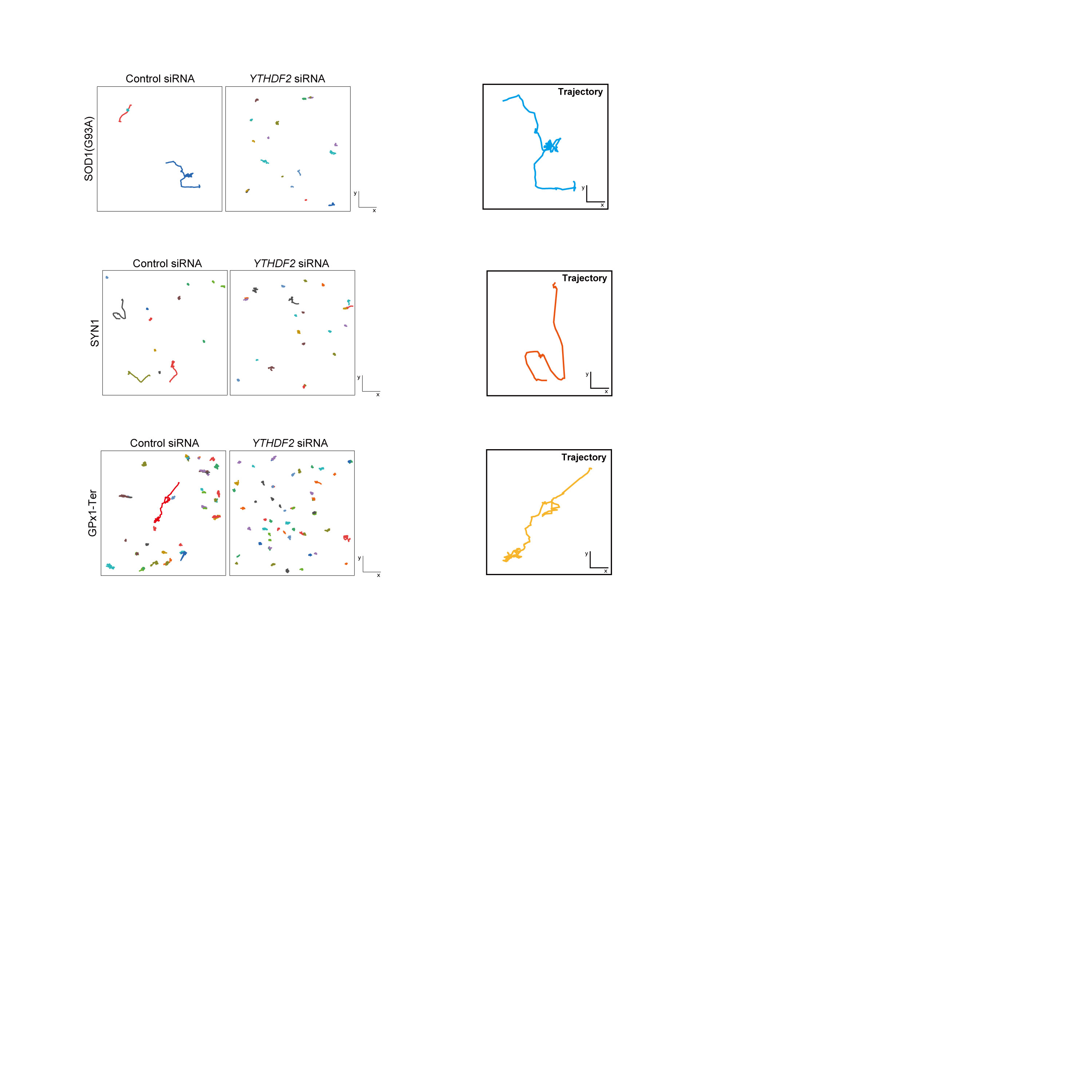

Supplement: Supplementary file 13 — Figure 3 (OLD) [file 41467_2023_42015_MOESM13_ESM.zip › Supplementary Fig. 6/Trajectory-for-processivity.jpg]

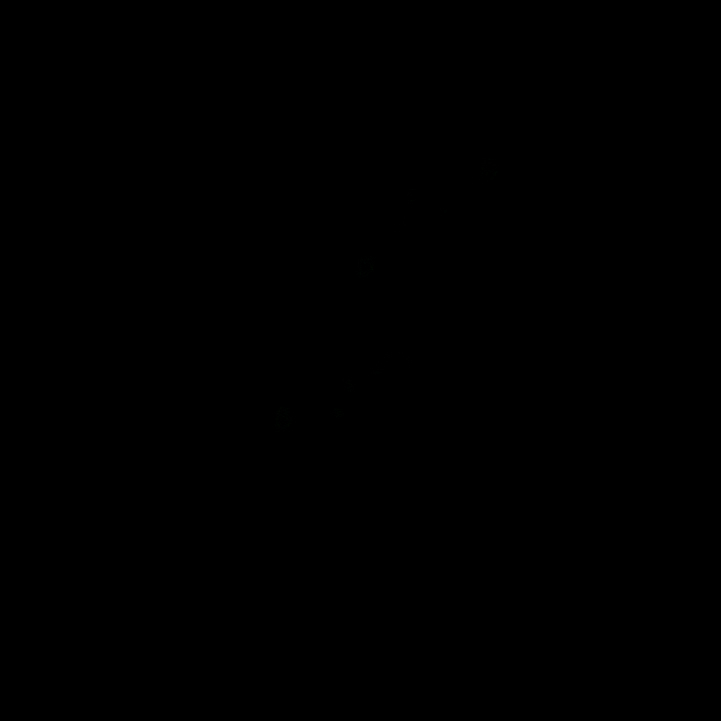

Supplement: Supplementary file 13 — Figure 3 (OLD) [file 41467_2023_42015_MOESM13_ESM.zip › Figure 1/Figure1c/508_FLAG-YTHDF2-Cterm_DMSO_508.jpg]

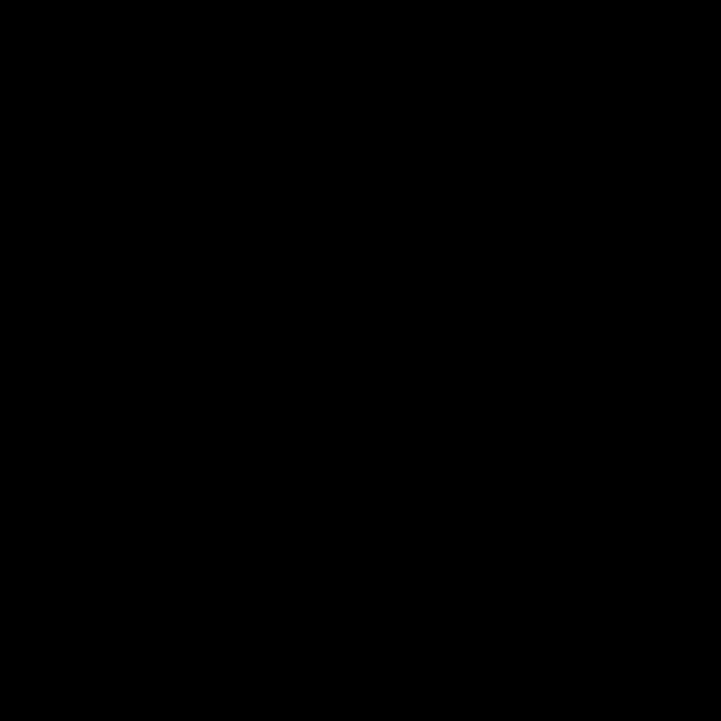

Supplement: Supplementary file 13 — Figure 3 (OLD) [file 41467_2023_42015_MOESM13_ESM.zip › Figure 1/Figure1c/508_FLAG-YTHDF2-Nterm_DMSO_508.jpg]

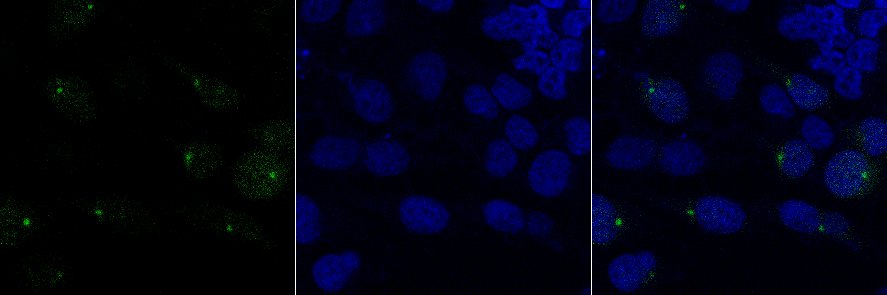

Supplement: Supplementary file 13 — Figure 3 (OLD) [file 41467_2023_42015_MOESM13_ESM.zip › Supplementary Fig. 1/Supplementary Fig. 1b/FTOsi.jpg]

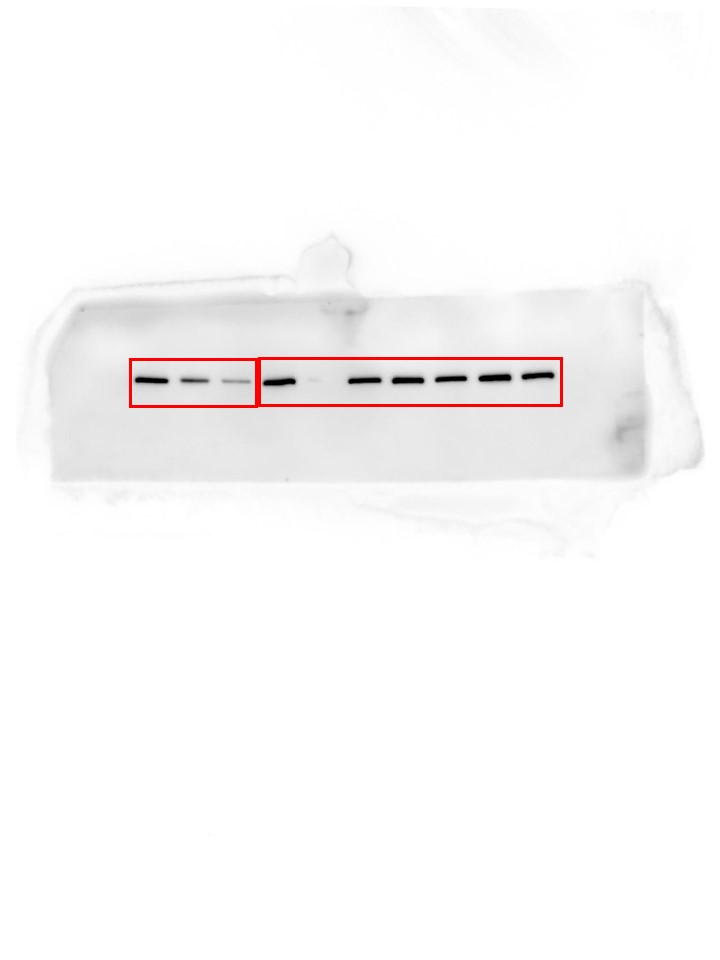

Supplement: Supplementary file 13 — Figure 3 (OLD) [file 41467_2023_42015_MOESM13_ESM.zip › Supplementary Fig. 1/Supplementary Fig. 1d/DCTN1.jpg]

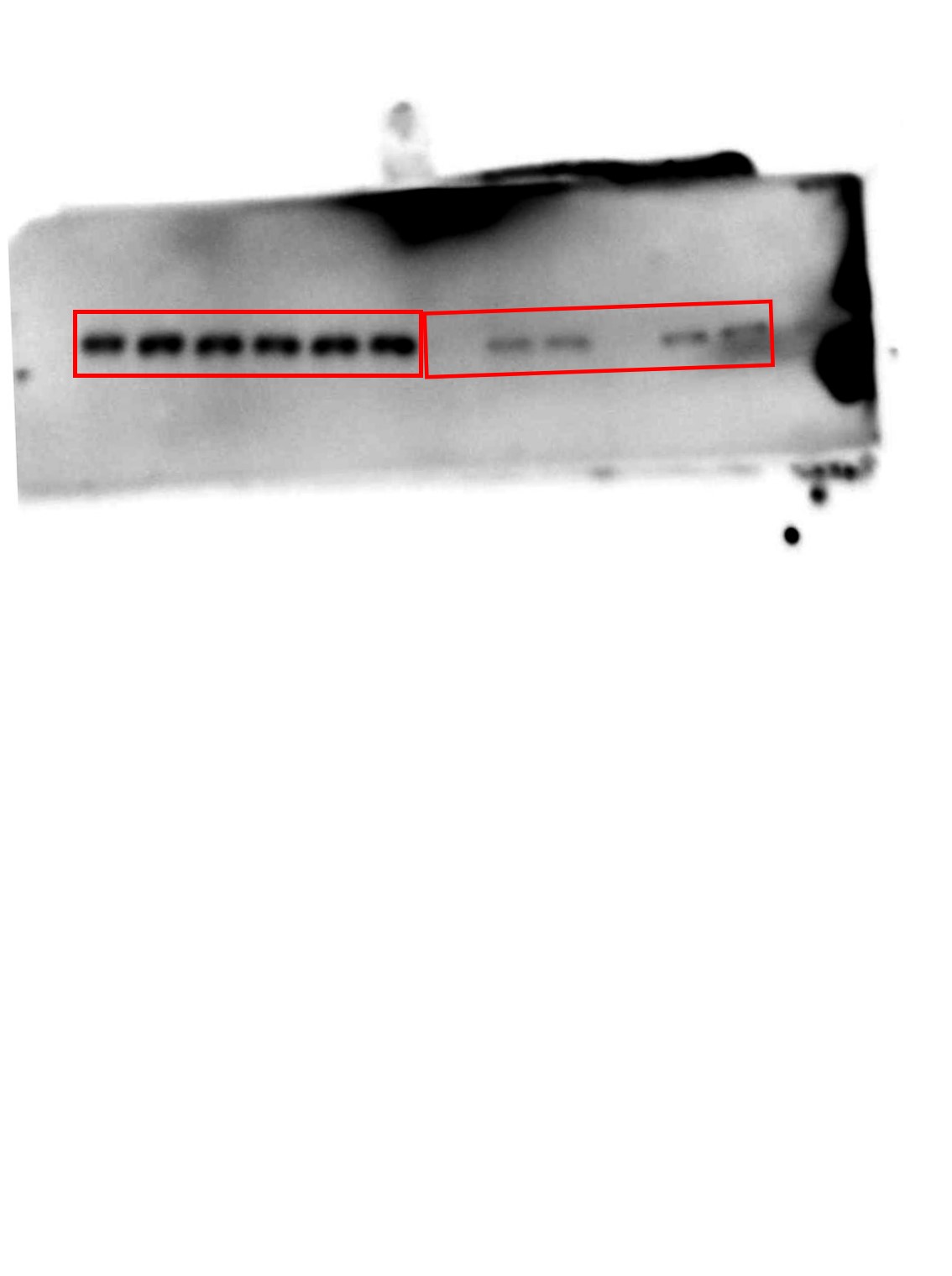

Supplement: Supplementary file 13 — Figure 3 (OLD) [file 41467_2023_42015_MOESM13_ESM.zip › Supplementary Fig. 3/Supplementary Fig. 3a/DCTN1.jpg]

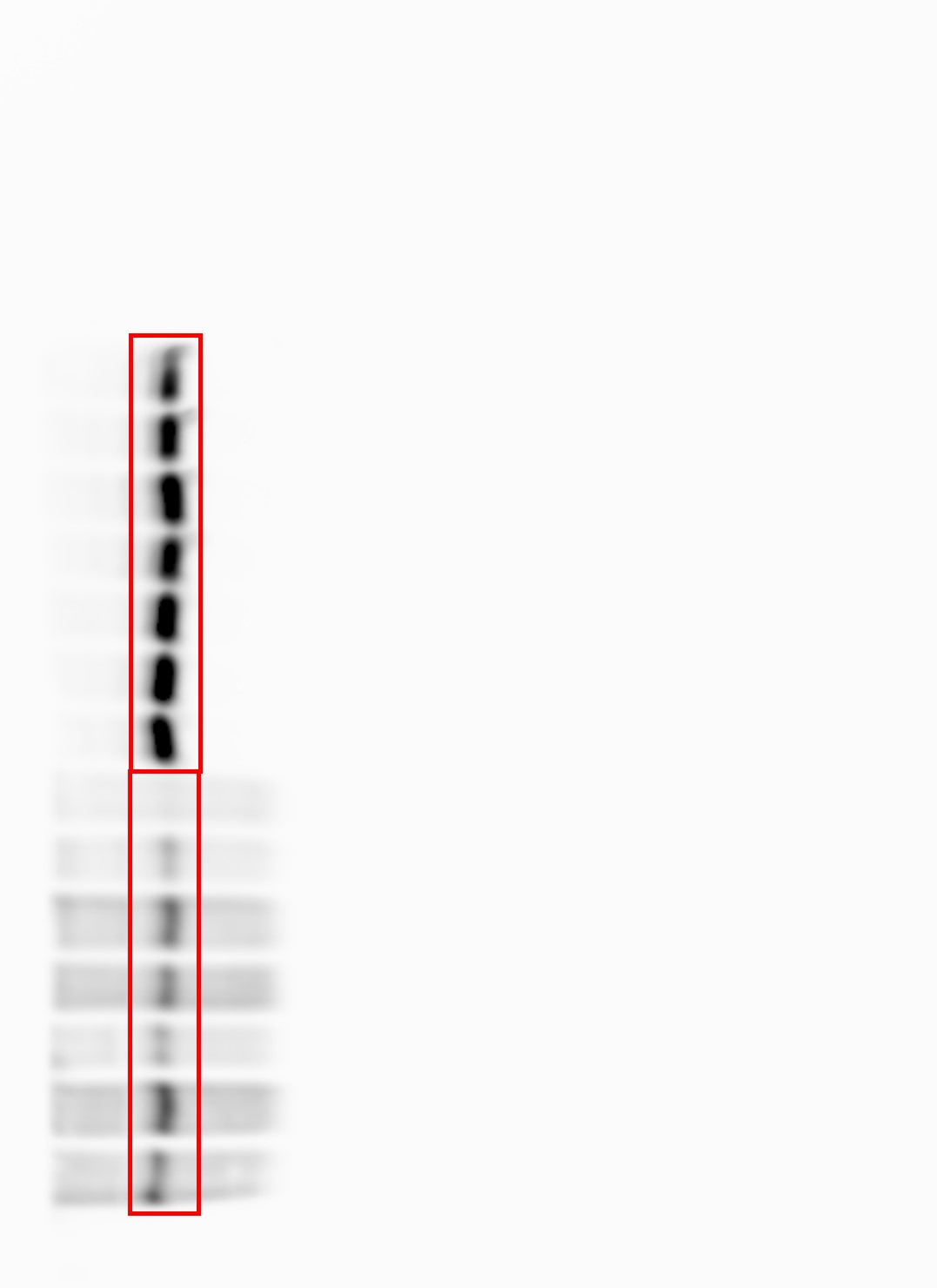

Supplement: Supplementary file 13 — Figure 3 (OLD) [file 41467_2023_42015_MOESM13_ESM.zip › Supplementary Fig. 3/Supplementary Fig. 3b/DCTN1.jpg]

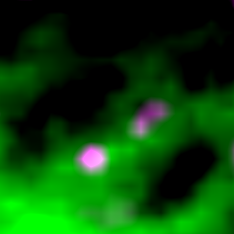

Supplement: Supplementary file 13 — Figure 3 (OLD) [file 41467_2023_42015_MOESM13_ESM.zip › Supplementary Fig. 6/GPx1-Ter/Cell_1476_15s_zoom.png]

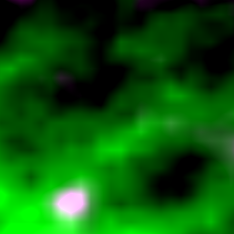

Supplement: Supplementary file 13 — Figure 3 (OLD) [file 41467_2023_42015_MOESM13_ESM.zip › Supplementary Fig. 6/GPx1-Ter/Cell_1476_50s_zoom.png]

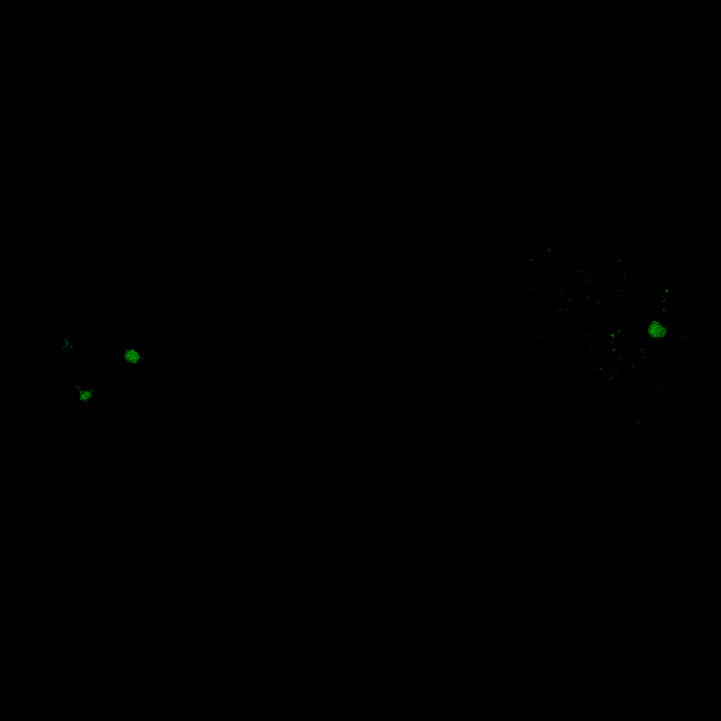

Supplement: Supplementary file 13 — Figure 3 (OLD) [file 41467_2023_42015_MOESM13_ESM.zip › Figure 1/Figure1c/508_FLAG-YTHDF2-Nterm_MG132_508.jpg]

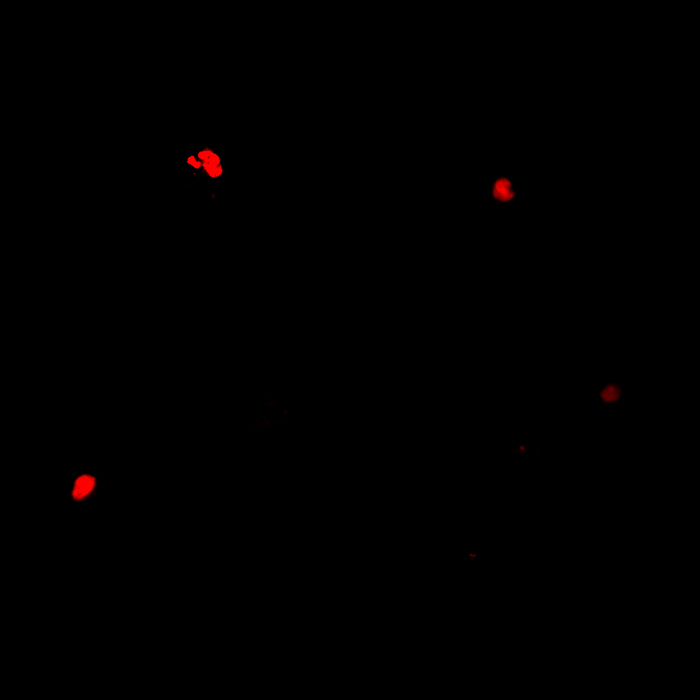

Supplement: Supplementary file 13 — Figure 3 (OLD) [file 41467_2023_42015_MOESM13_ESM.zip › Figure 5/Figure5a/YTHDF2si_Myc-YTHDF2-R527A_TUNEL.jpg]

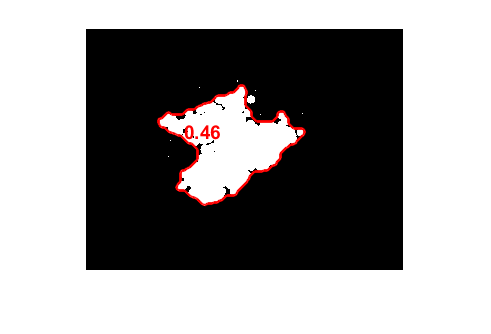

Supplement: Supplementary file 13 — Figure 3 (OLD) [file 41467_2023_42015_MOESM13_ESM.zip › Figure 6/Figure 6b/YTHDF2si_Dendra2-FLAG-GPx1-Ter.png]

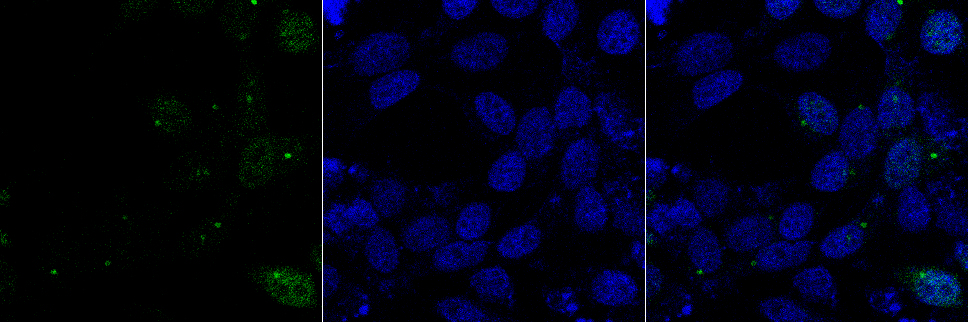

Supplement: Supplementary file 13 — Figure 3 (OLD) [file 41467_2023_42015_MOESM13_ESM.zip › Supplementary Fig. 1/Supplementary Fig. 1b/SMG6si.jpg]

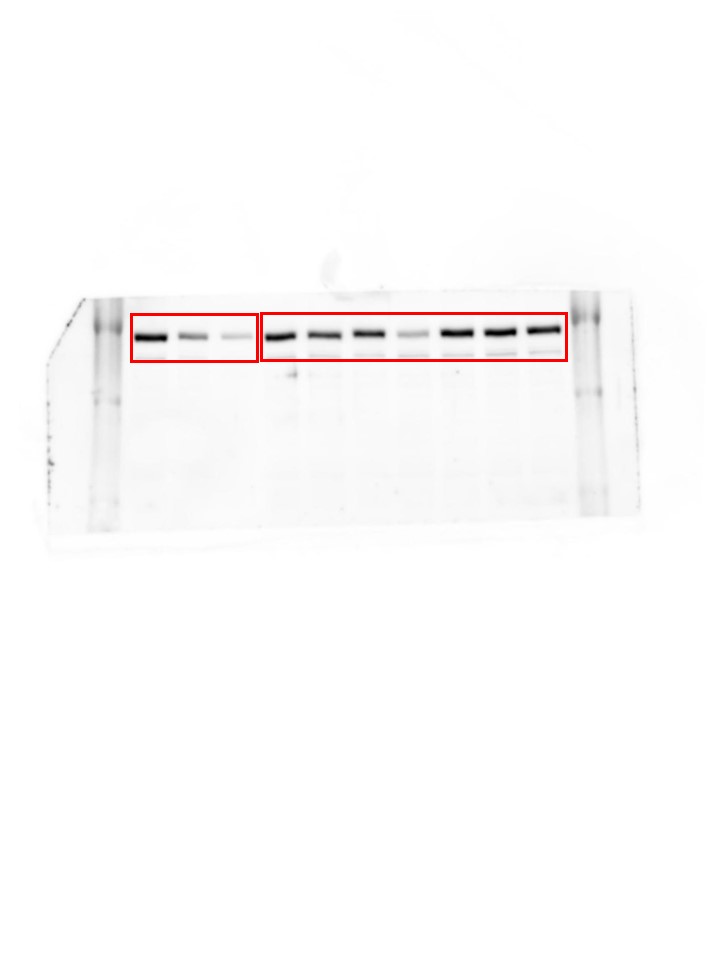

Supplement: Supplementary file 13 — Figure 3 (OLD) [file 41467_2023_42015_MOESM13_ESM.zip › Supplementary Fig. 1/Supplementary Fig. 1d/METTL3.jpg]

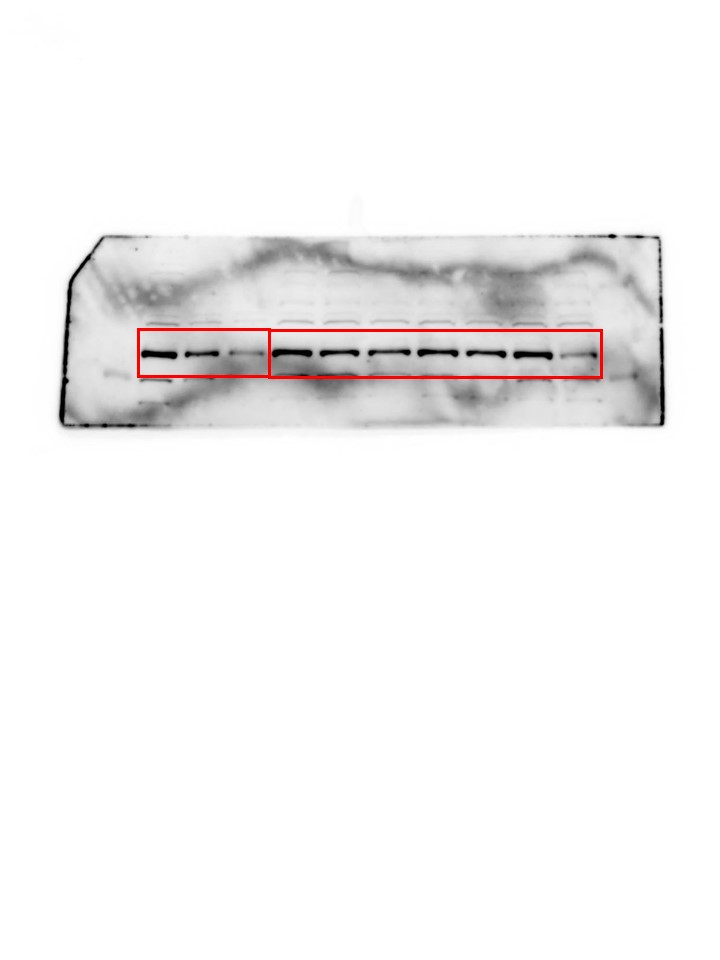

Supplement: Supplementary file 13 — Figure 3 (OLD) [file 41467_2023_42015_MOESM13_ESM.zip › Supplementary Fig. 1/Supplementary Fig. 1d/YTHDF2.jpg]

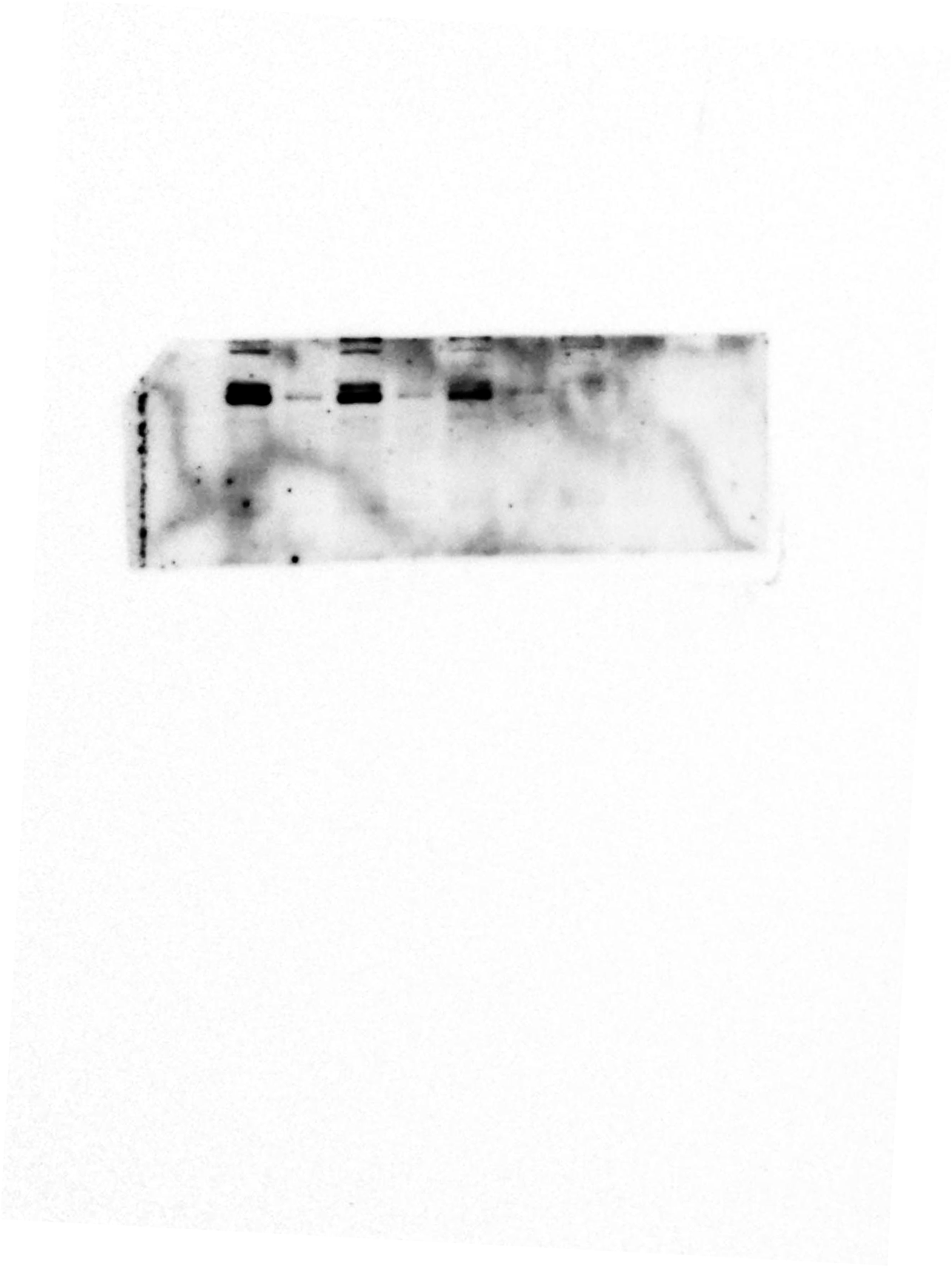

Supplement: Supplementary file 13 — Figure 3 (OLD) [file 41467_2023_42015_MOESM13_ESM.zip › Supplementary Fig. 2/Supplementary Fig. 2c/YTHDF2.tif]

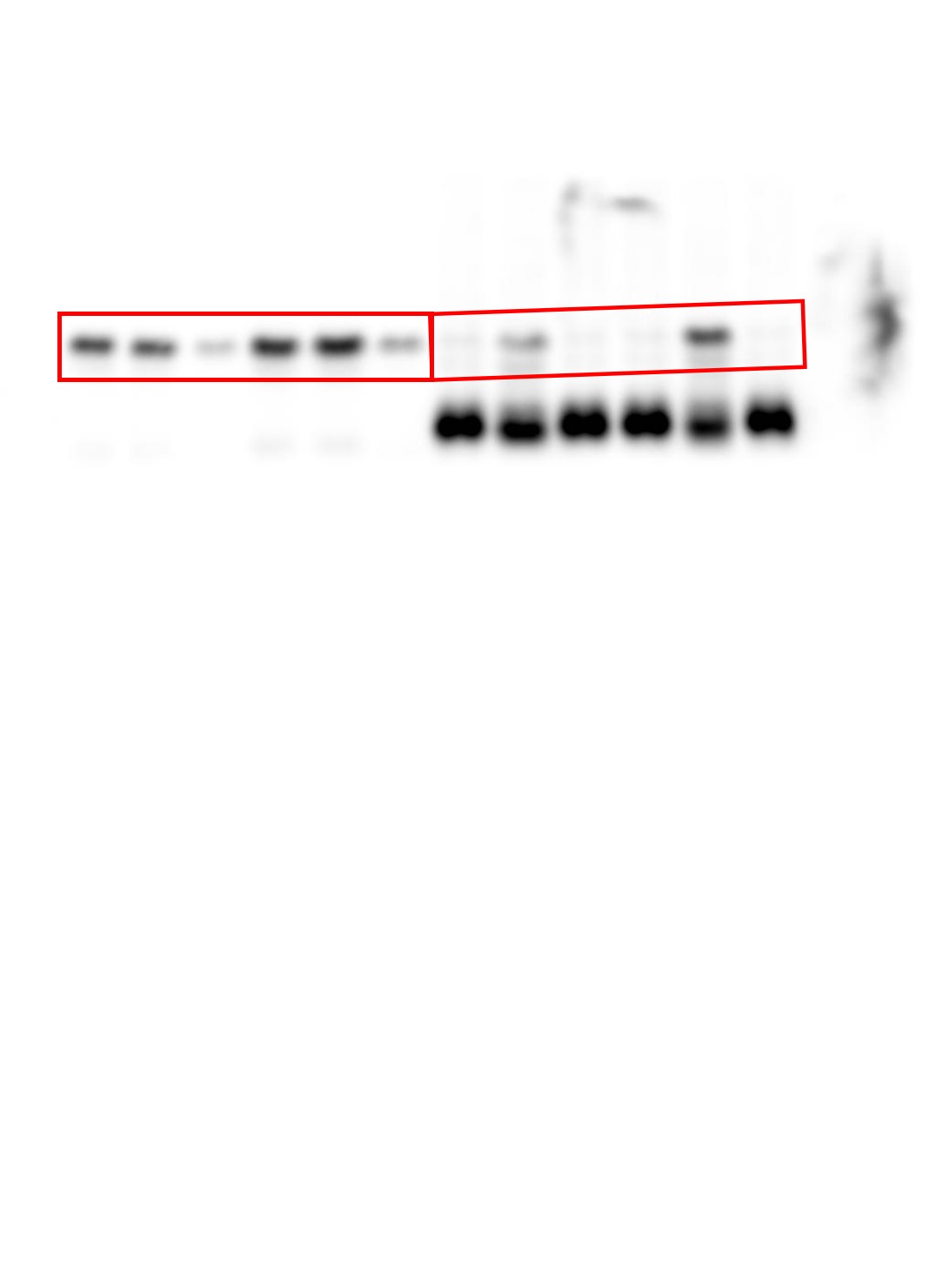

Supplement: Supplementary file 13 — Figure 3 (OLD) [file 41467_2023_42015_MOESM13_ESM.zip › Supplementary Fig. 3/Supplementary Fig. 3a/YTHDF2.jpg]

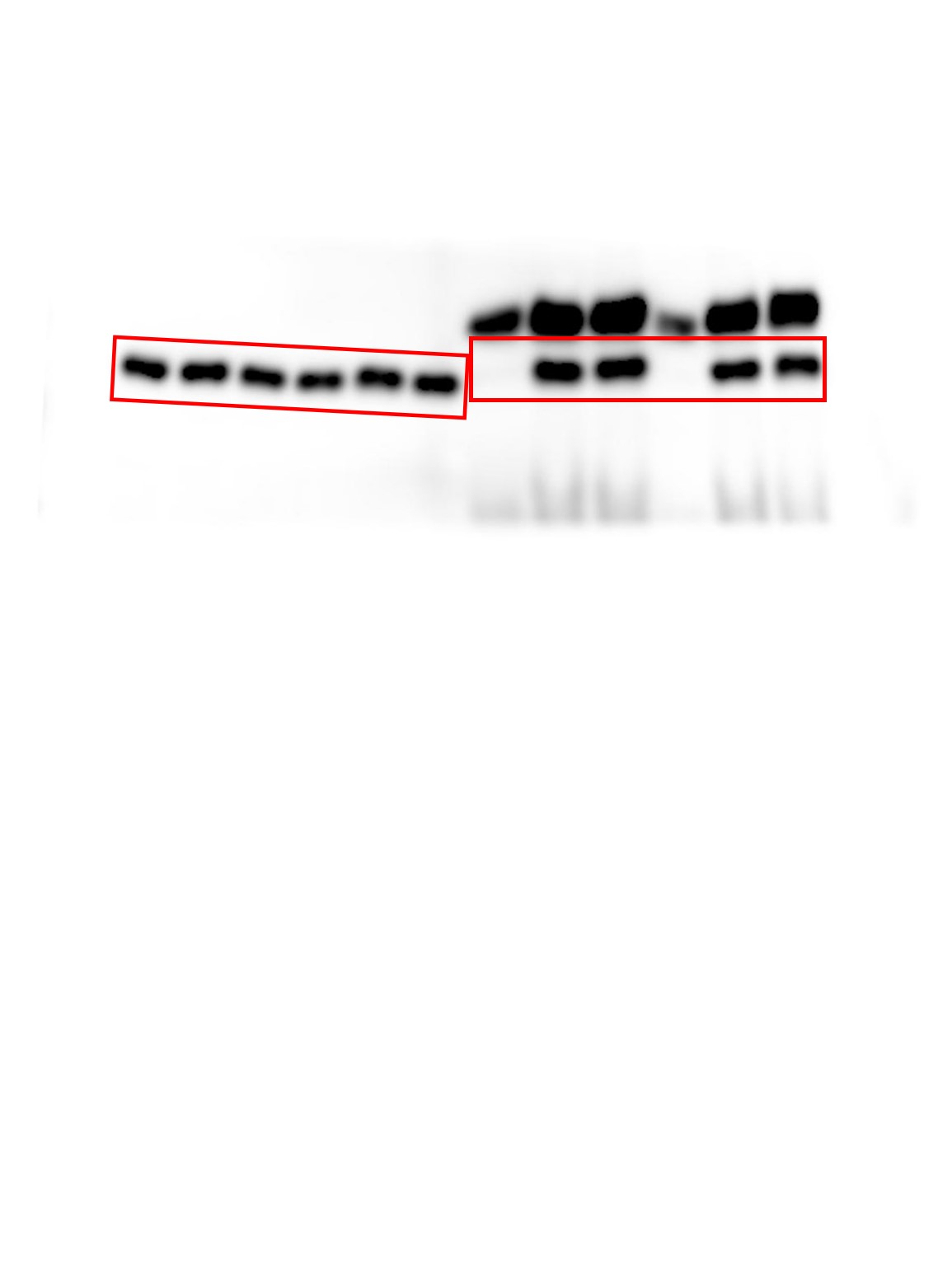

Supplement: Supplementary file 13 — Figure 3 (OLD) [file 41467_2023_42015_MOESM13_ESM.zip › Supplementary Fig. 3/Supplementary Fig. 3a/eEF1A1.jpg]

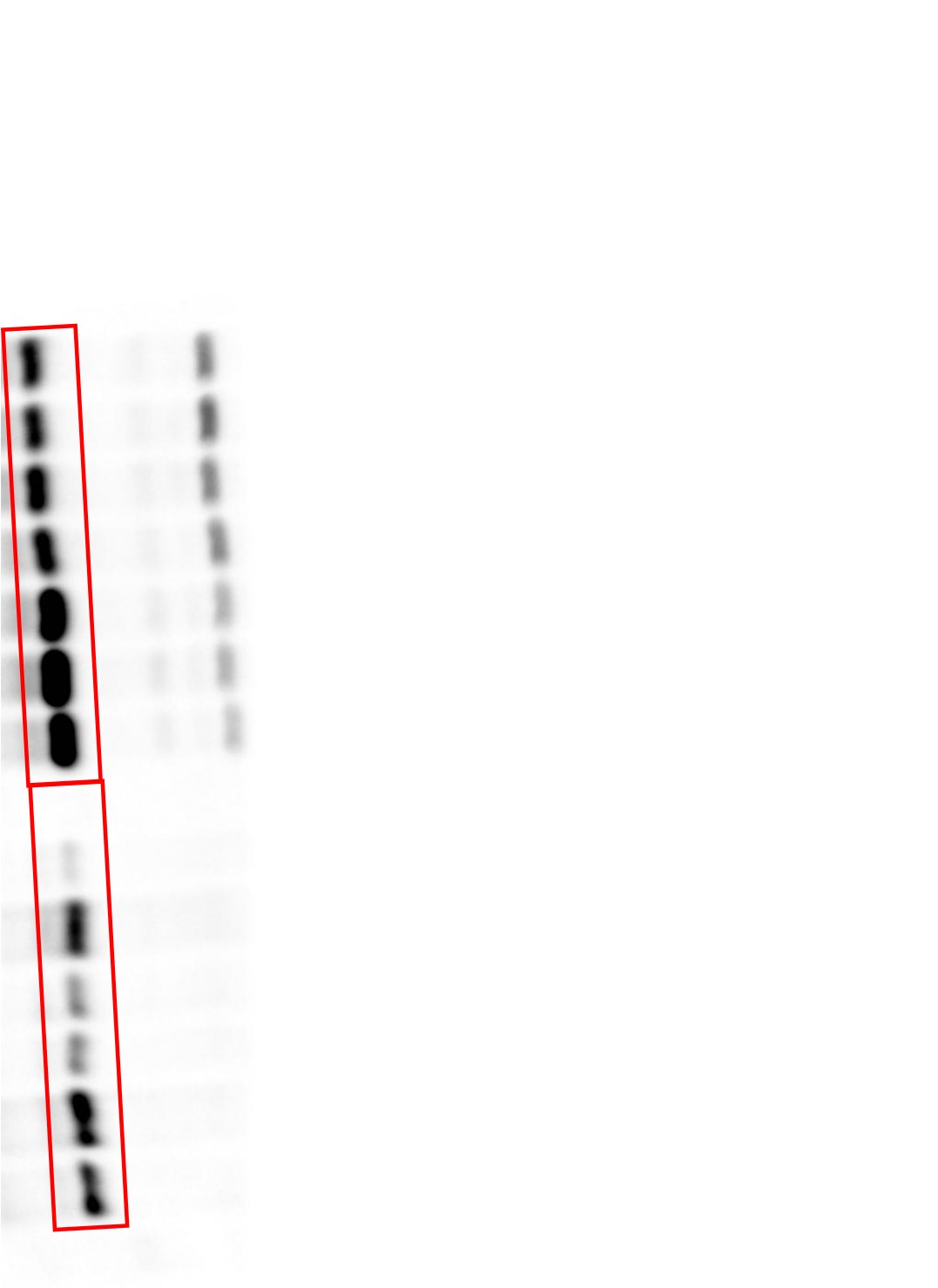

Supplement: Supplementary file 13 — Figure 3 (OLD) [file 41467_2023_42015_MOESM13_ESM.zip › Supplementary Fig. 3/Supplementary Fig. 3b/YTHDF2.jpg]

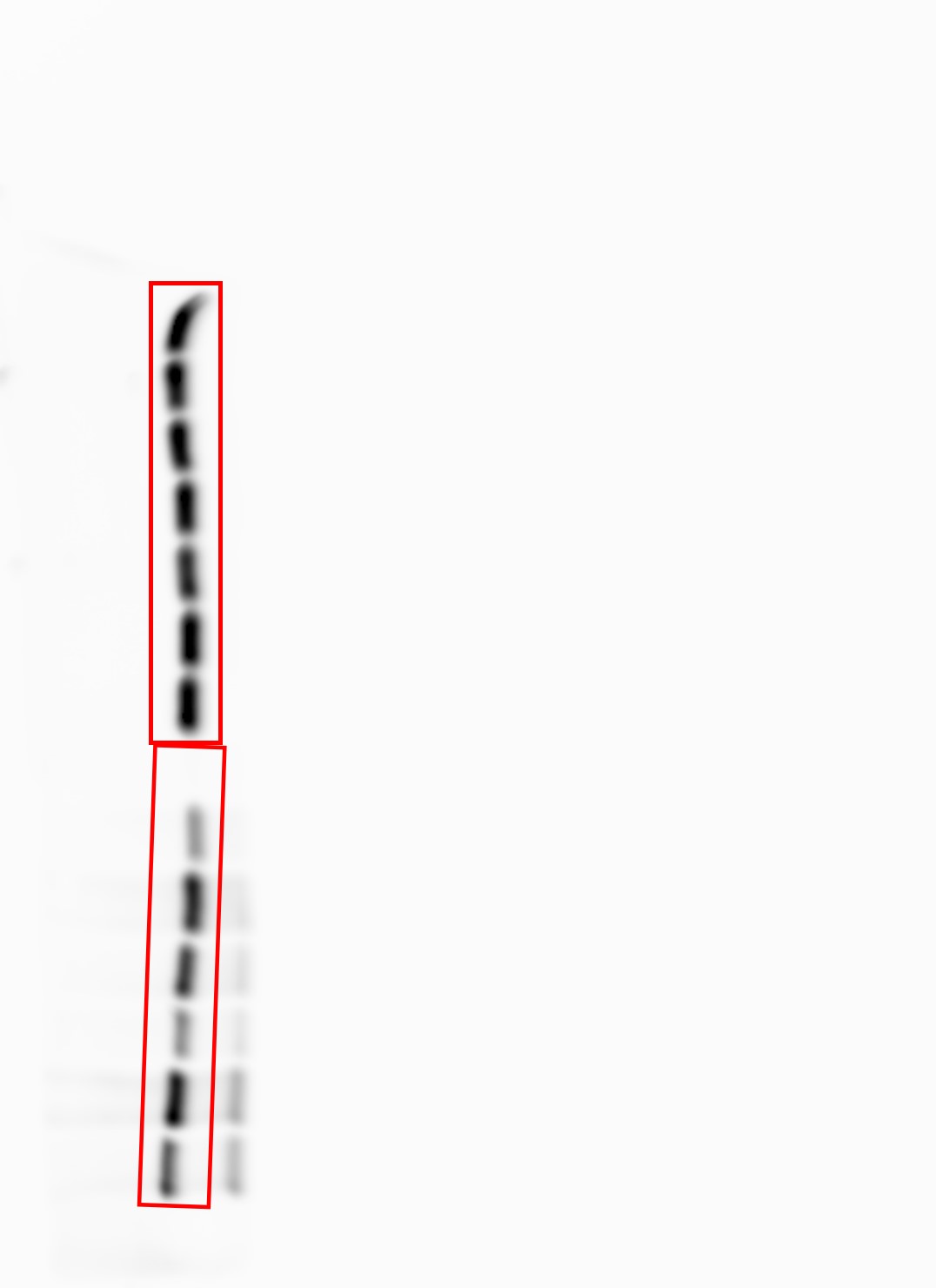

Supplement: Supplementary file 13 — Figure 3 (OLD) [file 41467_2023_42015_MOESM13_ESM.zip › Supplementary Fig. 3/Supplementary Fig. 3b/eEF1A1.jpg]

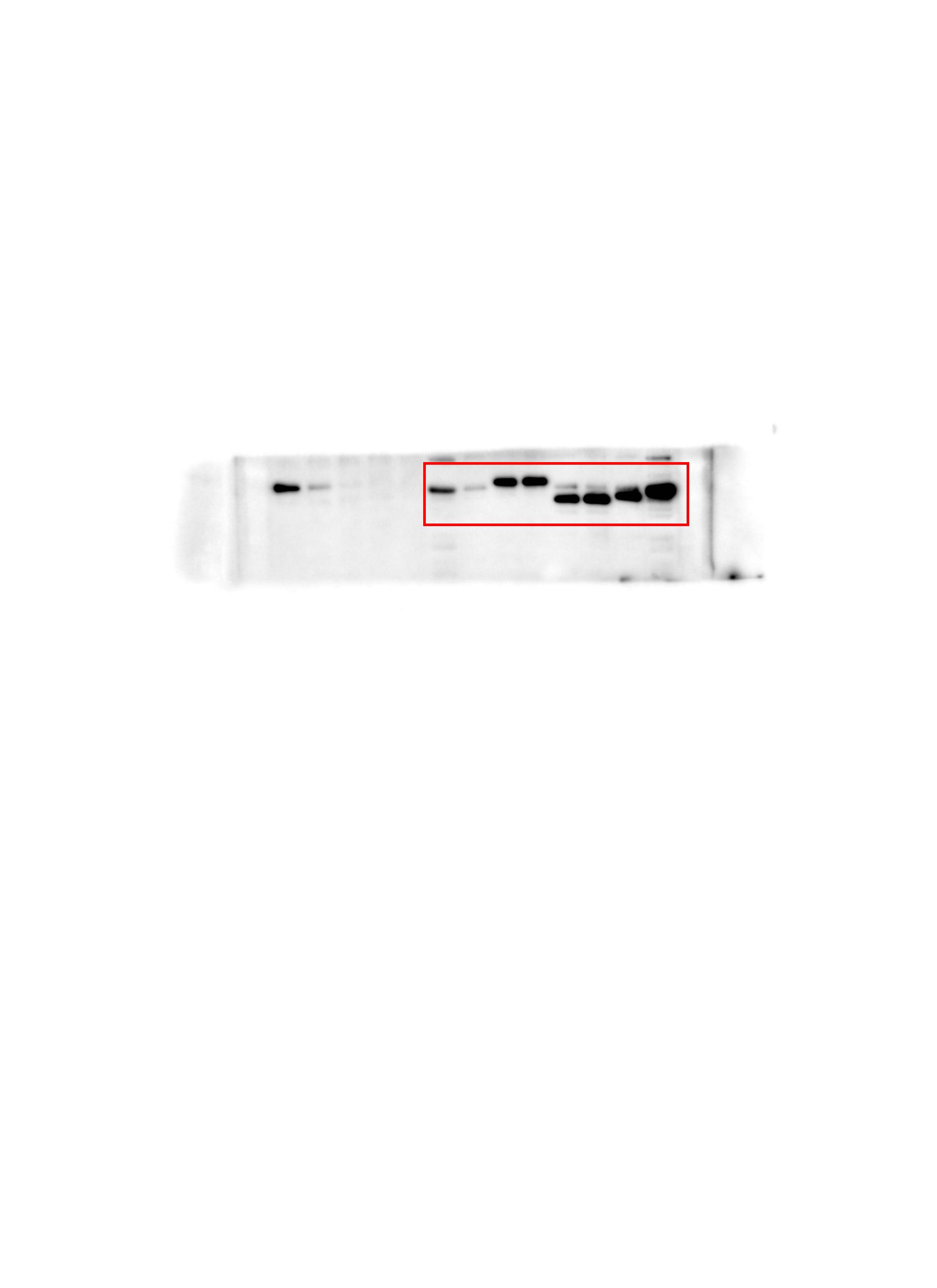

Supplement: Supplementary file 13 — Figure 3 (OLD) [file 41467_2023_42015_MOESM13_ESM.zip › Supplementary Fig. 4/Supplementary Fig. 4b/YTHDF2.jpg]

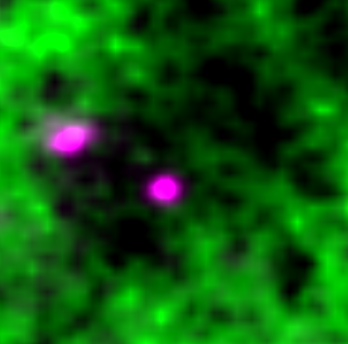

Supplement: Supplementary file 13 — Figure 3 (OLD) [file 41467_2023_42015_MOESM13_ESM.zip › Supplementary Fig. 6/SOD1(G93A)/Cell_1402_0s_zoom.png]

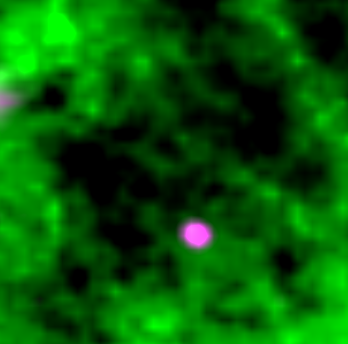

Supplement: Supplementary file 13 — Figure 3 (OLD) [file 41467_2023_42015_MOESM13_ESM.zip › Supplementary Fig. 6/SOD1(G93A)/Cell_1402_5s_zoom.png]

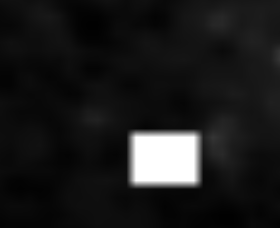

Supplement: Supplementary file 13 — Figure 3 (OLD) [file 41467_2023_42015_MOESM13_ESM.zip › Supplementary Fig. 6/SYN1/Cell_1446_1um scale bar.tif]

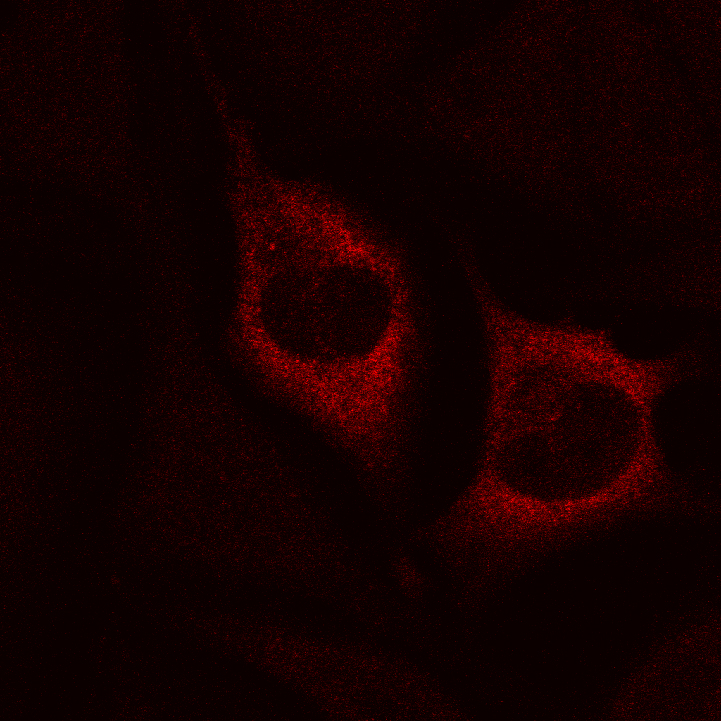

Supplement: Supplementary file 13 — Figure 3 (OLD) [file 41467_2023_42015_MOESM13_ESM.zip › Figure 1/Figure1c/508_FLAG-YTHDF2_DMSO_FLAG-YTHDF2.jpg]

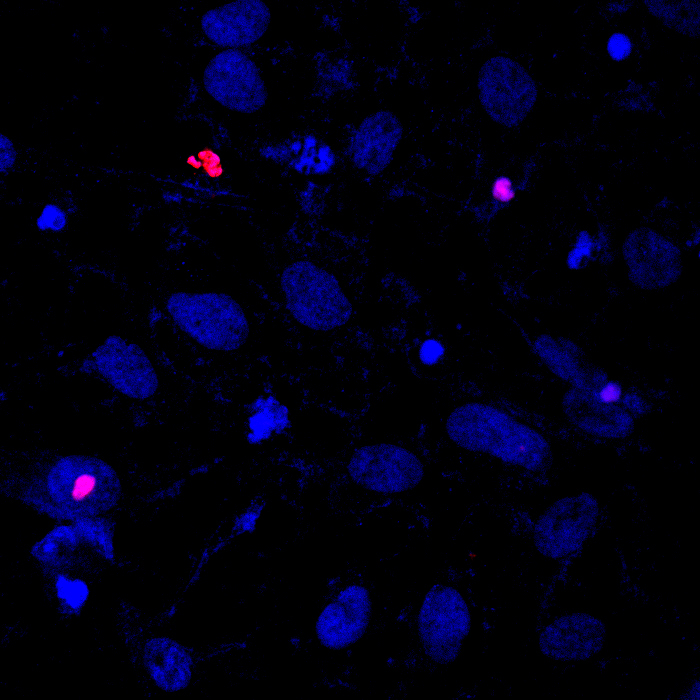

Supplement: Supplementary file 13 — Figure 3 (OLD) [file 41467_2023_42015_MOESM13_ESM.zip › Figure 5/Figure5a/YTHDF2si_Myc-YTHDF2-R527A_Merged.jpg]

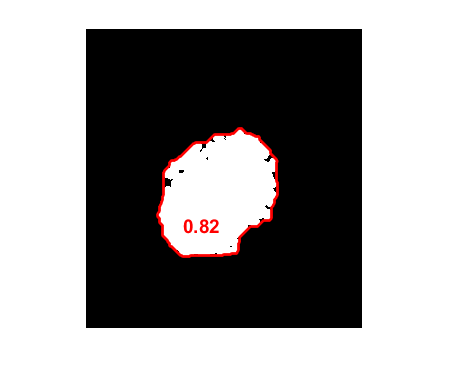

Supplement: Supplementary file 13 — Figure 3 (OLD) [file 41467_2023_42015_MOESM13_ESM.zip › Figure 6/Figure 6b/Controlsi_Dendra2-FLAG-GPx1-Ter.tif]

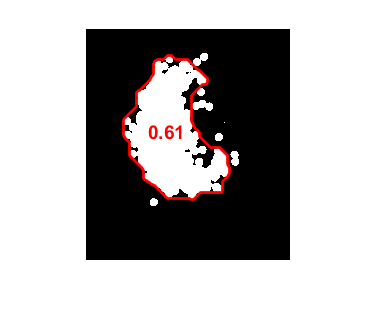

Supplement: Supplementary file 13 — Figure 3 (OLD) [file 41467_2023_42015_MOESM13_ESM.zip › Figure 6/Figure 6b/YTHDF2si_SOD(G93A)-Dendra2-FLAG.png]

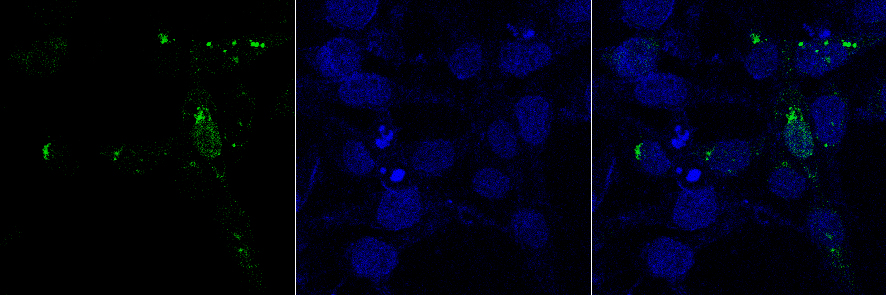

Supplement: Supplementary file 13 — Figure 3 (OLD) [file 41467_2023_42015_MOESM13_ESM.zip › Supplementary Fig. 1/Supplementary Fig. 1b/DCTN1si.jpg]

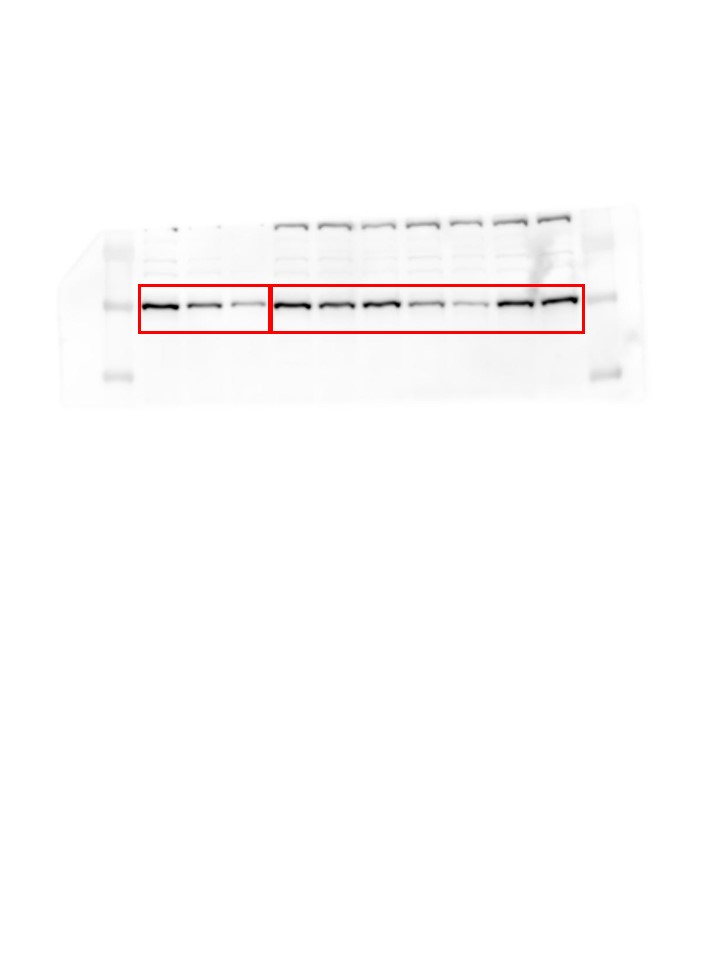

Supplement: Supplementary file 13 — Figure 3 (OLD) [file 41467_2023_42015_MOESM13_ESM.zip › Supplementary Fig. 1/Supplementary Fig. 1d/METTL14.jpg]

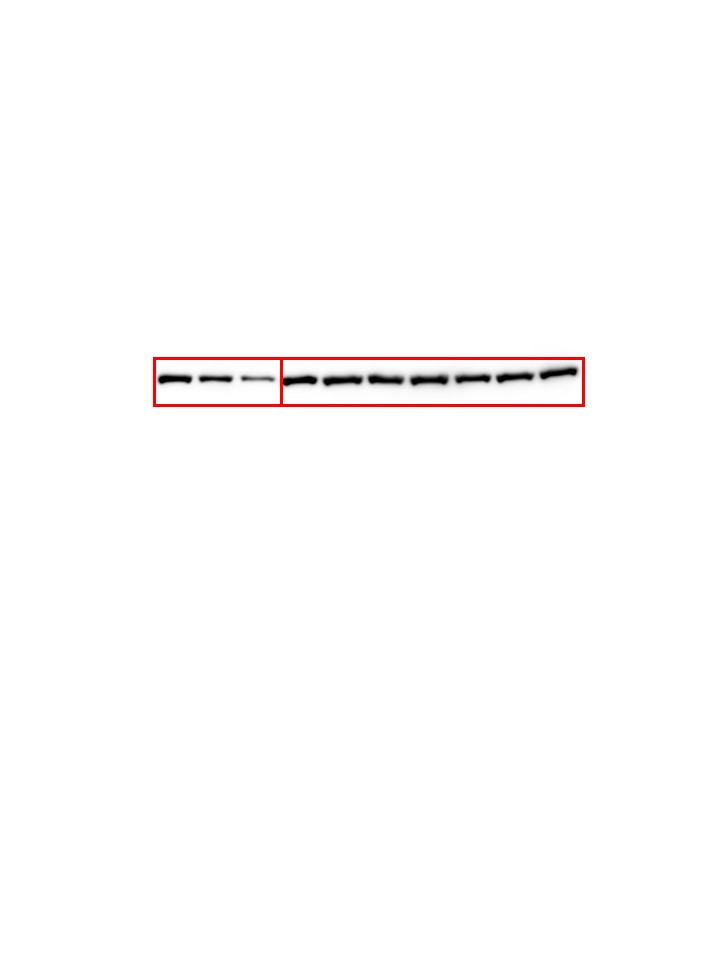

Supplement: Supplementary file 13 — Figure 3 (OLD) [file 41467_2023_42015_MOESM13_ESM.zip › Supplementary Fig. 1/Supplementary Fig. 1d/b-actin.jpg]

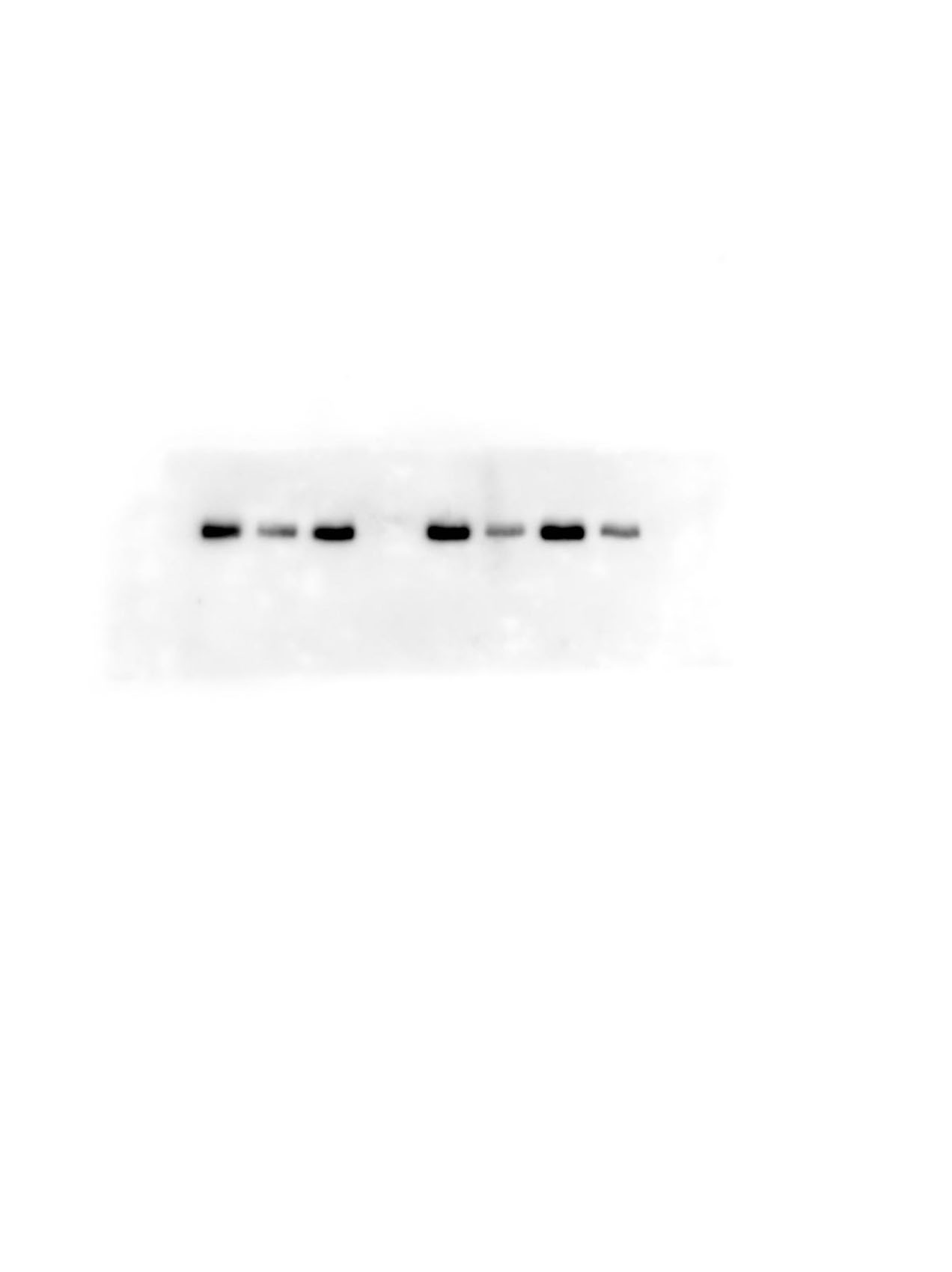

Supplement: Supplementary file 13 — Figure 3 (OLD) [file 41467_2023_42015_MOESM13_ESM.zip › Supplementary Fig. 2/Supplementary Fig. 2c/Tubulin.jpg]

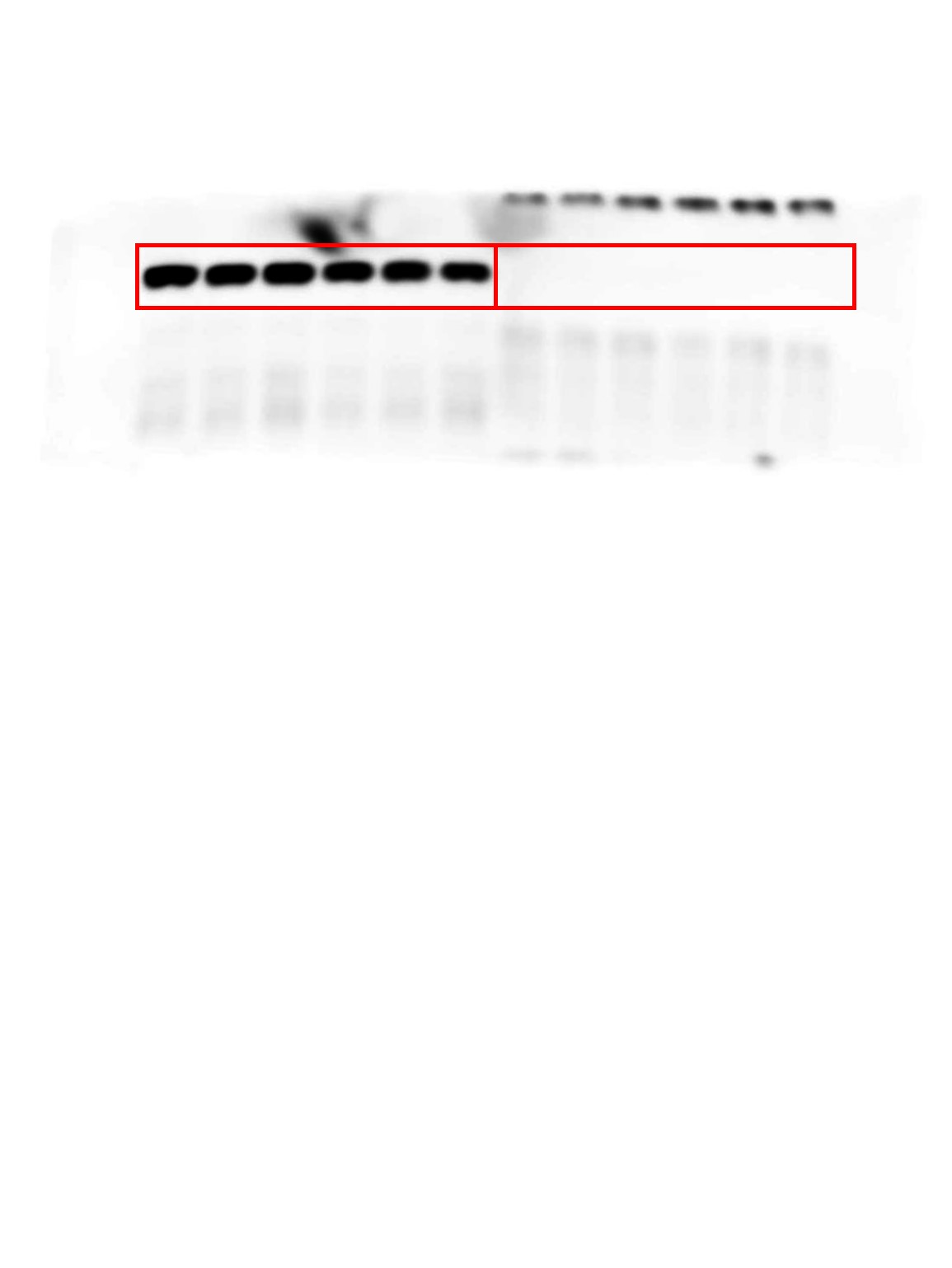

Supplement: Supplementary file 13 — Figure 3 (OLD) [file 41467_2023_42015_MOESM13_ESM.zip › Supplementary Fig. 3/Supplementary Fig. 3a/b-actin.jpg]

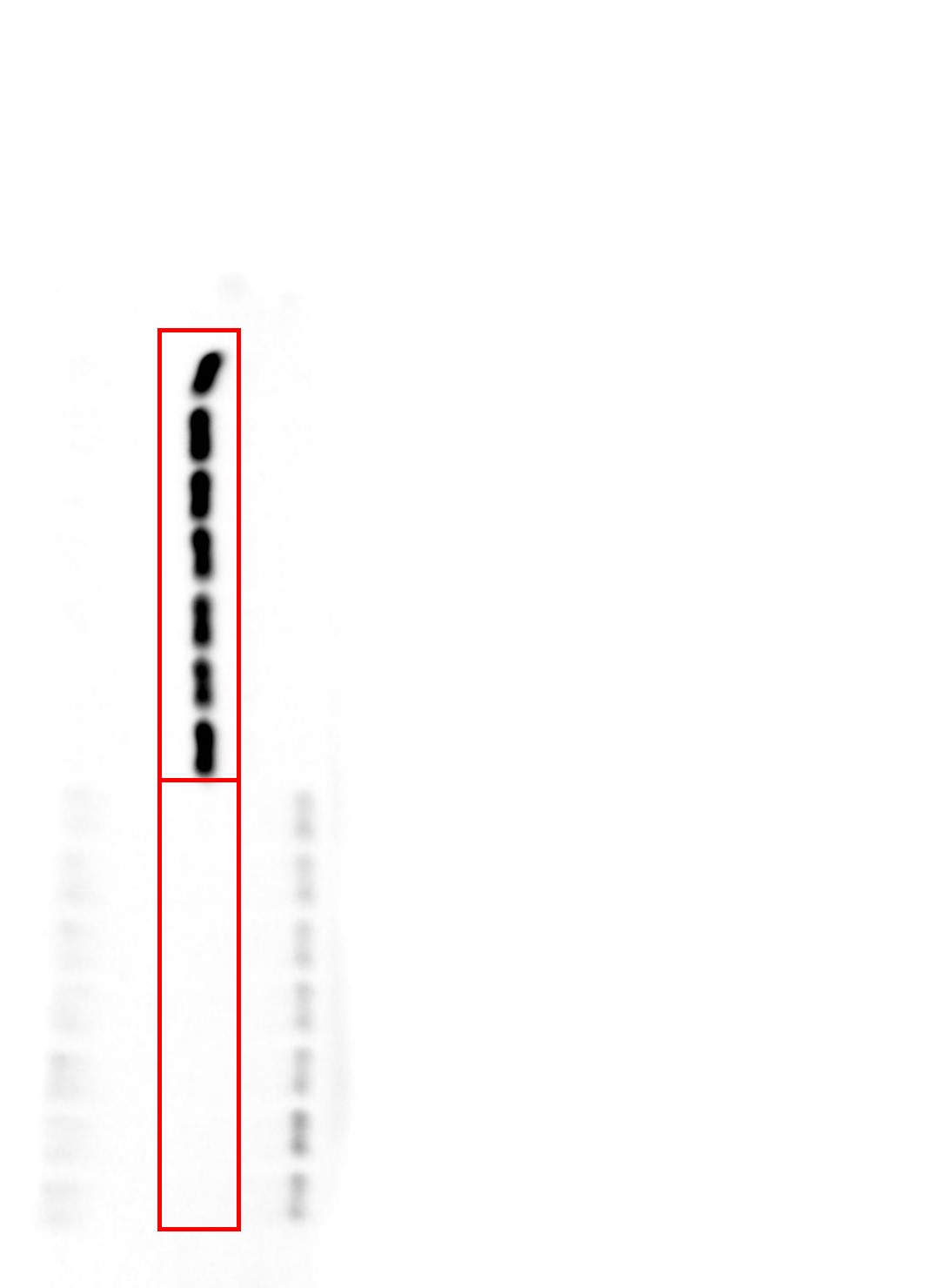

Supplement: Supplementary file 13 — Figure 3 (OLD) [file 41467_2023_42015_MOESM13_ESM.zip › Supplementary Fig. 3/Supplementary Fig. 3b/b-actin.jpg]

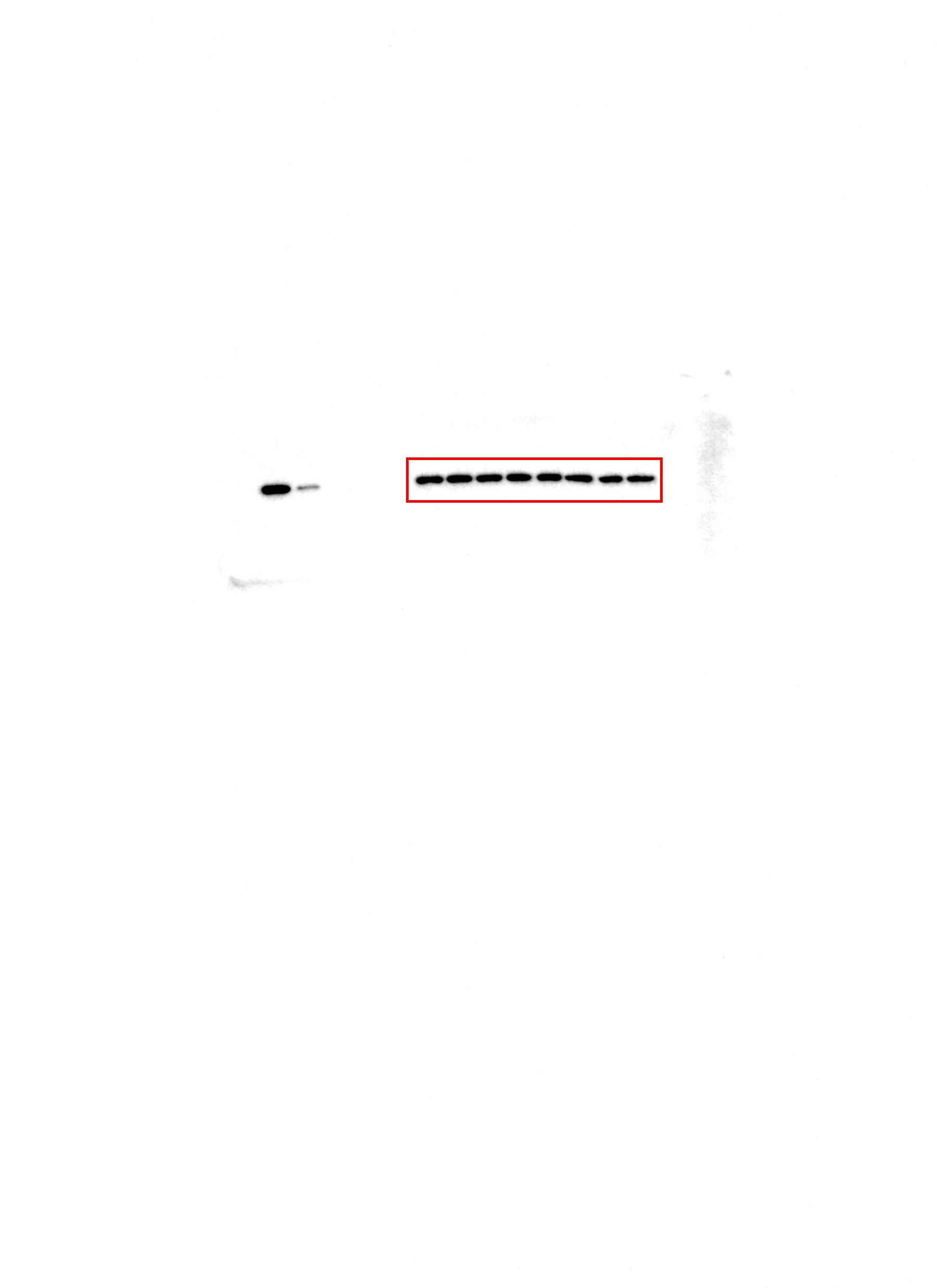

Supplement: Supplementary file 13 — Figure 3 (OLD) [file 41467_2023_42015_MOESM13_ESM.zip › Supplementary Fig. 4/Supplementary Fig. 4b/b-actin.jpg]

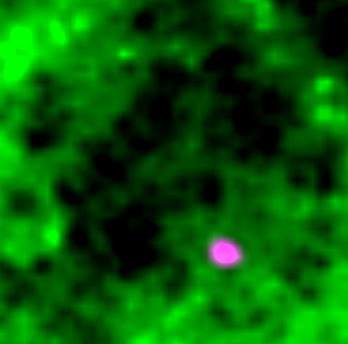

Supplement: Supplementary file 13 — Figure 3 (OLD) [file 41467_2023_42015_MOESM13_ESM.zip › Supplementary Fig. 6/SOD1(G93A)/Cell_1402_24s_zoom.png]

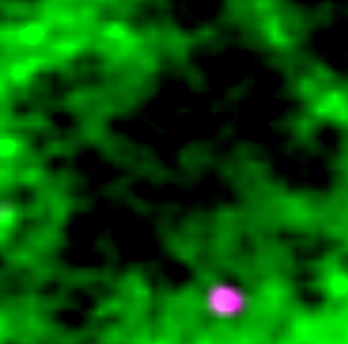

Supplement: Supplementary file 13 — Figure 3 (OLD) [file 41467_2023_42015_MOESM13_ESM.zip › Supplementary Fig. 6/SOD1(G93A)/Cell_1402_36s_zoom.png]

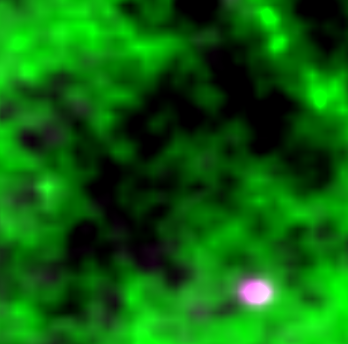

Supplement: Supplementary file 13 — Figure 3 (OLD) [file 41467_2023_42015_MOESM13_ESM.zip › Supplementary Fig. 6/SOD1(G93A)/Cell_1402_40s_zoom.png]

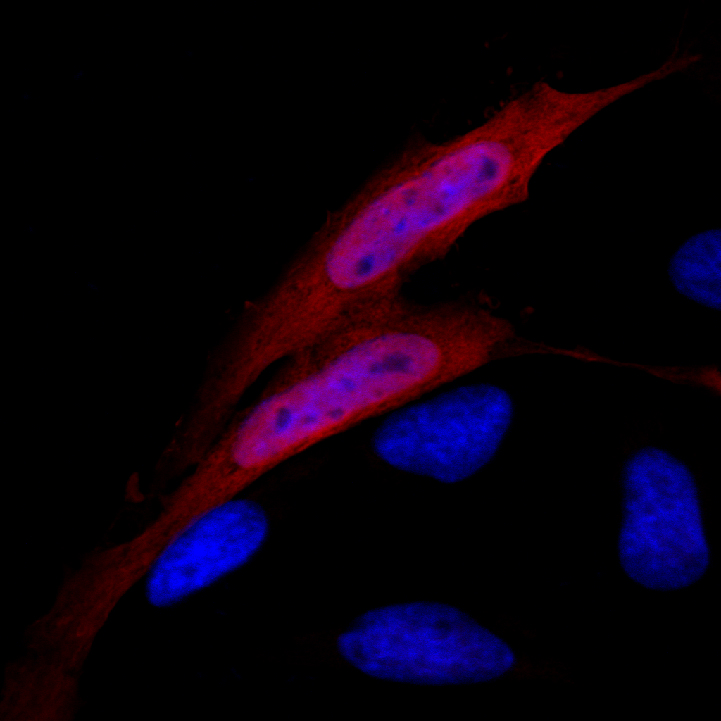

Supplement: Supplementary file 13 — Figure 3 (OLD) [file 41467_2023_42015_MOESM13_ESM.zip › Figure 1/Figure1c/508_FLAG-YTHDF2-Cterm_DMSO_merged.jpg]

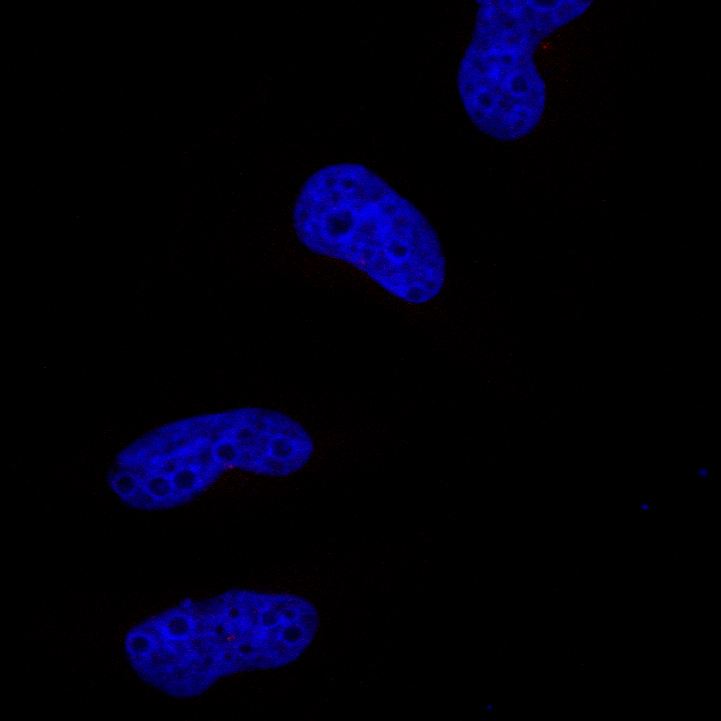

Supplement: Supplementary file 13 — Figure 3 (OLD) [file 41467_2023_42015_MOESM13_ESM.zip › Figure 1/Figure1c/508_FLAG-YTHDF2-Nterm_DMSO_merged.jpg]

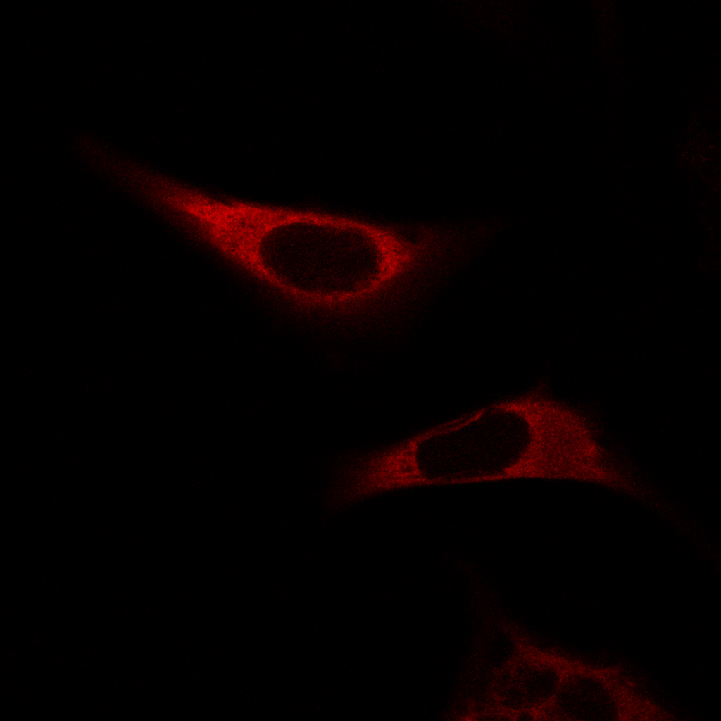

Supplement: Supplementary file 13 — Figure 3 (OLD) [file 41467_2023_42015_MOESM13_ESM.zip › Figure 1/Figure1c/508_FLAG-YTHDF2_MG132_FLAG-YTHDF2.jpg]

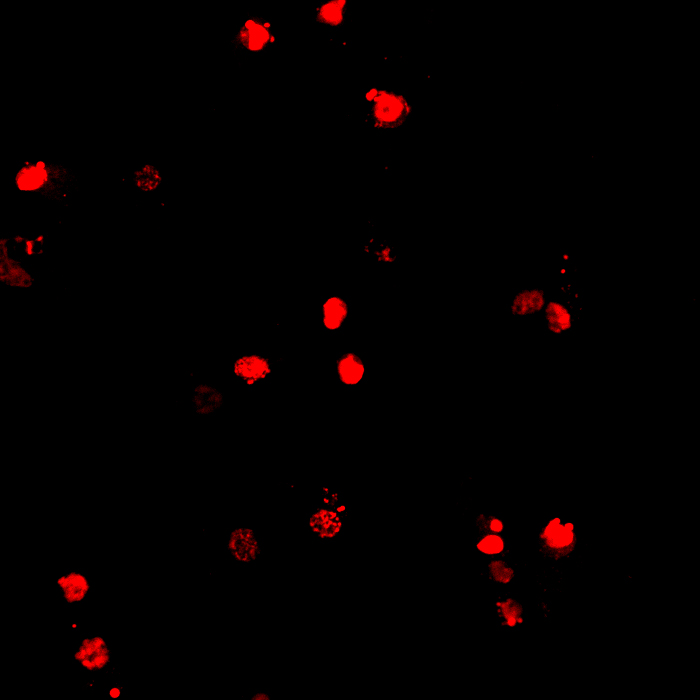

Supplement: Supplementary file 13 — Figure 3 (OLD) [file 41467_2023_42015_MOESM13_ESM.zip › Figure 5/Figure5a/YTHDF2si_Myc-YTHDF2-101-168_TUNEL.jpg]

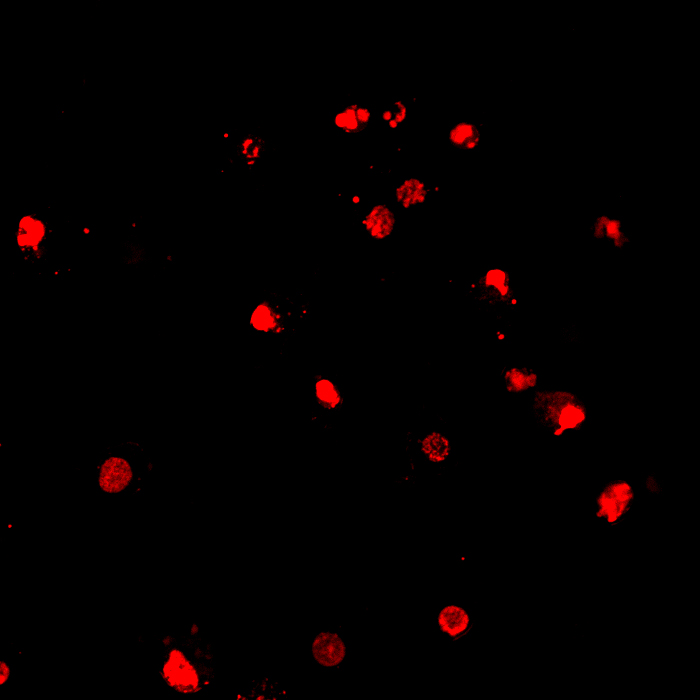

Supplement: Supplementary file 13 — Figure 3 (OLD) [file 41467_2023_42015_MOESM13_ESM.zip › Figure 5/Figure5a/YTHDF2si_Myc-YTHDF2-101-200_TUNEL.jpg]

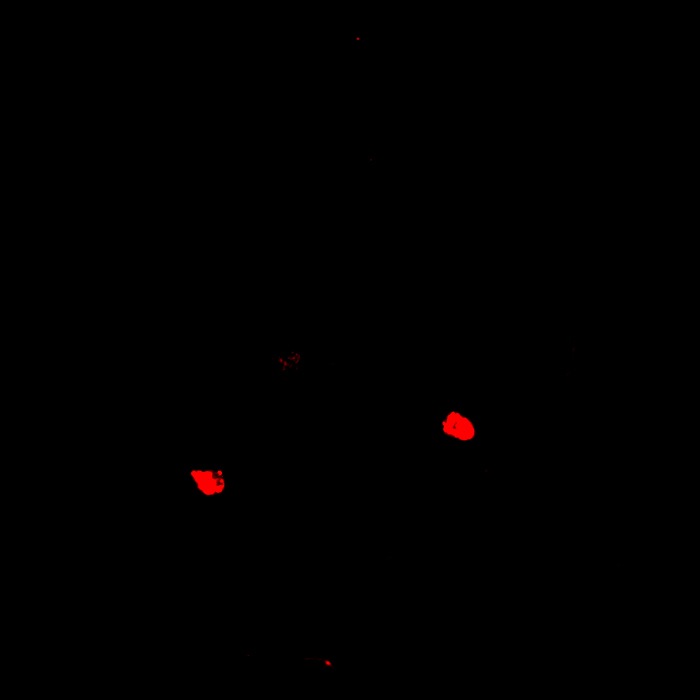

Supplement: Supplementary file 13 — Figure 3 (OLD) [file 41467_2023_42015_MOESM13_ESM.zip › Figure 5/Figure5a/YTHDF2si_Myc-YTHDF2-169-200_TUNEL.jpg]

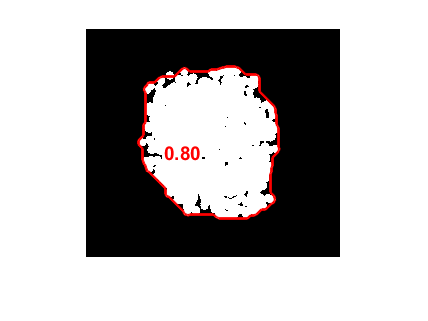

Supplement: Supplementary file 13 — Figure 3 (OLD) [file 41467_2023_42015_MOESM13_ESM.zip › Figure 6/Figure 6b/Controlsi_SOD(G93A)-Dendra2-FLAG.tif]

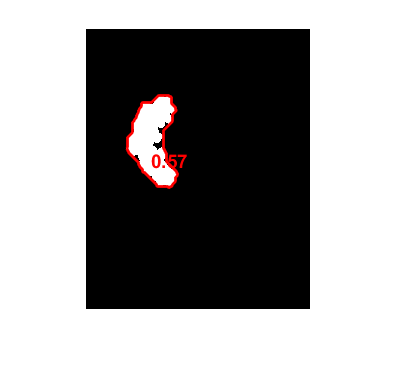

Supplement: Supplementary file 13 — Figure 3 (OLD) [file 41467_2023_42015_MOESM13_ESM.zip › Figure 6/Figure 6b/YTHDF2si_Synphilin1-Dendra2-FLAG.png]

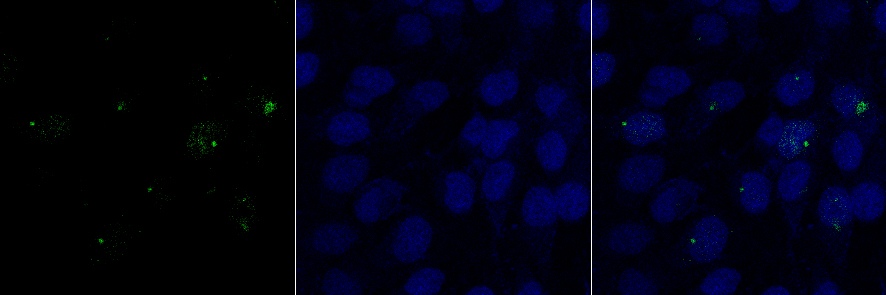

Supplement: Supplementary file 13 — Figure 3 (OLD) [file 41467_2023_42015_MOESM13_ESM.zip › Supplementary Fig. 1/Supplementary Fig. 1b/METTL3si.jpg]

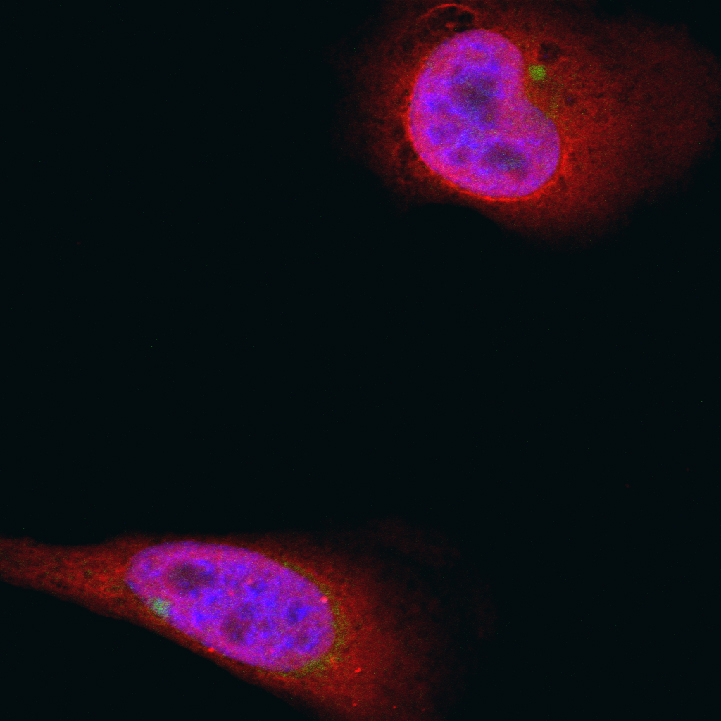

Supplement: Supplementary file 13 — Figure 3 (OLD) [file 41467_2023_42015_MOESM13_ESM.zip › Figure 1/Figure1c/508_FLAG-YTHDF2-Cterm_MG132_merged.jpg]

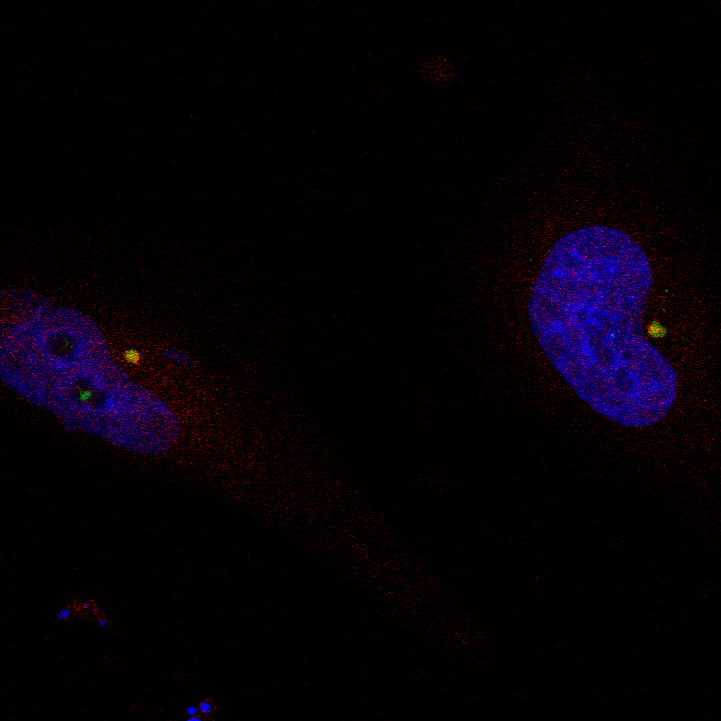

Supplement: Supplementary file 13 — Figure 3 (OLD) [file 41467_2023_42015_MOESM13_ESM.zip › Figure 1/Figure1c/508_FLAG-YTHDF2-Nterm_MG132_merged.jpg]

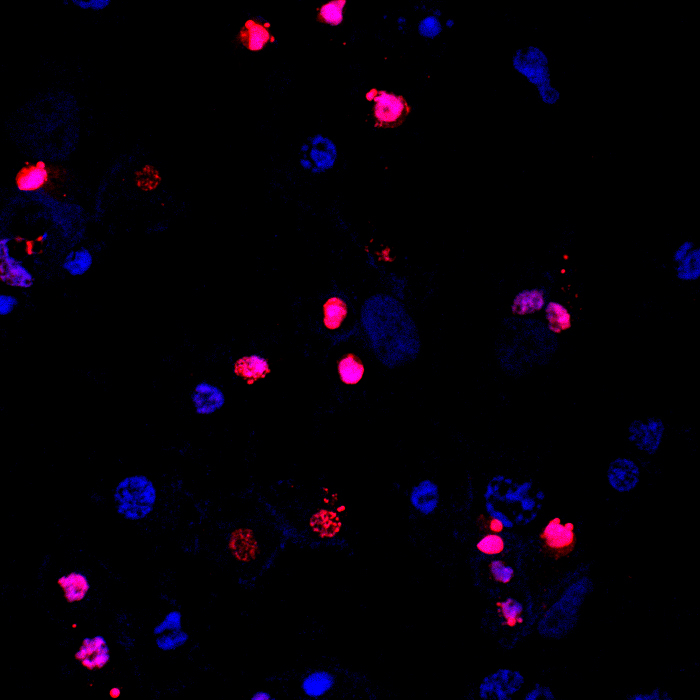

Supplement: Supplementary file 13 — Figure 3 (OLD) [file 41467_2023_42015_MOESM13_ESM.zip › Figure 5/Figure5a/YTHDF2si_Myc-YTHDF2-101-168_Merged.jpg]

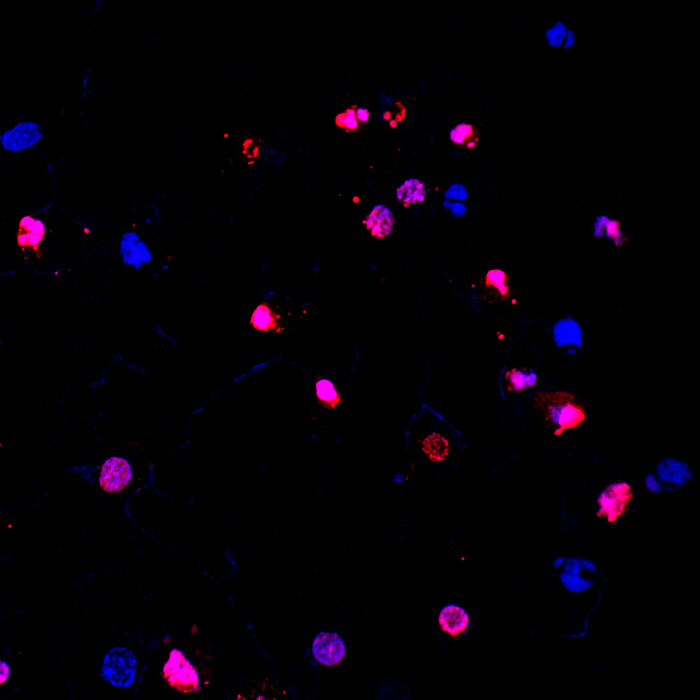

Supplement: Supplementary file 13 — Figure 3 (OLD) [file 41467_2023_42015_MOESM13_ESM.zip › Figure 5/Figure5a/YTHDF2si_Myc-YTHDF2-101-200_Merged.jpg]

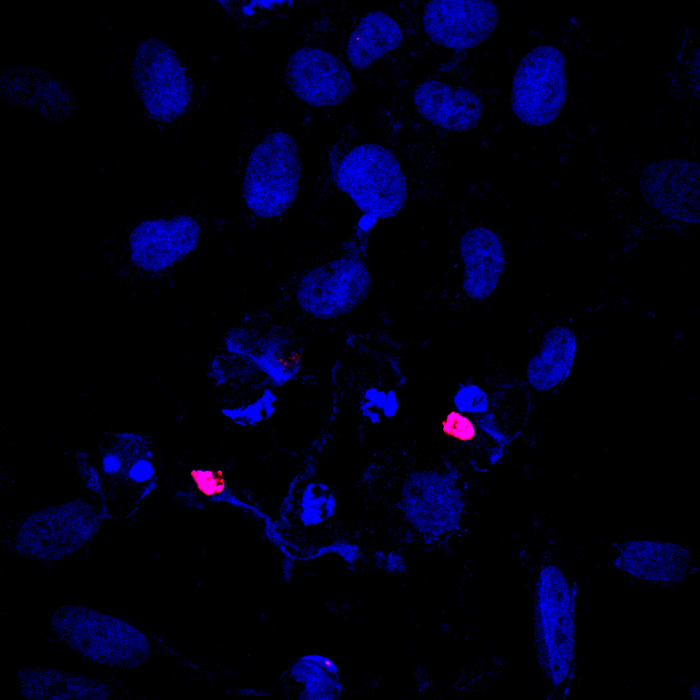

Supplement: Supplementary file 13 — Figure 3 (OLD) [file 41467_2023_42015_MOESM13_ESM.zip › Figure 5/Figure5a/YTHDF2si_Myc-YTHDF2-169-200_Merged.jpg]

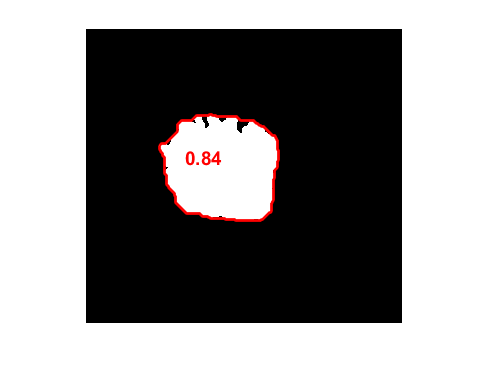

Supplement: Supplementary file 13 — Figure 3 (OLD) [file 41467_2023_42015_MOESM13_ESM.zip › Figure 6/Figure 6b/Controlsi_Synphilin1-Dendra2-FLAG.png]

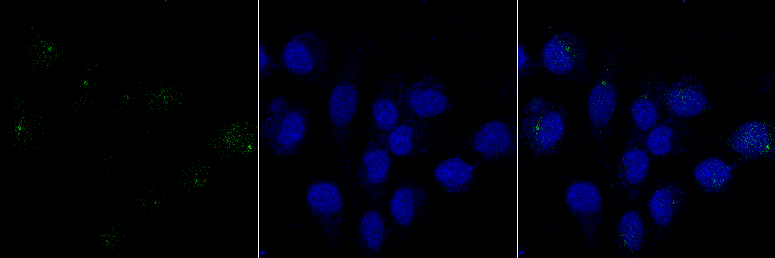

Supplement: Supplementary file 13 — Figure 3 (OLD) [file 41467_2023_42015_MOESM13_ESM.zip › Supplementary Fig. 1/Supplementary Fig. 1b/Controlsi.jpg]

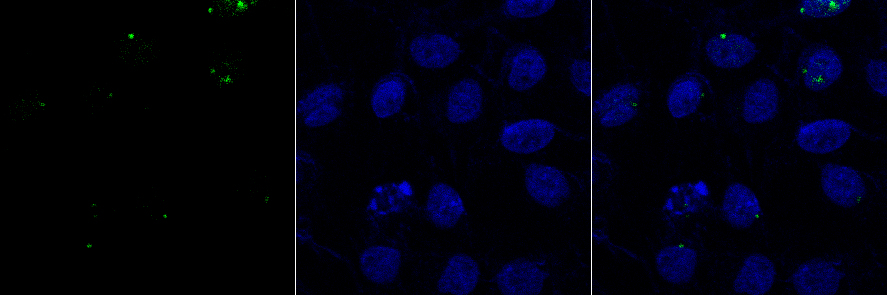

Supplement: Supplementary file 13 — Figure 3 (OLD) [file 41467_2023_42015_MOESM13_ESM.zip › Supplementary Fig. 1/Supplementary Fig. 1b/METTL14si.jpg]

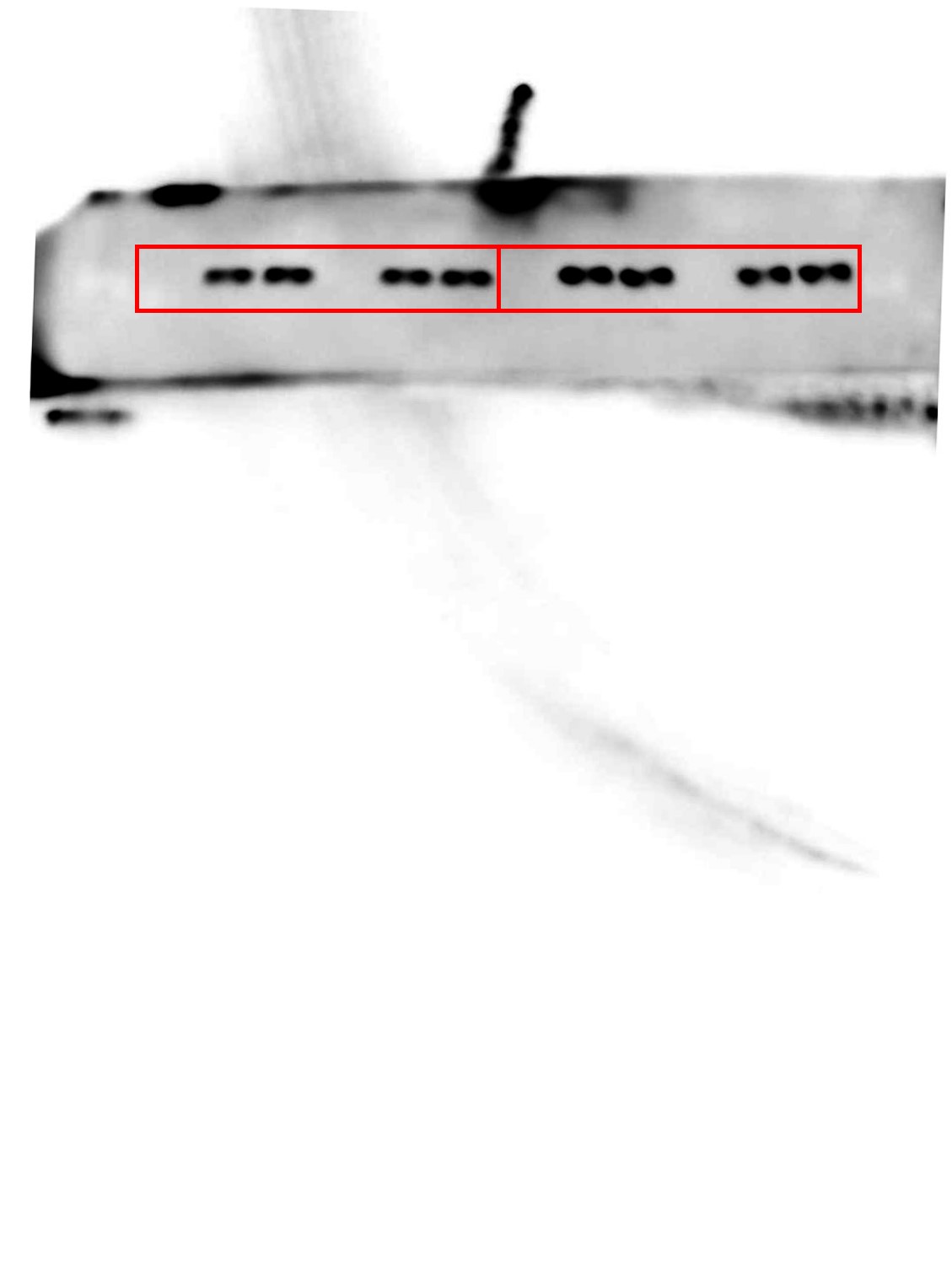

Supplement: Supplementary file 13 — Figure 3 (OLD) [file 41467_2023_42015_MOESM13_ESM.zip › Supplementary Fig. 3/Supplementary Fig. 3a/FLAG-CTIF.jpg]

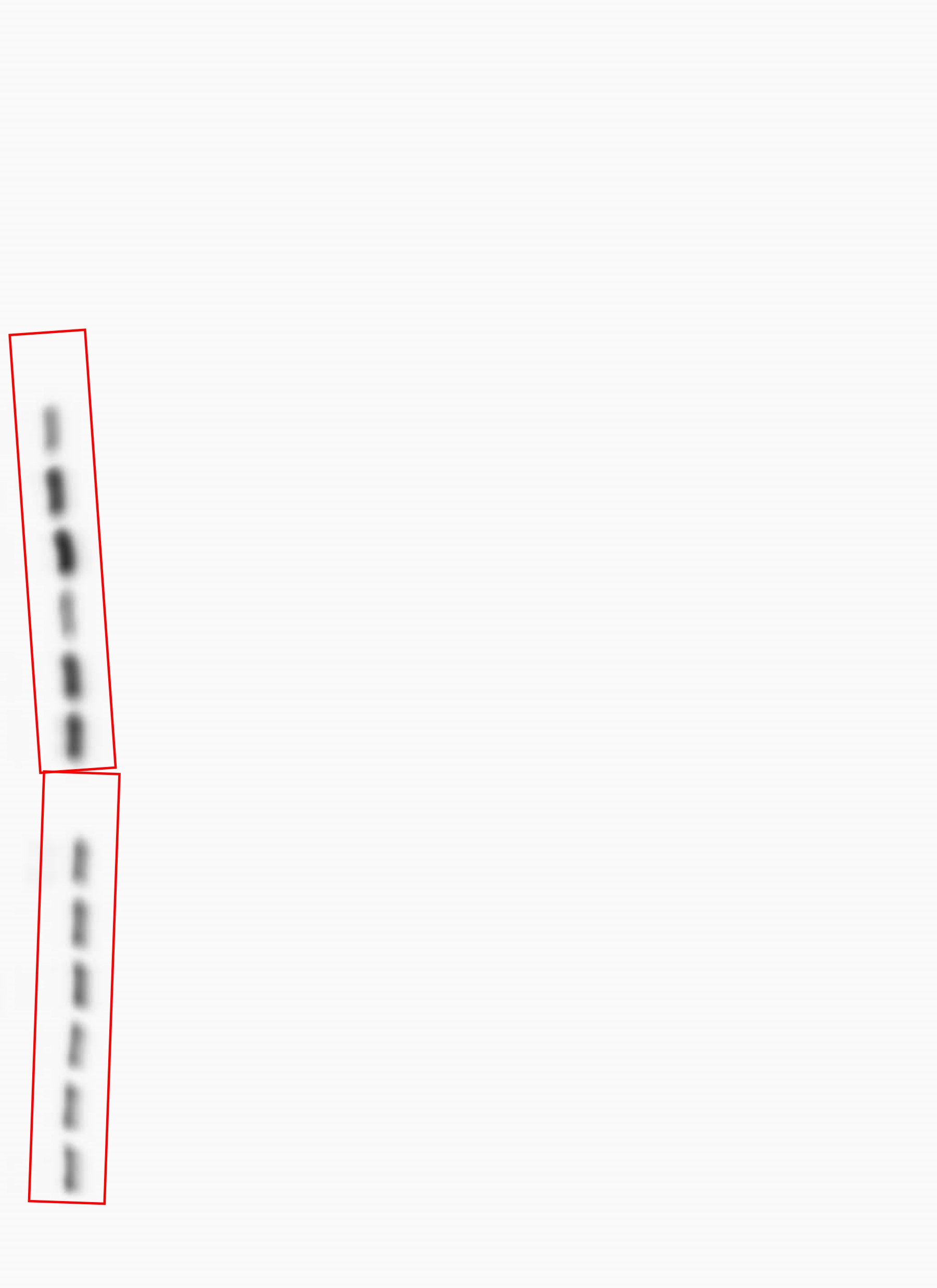

Supplement: Supplementary file 13 — Figure 3 (OLD) [file 41467_2023_42015_MOESM13_ESM.zip › Supplementary Fig. 3/Supplementary Fig. 3b/FLAG-UPF1.jpg]

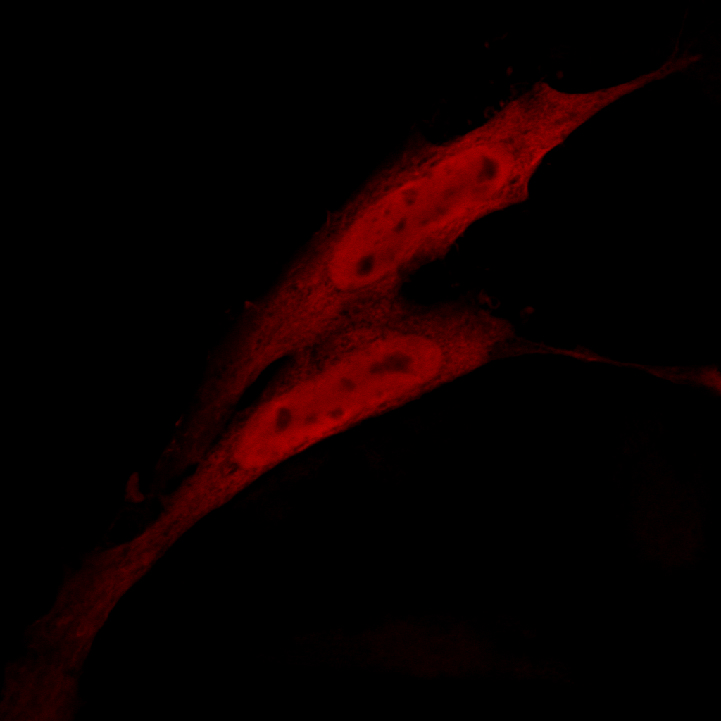

Supplement: Supplementary file 13 — Figure 3 (OLD) [file 41467_2023_42015_MOESM13_ESM.zip › Figure 1/Figure1c/508_FLAG-YTHDF2-Cterm_DMSO_YTHDF2 C.jpg]

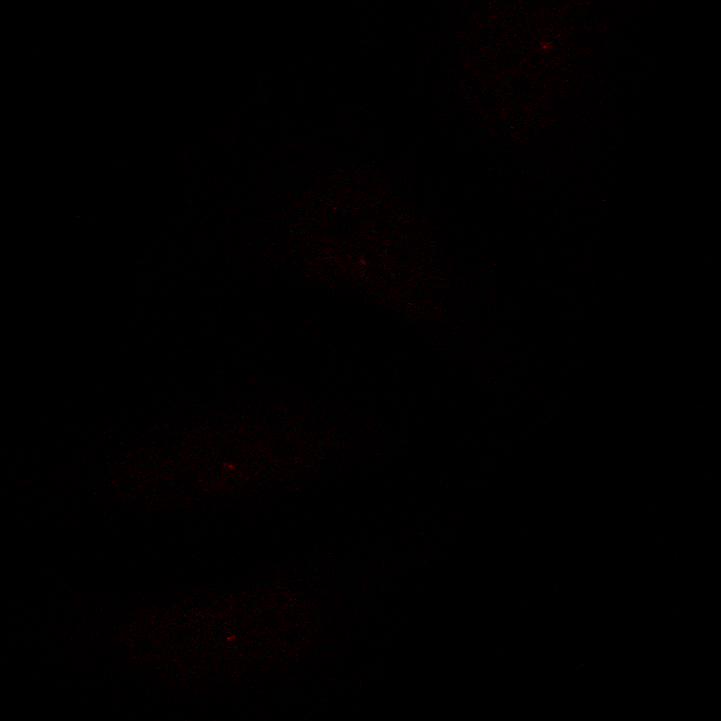

Supplement: Supplementary file 13 — Figure 3 (OLD) [file 41467_2023_42015_MOESM13_ESM.zip › Figure 1/Figure1c/508_FLAG-YTHDF2-Nterm_DMSO_YTHDF2 N.jpg]

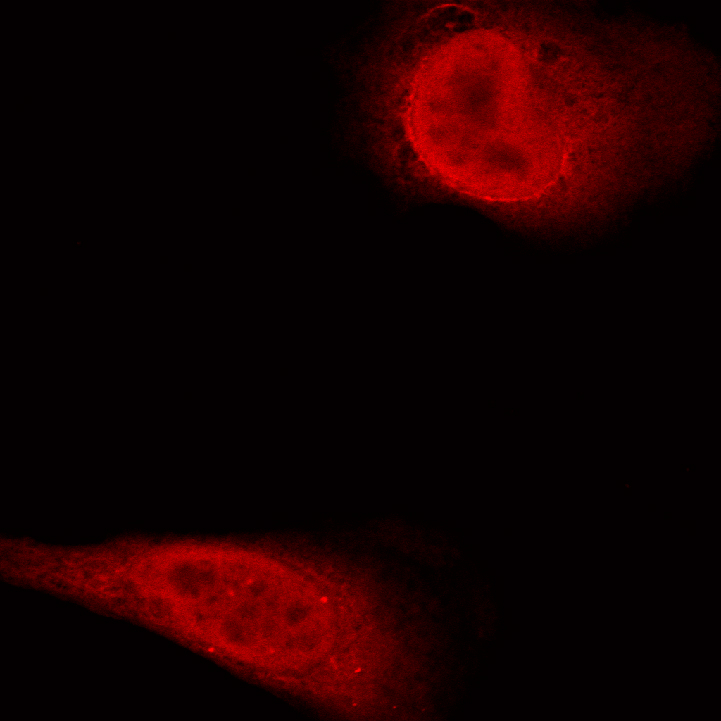

Supplement: Supplementary file 13 — Figure 3 (OLD) [file 41467_2023_42015_MOESM13_ESM.zip › Figure 1/Figure1c/508_FLAG-YTHDF2-Cterm_MG132_YTHDF2 C.jpg]

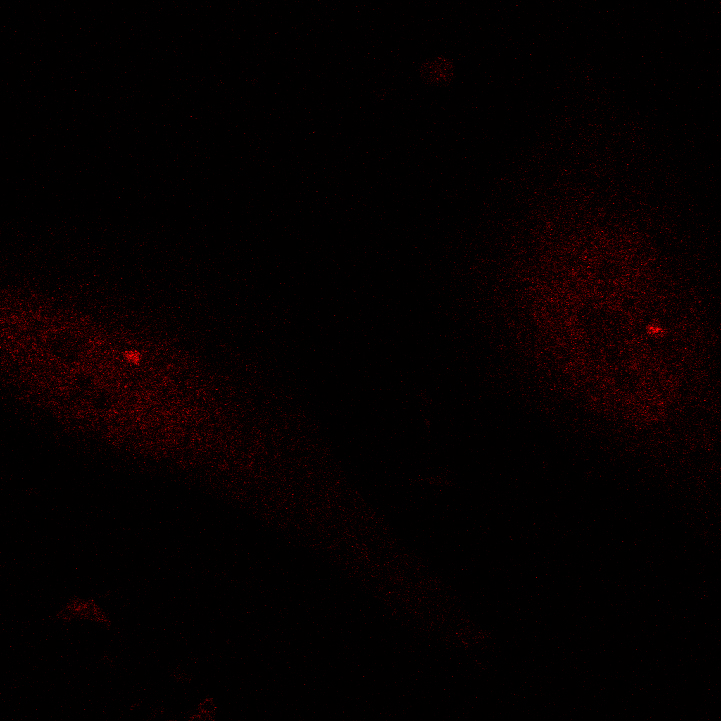

Supplement: Supplementary file 13 — Figure 3 (OLD) [file 41467_2023_42015_MOESM13_ESM.zip › Figure 1/Figure1c/508_FLAG-YTHDF2-Nterm_MG132_YTHDF2 N.jpg]
